# Orally Bioavailable and Site-Selective Covalent STING Inhibitor Derived from a Macrocyclic Marine Diterpenoid

**DOI:** 10.1021/acs.jmedchem.4c02665

**Published:** 2025-02-27

**Authors:** Guang-Hao Niu, Wan-Chi Hsiao, Po-Hsun Lee, Li-Guo Zheng, Yu-Shao Yang, Wei-Cheng Huang, Chih-Chien Hsieh, Tai-Yu Chiu, Jing-Ya Wang, Ching-Ping Chen, Chen-Lung Huang, May-Su You, Yi-Ping Kuo, Chien-Ming Wang, Zhi-Hong Wen, Guann-Yi Yu, Chiung-Tong Chen, Ya-Hui Chi, Chun-Wei Tung, Shu-Ching Hsu, Teng-Kuang Yeh, Ping-Jyun Sung, Mingzi M. Zhang, Lun Kelvin Tsou

**Affiliations:** † Institute of Biotechnology and Pharmaceutical Research, National Health Research Institutes, Zhunan, Miaoli 35053, Taiwan; ‡ Institute of Molecular and Genomic Medicine, National Health Research Institutes, Zhunan, Miaoli 35053, Taiwan; § National Institute of Infectious Diseases and Vaccinology, National Health Research Institutes, Zhunan, Miaoli 35053, Taiwan; ∥ Institute of Biotechnology, National Tsing Hua University, Hsinchu 30013, Taiwan; ⊥ Department of Marine Biotechnology and Resources, National Sun Yat-Sen University, Kaohsiung 804201, Taiwan; ¶ National Museum of Marine Biology and Aquarium, Pingtung 944401, Taiwan

## Abstract

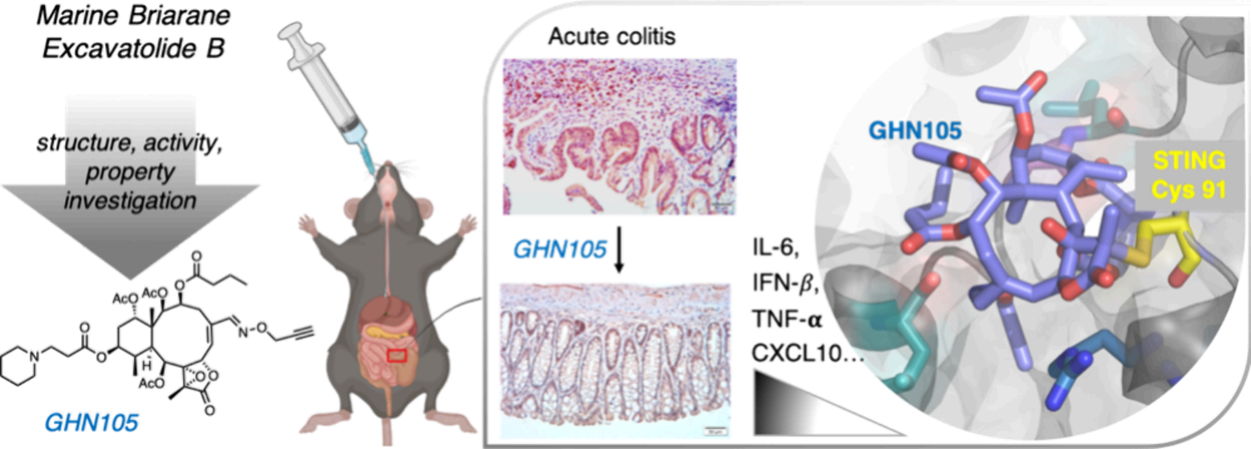

Pharmacological inhibition of the cGAS-STING-controlled innate immune pathway is an emerging therapeutic strategy for a myriad of inflammatory diseases. Here, we report **GHN105** as an orally bioavailable covalent STING inhibitor. Late-stage diversification of the briarane-type diterpenoid excavatolide B allowed the installation of solubility-enhancing functional groups while enhancing its activity as a covalent STING inhibitor against multiple human STING variants, including the S154 variant responsible for a genetic autoimmune disease. Selectively engaging the membrane-proximal Cys91 residue of STING, **GHN105** dose-dependently inhibited cGAS-STING signaling and type I interferon responses in cells and in vivo. Moreover, orally administered **GHN105** exhibited on-target engagement in vivo and markedly reversed key pathological features in a delayed treatment of the acute colitis mouse model. Our study provided proof of concept that the synthetic briarane analog **GHN105** serves as a safe, site-selective, and orally active covalent STING inhibitor and devises a regimen that allows long-term systemic administration.

## Introduction

The cyclic GMP-AMP synthase (cGAS)-stimulator of interferon genes (STING) signaling cascade orchestrates innate immunity in response to intracellular nucleic acids.^1^ While activation of the cGAS-STING pathway to bolster antitumor immunity has been evaluated as an immunotherapy strategy,^2^ excessive STING activation is linked to various autoimmune diseases such as STING-associated vasculopathy in infants (SAVI), systemic lupus erythematosus, Aicardi–Goutières syndrome, and ataxia-telangiectasia.^3^ Moreover, the cGAS-STING pathway has been established as a driver of inflammatory diseases in many peripheral organs and aging-related neurodegeneration.^4^ Efforts to antagonize pathological STING signaling or type I interferon (IFN) associated inflammations have yielded small molecules or peptides that target its ligand-binding pocket as well as the post-translational modification sites that regulate STING activation and stability.^5−7^ Yet, limitations on pharmacokinetic (PK) properties and chemotypes have hindered the development of site-selective, efficient, and orally bioavailable STING inhibitors.

Natural products have long served as a source of drug discovery and continue to inspire the development of novel therapies.^8,9^ In particular, marine natural products have demonstrated significant progress, with several compounds successfully entering clinical trials or gaining approval.^8,10^ Marine-derived anti-inflammatory pharmacophores, such as cyclic peptides,^11^ meroterpenoids,^12^ polyketides,^13^ alkaloids,^14^ and diterpenoids,^15^ have provided diverse platform for new therapeutic strategies. Indeed, through chemoproteomic profiling, our previous study has identified that marine diterpenoid excavatolide B (ExcB) from gorgonian octocorals as a new class of covalent STING inhibitors.^16^ Harnessing an embedded epoxy-lactone warhead, ExcB is structurally distinct from the chloroacetamidine, nitroalkene, nitrofuran, and acrylamide warheads of other covalent STING inhibitors reported to date.^6,17^ Despite >700 briarane diterpenoids have been identified to date, systematic structural diversification to elucidate their structure–activity/property relationship (SAR/SPR) and to optimize their pharmacological properties remained elusive.^18^ Progress on several synthetic endeavors toward the bioactive briarane-type diterpenoids was reported, but no complete total synthesis was reported as a significant technical bottleneck exists with the installation of epoxy-lactone to the briarane skeleton.^19^

Late-stage chemical modifications allow for the *stereo-* or *regio-*selective alteration of reactive sites while preserving the core structure of the molecule and have emerged as valuable strategies to optimize the pharmacokinetic and pharmacodynamic properties of natural products. For example, artemether and artesunate were developed through a late-stage modification to overcome artemisinin’s short half-life.^20^ Similarly, the esterification of ingenol, a diterpenoid from *Euphorbia ingens*, led to the development of ingenol mebutate, a prodrug used for treating actinic keratosis.^21^ In this study, to extend the scope from our previous finding, we leveraged the embedded electrophilic epoxy-lactone warhead in ExcB as a new chemotype for covalent inhibitor design and synthesized derivatives using the late-stage diversification strategy to optimize its STING inhibitory activity. Concurrently, the incorporated drug-like appendages significantly increased its solubility and facilitated in vivo systemic exposure from oral administration. Mechanistically, through the clickable handle and site-directed mutagenesis, we showed that **GHN105** selectively engaged with STING through Cys91, a conserved post-translational modification site for STING activation. Insights from the in vivo pharmacokinetic and toxicity studies then culminated a safe and oral delivery regime of **GHN105** to significantly reduce the production of downstream type-I interferons and proinflammatory cytokines. We demonstrated that this site-selective and orally active covalent inhibitor **GHN105** can reverse key pathogenic features in an acute colitis model and validated in situ STING engagement by **GHN105** in the colons of the mice that received the treatment. Collectively, our findings underpin a new treatment strategy for chronic inflammatory diseases associated with the overactivation of STING signaling.

## Results and Discussion

### Design and Synthesis of Briarane Ditepenoid Derivatives

**Figure 1:**
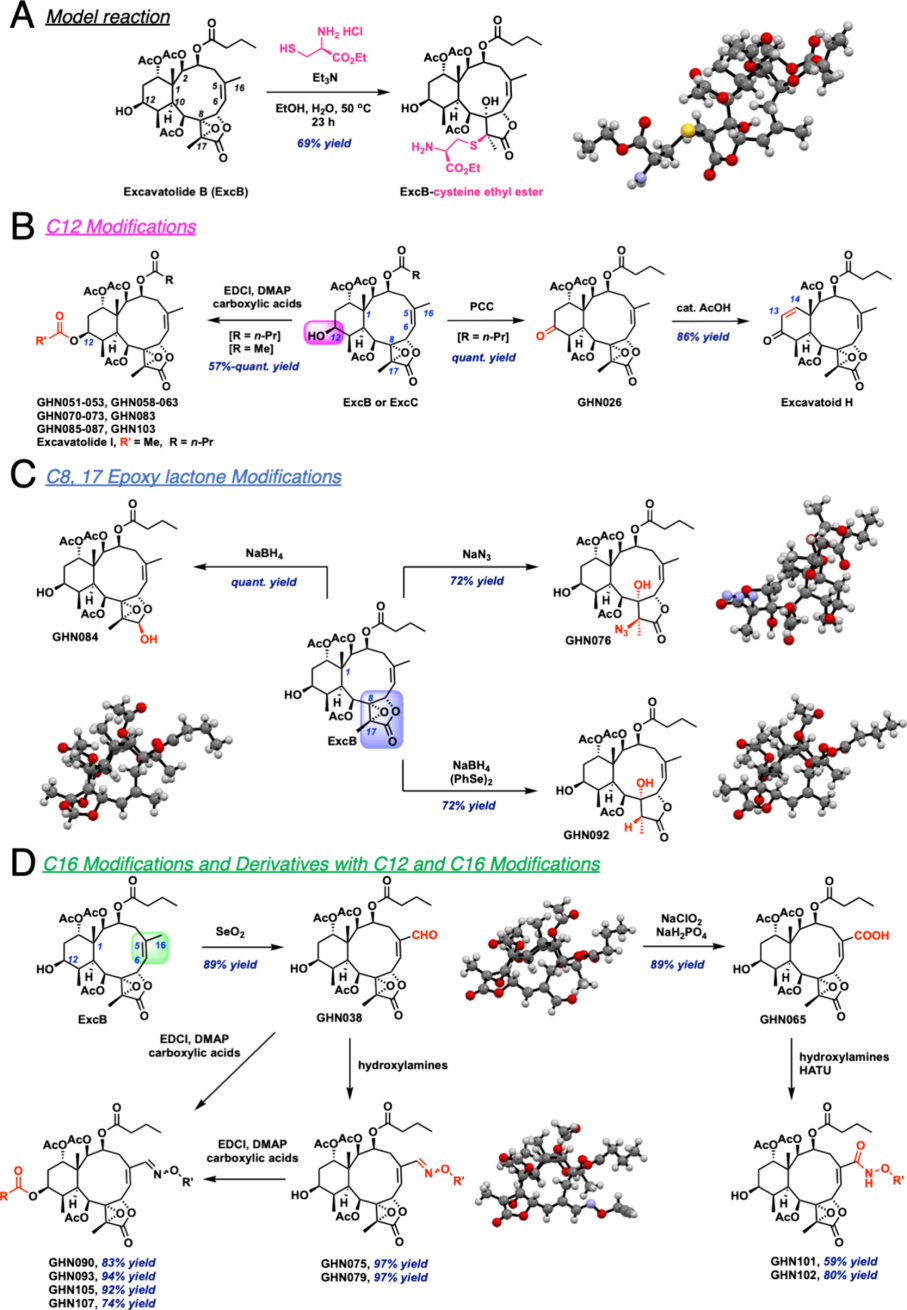
Design and synthetic routes of ExcB analogs (GHN series and the structures of each analog were shown in Figure 2A. X-ray crystal structures of nucleophilic addition product and several analogs were obtained, and analysis was reported in Tables S1–S8. (A) Model reaction of ExcB with cysteine and X-ray confirmation of nucleophilic addition product. (B) Reactions to obtain other briarane natural products and analogs with C12 modifications. (C) Scheme of transformations at the epoxy lactone portion and X-ray crystals of the respective analogs. (D) Representative reactions carried out at C16 and synthetic routes for dual modifications on C12 and C16.

**Figure 2:**
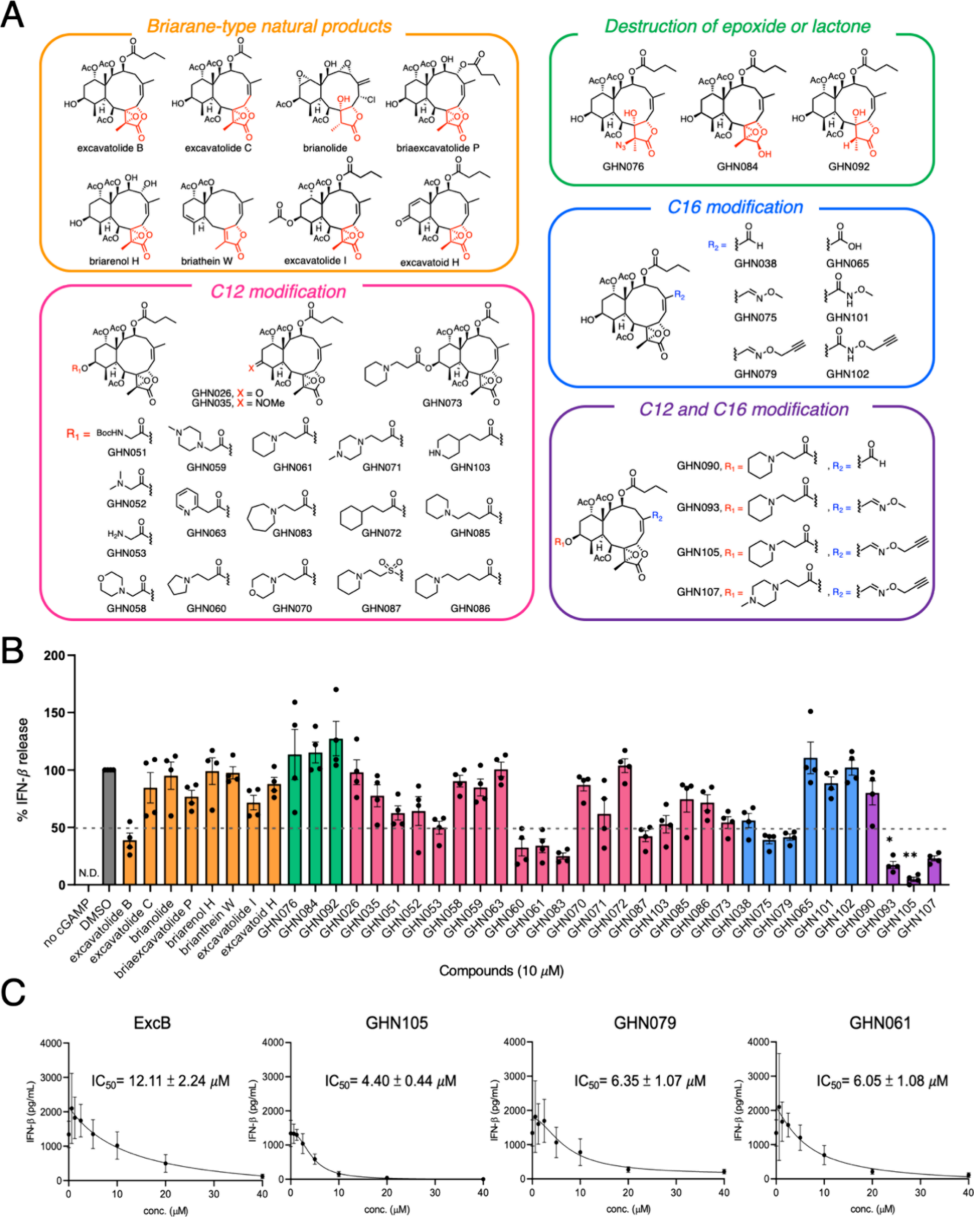
Structure–activity relationships of ExcB analogs. (A) Diversification strategies and structures of natural or synthetic briarane analogs. (B) ELISA analysis of IFN-β released from THP1 cells pretreated with vehicle control (DMSO) or the indicated compounds at 10 μM for 1 h, followed by cGAMP treatment of for 24 h. Error bars, s.e.m. *n* = 4. (C) ELISA analysis of IFN-β levels in culture supernatants of THP1 macrophages pretreated with the indicated concentrations of ExcB, **GHN079**, **GHN061**, **GHN105** prior to cGAMP treatment for 24 h. Error bars, s.e.m. *n* = 3. The calculated IC_50_ value ± s.e.m. for each indicated compound is listed.

The stability and safety under physiological conditions of epoxides as structural components have been demonstrated in several FDA-approved drugs.^22^ As the substituents at the epoxide three-membered ring allow tuning of the epoxide’s chemical reactivity toward nucleophilic groups,^23^ we envisioned that the embedded epoxy lactone group in ExcB represents a conjugation site. We first incubated ExcB with cysteine residue at 50 °C under basic conditions and isolated a single covalent adduct. Through X-ray crystal analysis (Table S1), the thiol group of cysteine cross-linked to the parent compound ExcB at electrophilic C17, an activated portion of the epoxide adjacent to an electron-withdrawing lactone moiety (Figure 1A). Based on the macrocyclic frame of ExcB, we devised several synthetic routes to obtain new ExcB analogs (GHN series, Figure 1B–D). We first synthesized two additional naturally occurring briaranes from ExcB. Esterification of the C12 hydroxyl group in the presence of acetic anhydride and triethylamine afforded excavatolide I,^24^ while pyridinium chlorochromate oxidation followed by elimination with a catalytic amount of acetic acid in toluene afforded excavatoid H (Figure 1B).^25^ As palmitoylation at Cys91 of STING drives its activation and downstream type I interferon (IFN) response, we then assessed the activities of the newly synthesized ExcB analogs in modulating the IFN response (Figure 2A,B). With 24 h incubation at 10 μM, ExcB, related briarane natural products, and new derivatives did not affect the cell viability of THP-1 human macrophages (Figure S1A). To examine the IFN responses, we then incubated these compounds at 10 μM with THP-1 cells for 1 h before the addition of human endogenous STING agonist 2′,3′-cyclic guanosine monophosphate adenosine monophosphate (cGAMP). Interestingly, when compared to ExcB, ELISA analysis showed a loss of anti-IFNβ secretion activities among these natural products, suggesting that in addition to the electrophilic epoxy lactone group, peripheral butyryloxy functional group on the C1–C5 of the macrocyclic 10-member ring also contributed to the inhibitory activities (Figure 2B). Highlighting the importance of the electrophilic warhead in ExcB for STING inhibition, perturbation of the epoxy lactone through NaBH_4_ reduction of γ-lactone to lactol (**GHN084**), selective ring-opening of 8,17-epoxide using azide (**GHN076**) or organoselenium reagent (**GHN092**) yielded analogs unable to inhibit STING-dependent IFN-β secretion in THP-1 cells (Figures 1C and 2B). These results showed that geometry, binding orientation, electronic properties of the epoxy lactone warhead, and the peripheral butyryloxy functional groups on C1–C5 of ExcB are instrumental for the covalent engagement and inhibition of STING.

### Structure–Activity Relationship Investigation and Identification of GHN105

By keeping the electrophilic epoxy lactone and peripheral butyryloxy groups on the flexible macrocyclic portion, we hypothesized that modifications at other positions around the ExcB carbo-frame could allow the discovery of potent STING antagonists. Possessing the same characteristic [8.4.0] bicyclic tetradecane core structures that are commonly associated with a γ-lactone spanning C7–C8 (Figure 2A),^26,27^ naturally occurring briaranes offered initial insights into the structure–activity relationships of ExcB analogs as STING inhibitors. Isolated from *Briareum stechei*, excavatolide C,^27^ briaexcavatolide P,^28^ and briarenol H^29^ harnessed different hydroxyl functional groups at C3 and C4 (Figure 2A). With the incorporation of heteroatoms for solubility enhancement, we also profiled both the addition and orientation of the nitrogen atom in the drug-like piperidine group, as well as the substituents of morpholino and methylpiperazine moieties (Figure 2A,B). We first focused on the esterification of C12 of ExcB. The extension with glycine (**GHN053**) exhibited only a marginal loss of activity to that of ExcB. However, a methylene extension of morpholino (**GHN058**), methylpiperazine (**GHN059**), and 2-(pyrid-2-yl) (**GHN063**) were found to be inactive, suggesting that the projected length of the functional groups and a planar aromatic ring might not provide suitable interactions. We then observed that modifications of C12 hydroxyl with 3-(pyrrolidin-1-yl) (**GHN060**), 3-(piperidin-1-yl) (**GHN061**), or 3(azepan-1-yl) (**GHN083**) propanoic acids has resulted in improved activities, suggesting an ethylene spacer might be the preferred length together with different ring sizes of cyclic hydrophilic groups were tolerated without significant modulation of the bioactivities. With **GHN061**, the importance of nitrogen atom’s position in the cyclic ring was explored. By keeping the same ethylene spacer, analogs harnessing 3-morpholino (**GHN070**), 3-(4-methylpiperazin-1-yl) (**GHN071**), cyclohexane ring in (**GHN072**), and 3-(piperidin-4-yl) (**GHN103**) showed loss of activities when compared to that of **GHN061**. In terms of the spacer length, esterifications of C12 hydroxyl with either 4-(piperidin-1-yl)butanoic acid (**GHN085**) or 5-(piperidin-1-yl)pentanoic acid (**GHN086**) less active analogs to **GHN061**. Moreover, substituting the bioisosteric sulfonate ester (**GHN087**) had weaker inhibitory activity than ester **GHN061**. Interestingly, by incorporation of the same 3-(piperidin-1-yl) functional chain onto another naturally occurring briarane Excavatolide C, similar trend of activity enhancement was observed (ExcC vs **GHN073**). For the C12 hydroxyl modifications, we have identified several ester analogs (**GHN060**, **GHN061**, and **GHN083**), bearing ethylene spacer with cyclic hydrophobic functional groups, with improved IFNβ release inhibitory activities.

Concurrently, to modify the larger macrocyclic carbon frame, activation of the C16 allylic C–H bond with an excess of SeO_2_ in 1,4-dioxane at 80 °C, smoothly afforded the enal **GHN038** as a sole product, as confirmed by X-ray crystal analysis (Table S4). With **GHN038** as an intermediate, we condensed different hydroxylamines to obtain active analogs **GHN075** and **GHN079** (Figures 1D and 2A). On the other hand, carboxylic analog **GHN065** and amide-based extensions in analogs **GHN101** and **GHN102** showed a loss of STING inhibition, providing insights into the tolerability of chemical reactions and functional groups at C16 (Figure 1D). Notably, combining advantageous C12 and C16 modifications yielded **GHN105** (IC_50_ of 4.4 μM), which exerted the strongest inhibition of STING-dependent IFN-β secretion in THP1 macrophages in a dose-dependent manner among the analogs screened (Figures 2C and S1B). Taken together, we have identified **GHN105**, with at least a 20-fold improvement of solubility in aqueous media over ExcB (Figure S2), to exert potent anti-IFNβ secretion activity in THP-1 human macrophages.

### GHN105 Covalently Engaged with STING at Cys91

**Figure 3:**
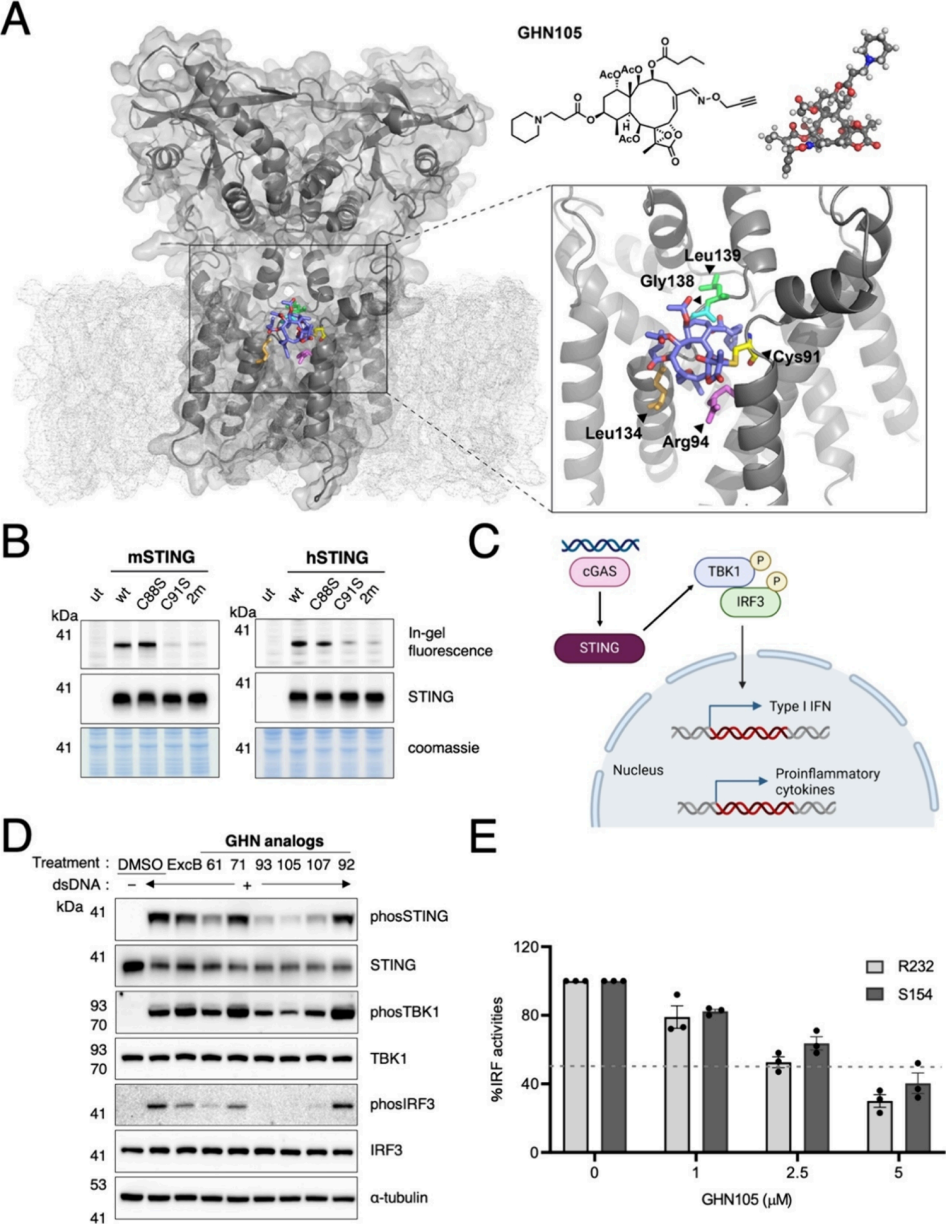
Covalent engagement of hSTING by GHN105 at Cys91. (A) Covalent docking of GHN105 (blue) at Cys91 of apo-hSTING dimer (6NT5). An X-ray diagram of GHN105 was shown. The membrane bilayer environment was simulated to exhibit the embedding position of the hSTING-Cys91-GHN105 complex. (B) HEK293T cells expressing wild type (wt) mSTING or hSTING and the indicated cysteine-to-serine mutants were labeled with 2 μM GHN105 for 15 min ut, untransfected; 2m, C88S/C91S double mutant. GHN105 engagement of mSTING or hSTING was monitored by in-gel fluorescence. Anti-STING Western blot and Coomassie blue stains serve as loading controls for the accompanying fluorescence gels. (C) Scheme created with BioRender.com showing the cGAS-STING signaling cascade in response to intracellular nucleic acids. (D) Western blot analyses of phosphorylated and total STING, TBK1, and IRF3 in NIH3T3 cells pretreated with DMSO or 2 μM of the indicated analogs for 1 h, followed by dsDNA induction of STING signaling for 2 h. Antitubulin blots served as loading controls for the accompanying blots. (E) THP1-Dual KI-hSTING monocytes were pretreated with GNH105 for 1 h prior to cGAMP addition for 24 h. Luciferase reporter activity was normalized to DMSO-treated controls (100%), and the dotted line showed 50% of activity. Error bars, s.e.m. *n* = 3.

Similar to ExcB,^16^ in silico docking studies predicted **GHN105** to be associated with the external membrane-proximal binding pocket of the full-length apo-hSTING dimer, where Cys91 is available for nucleophilic attack (Figure 3A). The docking analysis of **GHN105** revealed that the ethylene spacer positioned the piperidine group within the STING dimer interface, allowing it to engage with a hydrophobic pocket created by the side chains of Leu136 and Leu139 (B-chain) and Leu58 (A-chain). Additionally, the suitable spacer fostered a polar interaction between the nitrogen atom of the piperidine and the backbone carbonyl of Gly138 (B-chain), consistent with the structure–activity relationship of the C12 hydroxyl modification (Figure 3A). Concurrently, the alkynyl hydroxylamine moiety projected from C16 interacted with the backbone of Leu134 (B-chain), and the guanidine group of Arg94 (A-chain) formed a hydrogen bonding with **GHN105**’s lactone to facilitate the positioning of C17 close to STING-Cys91, which may account for the improved activity of **GHN105** compared to ExcB. Taking advantage of the alkynyl handle within **GHN105,** we confirmed that **GHN105** primarily engaged wild-type mSTING and hSTING (His232) at Cys91 in HEK293T cells by in-gel fluorescence after copper-mediated azide–alkyne cycloaddition (CuAAC) of azide-Cy5. mSTING- and hSTING-associated fluorescence was markedly reduced when Cys91, but not the neighboring Cys88, was mutated to serine (Figure 3B). These results indicated that **GHN105** site-selectively engaged both mSTING and hSTING at the conserved Cys91, which has been shown to be an S-palmitoylation site critical for STING-mediated signaling.^30^

### GHN105 Inhibited STING Signaling and Activation in Cells

We then profiled the abilities of active briarane analogs to modulate the STING-associated signaling pathway (Figure 3C). Following cGAMP-driven conformational changes and trafficking through the ER–Golgi intermediate compartment,^31^ STING is activated through palmitoylation at the Golgi.^30^ The activated STING then recruits TANK-binding kinase 1 (TBK1) and IκB kinase (IKK), which phosphorylate interferon regulatory factor 3 (IRF3) and the nuclear factor-κB (NFκB) inhibitor IκBα, respectively.^32^ We tested the activity of **GHN105** in multiple noncancer cell lines against mouse STING (mSTING) and different human STING (hSTING) variants. In murine NIH3T3 cells, **GHN105** strongly reduced dsDNA-induced phosphorylation of STING, TANK-binding kinase 1 (TBK1), and IFN regulatory factor 3 (IRF3) (Figure 3D), which are key signaling effectors required for STING-dependent IFN response.^30−33^ Consistent with its STING inhibitory activities,^34^**GHN105** suppressed host antiviral responses in human mesenchymal stem cells (Figure S3). Notably, **GHN105** readily reduced IFN response in wild type (Arg232) and gain-of-function (Ser154) hSTING knock-in THP1 reporter cells, supporting its efficacy against the most common hSTING allele as well as the pathogenic variant causing SAVI (Figure 3E).

### Identification of GHN105 as an Orally Bioavailable STING Inhibitor

**Figure 4:**
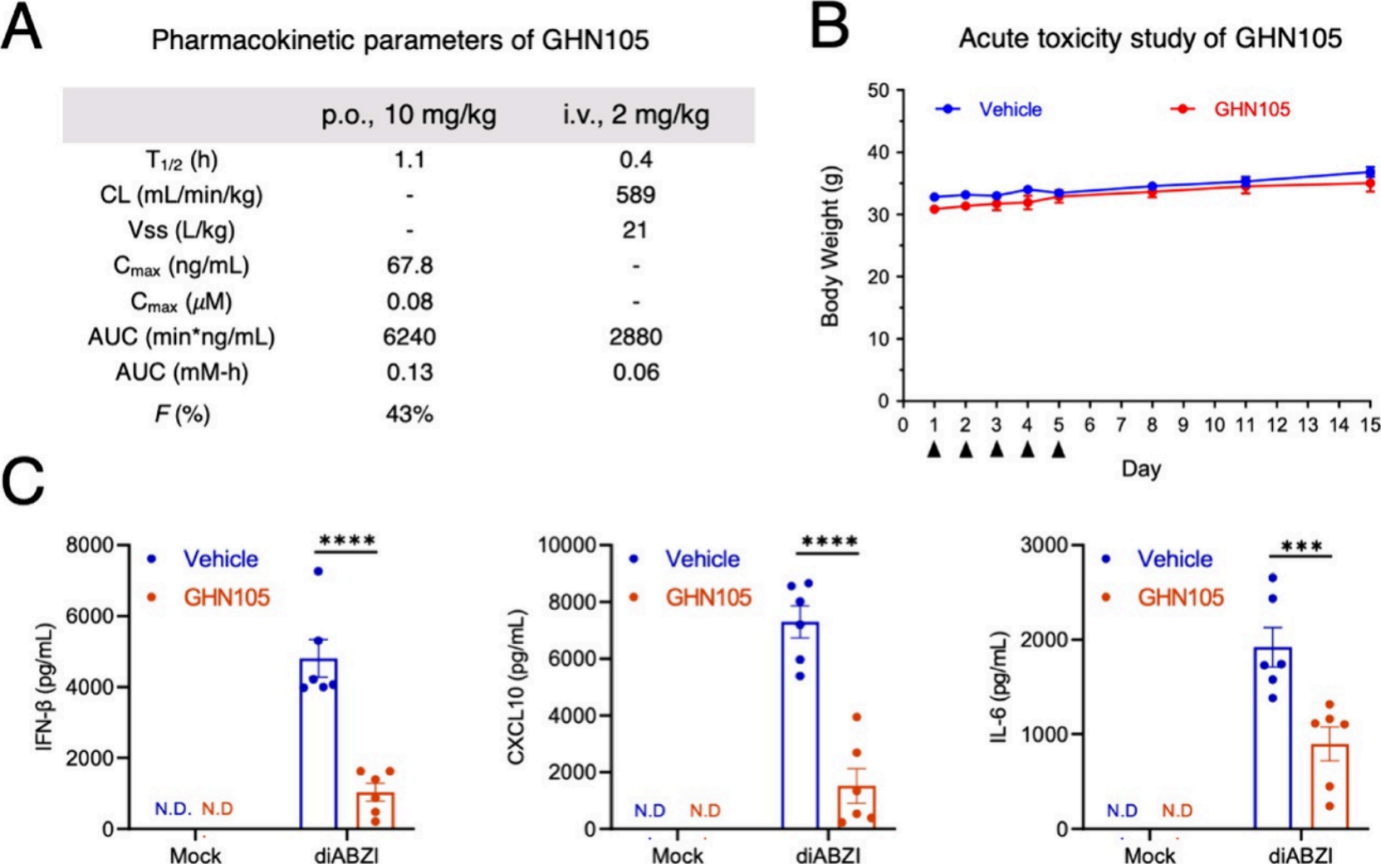
Orally administered GHN105 suppressed STING-dependent increase in serum levels of IFN-β and proinflammatory cytokines. (A) In vivo PK properties of GHN105 with 2 mg/kg intravenous injection (i.v.) and 10 mg/kg oral gavage (p.o.) in male ICR mice (*n* = 3). *T*_1/2_, time estimate of amount of drug to be reduced by one-half in animal; *C*_max_, peak serum concentration achieved by compound; CL, clearance; *V*_ss_, apparent volume of distribution at steady state; AUC, area of drug concentration under curve. (B) Acute toxicity assessment in ICR mice with 5 consecutive days of GHN105 (100 mg/kg, p.o.) or vehicle administration. All mice survived and body weights over 14 days after 1st dosing were presented. Error bars, s.e.m. *n* = 3. (C) C57BL/6J mice were pretreated with GHN105 (100 mg/kg, p.o) or vehicle for 1 h, followed by diABZI (2 mg/kg, i.p.) for 3.5 h. Serum levels of IFN-β, IL-6, and CXCL10 after 3.5 h were measured using ELISA. Error bars, s.e.m. *n* = 6. N.D., not detectable. Two-way ANOVA, ***p < 0.001, ****p < 0.0001.

To investigate the in vivo efficacy of **GHN105**, we first carried out pharmacokinetic (PK) and toxicity studies. To evaluate **GHN105**’s bioavailability, we evaluated the in vivo pharmacokinetic properties of **GHN105** with dosages of 2 mg/kg of intravenous injection (i.v.), 10 mg/kg of oral gavage (p.o.), and 15 mg/kg of intraperitoneal injection (i.p.) (Figures 4A and S4A). Notably, oral administration of **GHN105** possessed satisfactory PK parameters with a half-life of 1.1 h and oral bioavailability of 43% (Figure 4A). To examine **GHN105**’s in vivo efficacy, we first pretreated mice for 1 h with **GHN105** (25 or 50 mg/kg, i.p.) prior to treatment of STING agonist amidobenzimidazole (diAZBI, 2 mg/kg, i.p.) for 3.5 h. The dose-dependent reduction in serum IFN-β levels in **GHN105**-treated mice demonstrated the in vivo efficacy of **GHN105** as a STING inhibitor (Figure S4B). Despite the i.p. route resulting in nearly a 10-fold increase in the *C*_max_ of **GHN105** compared to the oral route, the advantages of the oral administration, such as its noninvasive nature, enhanced patient compliance for chronic inflammatory diseases related to the STING pathway, cost-effectiveness in drug development, together with an observed oral bioavailability of 43%, have encouraged us to investigate oral delivery of **GHN105** for in vivo efficacy assessment. Based on the *C*_max_ value from the PK studies indicating that sufficient inhibitory concentrations could be achieved with a 100 mg/kg oral dose, we, therefore, performed toxicity studies in mice with of 100 mg/kg per day for 5 days. The results showed no apparent adverse effects or body weight loss in this dosing regimen (Figure 4B). As an additional safety index of **GHN105**, no apparent developmental defects were observed in a zebrafish embryo acute toxicity assay (Figure S5). Indeed, consistent with the PK data, mice pretreated with 100 mg/kg of **GHN105** through the oral route before diABZI introduction exhibited significantly reduced serum levels of IFN-β, IL-6, and CXCL10 (Figure 4C). Together, these results demonstrated that **GHN105** is a safe and orally bioavailable STING inhibitor.

### GHN105 Engaged STING In Situ and Suppressed Acute Colitis Progression

**Figure 5:**
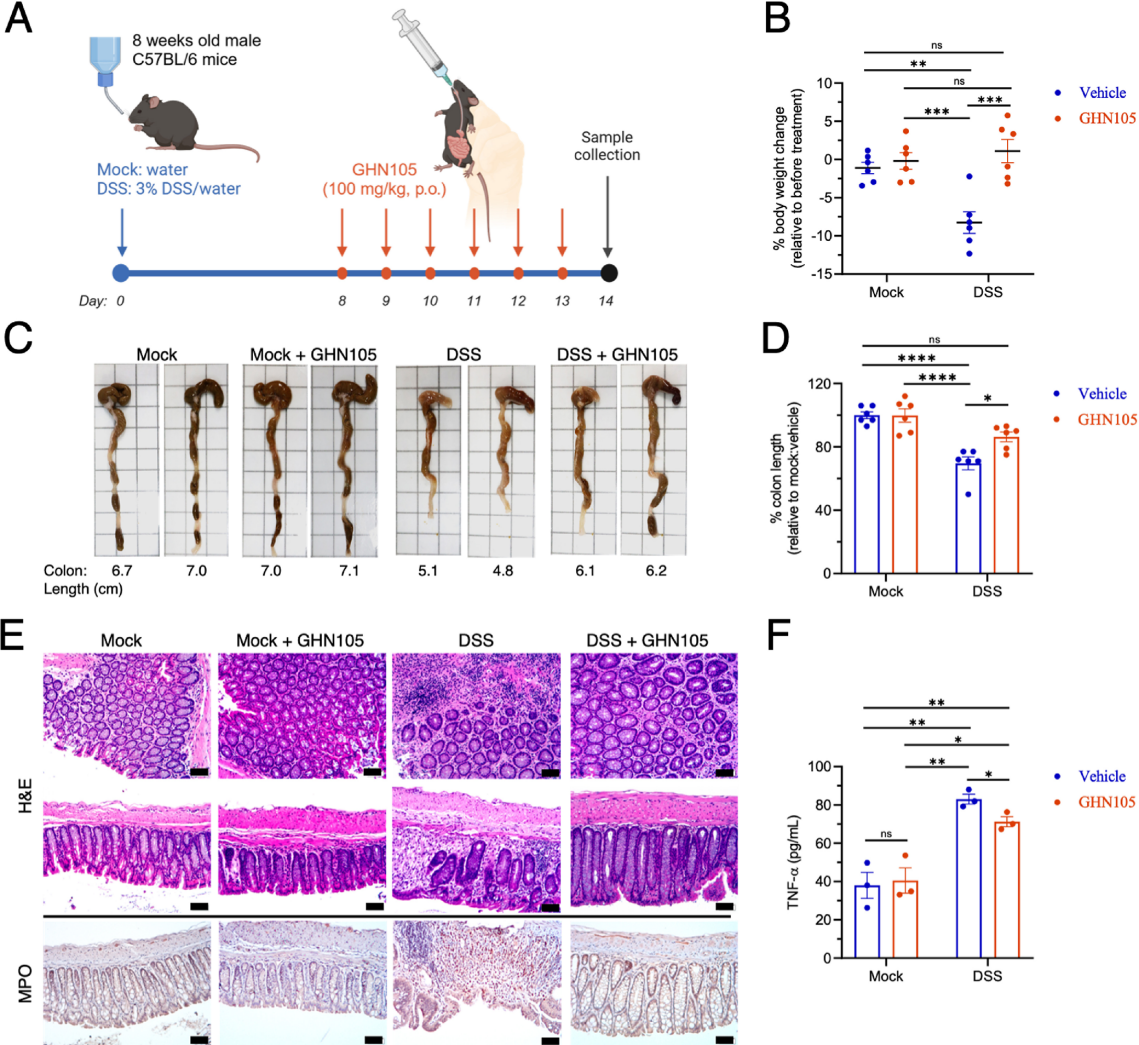
Orally administrated GHN105 reversed hallmark pathological features in a delayed treatment acute colitis mouse model. (A) DSS-induced acute colitis murine model and a delayed-treatment regimen for GHN105. For mock treatments, no DSS was included in the drinking water. Scheme was created with BioRender.com. (B) Percent change in body weight change (day 14 compared to day 8). (C) Representative images of colons from the indicated treatment groups. Measured colon lengths are indicated. (D) Colon lengths of the indicated groups: healthy controls (mock), mock treated with GHN105 (mock+GHN105), DSS-only control (DSS), and DSS followed by GHN105 cotreatment (DSS+GHN105). Error bars, s.e.m. (*n* = 6, 3 mice for each group over two independent biological repeats. Two-way ANOVA, *p < 0.05; **p < 0.01; ***p < 0.001; ****p < 0.0001; ns, not significant. (E) Representative images of hematoxylin/eosin (H&E) and myeloperoxidase (MPO) stains of colon sections from mice of indicated treatment groups. Scale bars, 50 μm. (F) Plasma TNF-α levels of mice from indicated treatment groups. Error bars, s.e.m. (*n* = 3). Two-tailed *t* test, *p < 0.05; **p < 0.01.

**Figure 6:**
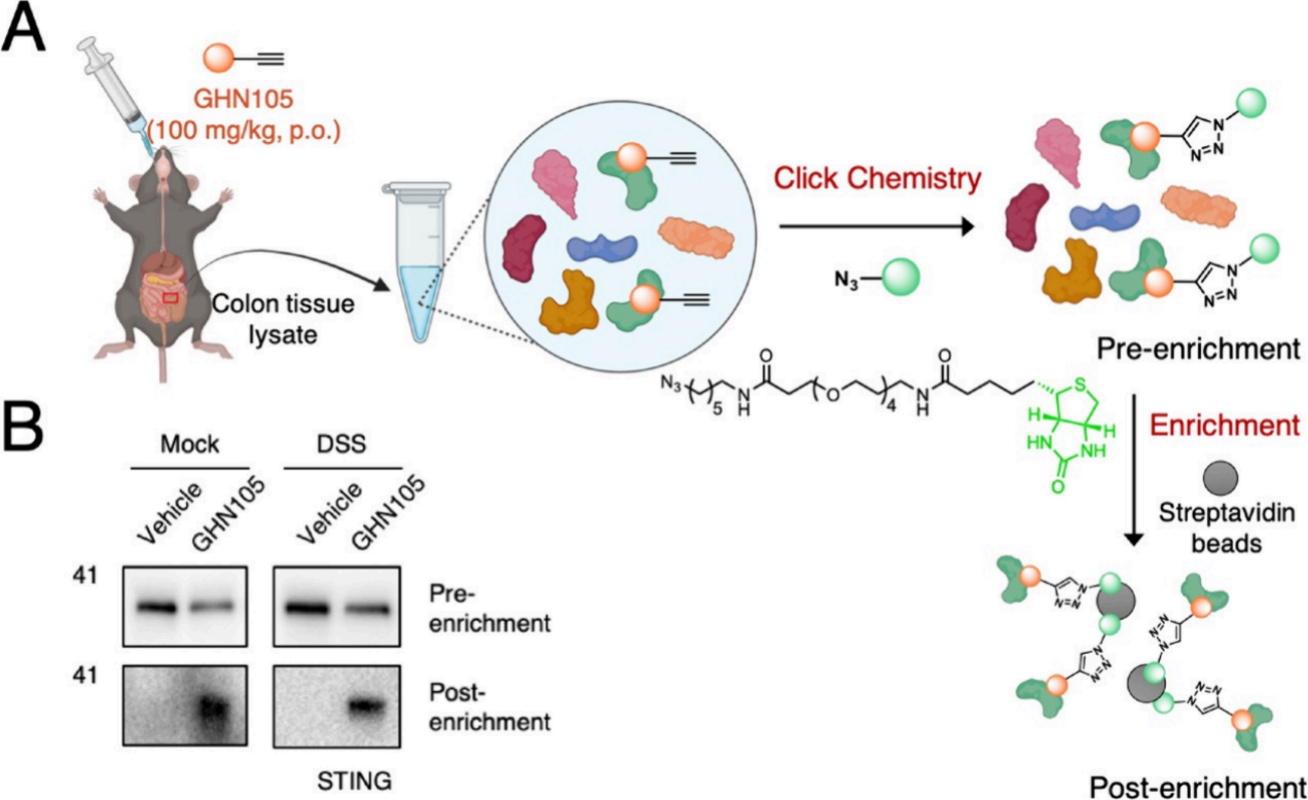
Covalent engagement of mSTING in **GHN105**-treated animals. (A) Colon protein samples from the indicated treatment groups were reacted with azide-biotin. Scheme was created with BioRender.com. (B) Protein samples before and after streptavidin enrichment were immunoblotted for STING, providing in vivo on-target validation in the acute colitis model.

We then investigated the therapeutic effects of **GHN105** in a dextran sodium sulfate (DSS)-induced colitis mouse model, which has been shown to be dependent on STING signaling.^7,35^ With a delayed treatment regimen (Figure 5A), mice were first fed 3% DSS for 8 days, during which significant body weight loss (Figure 5B) and hallmark reduction of colon lengths were readily observed (Figure 5C,D). This was followed by a treatment phase of six daily oral administrations of 100 mg/kg **GHN105** or vehicle control. Strikingly, despite continued DSS exposure during the treatment phase, we observed significant recovery of body weights and colon lengths with **GHN105** treatment (Figure 5C,D). Histological staining showed extensive neutrophil infiltration by myeloperoxidase staining, effacement of epithelial architecture, and swelling in colon sections of mice receiving vehicle treatment, but these pathological features were markedly alleviated in **GHN105**-treated mice (Figure 5E). Concurrently, elevated plasma levels of TNF-α induced by DSS exposure were significantly suppressed with **GHN105** treatment (Figure 5F). We demonstrated that GHN105 is the first orally bioavailable covalent STING inhibitor to exert significant therapeutic effects in a DSS-induced acute colitis model when administered following a delayed treatment regimen. Furthermore, to verify that **GHN105** could interact with STING in vivo in the colon, we took advantage of the clickable alkynyl handle within **GHN105**. Protein samples were prepared from mock and DSS-treated colon sections and reacted with azide-biotin via CuAAC condition (Figure 6A). After affinity purification, Western blot analysis showed the in vivo on-target engagement of STING by **GHN105** in the colons of mice receiving oral **GHN105** treatment (Figure 6B).

## Conclusions

Here, we detailed the discovery and validation of **GHN105** as the first oral bioavailable covalent STING inhibitor. Utilizing the electrophilic epoxy lactone moiety, we achieved late-stage diversifications on the marine diterpenoid briarane-type structure, enhancing its STING inhibitory activity and solubility compared to the parent natural product ExcB. As a new warhead for covalent interaction with STING, in-gel fluorescence demonstrated that **GHN105** binds to mSTING or hSTING through Cys91, a conserved STING activation post-translational modification site. Notably, **GHN105** inhibited TBK1 and IRF3 phosphorylation and maintained strong activity against different hSTING variants. Our study revealed another example of a macrocyclic natural product that defies the “rule of five” for oral bioavailability. Importantly, we confirmed the therapeutic efficacy of **GHN105** in a preclinical acute colitis model, and demonstrated biochemically that orally administered **GHN105** could reach and covalently engage STING in vivo in the colon. With growing evidence on cGAS-STING’s role in sterile inflammations which forms the basis of many human diseases, our discovery identifies an orally active covalent chemotype targeting STING and supports a new treatment strategy for chronic inflammatory diseases linked to the overactive cGAS-STING pathway.

## Experimental Section

### Chemistry

Reactions were carried out under ambient atmosphere unless otherwise specified. Anhydrous dimethylformaldehyde (DMF) was purchased form Acros. Dry dichloromethane (CH_2_Cl_2_) was obtained by passing through activated aluminum column. All commercially obtained reagents were used as received unless otherwise specified. Yields refer to purified and spectroscopically pure compounds. All reactions were monitored by thin-layer chromatography (TLC) or Agilent 1290 infinity II UPLC-MS. Melting points were measured in one end open capillary tubes on a Kruss melting point apparatus. TLC was performed using Merck silica gel 60 F_254_ precoated glass plates and visualized by UV fluorescence quenching (254 nm) or KMnO_4_ staining. Fuji silysia (CHROMATOREX-MB70, 40–75 μm) silica gel was used for flash column chromatography. Eluent systems are given in volume/volume concentrations. NMR spectra were recorded on a Bruker Ascend 400 spectrometer operating at 400 MHz for ^1^H and 100 MHz for ^13^C, Bruker Ascend 600 operating at 600 MHz for ^1^H and 151 MHz for ^13^C acquisitions, respectively. The following solvent chemical shifts were used as reference values (ppm): CDCl_3_ = 7.26 (^1^H), 77.00 (^13^C); (CD_3_)_2_CO = 2.05 (^1^H), 29.80 (^13^C). Data for ^1^H NMR spectra are reported as follows: chemical shift (δ ppm), multiplicity, coupling constant (Hz) and integration. For most of the synthetic analogs, both ^1^H and ^13^C NMR were measured at −40 °C in (CD_3_)_2_CO due to the interconverting conformations. High-resolution mass spectra (HRMS) were obtained on JEOL JMS-700 (FAB) or Waters LCT (ESI) at the Academia Sinica. All X-ray diffraction data were collected and analyzed by National Taiwan Normal University Instrument Center. The purities of the target compounds were found to be ≥95% determined by ^1^H NMR and HPLC or UPLC. HPLC performed on a Agilent 1100 series or Waters alliance e2695 with a UV detector at 210 or 254 nm and C18 column (Hypersil GOLD C18 selectivity HPLC column, 250 × 4.6 mm, 5 μm, Thermo Scientific), eluting with a gradient of acetonitrile/water (40–100%) containing 0.1% trifluoroacetic acid with a flow rate of 1 mL/min. UPLC performed on a Waters Acquity UPLC/BSM with photodiode array detector at 254 nm and C18 column (Waters Acquity BEH-C18, 50 × 2.1 mm, 1.7 μm), eluting with a gradient of acetonitrile/water (10–90%) containing 2 mM ammonium acetate and 0.1% formic acid with a flow rate of 0.6 mL/min.

### Synthesis and Characterizations of Briarane Natural Products and Key Derivatives

#### Synthesis of **Excavatolide I**

Under ambient atmosphere, a 4 mL vial equipped with a stir bar was charged with **ExcB** (11.1 mg, 0.0187 mmol, 1.0 equiv), dried Et_3_N (36.3 mg, 0.356 mmol, 19.0 equiv), DMAP (a catalytic amount) dried CH_2_Cl_2_ (0.5 mL) and acetic anhydride (2 drops, excess). The vial was sealed with a Teflon cap, and the resulting solution was stirred at room temperature. After 3 h, the reaction mixture was diluted with CH_2_Cl_2_ and the organic layer was washed with H_2_O. The organic layer was dried over MgSO_4_, filtered, and concentrated by rotary evaporation. The residue was purified by flash column chromatography (EtOAc:*n*-Hexane = 0:1 → 2:3 → 1:1) to afford **excavatolide I** (10.3 mg, 87% yield) as a yellowish oil. **TLC**: *R*_f_ = 0.50 (EtOAc: *n*-Hexane = 1:1, KMnO_4_). ^1^**H NMR** (600 MHz, (CD_3_)_2_CO, −40 °C) δ 5.84 (dd, *J* = 7.1, 1.7 Hz, 1H), 5.67 (d, *J* = 7.3 Hz, 1H), 5.52 (d, *J* = 10.4 Hz, 1H), 5.36 (d, *J* = 7.4 Hz, 1H), 5.16 (d, *J* = 2.4 Hz, 1H), 5.00–4.91 (m, 1H), 4.69 (dd, *J* = 4.0, 2.1 Hz, 1H), 4.01 (dd, *J* = 15.9, 7.3 Hz, 1H), 3.15 (dd, *J* = 10.4, 5.1 Hz, 1H), 2.66–2.58 (m, 1H), 2.40 (s, 3H), 2.23 (s, 3H), 2.21 (s, 3H), 2.20–2.06 (m, 4H), 1.98 (s, 3H), 1.95 (s, 3H), 1.88–1.82 (m, 1H), 1.55–1.45 (m, 2H), 1.50 (s, 3H), 1.08 (d, *J* = 7.1 Hz, 3H), 0.86 (s, 3H), 0.85 (t, *J* = 7.4 H, 3H). ^**13**^**C NMR** (151 MHz, (CD_3_)_2_CO, −40 °C) δ 172.3, 172.1, 171.9, 170.7, 170.3, 170.1, 140.0, 122.6, 81.5, 81.4, 74.2, 73.6, 70.1, 69.2, 65.2, 60.6, 44.2, 40.3, 35.9, 34.3, 33.0, 27.1, 22.6, 22.1, 21.4, 21.0, 18.4, 18.0, 13.7, 10.2, 10.0. **HRMS-ESI** (*m*/*z*) calcd for C_32_H_44_O_13_Na [M + Na]^+^ 659.2674, found 659.2675. **HPLC purity**: *t*_R_ = 18.3 min, 100.0% (λ = 210 nm).

#### Synthesis of **Excavatoid H**

Under ambient atmosphere, a 4 mL vial equipped with a stir bar was charged with **GHN026** (22.6 mg, 0.0371 mmol, 1.00 equiv), acetic acid (3 drops) and toluene (0.4 mL). The vial was sealed with a Teflon cap, and the reaction mixture was stirred at reflux temperature. After 18 h, the reaction mixture was diluted with EtOAc and the organic layer was washed with H_2_O. The organic layer was dried over MgSO_4_, filtered, and concentrated by rotary evaporation. The residue was purified by flash column chromatography (EtOAc:*n*-Hexane = 0:1 → 1:3 → 2:3) to afford **excavatoid H** (17.0 mg, 86% yield) as a colorless solid. **TLC**: *R*_f_ = 0.20 (EtOAc: *n*-Hexane = 1:1, KMnO_4_). ^**1**^**H NMR** (600 MHz, CDCl_3_, 22 °C) δ 6.56 (d, *J* = 10.4 Hz, 1H), 6.10 (dd, *J* = 10.4, 0.8 Hz, 1H), 5.38 (d, *J* = 7.4 Hz, 1H), 5.36–5.33 (m, 2H), 5.20 (d, *J* = 10.0 Hz, 1H), 4.83 (dd, *J* = 3.1, 1.5 Hz, 1H), 3.47 (dd, *J* = 15.6, 4.9 Hz, 1H), 3.30 (dd, *J* = 10.0, 4.6 Hz, 1H), 2.86 (qd, *J* = 7.5, 4.7 Hz, 1H), 2.31 (s, 3H), 2.26 (s, 3H), 2.26–2.23 (m, 2H), 2.07 (d, *J* = 16.1 Hz, 1H), 1.73 (s, 3H), 1.63 (sext, *J* = 7.6 Hz, 2H), 1.58 (s, 3H), 1.21 (d, *J* = 7.4 Hz, 3H), 1.05 (s, 3H), 0.95 (t, *J* = 7.4 Hz, 3H). ^**13**^**C NMR** (151 MHz, CDCl_3_, 22 °C) δ 201.0, 172.6, 171.2, 170.4, 169.0, 155.2, 139.4, 126.3, 123.0, 81.8, 73.7, 71.8, 69.2, 66.9, 60.0, 43.1, 41.4, 38.7, 36.1, 33.5, 21.9, 21.8, 21.5, 18.1, 17.1, 14.4, 13.6, 10.2. **HRMS-FAB** (*m*/*z*) calcd for C_28_H_37_O_10_ [M + H]^+^ 533.2387, found 533.2383. **HPLC purity**: *t*_R_ = 17.0 min, 95.8% (λ = 210 nm).

#### Synthesis of **GHN076**

Under ambient atmosphere, a 4 mL vial equipped with a stir bar was charged with **ExcB** (20.1 mg, 0.0338 mmol, 1.00 equiv), NaN_3_ (8.8 mg, 0.135 mmol, 4.00 equiv) and DMF (0.3 mL). The vial was sealed with a Teflon cap, and the reaction mixture was stirred at 80 °C. After 16 h, the reaction mixture was cooled to rt and quenched with H_2_O. The organic layer was separated and the aqueous layer was extracted with EtOAc. The combined organic layers were dried over MgSO_4_, filtered, and concentrated by rotary evaporation. The residue was purified by flash column chromatography (EtOAc:*n*-Hexane = 0:1 → 1:1) to afford **GHN076** (15.5 mg, 72% yield) as a colorless solid. **TLC**: *R*_f_ = 0.33 (EtOAc: *n*-Hexane = 1:1, KMnO_4_). ^**1**^**H NMR** (600 MHz, (CD_3_)_2_CO, −40 °C) δ 5.83 (d, *J* = 2.9 Hz, 1H), 5.80–5.74 (m, 1H), 5.65 (d, *J* = 5.4 Hz, 1H), 5.63–5.56 (m, 1H), 5.49 (d, *J* = 9.7 Hz, 1H), 5.46–5.40 (m, 1H), 5.19 (d, *J* = 6.4 Hz, 1H), 5.05 (br s, 1H), 4.78 (d, *J* = 3.3 Hz, 1H), 4.53 (t, *J* = 2.9 Hz, 1H), 4.46 (d, *J* = 4.2 Hz, 1H), 4.03 (dd, *J* = 16.1, 5.3 Hz, 1H), 3.99 (d, *J* = 4.3 Hz, 1H), 3.94–3.86 (m, 1H), 3.77 (d, *J* = 4.3 Hz, 1H), 3.51 (dd, *J* = 15.5, 3.9 Hz, 1H), 2.81–2.72 (m, 1H), 2.69 (dd, *J* = 9.5, 4.3 Hz, 1H), 2.65 (d, *J* = 5.2 Hz, 1H), 2.40 (s, 3H), 2.32–2.23 (m, 2H), 2.18 (s, 3H), 2.16 (s, 3H), 2.17–2.14 (m, 1H), 2.12 (s, 3H), 2.11 (s, 3H), 2.00 (apparent d, *J* = 15.7 Hz, 1H), 1.94 (s, 3H), 1.92 (apparent d, *J* = 13.4 Hz, 1H), 1.85 (s, 3H), 1.80 (s, 3H), 1.78 (s, 3H), 1.79–1.70 (m, 1H), 1.54–1.59 (m, 2H), 1.49 (s, 3H), 1.48 (s, 3H), 1.51–1.45 (m, 2H), 1.17 (d, *J* = 7.1 Hz, 3H), 0.99 (d, *J* = 6.9 Hz, 3H), 0.89 (t, *J* = 7.4 Hz, 3H), 0.84 (t, *J* = 7.4 Hz, 3H), 0.80 (s, 3H). ^**13**^**C NMR** (151 MHz, (CD_3_)_2_CO, −40 °C) δ 173.3, 172.6, 172.1, 172.0, 171.8, 170.9, 170.3, 170.1, 170.0, 143.3, 141.0, 122.2, 121.6, 84.5, 82.9, 82.7, 81.9, 81.0, 79.5, 75.7, 74.6, 74.0, 73.7, 72.4, 69.7, 69.6, 68.8, 67.9, 65.8, 45.4, 44.1, 43.2, 39.7, 38.3, 36.2, 36.0, 35.8, 35.7, 33.3, 32.8, 23.5, 23.5, 22.4, 22.2, 21.8, 21.3, 21.0, 20.9, 18.5, 18.3, 18.2, 17.2, 16.5, 14.4, 13.7, 13.6, 12.3, 11.8, 9.8. *Note: Two major conformers were observed at 233 K in (CD*_*3*_*)*_*2*_*CO in a 75/25 ratio.***HRMS-FAB** (*m*/*z*) calcd for C_30_H_44_N_3_O_12_ [M + H]^+^ 638.2925, found 638.2921. **HPLC purity**: *t*_R_ = 16.6 min, 100.0% (λ = 210 nm). Crystals suitable for X-ray crystallography were grown using layer diffusion of *n*-hexane into a solution of **GHN076** in CH_2_Cl_2_.

#### Synthesis of **GHN084**

Under an ambient atmosphere, a 4 mL vial equipped with a stir bar was charged with **ExcB** (16.4 mg, 0.028 mmol, 1.00 equiv), MeOH (0.3 mL) was added NaBH_4_ (1.6 mg, 0.041 mmol, 1.5 equiv) at 0 °C. The vial was sealed with a Teflon cap, and the reaction mixture was stirred at 0 °C. After 3 h, the reaction mixture was concentrated by rotary evaporation. The residue was purified by flash column chromatography (MeOH:CH_2_Cl_2_ = 1:10) to afford **GHN084** (16.7 mg, quantitative yield) as a colorless solid. **TLC**: *R*_f_ = 0.13 (EtOAc: *n*-Hexane = 1:1, KMnO_4_). ^**1**^**H NMR** (600 MHz, (CD_3_)_2_CO, −40 °C) δ 6.15 (d, *J* = 4.3 Hz, 1H, 19-OH), 5.82 (dd, *J* = 8.0, 2.7 Hz, 1H), 5.42 (d, *J* = 10.8 Hz, 1H), 5.29 (d, *J* = 7.4 Hz, 1H), 5.16 (d, *J* = 4.1 Hz, 1H), 5.14–5.08 (m, 2H), 4.70–4.63 (m, 1H), 4.15 (d, *J* = 3.8 Hz, 1H, 12-OH), 4.04 (dd, *J* = 15.5, 8.1 Hz, 1H), 3.91–3.84 (m, 1H), 3.22 (dd, *J* = 10.9, 5.2 Hz, 1H), 2.48–2.41 (m, 1H), 2.36 (s, 3H), 2.19 (s, 3H), 2.17 (s, 3H), 2.17–2.05 (m, 2H), 1.99–1.92 (m, 2H), 1.90 (s, 3H), 1.71–1.64 (m, 1H), 1.56–1.44 (m, 2H), 1.40 (s, 3H), 0.99 (d, *J* = 7.1 Hz, 3H), 0.84 (t, *J* = 7.4 Hz, 3H), 0.80 (s, 3H). ^**13**^**C NMR** (151 MHz, (CD_3_)_2_CO, −40 °C) δ 171.9, 170.3, 170.1, 137.7, 126.1, 96.6, 81.8, 81.4, 73.7, 71.2, 67.5, 66.8, 66.1, 65.7, 43.8, 39.3, 36.0, 35.2, 34.6, 30.5, 22.5, 22.3, 22.2, 21.3, 18.4, 18.1, 13.7, 11.5, 9.0. **HRMS-ESI** (*m*/*z*) calcd for C_30_H_44_O_12_Na [M + Na]^+^ 619.2725, found 619.2734. **HPLC purity**: *t*_R_ = 10.0 min, 100.0% (λ = 210 nm). Crystals suitable for X-ray crystallography were grown using layer diffusion of *n*-hexane into a solution of **GHN084** in CH_2_Cl_2_.

#### Synthesis of **GHN092**

To a stirring solution of diphenyl diselenide (16.7 mg, 0.054 mmol, 1.5 equiv) in EtOH (0.6 mL) under a nitrogen atmosphere was added NaBH_4_ (4.1 mg, 0.107 mmol, 3.0 equiv) at room temperature. After evolution of hydrogen ceased, the light-yellow solution was cooled to 0 °C, followed by the addition of acetic acid (3.1 μL, 0.054 mmol, 1.5 equiv), and the mixture was further stirred at 0 °C for 50 min. The resulting colorless solution was then added to a solution of **ExcB** (21.2 mg, 0.036 mmol, 1.00 equiv) in EtOH (0.6 mL) at room temperature. The reaction mixture was diluted with EtOAc, and oxygen was passed through the solution for 5 min. The reaction mixture was washed with H_2_O. The organic layer dried over MgSO_4_, filtered, and concentrated by rotary evaporation. The residue was purified by flash column chromatography (EtOAc:*n*-Hexane = 0:1 → 2:3 → 1:1) to afford **GHN092** (20.8 mg, 98% yield) as a colorless solid of a 91:9 mixture of an inseparable diastereomers. **TLC**: *R*_f_ = 0.25 (EtOAc: *n*-Hexane = 1:1, KMnO_4_). ^**1**^**H NMR** (400 MHz, CDCl_3_, 24.5 °C) δ 5.84 (br s, 1H), 5.55 (d, *J* = 9.6 Hz, 1H), 5.51 (d, *J* = 9.6 Hz, 1H), 5.40 (s, 1H), 5.29 (br t, *J* = 6.4 Hz, 1H), 4.87 (br s, 1H), 3.89 (br s, 1H), 3.35 (br d, *J* = 16.0 Hz, 1H), 2.76 (q, *J* = 7.6 Hz, 1H), 2.60 (d, *J* = 5.2 Hz, 1H), 2.24 (t, *J* = 7.2 Hz, 2H), 2.21 (s, 3H), 2.17 (m, 1H), 2.11 (s, 3H), 2.04 (dd, *J* = 16.0, 3.2 Hz, 1H), 1.97 (s, 3H), 1.93–1.89 (m, 2H), 1.84 (s, 3H), 1.63 (s, 3H), 1.63 (sext, *J* = 7.2 Hz, 2H), 1.18 (d, *J* = 7.6 Hz, 3H), 1.10 (d, *J* = 7.2 Hz, 3H), 0.96 (t, *J* = 7.6 Hz, 3H). ^**13**^**C NMR** (100 MHz, CDCl_3_, 24.5 °C) δ 176.7, 173.2, 170.7, 170.1, 169.4, 142.4, 121.7, 85.0, 79.6, 74.3, 73.6, 73.4, 72.1, 69.9, 43.6, 42.5, 41.9, 35.9, 35.1, 35.1, 32.9, 23.8, 21.3, 21.0, 18.5, 18.0, 16.5, 13.6, 8.1. **HRMS-ESI** (*m*/*z*) calcd for C_30_H_44_NO_12_Na [M + Na]^+^ 619.2725, found 619.2735. **HPLC purity**: *t*_R_ = 13.4 min, 91.2%, *t*_R_ = 13.9 min, 8.8% (λ = 210 nm).

The major diastereomer of **GHN092** was verified by 2D-NOESY.

#### Synthesis of **GHN061**

Under an ambient atmosphere, a 4 mL vial equipped with a stir bar was charged with **ExcB** (266.5 mg, 0.448 mmol, 1.0 equiv), 3-(piperidin-1-yl)propanoic acid (140.9 mg, 0.896 mmol, 2.0 equiv), DMAP (16.4 mg, 0.134 mmol, 0.3 equiv), EDCI (257.7 mg, 1.344 mmol, 3.0 equiv) and dried CH_2_Cl_2_ (5.0 mL). The vial was sealed with a Teflon cap, and the resulting solution was stirred at room temperature. After 18 h, the reaction mixture was diluted with EtOAc and the organic layer was washed with H_2_O. The organic layer was dried over MgSO_4_, filtered, and concentrated by rotary evaporation. The residue was purified by flash column chromatography (EtOAc:*n*-Hexane = 1:1 → MeOH:CH_2_Cl_2_ = 1:10) gave **GHN061** (327.1 mg, 99% yield) as a yellowish foam. **TLC**: *R*_f_ = 0.35 (MeOH:CH_2_Cl_2_ = 1:10, KMnO_4_). ^**1**^**H NMR** (600 MHz, (CD_3_)_2_CO, −40 °C) δ 5.88–5.82 (m, 1H), 5.67 (d, *J* = 7.3 Hz, 1H), 5.53 (d, *J* = 10.3 Hz, 1H), 5.38–5.34 (m, 1H), 5.16 (d, *J* = 2.3 Hz, 1H), 5.00 (dt, *J* = 12.4, 4.3 Hz, 1H), 4.73–4.67 (m, 1H), 4.01 (dd, *J* = 15.8, 7.2 Hz, 1H), 3.14 (dd, *J* = 10.3, 5.1 Hz, 1H), 2.94–2.80 (m, 2H), 2.69–2.61 (m, 1H), 2.57–2.42 (m, 4H), 2.41 (s, 3H), 2.23 (s, 3H), 2.23 (s, 3H), 2.22–2.00 (m, 4H), 1.95 (s, 3H), 1.90–1.74 (m, 3H), 1.66–1.35 (m, 7H), 1.50 (s, 3H), 1.17–1.04 (m, 1H), 1.10 (d, *J* = 7.1 Hz, 3H), 0.86 (s, 3H), 0.85 (t, *J* = 7.4 Hz, 3H). ^**13**^**C NMR** (151 MHz, (CD_3_)_2_CO, −40 °C) δ 172.3, 172.2, 171.9, 171.9, 170.7, 170.2, 139.9, 122.6, 81.5, 81.4, 74.3, 73.6, 69.9, 69.2, 65.2, 60.6, 54.7, 54.6, 44.2, 40.3, 35.9, 34.2, 33.0, 32.6, 27.1, 26.2, 24.7, 22.6, 22.2, 22.1, 21.4, 18.4, 18.0, 13.6, 10.3, 10.0. **HRMS-ESI** (*m*/*z*) calcd for C_38_H_56_NO_13_ [M + H]^+^ 734.3746, found 734.3741. **HPLC purity**: t_R_ = 13.3 min, 99.4% (λ = 210 nm).

#### Synthesis of **GHN071**

Under an ambient atmosphere, a 4 mL vial equipped with a stir bar was charged with **ExcB** (17.7 mg, 0.0298 mmol, 1.0 equiv), 3-(4-methylpiperazin-1-yl)propanoic acid (10.3 mg, 0.0596 mmol, 2.0 equiv), DMAP (1.0 mg, 8.9 μmol, 0.3 equiv), EDCI (17.1 mg, 0.0893 mmol, 3.0 equiv) and dried DMF (0.3 mL). The vial was sealed with a Teflon cap, and the resulting solution was stirred at room temperature. After 18 h, the reaction mixture was diluted with EtOAc and the organic layer was washed with H_2_O. The organic layer was dried over MgSO_4_, filtered, and concentrated by rotary evaporation. The residue was purified by flash column chromatography (EtOAc:*n*-Hexane = 1:1 → MeOH:CH_2_Cl_2_ = 1:10) gave **GHN071** (13.8 mg, 62% yield) as a yellowish foam. **TLC**: R*_f_* = 0.23 (MeOH:CH_2_Cl_2_ = 1:10, KMnO_4_). ^**1**^**H NMR** (600 MHz, (CD_3_)_2_CO, −40 °C) δ 5.85 (dd, *J* = 7.3, 1.3 Hz, 1H), 5.67 (d, *J* = 7.2 Hz, 1H), 5.53 (d, *J* = 10.3 Hz, 1H), 5.37 (dt, *J* = 7.4, 1.7 Hz, 1H), 5.16 (d, *J* = 2.4 Hz, 1H), 5.00 (ddd, *J* = 12.4, 5.0, 3.6 Hz, 1H), 4.70 (dd, *J* = 4.0, 2.1 Hz, 1H), 4.02 (dd, *J* = 15.9, 7.2 Hz, 1H), 3.14 (dd, *J* = 10.4, 5.1 Hz, 1H), 2.77–2.70 (m, 2H), 2.69–2.59 (m, 3H), 2.57–2.42 (m, 4H), 2.41 (s, 3H), 2.23 (s, 6H), 2.21–2.06 (m, 3H), 2.13 (s, 3H), 2.03–1.89 (m, 5H), 1.95 (s, 3H), 1.89–1.82 (m, 1H), 1.56–1.46 (m, 2H), 1.51 (s, 3H), 1.10 (d, *J* = 7.1 Hz, 3H), 0.86 (s, 3H), 0.85 (t, *J* = 7.4 Hz, 3H). ^**13**^**C NMR** (151 MHz, (CD_3_)_2_CO, −40 °C) δ 172.3, 172.1, 171.9, 171.8, 170.7, 170.1, 139.9, 122.6, 81.5, 74.2, 73.7, 69.8, 69.2, 65.3, 60.5, 55.4, 55.4, 54.0, 53.3, 53.2, 46.1, 44.2, 40.3, 35.9, 34.3, 33.0, 32.8, 27.2, 22.6, 22.2, 22.2, 21.4, 18.4, 18.0, 13.7, 10.3, 10.2. **HRMS-ESI** (*m*/*z*) calcd for C_38_H_57_N_2_O_13_ [M + H]^+^ 749.3855, found 749.3864.

**HPLC purity**: *t*_R_ = 9.7 min, 97.6% (λ = 210 nm).

#### Synthesis of **GHN083**

Under an ambient atmosphere, a 4 mL vial equipped with a stir bar was charged with **ExcB** (21.6 mg, 0.0363 mmol, 1.0 equiv), 3-(azepan-1-yl)propanoic acid hydrochloride (15.1 mg, 0.0726 mmol, 2.0 equiv), DIPEA (2 drops), DMAP (1.3 mg, 11.0 μmol, 0.3 equiv), EDCI (20.9 mg, 0.1090 mmol, 3.0 equiv) and dried CH_2_Cl_2_ (0.4 mL). The vial was sealed with a Teflon cap, and the resulting solution was stirred at room temperature. After 18 h, the reaction mixture was diluted with EtOAc and the organic layer was washed with H_2_O. The organic layer was dried over MgSO_4_, filtered, and concentrated by rotary evaporation. The residue was purified by flash column chromatography (EtOAc:*n*-Hexane = 1:1 → MeOH:CH_2_Cl_2_ = 1:10) gave **GHN083** (28.8 mg, quantitative yield) as a yellowish foam. **TLC**: *R*_f_ = 0.48 (MeOH:CH_2_Cl_2_ = 1:10, KMnO_4_). ^**1**^**H NMR** (600 MHz, (CD_3_)_2_CO, −40 °C) δ 5.88–5.80 (m, 1H), 5.67 (d, *J* = 7.3 Hz, 1H), 5.53 (d, *J* = 10.3 Hz, 1H), 5.41–5.33 (m, 1H), 5.16 (d, *J* = 2.4 Hz, 1H), 5.00 (ddd, *J* = 12.5, 5.1, 3.7 Hz, 1H), 4.75–4.65 (m, 1H), 4.01 (dd, *J* = 15.8, 7.2 Hz, 1H), 3.15 (dd, *J* = 10.4, 5.1 Hz, 1H), 2.72 (br s, 2H), 2.66 (td, *J* = 7.0, 5.2 Hz, 1H), 2.57 (br s, 4H), 2.44 (br s, 2H), 2.41 (s, 3H), 2.23 (s, 3H), 2.22 (s, 3H), 2.21–2.02 (m, 4H), 1.95 (s, 3H), 1.92–1.84 (m, 1H), 1.61–1.45 (m, 10H), 1.50 (s, 3H), 1.11 (d, *J* = 7.1 Hz, 3H), 0.86 (s, 3H), 0.85 (t, *J* = 7.3 Hz, 3H). ^**13**^**C NMR** (151 MHz, (CD_3_)_2_CO, −40 °C) δ 172.3, 172.1, 172.0, 171.9, 170.7, 170.1, 139.9, 122.6, 81.5, 74.2, 73.6, 69.9, 69.2, 65.3, 60.5, 55.1, 54.2, 44.2, 40.3, 35.9, 34.3, 33.6 (br), 33.0, 27.2, 27.2, 22.6, 22.2, 21.4, 18.4, 18.1, 13.7, 10.3, 10.1. **HRMS-ESI** (*m*/*z*) calcd for C_39_H_58_NO_13_ [M + H]^+^ 748.3903, found 748.3900. **HPLC purity**: *t*_R_ = 13.9 min, 96.1% (λ = 210 nm).

#### Synthesis of **GHN087**

Under ambient atmosphere, a 4 mL vial equipped with a stir bar was charged with **ExcB** (23.5 mg, 0.0395 mmol, 1.0 equiv), dried Et_3_N (12.0 mg, 0.1185 mmol, 3.0 equiv), dried CH_2_Cl_2_ (0.4 mL) and 2-(piperidin-1-yl)ethane-1-sulfonyl chloride hydrochloride (19.6 mg, 0.0790 mmol, 2.0 equiv). The vial was sealed with a Teflon cap, and the resulting solution was stirred at room temperature. After 24 h, the reaction mixture was quenched by H_2_O. After the separation, the aqueous layer was extracted with CH_2_Cl_2_. The combined organic layers were dried over MgSO_4_, filtered, and concentrated by rotary evaporation. The residue was purified by flash column chromatography (EtOAc:*n*-Hexane = 0:1 → 1:1 → 5:1) to afford **GHN087** (24.5 mg, 81% yield) as a yellowish foam.

**TLC**: *R*_f_ = 0.28 (EtOAc: *n*-Hexane = 1:1, KMnO_4_). ^**1**^**H NMR** (600 MHz, (CD_3_)_2_CO, −40 °C) δ 5.80 (dd, *J* = 7.3, 1.7 Hz, 1H), 5.66 (d, *J* = 7.2 Hz, 1H), 5.55 (d, *J* = 10.2 Hz, 1H), 5.32 (d, *J* = 7.3 Hz, 1H), 5.16 (d, *J* = 2.3 Hz, 1H), 4.94 (dt, *J* = 12.6, 4.1 Hz, 1H), 4.76–4.72 (m, 1H), 4.00 (dd, *J* = 15.9, 7.0 Hz, 1H), 3.45–3.33 (m, 2H), 3.18 (dd, *J* = 10.2, 5.1 Hz, 1H), 2.96–2.76 (m, 3H), 2.65 (hept, *J* = 7.1, 6.4 Hz, 2H), 2.41 (s, 3H), 2.34–2.28 (m, 1H), 2.25 (s, 3H), 2.23 (s, 3H), 2.22–2.06 (m, 4H), 1.97–1.84 (m, 2H), 1.91 (s, 3H), 1.61–1.39 (m, 11H), 1.15 (d, *J* = 7.1 Hz, 3H), 0.87 (s, 3H), 0.85 (t, *J* = 7.4 Hz, 3H). ^**13**^**C NMR** (151 MHz, (CD_3_)_2_CO, −40 °C) δ 172.2, 172.1, 172.0, 170.7, 170.1, 139.7, 122.6, 81.3, 78.4, 74.2, 73.6, 69.3, 65.1, 60.8, 54.4, 53.1, 48.2, 44.1, 40.5, 35.9, 34.5, 34.1, 28.8, 26.3, 24.6, 22.6, 22.2, 22.1, 21.4, 18.4, 18.0, 13.6, 10.1, 10.1. **HRMS-ESI** (*m*/*z*) calcd for C_37_H_56_NO_14_S [M + H]^+^ 770.3416, found 770.3410.

**HPLC purity**: *t*_R_ = 13.7 min, 98.0% (λ = 210 nm).

#### Synthesis of **GHN038**

Under ambient atmosphere, a 4 mL vial equipped with a stir bar was charged with **ExcB** (54.1 mg, 0.091 mmol, 1.00 equiv), SeO_2_ (54.5 mg, 0.455 mmol, 5.00 equiv) and 1,4-dioxane (1.0 mL). The vial was sealed with a Teflon cap, and the reaction mixture was stirred at 90 °C. After 16 h, the reaction mixture was cooled to room temperature, filtered through a short pad of Celite. The filtrate was concentrated under reduced pressure. The residue was purified by flash column chromatography (EtOAc:*n*-Hexane = 0:1 → 2:3 → 1:1) to afford **GHN038** (49.4 mg, 89% yield) as a colorless solid. **MP**: 132–134 °C. **TLC**: *R*_f_ = 0.30 (EtOAc: *n*-Hexane = 1:1, KMnO_4_). ^**1**^**H NMR** (600 MHz, (CD_3_)_2_CO, −40 °C) δ 9.69 (d, *J* = 1.6 Hz, 1H), 6.88 (dd, *J* = 7.5, 2.1 Hz, 1H), 5.96 (dd, *J* = 7.4, 1.5 Hz, 1H), 5.71 (dd, *J* = 7.6, 2.7 Hz, 1H), 5.64 (d, *J* = 10.4 Hz, 1H), 5.19 (d, *J* = 2.5 Hz, 1H), 4.59–4.51 (m, 1H), 4.20–4.11 (m, 1H), 3.96–3.79 (m, 2H), 2.87 (dd, *J* = 10.4, 5.0 Hz, 1H), 2.57–2.50 (m, 1H), 2.46 (d, *J* = 15.1 Hz, 1H), 2.42 (s, 3H), 2.21 (s, 3H), 2.22–2.04 (m, 2H), 1.99 (s, 3H), 1.89–1.81 (m, 1H), 1.79–1.71 (m, 1H), 1.55 (s, 3H), 1.54–1.49 (m, 2H), 1.03 (d, *J* = 7.2 Hz, 3H), 0.90 (t, *J* = 7.4 Hz, 3H), 0.79 (s, 3H). ^**13**^**C NMR** (151 MHz, (CD_3_)_2_CO, −40 °C) δ 194.9, 172.2, 171.9, 171.7, 170.8, 170.0, 148.1, 144.5, 82.0, 81.3, 74.6, 73.9, 69.9, 65.8, 65.3, 60.4, 44.7, 40.8, 36.0, 35.9, 29.8, 27.7, 22.7, 22.2, 21.4, 18.5, 18.0, 13.7, 10.0, 9.4. **HRMS-ESI** (*m*/*z*) calcd for C_30_H_40_O_14_Na [M + Na]^+^ 647.2370, found 647.2361. **HPLC purity**: *t*_R_ = 12.8 min, 100.0% (λ = 210 nm). Crystals suitable for X-ray crystallography were grown using layer diffusion of *n*-hexane into a solution of **GHN038** in CH_2_Cl_2_.

#### Synthesis of **GHN075**

Under an ambient atmosphere, a 4 mL vial equipped with a stir bar was charged with **GHN038** (18.2 mg, 0.0299 mmol, 1.0 equiv), CH_2_Cl_2_/pyridine (14:1, 0.3 mL) and *O*-methylhydroxylamine hydrochloride (3.0 mg, 0.0359 mmol, 1.2 equiv). The vial was sealed with a Teflon cap, and the resulting solution was stirred at room temperature. After 18 h, the reaction mixture was directly purified by flash column chromatography (EtOAc:*n*-Hexane = 0:1 → 1:1) gave **GHN075** (18.4 mg, 97% yield, *E:Z* = 98:2, determined by HPLC analysis of isolated material) as a colorless solid. **TLC**: *R*_f_ = 0.23 (EtOAc: *n*-Hexane = 1:1, UV 254 nm, KMnO_4_). ^**1**^**H NMR** (600 MHz, (CD_3_)_2_CO, −40 °C) δ 7.97 (s, 1H), 6.06–5.99 (m, 1H), 5.92 (d, *J* = 6.0 Hz, 1H), 5.83 (d, *J* = 7.4 Hz, 1H), 5.61 (d, *J* = 10.3 Hz, 1H), 5.17 (s, 1H), 4.58 (s, 1H), 4.22–4.15 (m, 1H), 3.97 (dd, *J* = 15.7, 7.3 Hz, 1H), 3.91–3.83 (m, 1H), 3.75 (s, 3H), 2.98 (dd, *J* = 10.4, 5.0 Hz, 1H), 2.57–2.48 (m, 2H), 2.41 (s, 3H), 2.21 (s, 3H), 2.19–2.08 (m, 2H), 2.05 (s, 3H), 1.91–1.80 (m, 1H), 1.80–1.73 (m, 1H), 1.58–1.46 (m, 2H), 1.53 (s, 3H), 1.04 (d, *J* = 7.2 Hz, 3H), 0.89 (t, *J* = 7.4 Hz, 3H), 0.81 (s, 3H). ^**13**^**C NMR** (151 MHz, (CD_3_)_2_CO, −40 °C) δ 172.2, 172.1, 171.7, 170.7, 169.9, 150.1, 138.1, 133.2, 82.0, 81.5, 75.4, 74.0, 69.7, 65.8, 65.4, 61.9, 60.5, 44.6, 40.6, 36.1, 36.0, 29.8, 29.2, 22.7, 22.2, 21.4, 18.6, 18.1, 13.9, 10.0, 9.5. **HRMS-ESI** (*m*/*z*) calcd for C_31_H_43_NO_13_Na [M + Na]^+^ 660.2627, found 660.2634. **HPLC purity**: *t*_R_ = 13.7 min, 1.6%; t_R_ = 15.2 min, 97.4% (λ = 210 nm).

#### Synthesis of **GHN079**

Under an ambient atmosphere, a 4 mL vial equipped with a stir bar was charged with **GHN038** (103.7 mg, 0.170 mmol, 1.0 equiv), CH_2_Cl_2_/pyridine (14:1, 2.0 mL) and *O*-(prop-2-yn-1-yl)hydroxylamine hydrochloride (22.0 mg, 0.204 mmol, 1.2 equiv). The vial was sealed with a Teflon cap, and the resulting solution was stirred at room temperature. After 18 h, the reaction mixture was directly purified by flash column chromatography (EtOAc:*n*-Hexane = 0:1 → 2:3 → 1:1) gave **GHN079** (109.6 mg, 97% yield, *E*:*Z* = 98:2, determined by HPLC analysis of isolated material) as a colorless solid. **TLC** (EtOAc: *n*-Hexane = 1:1): *R*_f_ = 0.25 (UV 254 nm, KMnO_4_). ^**1**^**H NMR** (600 MHz, (CD_3_)_2_CO, −40 °C) δ 8.04 (s, 1H), 6.09 (dd, *J* = 7.5, 2.0 Hz, 1H), 5.92 (dd, *J* = 6.6, 2.0 Hz, 1H), 5.84 (d, *J* = 7.4 Hz, 1H), 5.62 (d, *J* = 10.4 Hz, 1H), 5.16 (d, *J* = 2.5 Hz, 1H), 4.65 (dd, *J* = 15.8, 2.5 Hz, 1H), 4.61–4.55 (m, 2H), 4.17 (d, *J* = 4.0 Hz, 1H), 4.01 (dd, *J* = 15.6, 6.7 Hz, 1H), 3.91–3.84 (m, 1H), 3.26 (t, *J* = 2.4 Hz, 1H), 2.96 (dd, *J* = 10.4, 5.1 Hz, 1H), 2.58–2.49 (m, 2H), 2.41 (s, 3H), 2.21 (s, 3H), 2.17–2.05 (m, 2H), 2.05 (s, 3H), 1.90–1.80 (m, 1H), 1.79–1.72 (m, 1H), 1.58–1.48 (m, 2H), 1.53 (s, 3H), 1.04 (d, *J* = 7.3 Hz, 3H), 0.89 (t, *J* = 6.0 Hz, 3H), 0.81 (s, 3H). ^**13**^**C NMR** (151 MHz, (CD_3_)_2_CO, −40 °C) δ 172.2, 172.0, 171.9, 170.7, 169.9, 151.2, 137.8, 134.2, 82.0, 81.6, 79.9, 76.7, 75.2, 74.0, 69.7, 65.8, 65.4, 61.9, 60.5, 44.6, 40.6, 36.1, 36.1, 30.1, 29.2, 22.7, 22.3, 21.4, 18.5, 18.0, 13.9, 10.0, 9.5. **HRMS-FAB** (*m*/*z*) calcd for C_33_H_43_NO_13_Na [M + Na]^+^ 684.2627, found 684.2620. **HPLC purity**: *t*_R_ = 13.0 min, 1.5%; *t*_R_ = 14.0 min, 97.9% (λ = 254 nm).

#### Synthesis of **GHN101**

Under an ambient atmosphere, a 4 mL vial equipped with a stir bar was charged with **GHN065** (34.1 mg, 0.055 mmol, 1.0 equiv), *O*-methylhydroxylamine hydrochloride (5.0 mg, 0.060 mmol, 1.1 equiv), HATU (31.1 mg, 0.082 mmol, 1.5 equiv), DIPEA (21.2 mg, 0.164 mmol, 3.0 equiv) and dried DMF (0.5 mL). The vial was sealed with a Teflon cap, and the resulting solution was stirred at room temperature. After 18 h, the reaction mixture was diluted with EtOAc and the organic layer was washed with H_2_O and brine. The organic layer was dried over MgSO_4_, filtered, and concentrated by rotary evaporation. The residue was purified by flash column chromatography (EtOAc:*n*-Hexane = 1:1 → 3:1) gave **GHN101** (21.2 mg, 59% yield) as a colorless oil. **TLC** (EtOAc: *n*-Hexane = 5:1): R*_f_* = 0.43 (KMnO_4_). ^**1**^**H NMR** (600 MHz, (CD_3_)_2_CO, −40 °C) δ 11.61 (s, 1H), 6.11 (dd, *J* = 7.5, 2.3 Hz, 1H), 5.75 (d, *J* = 8.2 Hz, 1H), 5.60 (d, *J* = 10.3 Hz, 1H), 5.53 (dd, *J* = 7.2, 2.4 Hz, 1H), 5.16 (d, *J* = 2.5 Hz, 1H), 4.55 (d, *J* = 3.1 Hz, 1H), 4.09 (d, *J* = 4.4 Hz, 1H), 4.01–3.95 (m, 1H), 3.91 (dd, *J* = 15.7, 7.5 Hz, 1H), 3.70 (s, 3H), 2.99 (dd, *J* = 10.4, 4.9 Hz, 1H), 2.72 (d, *J* = 15.6 Hz, 1H), 2.57–2.51 (m, 1H), 2.40 (s, 3H), 2.20 (s, 3H), 2.17–2.08 (m, 1H), 2.08–1.99 (overlapped with residual solvent peak, 5H), 1.89–1.82 (m, 1H), 1.79–1.73 (m, 1H), 1.51 (s, 3H), 1.56–1.44 (m, 2H), 1.01 (d, *J* = 7.2 Hz, 3H), 0.88 (t, *J* = 7.4 Hz, 3H), 0.78 (s, 3H). ^**13**^**C NMR** (151 MHz, (CD_3_)_2_CO, −40 °C) δ 172.2, 172.1, 171.7, 170.7, 170.1, 165.1, 138.9, 129.4, 82.1, 81.4, 74.8, 74.1, 69.5, 65.7, 65.4, 63.8, 60.1, 44.6, 40.5, 35.9, 35.6, 30.3, 22.7, 22.1, 21.4, 18.5, 18.1, 13.7, 10.0, 9.3. **HRMS-ESI** (*m*/*z*) calcd for C_31_H_43_NO_14_Na [M + Na]^+^ 676.2576, found 676.2573. **HPLC purity**: *t*_R_ = 6.8 min, 98.8% (λ = 210 nm).

#### Synthesis of **GHN093**

Under an ambient atmosphere, a 4 mL vial equipped with a stir bar was charged with **GHN075** (32.3 mg, 0.051 mmol, 1.0 equiv), 3-(piperidin-1-yl)propanoic acid (10.4 mg, 0.066 mmol, 1.3 equiv), DMAP (1.9 mg, 0.015 mmol, 0.3 equiv), EDCI (19.4 mg, 0.101 mmol, 2.0 equiv) and dried CH_2_Cl_2_ (0.5 mL). The vial was sealed with a Teflon cap, and the resulting solution was stirred at room temperature. After 20 h, the reaction mixture was directly purified by flash column chromatography (EtOAc:*n*-Hexane = 1:1 → MeOH: CH_2_Cl_2_ = 1:10) gave **GHN093** (37.0 mg, 94% yield, *E*:*Z* = 98:2, determined by HPLC analysis of isolated material) as a colorless oil. **TLC** (MeOH: CH_2_Cl_2_ = 1:10): *R*_f_ = 0.25 (UV 254 nm). ^**1**^**H NMR** (600 MHz, (CD_3_)_2_CO, −40 °C) δ 7.99 (s, 1H), 6.04 (dd, *J* = 7.4, 1.9 Hz, 1H), 5.94 (dd, *J* = 6.8, 2.4 Hz, 1H), 5.83 (d, *J* = 7.5 Hz, 1H), 5.59 (d, *J* = 10.3 Hz, 1H), 5.18 (d, *J* = 2.4 Hz, 1H), 4.96 (dt, *J* = 12.6, 4.4 Hz, 1H), 4.63 (s, 1H), 3.97 (dd, *J* = 15.6, 7.0 Hz, 1H), 3.76 (s, 3H), 3.06 (dd, *J* = 10.3, 5.0 Hz, 1H), 2.82 (br s, 2H), 2.71–2.65 (m, 1H), 2.53 (d, *J* = 15.7 Hz, 1H), 2.50–2.39 (m, 4H), 2.42 (s, 3H), 2.22 (s, 3H), 2.18–2.13 (m, 1H), 2.12–2.07 (m, 1H), 2.08 (s, 3H), 2.05–1.98 (m, 1H), 1.91–1.83 (m, 1H), 1.76 (br s, 2H), 1.65–1.34 (m, 7H), 1.51 (s, 3H), 1.17–1.06 (m, 1H), 1.11 (d, *J* = 7.2 Hz, 3H), 0.89 (t, *J* = 7.4 Hz, 3H), 0.84 (s, 3H). ^**13**^**C NMR** (151 MHz, (CD_3_)_2_CO, −40 °C) δ 172.2, 171.9, 171.9, 171.7, 170.7, 169.9, 150.2, 138.0, 133.3, 81.4, 81.4, 75.3, 74.0, 69.8, 69.6, 65.0, 61.9, 60.6, 54.8, 54.6, 44.6, 40.6, 36.0, 33.2, 32.8, 29.1, 26.9, 26.3, 24.8, 22.6, 22.1, 21.4, 18.6, 17.9, 13.9, 10.4, 10.0.

**HRMS-ESI** (*m*/*z*) calcd for C_39_H_56_N_2_O_14_Na [M + Na]^+^ 799.3624, found 799.3618.

**UPLC purity**: *t*_R_ = 2.40 min, 2.2%; *t*_R_ = 2.50 min, 96.6% (λ = 254 nm)

#### Synthesis of **GHN105**

Under an ambient atmosphere, a 20 mL vial equipped with a stir bar was charged with **GHN079** (273.1 mg, 0.413 mmol, 1.0 equiv), 3-(piperidin-1-yl)propanoic acid (84.3 mg, 0.537 mmol, 1.3 equiv), DMAP (15.1 mg, 0.124 mmol, 0.3 equiv), EDCI (158.3 mg, 0.826 mmol, 2.0 equiv) and dried CH_2_Cl_2_ (4.0 mL). The vial was sealed with a Teflon cap, and the resulting solution was stirred at room temperature. After 20 h, the reaction mixture was directly purified by flash column chromatography (EtOAc:*n*-Hexane = 1:1 → MeOH: CH_2_Cl_2_ = 1:10) gave **GHN105** (304.6 mg, 92% yield, *E*: *Z* = 98:2, determined by HPLC analysis of isolated material) as a colorless solid. **TLC** (MeOH: CH_2_Cl_2_ = 1:10): *R*_f_ = 0.43 (UV 254 nm). ^**1**^**H NMR** (600 MHz, (CD_3_)_2_CO, −40 °C) δ 8.07 (s, 1H), 6.10 (dd, *J* = 7.4, 1.9 Hz, 1H), 5.94 (dd, *J* = 7.2, 2.4 Hz, 1H), 5.84 (dd, *J* = 7.4, 1.2 Hz, 1H), 5.60 (d, *J* = 10.3 Hz, 1H), 5.17 (d, *J* = 2.3 Hz, 1H), 4.96 (dt, *J* = 12.5, 4.4 Hz, 1H), 4.67 (dd, *J* = 15.8, 2.5 Hz, 1H), 4.64–4.61 (m, 1H), 4.59 (dd, *J* = 15.8, 2.4 Hz, 1H), 4.00 (ddd, *J* = 15.9, 7.0, 1.4 Hz, 1H), 3.30–3.26 (m, 1H), 3.05 (dd, *J* = 10.3, 5.0 Hz, 1H), 2.84 (br s, 2H), 2.68 (td, *J* = 6.9, 3.5 Hz, 1H), 2.54 (d, *J* = 15.7 Hz, 1H), 2.52–2.38 (m, 4H), 2.42 (s, 3H), 2.27–2.20 (m, 1H), 2.23 (s, 3H), 2.14–2.09 (m, 1H), 2.08 (s, 3H), 2.03–1.99 (m, 1H), 1.90–1.84 (m, 1H), 1.77 (br s, 2H), 1.68–1.30 (m, 7H), 1.52 (s, 3H), 1.17–1.04 (m, 1H), 1.11 (d, *J* = 7.1 Hz, 3H), 0.89 (t, *J* = 7.4 Hz, 3H), 0.85 (s, 3H). ^**13**^**C NMR** (151 MHz, (CD_3_)_2_CO, −40 °C) δ 172.2, 171.9, 171.9, 171.8, 170.7, 169.9, 151.4, 137.7, 134.3, 81.4, 80.0, 76.7, 75.2, 74.0, 69.8, 69.6, 65.0, 61.9, 60.6, 54.7, 54.6, 44.6, 40.7, 36.0, 33.2, 32.7, 29.2, 26.9, 26.2, 24.7, 22.6, 22.3, 21.4, 18.5, 17.9, 13.9, 10.4, 10.0. **HRMS-ESI** (*m*/*z*) calcd for C_41_H_56_N_2_O_14_Na [M + Na]^+^ 823.3624, found 823.3615. **UPLC purity**: *t*_R_ = 2.46 min, 1.7%; *t*_R_ = 2.53 min, 97.7% (λ = 254 nm).

#### Synthesis of **GHN107**

Under an ambient atmosphere, a 4 mL vial equipped with a stir bar was charged with **GHN079** (54.7 mg, 0.083 mmol, 1.0 equiv), 3-(4-methylpiperazin-1-yl)propanoic acid (17.1 mg, 0.099 mmol, 1.2 equiv), DMAP (2.0 mg, 0.017 mmol, 0.2 equiv), EDCI (31.7 mg, 0.165 mmol, 2.0 equiv) and dried CH_2_Cl_2_ (1.0 mL). The vial was sealed with a Teflon cap, and the resulting solution was stirred at room temperature. After 20 h, the reaction mixture was directly purified by flash column chromatography (EtOAc:*n*-Hexane = 1:1 → MeOH: CH_2_Cl_2_ = 1:10) gave **GHN107** (50.1 mg, 74% yield, *E*:*Z* = 98:2, determined by HPLC analysis of isolated material) as a colorless oil. **TLC** (MeOH: CH_2_Cl_2_ = 1:10): R*_f_* = 0.33 (UV 254 nm). ^**1**^**H NMR** (600 MHz, (CD_3_)_2_CO, −40 °C) δ 8.07 (s, 1H), 6.11 (dd, *J* = 7.4, 1.9 Hz, 1H), 5.97–5.92 (m, 1H), 5.85 (dd, *J* = 7.3, 1.2 Hz, 1H), 5.60 (d, *J* = 10.3 Hz, 1H), 5.17 (d, *J* = 2.3 Hz, 1H), 4.97 (dt, *J* = 12.5, 4.6 Hz, 1H), 4.67 (dd, *J* = 15.9, 2.4 Hz, 1H), 4.65–4.62 (m, 1H), 4.59 (dd, *J* = 15.8, 2.4 Hz, 1H), 4.01 (dd, *J* = 15.7, 6.9 Hz, 1H), 3.29–3.25 (m, 1H), 3.05 (dd, *J* = 10.3, 5.0 Hz, 1H), 2.80–2.72 (m, 2H), 2.72–2.64 (m, 3H), 2.58–2.49 (m, 3H), 2.49–2.38 (m, 2H), 2.43 (s, 3H), 2.27–2.21 (m, 1H), 2.23 (s, 3H), 2.21–2.14 (m, 3H), 2.14–2.10 (m, 1H), 2.08 (s, 3H), 2.04–1.97 (m, 5H), 1.90–1.83 (m, 1H), 1.60–1.48 (m, 2H), 1.54 (s, 3H), 1.11 (d, *J* = 7.1 Hz, 3H), 0.89 (t, *J* = 7.4 Hz, 3H), 0.85 (s, 3H). ^**13**^**C NMR** (151 MHz, (CD_3_)_2_CO, −40 °C) δ 172.2, 171.9, 171.7, 170.7, 169.9, 151.4, 137.8, 134.3, 81.4, 80.0, 76.7, 75.2, 74.0, 69.8, 69.6, 65.0, 61.9, 60.6, 55.2, 53.9, 53.2, 46.1, 44.6, 40.7, 36.1, 33.2, 32.7, 29.2, 26.9, 22.6, 22.3, 21.4, 18.5, 17.8, 13.9, 10.4, 10.2. **HRMS-ESI** (*m*/*z*) calcd for C_41_H_57_N_3_O_14_Na [M + Na]^+^ 838.3733, found 838.3741. **UPLC purity**: *t*_R_ = 2.26 min, 1.2%; *t*_R_ = 2.35 min, 97.0% (λ = 254 nm).

### Cell Lines and Cell Culture

HEK293T and NIH3T3 cells were maintained in Dulbecco’s High Glucose Modified Eagle’s Medium (DMEM, HyClone) supplemented with 10% fetal bovine serum (FBS, Biological Industries), penicillin (100 U/mL) and streptomycin (100 μg/mL). THP1 monocytes were maintained in RPMI 1640 Medium (Hyclone, SH30605.01) supplemented with 10% FBS, penicillin (100 U/mL) and streptomycin (100 μg/mL). THP1 macrophages were differentiated from THP1 monocytes by 48 h incubation with 20 nM phorbol 12-myristate 13-acetate (PMA, Sigma). THP1-Dual KI hSTING cells harboring either WT (R232) or S154 STING variants (Invivogen, thpd-r232, thpd-s154) were grown in RPMI 1640 medium supplemented with 10% FBS, penicillin (100 U/mL), streptomycin (100 μg/mL) and 100 μg/mL normocin (Invivogen, ant-nr-1). Blasticidin (10 μg/mL, Invivogen, ant-bl-05) and Zeocin (100 μg/mL, Invivogen, ant-zn-1) were added to the growth medium to maintain selection pressure of THP1-Dual KI hSTING cells. Human mesenchymal stem cells (hMSCs; Lonza Walkersville Inc., PT-2501) were cultured in Iscove’s Modified Dulbecco’s Medium (IMDM, HyClone, SH30228.02) supplemented with 10% FBS, penicillin (10 U/mL), streptomycin (10 μg/mL) and 10 ng/mL of bFGF (peprotech #100-18B-100UG). All cell lines were grown at 37 °C in a humidified 5% CO_2_ atmosphere. Cell lines were tested and confirmed to be mycoplasma-free using the MycoAlert PLUS Mycoplasma Detection kit (Lonza, LT07–318).

### THP1 Luciferase Reporter Assay

THP1-Dual KI hSTING cells were seeded in 96-well plates at a density of 1 × 10^5^ cells/well in RPMI 1640 medium supplemented with 10% FBS, penicillin (100 U/mL) and streptomycin (100 μg/mL). Cells were treated with indicated concentrations of GHN105 or equal volume of DMSO for 1 h, followed by treatment with 30 μg/mL 2′3′-cGAMP (MedChemExpress, HY-100564A) in a final volume of 200 μL per well. After 24 h incubation, 20 μL of culture supernatant was collected into black opaque plates prior to addition of 50 μL of freshly prepared QUANTI-Luc reagent (Invivogen, rep-qlc4lg1). Luminescence was measured on an Envision plate reader (Promega, GM3000).

### ELISA Analyses of IFN-β, CXCL10, IL-6 and TNFα Levels

Human IFN-β production was measured in the culture supernatants of compound-treated THP1 macrophages with additional cGAMP induction. Briefly, THP1 monocytes at a density of 5 × 10^3^ cells per well in a 96-well plate were stimulated with 20 nM of PMA for 48 h. Cells were starved by replacing medium with serum-free RPMI 1640 for 1 h. After removing the medium, cells were replenished with fresh serum-free media containing 10 μM of indicated compounds for 1 h prior to the addition of cGAMP and Lipofectamine 2000 at a final concentration of 4 μg/mL in a final volume of 100 μL per well. After 24 h incubation, culture supernatants were collected and stored at −80 °C prior to cytokine detection. IFN-β in culture supernatants were quantified by human IFN-β DuoSet ELISA kit (R&D Systems, DY814) according to manufacturer’s recommendations. For mouse serum and plasma, IFN-β, CXCL10, IL-6 were measured using the ELISA kits DY8234, DY466, DY406 from R&D Systems, respectively, and TNF-α using ELISA kit (Invitrogen, 88-7324-88) according to manufacturer’s recommendations. Sample cytokine concentrations were calculated using linear regression curve fit by plotting the log of cytokine concentrations versus the log of the absorbance (450 nm) values.

### Cell Viability Assay

THP-1 cells were seeded in a 96-well plate at a density of 5 × 10^3^ cells per well in 150 μL of growth medium supplemented with 20 nM PMA for 48 h. Cells were treated with 10 μM of indicated compounds or vehicle (DMSO) prepared in culture medium to a final volume of 200 μL and incubated for 24 h. Cell viability was measured using the alamarBlue assay (DAL1025, Invitrogen) according to manufacturer’s recommendations. Fluorescence intensities (excitation 560 nm, emission 590 nm) of the samples were measured using a GloMax Discover microplate reader (Promega). Results were shown as % viability compared to control (DMSO). Each condition was carried out in biological duplicates.

### In-Gel Fluorescence Detection of GNH105-Labeled Proteins

STING expression plasmids were obtained in a previous study.^16^ For transient STING overexpression, constructs were transfected into HEK293T cells using the TransIT-LT1 Transfection Reagent (Mirus Bio, MIR2300) for 16 h prior to the probe labeling. Human or mouse STING overexpressing HEK293T cells were treated with 2 μM of GHN105 or equal volume of DMSO as control. After 15 min labeling, cells were washed thrice with ice-cold PBS and pelleted at 400 g for 5 min. Cells were flash-frozen in liquid nitrogen and stored at −80 °C prior to lysis. Frozen cells were lysed in SDS lysis buffer [4% SDS, 150 mM NaCl, 50 mM triethanolamine, pH 7.4, 2× EDTA-free protease inhibitor cocktail (Selleckchem, B14001), 10 mM phenylmethylsulfonyl fluoride, 50 U/mL SuperNuclease (Sino Biological, SSNP01)]. Protein concentrations were determined by BCA protein assay (Thermo Scientific, 23225). For in-gel fluorescence detection, 50 μg cell lysates were reacted with freshly made CuAAC reaction cocktail [100 μM azide-Cy5, 1 mM CuSO4, 1 mM tris(2-carboxyethyl) phosphine hydrochloride (TCEP), 100 μM tris[(1-benzyl-1H-1,2,3-triazol-4-yl)methyl]amine (TBTA)] in a total reaction volume of 50 μL for 1 h at room temperature protected from light. Proteins were chloroform–methanol precipitated and the protein pellet was washed twice with ice-cold methanol. Air-dried protein pellets were resuspended in 25 μL SDS buffer before the addition of 8.7 μL 4× SDS-loading buffer (20% glycerol, 125 mM Tris·HCl, pH 6.8, 4% SDS, 0.05% bromophenol blue) and 1.3 μL Bond-Breaker TCEP (Thermo Scientific, 77720). Samples were heated at 95 °C for 5 min, separated by SDS-PAGE, and imaged on a ChemiDoc MP Imaging System (Bio-Rad, 12003154). Cy5-associated signal was detected at excitation 625–650 nm/emission 675–725 nm. After fluorescence scanning, gels were either stained with Coomassie (BIO-HELIX, PS002-B500 ML) or transferred to PVDF membranes for Western blot analysis.

### Western Blot

The protein lysate lysed in SDS lysis buffer was resolved by SDS-PAGE and electrically transferred onto a PVDF membrane ((Millipore, IPVH85R). Immunoblot was probed with indicated primary follow by secondary antibodies. Blot images were visualized by chemiluminescent substrate (Thermo Scientific, 34580) and imaged on a ChemiDoc MP imaging system (Bio-Rad).

### Antibodies

The antibodies used in this study at the following dilutions for Western blot analyses: Antiphospho-STING (#72971, 1:1000), anti-STING (#13647, 1:1000), antiphospho-TBK1 (#5483, 1:1000), anti-TBK1 (#3504, 1:1000), antiphospho-IRF3 S396(#29047, 1:1000), anti-IRF3 (#4302, 1:1000), anti-α-tubulin (#2144, 1:2000) were purchased from Cell Signaling Technology. Antirabbit-HRP (#111-035-003, 1:20000) was purchased from Jackson ImmunoResearch Laboratories.

### HSV Infection of hMSCs and Viral Titer Measurement

hMSCs were seeded into 6-well plates at a density of 2 × 10^5^ cells per well. After overnight incubation, cells were pretreated with either 1 μM GHN105 or an equivalent volume of DMSO (as control) for 2 h. The cells were then infected with HSV-1 (#VI00053; National Infectious Diseases Bank, Taiwan) for 2 h in IMDM. After the infection period, the medium was replaced with either GHN105- or vehicle-containing IMDM, and the cells were cultured for an additional 28 h. Serum viral titers were determined using the TCID50 assay. For the TCID50 assay, Vero76 cells were seeded into 96-well plates at a density of 1 × 10^4^ cells per well and incubated for 24 h at 37 °C with 5% CO_2_. 10-fold serial dilutions of the viral stock were added to each well in quadruplicate (100 μL/well), and the cells were incubated for 3 days under the same conditions. After 3 days, cytopathic effects were observed under a microscope, and viral titers were calculated using the TCID50 calculator (Marco Binder, University of Heidelberg).

### In Silico hSTING-GHN105 Docking

The full-length hSTING protein structure, required for docking experiments, was obtained from the Protein Data Bank (PDB) with the PDB ID of 6NT5.^36^ The three-dimensional structures of the ligands were generated using the RDKit toolkit 2022.9.4^37^ within a Python programming language. The three-dimensional protein was prepared and protonated using the AMBER-FB15 force field.^38^ A three-step approach was employed for protein–ligand blind docking to identify potential ligand binding pockets and protein–ligand interactions. First, CB-Dock was used to determine cavities on the protein surface.^39^ Subsequently, AutoDock Vina was applied to perform docking according to the standard protein–ligand docking protocol.^40^ Third, covalent docking targeting the Cys91 residue was performed based on the results of the previous step. The covalent docking step explicitly specified the flexibility of four arginine residues (Arg83, Arg86, Arg94, and Arg95) following previous descripted covalent docking protocols using AutoDockFR and the AutoDock4 scoring function.^16,41^ The lowest-energy covalent docking poses for the hSTING-Cys91-GHN105 protein–ligand complex were obtained through this analysis. To further determine the membrane embedding position of the hSTING-Cys91-GHN105 complex, the bilayer builder function in CHARMM-GUI,^42^ along with PPM 2.0,^43^ was used to simulate the membrane environment.

### General Statement for Animal Use

All animal experiment procedures were performed according to the guidelines approved by the Institutional Animal Care and Use Committee (IACUC) of National Health Research Institutes (NHRI), Taiwan; NHRI-IACUC-111048, NHRI-IACUC-113051, and NHRI-IACUC-113021.

### Zebrafish Embryonic Toxicity Assay

Zebrafish embryonic toxicity assay was performed according to a modified OECD TG 236. Zebrafish (*Danio rerio*) were housed under standard conditions according to OECD TG 236 with an IACUC approved animal protocol. Fertilized zebrafish embryos were introduced to a Petri dish and kept in HEPES-buffered E3 (HE3) media (5 mM NaCl, 0.17 mM KCl, 0.33 mM CaCl_2_, 0.33 mM MgSO_4_, 10 mM HEPES, pH 7.2). For compound exposure, embryos at 16 cell-stage were transferred individually into wells of a flat-bottomed 96-well plate using 1000 μL wide-bore micropipette tips. Eight embryos were exposed to 40 μM of each compound or equal amount of DMSO as control in a total volume of 200 μL and incubated at 28.5 °C on a 14:10 light: dark cycle. Lethality and phenotypic observations were recorded visually based on the lethal end points (i.e., coagulation of the embryo, nondetachment of the tail and lack of heartbeat) of OECD TG 236 using a dissecting microscope at 72 hpf.

### In Vivo Pharmacokinetics Study

The animal studies were conducted according to the NHRI institutional animal care and committee-approved procedures. Male ICR mice (weighing approximately 25–28 g) were obtained from BioLASCO (Taiwan Co., Ltd., Ilan, Taiwan). A single 2 mg/kg (intravenous, i.v.), 10 mg/kg (oral, p.o.) and 15 mg/kg (intraperitoneal, i.p.) dose of the compound, formulated as a DMSO/Cremophor/95% glucose water, 10/20/70, v/v/v), was separately administered to mice. At 0 (before dosing), 0.03, 0.08, 0.25 (i.v. only), 0.5, 1, 2, 4, 6, 8, 16, and 24 h after dosing, a blood sample was collected from groups of three mice at each time point by cardiac puncture and plasma was separated from the blood by centrifugation and stored in a freezer (−80 °C) before analysis. All samples were analyzed for the compound by LC-MS/MS. Plasma concentration data were analyzed with a noncompartmental method.

### In Vivo Toxicity Study

Male ICR mice (*n* = 3 per group) were oral administered with vehicle (10 mL/kg) and GHN105 (100 mg/kg) once daily for 5 consecutive days. GHN105 was dissolved in DMA/Cremophor EL/5% dextrose solution (1:2:7, v/v/v). All animals were observed for clinical signs and body weight changes during the study period. The animals were euthanized with 100% CO_2_ at the end of the study.

### In Vivo Model for Pharmacological Inhibition of STING (diABZI)

C57BL/6J mice were purchased from the National Applied Research Laboratories and National Laboratory Animal Center, Taipei City, Taiwan. Animal care and handling followed the Animal Care and Use Policies and Guidelines during all experiments. The animal protocols were approved by the Institutional Animal Care and Use Committee, National Health Research Institute, Miaoli, Taiwan (Approval numbers: NHRI-IACUC-113051-A-S01). 6–8 weeks old male C57BL/6J mice were treated with GHN105 (100 mg/kg, p.o.) or vehicle control for 1 h prior to intraperitoneal administration of diABZI (2 mg/kg, i.p.) for 3.5 h. Mice were sacrificed, and blood samples were collected by cardiac puncture. Blood samples were left undisturbed at room temperature for 30 min for coagulation. After centrifugation at 13,000 ppm for 5 min, serum in the supernatant was collected for cytokine analysis.

### Delayed Treatment Acute Colitis Animal Model

Male C57BL/6J mice (8-week-old) were obtained from the National Applied Research Laboratories, National Laboratory Animal Center, Taipei City, Taiwan. The animal experiments were approved by the Institutional Animal Care and Use Committee, National Health Research Institutes, Miaoli, Taiwan (Approval numbers: NHRI-IACUC—113021-A-S01). Animal Care and Use Policies and Guidelines were followed during all experiments. Colitis was induced by the introduction of DSS (MW 36–50 kDa, MP Biomedicals, Cat#160110) into the drinking water of mice. For mock treatment groups, mice were kept on drinking water throughout the experiment. For in vivo validation of GHN105 in acute DSS-induced colitis, mice were first fed drinking water with 3% (w/v) DSS for 8 days (D0-D8) and then 3% (w/v) DSS with oral administration of vehicle or GHN105 (D8-D13; dose: 20 μg/mouse, 200 μL, daily). Mice were sacrificed on D14, as shown in Figure 4A. Body weight, stool softness, and blood in the stool were recorded daily. After sacrifice, the colons were harvested, followed by length measurement, and then subjected to histological study. For detection of GHN105-mSTING engagement, ∼2 cm colon tissue on the proximal end was harvested, rinsed in PBS, flash frozen in liquid nitrogen and stored at −80 °C until processing. Blood samples were taken via cardiac puncture and collected into K_2_-EDTA-coated anticoagulation tubes prior to centrifugation at 2000 g for 10 min to obtain plasma for cytokine analysis.

### Histological Studies

Briefly, the respective tissues were fixed overnight in 10% (v/v) formalin, dehydrated with different grades of alcohol and chloroform mixture, and embedded in molten paraffin wax. 7-μm-thick sections were prepared using a microtome, stained with H&E, and photographed using an Axio Imager A2 microscope (Zeiss). For immunohistochemistry, the paraffin sections were first deparaffinized by incubating in xylene for 5 min, then rehydration through different grades of alcohol and distilled water. The antigens were retrieved by immersing the slides with Tris-EDTA buffer (pH 9.0) at 95 °C for 30 min. Endogenous peroxidase activity was removed with 3% H_2_O_2_ for 20 min at room temperature and blocked by 2% BSA (w/w). Sections were then incubated with anti-MPO antibody (2 μg/mL, Proteintech, 22225-1-AP) at room temperature for 2 h. The sections were then washed with 0.05% TBST (0.05% Tween 20 in 1X PBS) and incubated with HRP-conjugated goat antirabbit antibodies (2 μg/mL, Abcam, ab205722) at room temperature for 1 h. The colored product of sections was developed by incubation with 3,30-diaminobenzidine tetrahydrochloride dihydrate substrate (DAB substrate, Abcam, 64238) and counter-stained with Hematoxylin Gill II reagent (Leica, 3801520). Slides were mounted with Surgipath Micromount mounting medium (Lieca, 3801731) and the immunohistochemical staining was photographed using an Axio Imager.A2 microscope (Zeiss).

### Detection of GHN105-mSTING Engagement in Mouse Colon Tissue

Frozen colon sections were ground to a fine powder using a pestle and mortar in the presence of liquid nitrogen. SDS lysis buffer was added to the samples, which were then sonicated at 50% amplitude for 20 min with 10 s intervals and 10 s pauses (Qsonica, Q700). Samples were centrifuged at 12,000 g for 10 min and soluble proteins in the supernatants were collected and protein concentrations were measured using BCA assay. For affinity purification and Western blot analysis of GHN105-engaged mSTING, 300–600 μg protein samples were reacted with azide-biotin (Vector Laboratories, CCT-1041) via CuAAC, followed by chloroform–methanol precipitation as described above. Air-dried protein pellets were resuspended in 20 μL SDS-HEPES buffer (4% SDS, 150 mM NaCl, 1 mM EDTA, 50 mM HEPES pH 7.4) and diluted to 0.25 mg/mL with HEPES buffer (150 mM NaCl, 50 mM HEPES pH 7.4). Protein samples were then added to 10 μL of prewashed streptavidin beads (Thermo Scientific, 20353), and incubated with end-overend rotation for 90 min at room temperature. The beads were sequentially washed once with 1 mL of 0.4% SDS in PBS followed by wash trice with 1 mL of chilled PBS. 25–50 μL of freshly prepared elution buffer (25 mM Na_2_S_2_O4 in PBS with 0.1% SDS) were added to the beads and the samples were eluted by 1 h incubation at room temperature for anti-STING Western blot analysis.

## Supporting Information Available

X-ray crystal analysis data, methods for additional compound synthesis, purification and characterization; ^1^H NMR, ^13^C NMR spectra, HPLC purity analysis, and mass spectra of the derivatives; the authors will release the atomic coordinates upon article publication (PDF)
hSTING-GHN105 docking study (PDB)
Molecular formula strings of the analogs (CSV)

## Supplementary Material

jm4c02665_si_001.pdf

jm4c02665_si_002.pdb

## Abbreviations

cGAS: cyclic GMP-AMP synthase
cGAMP: 2′,3′-cyclic guanosine monophosphate adenosine monophosphate
DSS: dextran sulfate sodium
ExcB: excavatolide B
IBD: inflammatory bowel disease
IFN: interferon
IRF3: IFN regulatory factor 3
PK: pharmacokinetics
SAVI: STING-associated vasculopathy in infants
STING: stimulator of interferon genes
TBK1: tank-binding kinase 1
