# Supplementary material for: Orally Bioavailable and Site-Selective Covalent STING Inhibitor Derived from a Macrocyclic Marine Diterpenoid
Source: J Med Chem. 2025 Feb 27;68(5):5471–87. doi: 10.1021/acs.jmedchem.4c02665 (PMC11912488; doi:10.1021/acs.jmedchem.4c02665)
Supplement: Supplementary file 1 — jm4c02665_si_001.pdf [file jm4c02665_si_001.pdf]

Supporting Information

Orally Bioavailable and Site-Selective Covalent STING Inhibitor Derived from a Macrocyclic Marine Diterpenoid

Guang-Hao Niu[1, #], Wan-Chi Hsiao[2, 4,#], Po-Hsun Lee[2, 4], Li-Guo Zheng[6], Yu-Shao Yang[3], Wei-Cheng Huang[1], Chih-Chien Hsieh[1], Tai-Yu Chiu[1], Jing-Ya Wang[1], Ching-Ping Chen[1], Chen-Lung Huang[1], May-Su You[2], Yi-Ping Kuo[3], Chien-Ming Wang[2], Zhi-Hong Wen[5], Guann-Yi Yu[3], Chiung-Tong Chen[1], Ya-Hui Chi[1], Chun-Wei Tung[1], Shu-Ching Hsu[3], Teng-Kuang Yeh[1], Ping-Jyun Sung[6, *], Mingzi M. Zhang[2, *], Lun Kelvin Tsou[1, *]

1. Institute of Biotechnology and Pharmaceutical Research, 2. Institute of Molecular and Genomic Medicine, 3. National Institute of Infectious Diseases and Vaccinology, National Health Research Institutes, Zhunan, Miaoli, 35053, Taiwan
4. Institute of Biotechnology, National Tsing Hua University, Hsinchu, 30013, Taiwan
5. Department of Marine Biotechnology and Resources, National Sun Yat-Sen University, Kaohsiung 804201, Taiwan
6. National Museum of Marine Biology and Aquarium, Pingtung 944401, Taiwan

[#]Equal contribution

Correspondence and material requests should be addressed to P.-J.S. (pjsung@nmmba.gov.tw), M.M.Z. (zhangmz@nhri.edu.tw), and L.K.T. (kelvintsou@nhri.edu.tw)

Table of Contents

Supporting Figures, Tables, and X-ray characterization of compounds …………………………………………………………………… S2-S22

Synthetic procedures ……,………………………………………S23-S46

Copies of ^{1}H, ^{13}C NMR, mass spectra, and HPLC traces ………………………S47-S119

Supplementary References……………………………………S120

Supplementary Figures

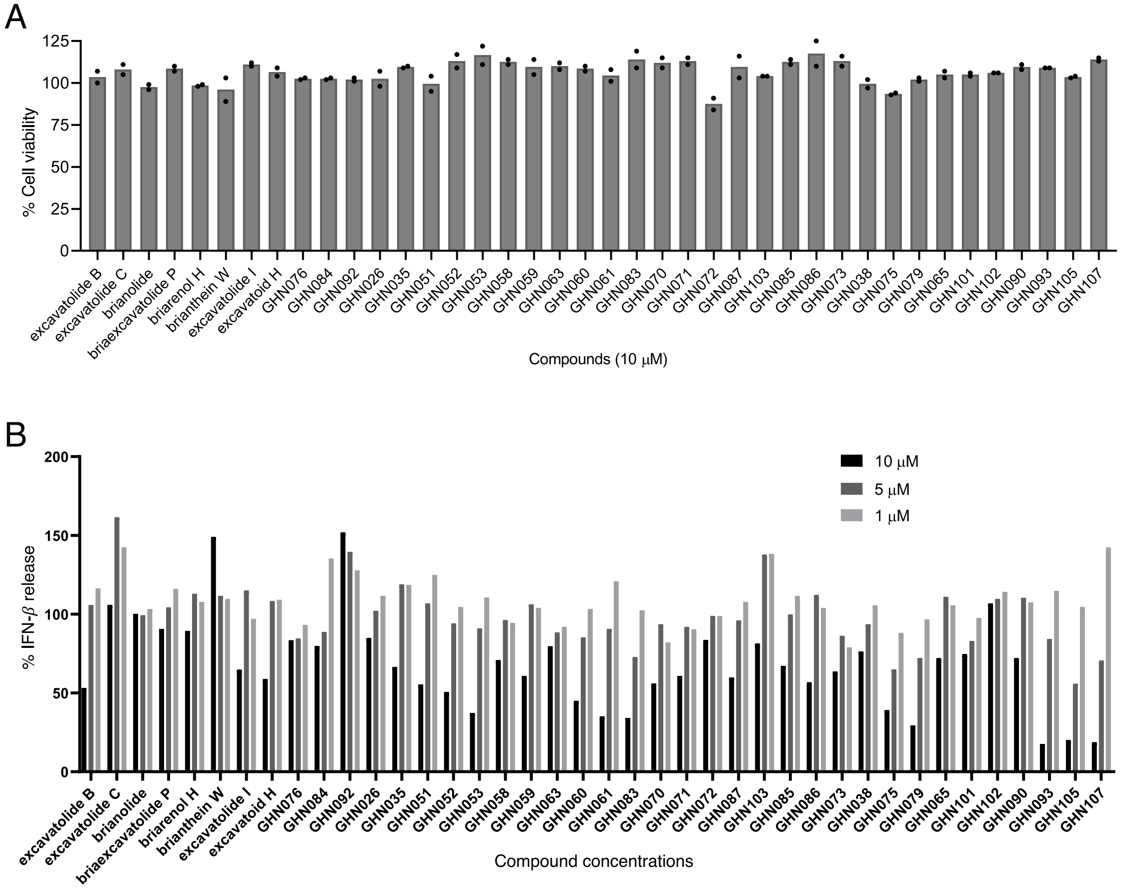

Figure S1. (A) Assessment of cell viability in THP-1 human macrophages exposed to ExcB, related briarane natural products, and new derivatives after 24 hours of incubation at a concentration of 10 μM. (B) ExcB analogs dose-dependently inhibited STING-dependent IFN-β release from THP1 cells. ELISA analysis of IFN-β released from THP1 macrophages pretreated with vehicle control (DMSO) or the indicated compounds at 10, 5, and 1 μM for 1 h, followed by activation with cGAMP treatment.

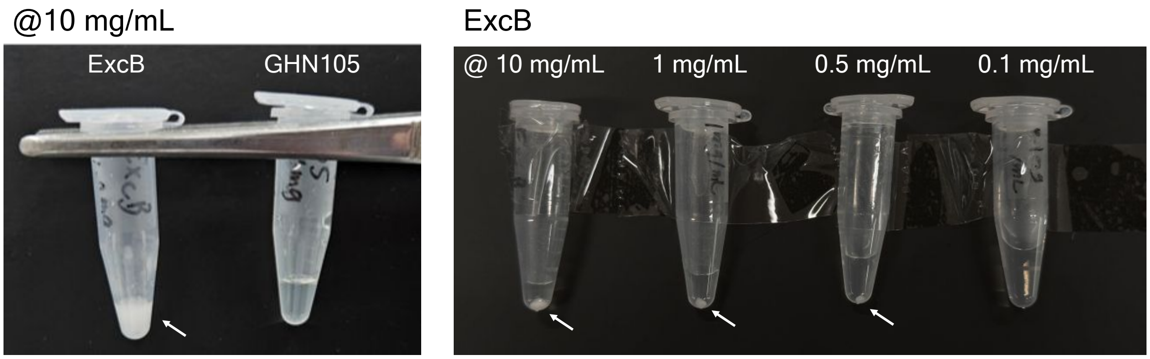

Figure S2. The solubility of the compounds ExcB and **GHN105** in aqueous media was evaluated. The representative photograph on the left demonstrated that GHN105 was fully dissolved at a concentration of 10 mg/mL. In contrast, the photograph on the right showed that a clear solution of ExcB was achieved only at a concentration of 0.1 mg/mL, with visible pellets of undissolved ExcB remaining in the other samples.

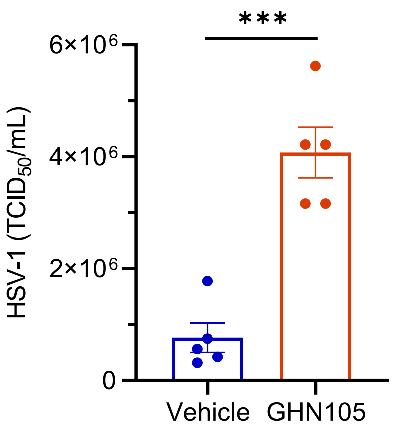

Figure S3. GHN105 suppressed the hSTING-mediated antiviral response. hMSCs were pretreated with either 1 μM of GHN105 or DMSO (vehicle) for 2 h. Following this, the cells were infected with HSV-1 (MOI=0.5) for 2 h. After the infection period, the medium was replaced with either drug- or vehicle-containing medium, and the cells were incubated for an additional 28 h. Viral titers were then determined using the $TCID_{50}$ assay. Error bars, s.e.m. n=5. Two-tailed t-test, ***$p < 0.001$.

A

	i.p., 15 mg/kg
$T_{1/2}$ (h)	1
T_{max} (h)	-
CL (mL/min/kg)	-
Vss (L/kg)	-
C_{max} (ng/mL)	784.7
C_{max} (μM)	0.98
AUC (min*ng/mL)	31680
AUC (mM-h)	0.66

B

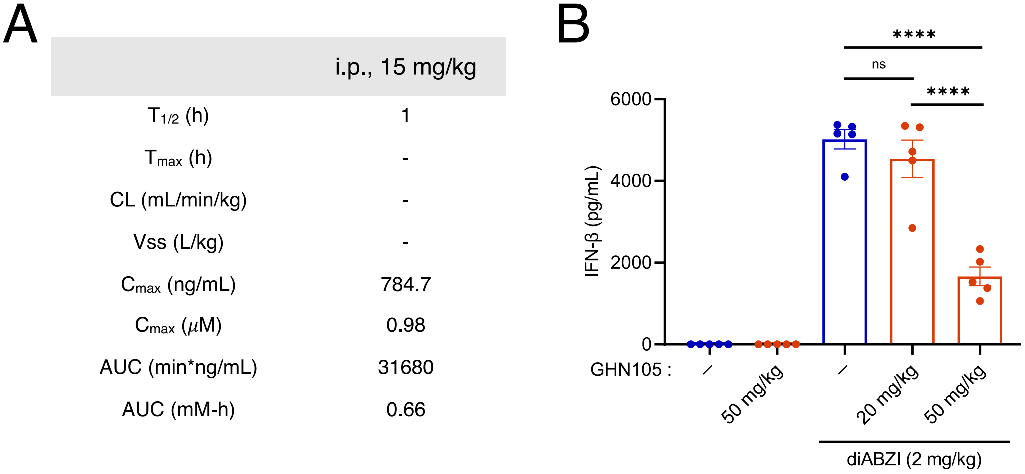

Figure S4. (A) The *in vivo* PK properties of GHN105 with 15 mg/kg intraperitoneal (i.p.) administration. (B) C57BL/6J mice were pretreated (i.p.) with 20 mg/kg or 50 mg/kg GHN105 for 1 h, followed by administration of diABZI (2 mg/kg, i.p.). Serum IFN-β levels were determined after 3.5 h by ELISA. N.D., not detected. Error bars, s.e.m. n=5. One-way ANOVA, ****$p < 0.0001$; ns, not significant.

Compounds	Survival rate	Coagulation of the embryo	Non-detachment of the tail	Lack of somite formation	Lack of heartbeat
Vehicle (DMSO)	100 % (16/16)	0% (0/16)	0% (0/16)	0% (0/16)	0% (0/16)
*Brianthein W	12.5 % (1/8)	0% (0/8)	87.5% (7/8)	0% (0/8)	12.5 % (1/8)
Excavatolide B	100 % (8/8)	0% (0/8)	0% (0/8)	0% (0/8)	0% (0/8)
GHN105	100 % (8/8)	0% (0/8)	0% (0/8)	0% (0/8)	0% (0/8)

*Positive control of a toxic briarane. Brianthein W was reported to cause developmental defects in zebrafish embryos.[1]

Figure S5. No toxic effects were observed for ExcB and GHN105 in zebrafish embryonic toxicity assay. For each group, 8 or16 embryos were exposed to DMSO vehicle or 40 μM of the indicated compounds, respectively. Lethality and phenotypic observations were recorded visually based on lethal endpoints (i.e. coagulation of the embryo, non-detachment of the tail, lack of somite formation, and lack of heartbeat) using a dissecting microscope at 72 hpf.

Supplementary Tables

X-ray Crystallographic Analysis of **ExcB-cysteine ethyl ester** (CCDC 2360376)

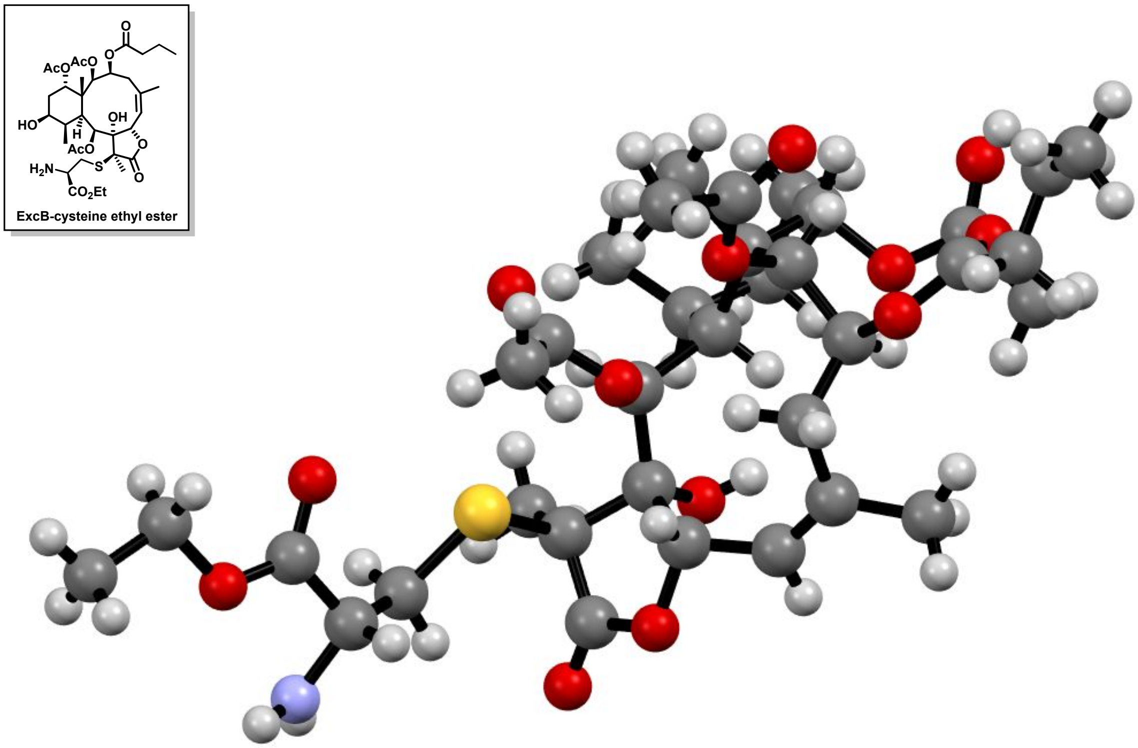

Table S1. Crystal data and structure refinement for **ExcB-cysteine ethyl ester**

Identification code	**ExcB-cysteine ethyl ester**	
Empirical formula	C36 H55 Cl2 N O14 S	
Formula weight	828.77	
Temperature	200(2) K	
Wavelength	0.71073 Å	
Crystal system	Monoclinic	
Space group	P 21	
Unit cell dimensions	a = 16.663(3) Å	α= 90°.
	b = 9.9575(19) Å	β= 107.440(5)°.
	c = 25.866(5) Å	γ = 90°.
Volume	4094.4(13) Å^3	

Z	4
Density (calculated)	1.344 Mg/m^3
Absorption coefficient	0.275 mm^{-1}
F(000)	1760
Crystal size	0.77 x 0.03 x 0.01 mm^3
Theta range for data collection	2.21 to 25.12°.
Index ranges	-19<=h<=19, -11<=k<=11, -30<=l<=30
Reflections collected	42947
Independent reflections	14422 [R(int) = 0.1801]
Completeness to theta = 25.12°	99.0 %
Absorption correction	multi-scan
Max. and min. transmission	0.9973 and 0.8164
Refinement method	Full-matrix least-squares on F^2
Data / restraints / parameters	14422 / 15 / 958
Goodness-of-fit on F^2	0.975
Final R indices [I>2sigma(I)]	R1 = 0.0958, wR2 = 0.2270
R indices (all data)	R1 = 0.2512, wR2 = 0.3210
Absolute structure parameter	-0.01(16)
Largest diff. peak and hole	0.522 and -0.475 e.$Å^{-3}$

X-ray Crystallographic Analysis of **GHN076** (CCDC 2250291)

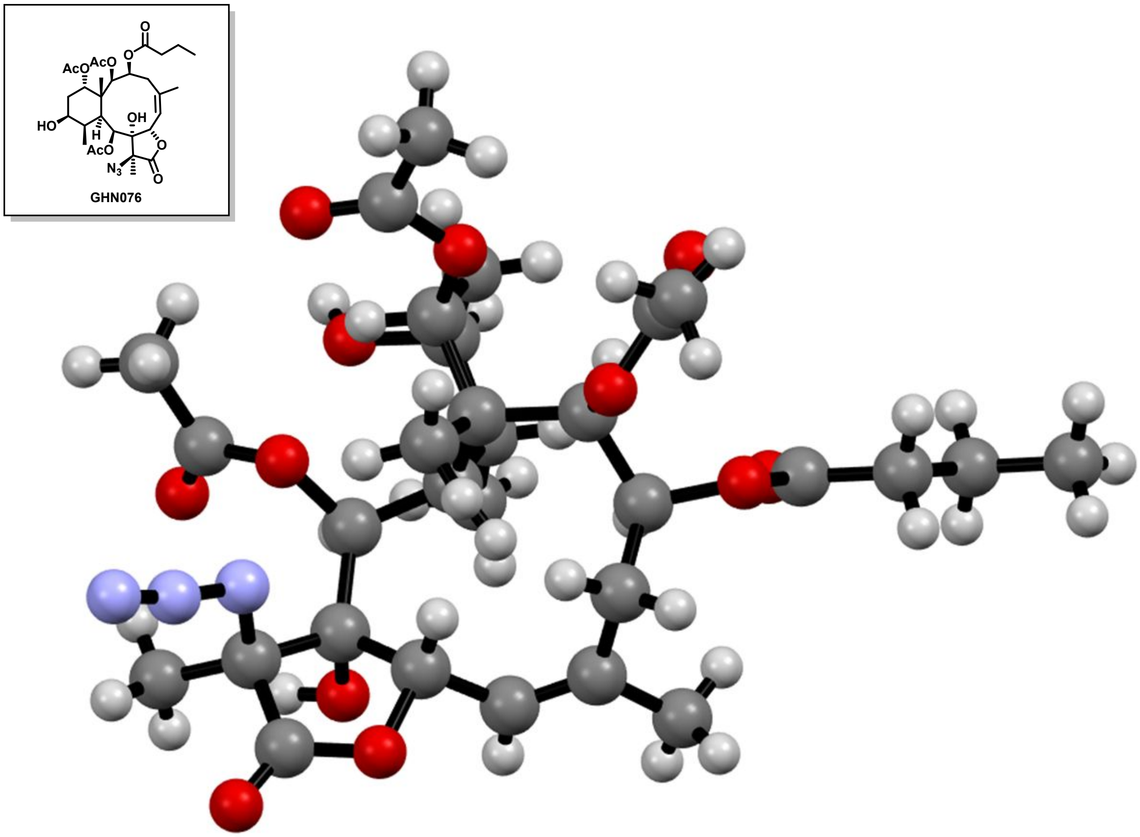

Table S2. Crystal data and structure refinement for **GHN076**.

Identification code	**GHN076**	
Empirical formula	C30 H43 N3 O12	
Formula weight	637.67	
Temperature	200(2) K	
Wavelength	0.71073 Å	
Crystal system	Orthorhombic	
Space group	P 21 21 21	
Unit cell dimensions	a = 9.0907(11) Å	α= 90°.
	b = 15.6084(15) Å	β= 90°.
	c = 22.069(2) Å	γ = 90°.
Volume	3131.4(6) $Å^3$	
Z	4	
Density (calculated)	1.353 Mg/m^3	
Absorption coefficient	0.105 mm^{-1}	
F(000)	1360	

Crystal size	0.44 x 0.20 x 0.02 mm^3
Theta range for data collection	2.26 to 25.10°.
Index ranges	-8<=h<=10, -16<=k<=18, -24<=l<=26
Reflections collected	15796
Independent reflections	5554 [R(int) = 0.0767]
Completeness to theta = 25.10°	99.8 %
Absorption correction	multi-scan
Max. and min. transmission	0.9979 and 0.9553
Refinement method	Full-matrix least-squares on F^2
Data / restraints / parameters	5554 / 0 / 414
Goodness-of-fit on F^2	1.019
Final R indices [I>2sigma(I)]	R1 = 0.0507, wR2 = 0.0978
R indices (all data)	R1 = 0.0867, wR2 = 0.1146
Absolute structure parameter	0.1(12)
Largest diff. peak and hole	0.207 and -0.273 e.$Å^{-3}$

X-ray Crystallographic Analysis of **GHN084** (CCDC 2250290)

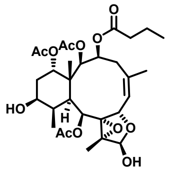

GHN084

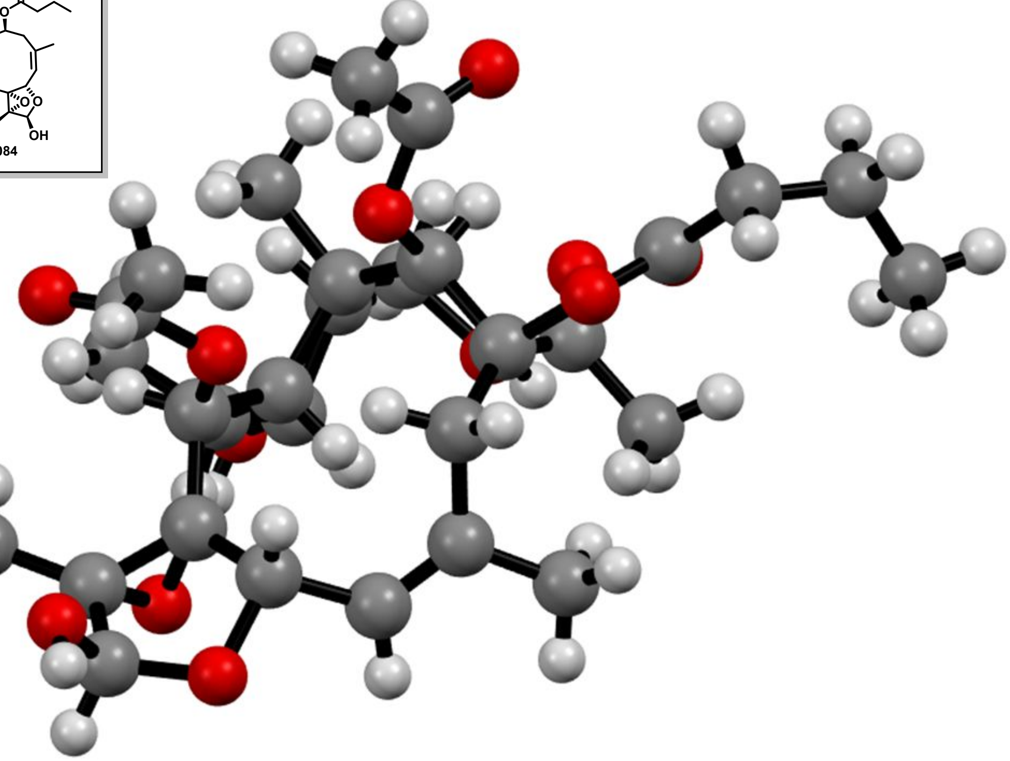

Table S3. Crystal data and structure refinement for **GHN084**.

Identification code	**GHN084**	
Empirical formula	C30 H44 O12	
Formula weight	596.65	
Temperature	200(2) K	
Wavelength	0.71073 Å	
Crystal system	Monoclinic	
Space group	P 21	
Unit cell dimensions	a = 10.4102(13) Å	α= 90°.
	b = 20.688(3) Å	β= 96.616(3)°.
	c = 14.889(2) Å	γ = 90°.
Volume	3185.0(7) Å^3	
Z	4	
Density (calculated)	1.244 Mg/m^3	
Absorption coefficient	0.096 mm^{-1}	
F(000)	1280	
Crystal size	0.08 x 0.05 x 0.02 mm^3	
Theta range for data collection	2.20 to 25.09°.	

Index ranges	-12<=h<=12, -24<=k<=24, -17<=l<=17
Reflections collected	37922
Independent reflections	11292 [R(int) = 0.1274]
Completeness to theta = 25.09°	99.6 %
Absorption correction	multi-scan
Max. and min. transmission	0.9981 and 0.9924
Refinement method	Full-matrix least-squares on F^2
Data / restraints / parameters	11292 / 17 / 759
Goodness-of-fit on F^2	0.982
Final R indices [I>2sigma(I)]	R1 = 0.0802, wR2 = 0.1870
R indices (all data)	R1 = 0.2098, wR2 = 0.2610
Absolute structure parameter	-0.7(17)
Largest diff. peak and hole	0.519 and -0.603 e.$Å^{-3}$

X-ray Crystallographic Analysis of **GHN038** (CCDC 2250289)

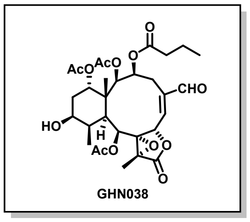

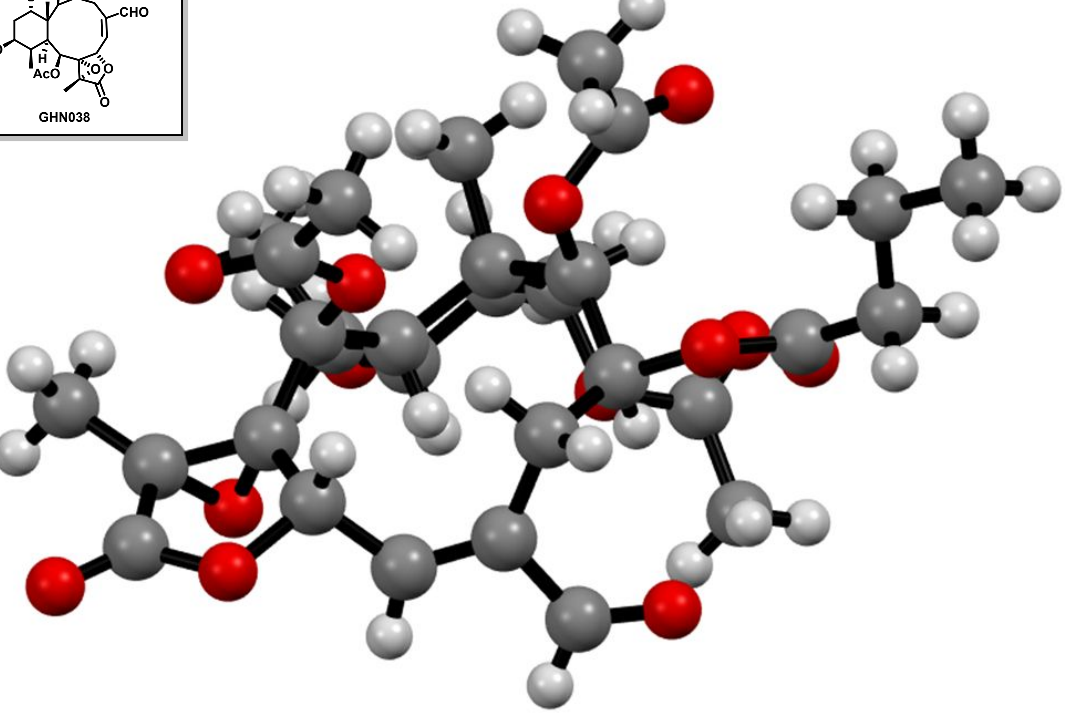

Table S4. Crystal data and structure refinement for **GHN038**.

Identification code	**GHN038**	
Empirical formula	C30 H40 O13	
Formula weight	608.62	
Temperature	200(2) K	
Wavelength	0.71073 Å	
Crystal system	Monoclinic	
Space group	P 21	
Unit cell dimensions	a = 11.1946(7) Å	α= 90°.
	b = 14.8564(8) Å	β= 91.243(2)°.
	c = 17.9993(12) Å	γ = 90°.
Volume	2992.8(3) Å^3	
Z	4	
Density (calculated)	1.351 Mg/m^3	
Absorption coefficient	0.106 mm^{-1}	
F(000)	1296	
Crystal size	0.68 x 0.43 x 0.24 mm^3	

Theta range for data collection	2.12 to 25.07°.
Index ranges	-13<=h<=13, -17<=k<=17, -21<=l<=21
Reflections collected	30124
Independent reflections	10564 [R(int) = 0.0735]
Completeness to theta = 25.07°	99.5 %
Absorption correction	multi-scan
Max. and min. transmission	0.9750 and 0.9315
Refinement method	Full-matrix least-squares on F^2
Data / restraints / parameters	10564 / 1 / 789
Goodness-of-fit on F^2	1.014
Final R indices [I>2sigma(I)]	R1 = 0.0489, wR2 = 0.1119
R indices (all data)	R1 = 0.0663, wR2 = 0.1218
Absolute structure parameter	0.2(7)
Largest diff. peak and hole	0.249 and -0.244 e.$Å^{-3}$

X-ray Crystallographic Analysis of **GHN075** (CCDC 2360374)

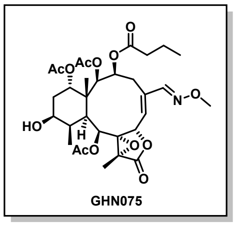

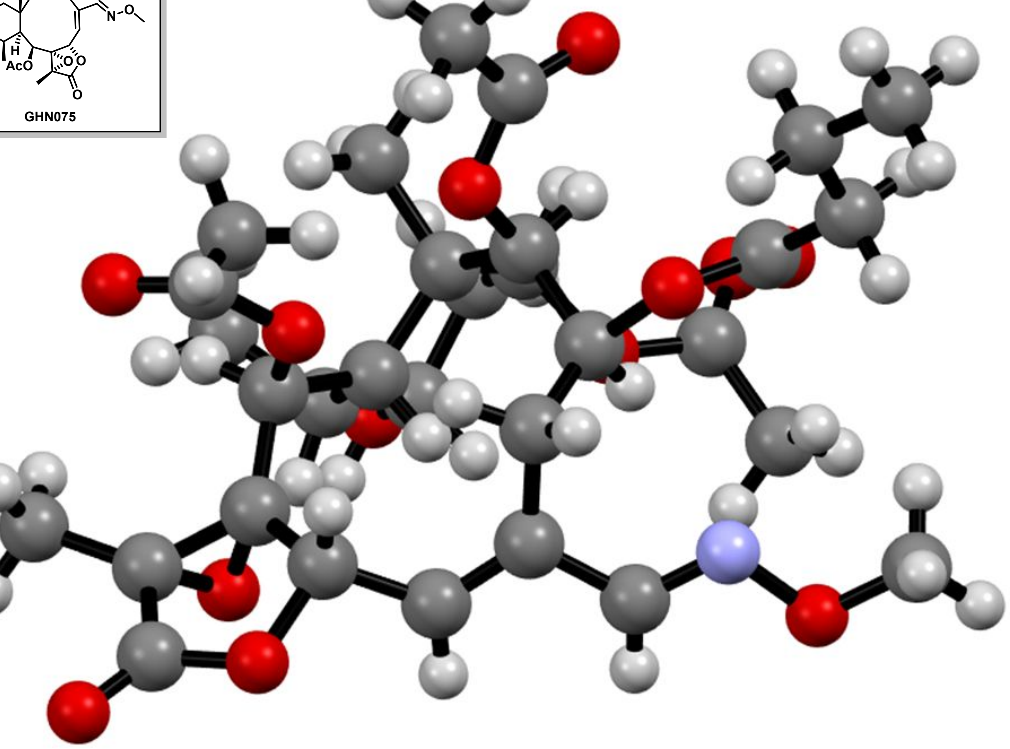

Table S5. Crystal data and structure refinement for **GHN075**.

Identification code	**GHN075**	
Empirical formula	C31 H43 N O13	
Formula weight	637.66	
Temperature	200(2) K	
Wavelength	0.71073 Å	
Crystal system	Monoclinic	
Space group	P 21	
Unit cell dimensions	a = 10.1302(3) Å	a= 90°.
	b = 30.2779(9) Å	b= 90.0510(10)°.
	c = 10.6210(3) Å	g = 90°.
Volume	3257.68(16) Å3	
Z	4	
Density (calculated)	1.300 Mg/m3	
Absorption coefficient	0.101 mm-1	
F(000)	1360	

Crystal size	0.23 x 0.15 x 0.11 mm3
Theta range for data collection	1.92 to 25.04°.
Index ranges	-12<=h<=12, -36<=k<=35, -12<=l<=12
Reflections collected	46678
Independent reflections	11302 [R(int) = 0.0672]
Completeness to theta = 25.04°	99.7 %
Absorption correction	None
Max. and min. transmission	0.9889 and 0.9771
Refinement method	Full-matrix least-squares on F2
Data / restraints / parameters	11302 / 1 / 828
Goodness-of-fit on F2	1.028
Final R indices [I>2sigma(I)]	R1 = 0.1019, wR2 = 0.2808
R indices (all data)	R1 = 0.1084, wR2 = 0.2858
Absolute structure parameter	-0.7(18)
Extinction coefficient	0.033(3)
Largest diff. peak and hole	0.960 and -0.542 e.Å-3

X-ray Crystallographic Analysis of **GHN079** (CCDC 2360375)

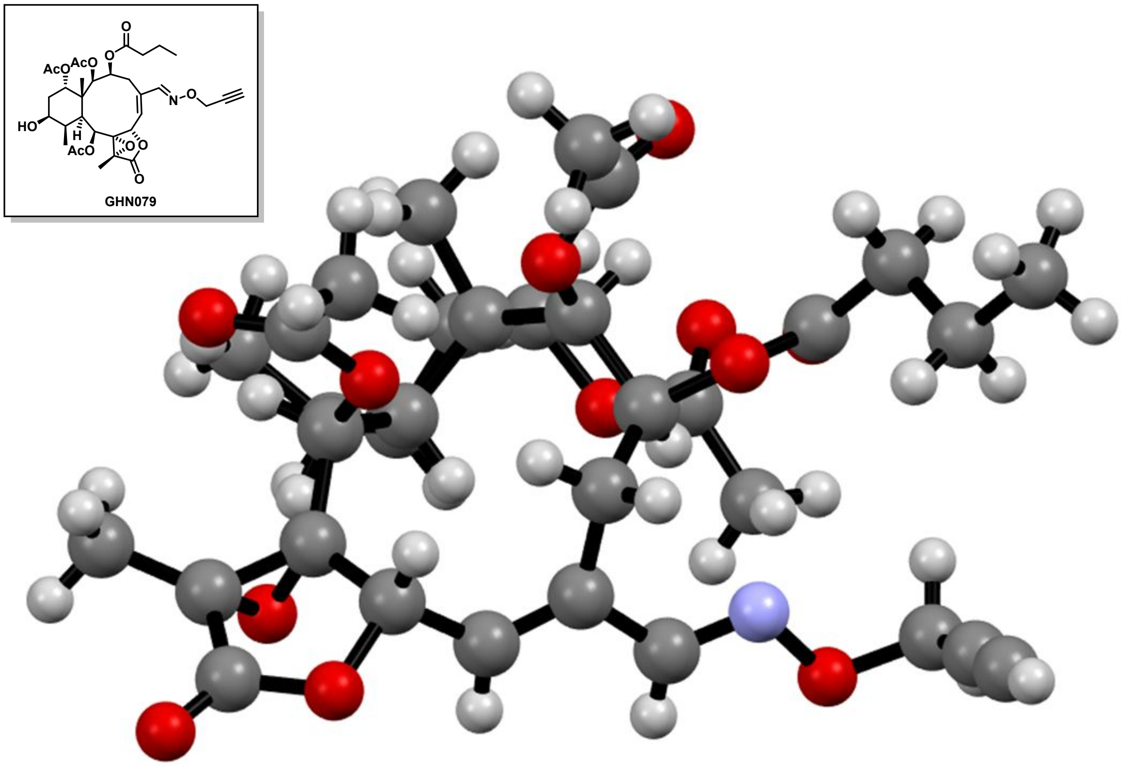

Table S6. Crystal data and structure refinement for **GHN079**.

Identification code	**GHN079**	
Empirical formula	C33 H43 N O13	
Formula weight	661.68	
Temperature	200(2) K	
Wavelength	0.71073 Å	
Crystal system	Orthorhombic	
Space group	P 21 21 21	
Unit cell dimensions	a = 10.2547(5) Å	a= 90°.
	b = 10.5261(4) Å	b= 90°.
	c = 31.0412(14) Å	g = 90°.
Volume	3350.6(3) Å3	
Z	4	
Density (calculated)	1.312 Mg/m3	
Absorption coefficient	0.101 mm-1	
F(000)	1408	
Crystal size	0.22 x 0.13 x 0.10 mm3	

Theta range for data collection	2.09 to 25.05°.
Index ranges	-12<=h<=10, -12<=k<=12, -36<=l<=36
Reflections collected	42685
Independent reflections	5922 [R(int) = 0.0841]
Completeness to theta = 25.05°	99.7 %
Absorption correction	None
Max. and min. transmission	0.9899 and 0.9781
Refinement method	Full-matrix least-squares on F2
Data / restraints / parameters	5922 / 0 / 429
Goodness-of-fit on F2	1.067
Final R indices [I>2sigma(I)]	R1 = 0.0579, wR2 = 0.1396
R indices (all data)	R1 = 0.0692, wR2 = 0.1481
Absolute structure parameter	-0.2(13)
Largest diff. peak and hole	0.897 and -0.215 e.Å-3

X-ray Crystallographic Analysis of **GHN092** (CCDC 2381440)

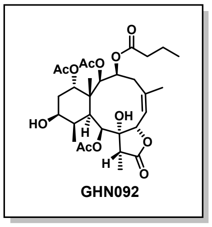

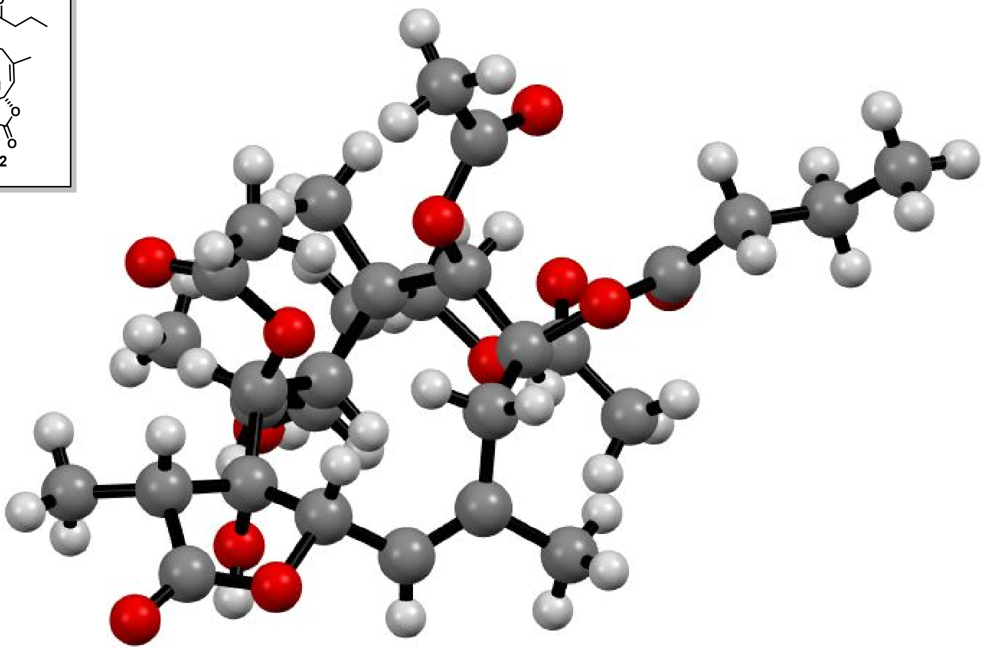

Table S7. Crystal data and structure refinement for **GHN092**.

Identification code	**GHN092**	
Empirical formula	C61 H90 Cl2 O24	
Formula weight	1278.23	
Temperature	200(2) K	
Wavelength	0.71073 Å	
Crystal system	Monoclinic	
Space group	P 21	
Unit cell dimensions	a = 10.2799(12) Å	a= 90°.
	b = 34.639(4) Å	b= 119.636(4)°.
	c = 10.3538(16) Å	g = 90°.
Volume	3204.5(7) Å3	
Z	2	
Density (calculated)	1.325 Mg/m3	
Absorption coefficient	0.181 mm-1	
F(000)	1364	
Crystal size	0.16 x 0.04 x 0.01 mm3	
Theta range for data collection	2.26 to 25.16°.	
Index ranges	-12<=h<=12, -41<=k<=41, -12<=l<=12	
Reflections collected	27096	
Independent reflections	11435 [R(int) = 0.0984]	

Completeness to theta = 25.16°	99.5 %
Absorption correction	None
Max. and min. transmission	0.9982 and 0.9717
Refinement method	Full-matrix least-squares on F2
Data / restraints / parameters	11435 / 6 / 789
Goodness-of-fit on F2	1.209
Final R indices [I>2sigma(I)]	R1 = 0.1420, wR2 = 0.3560
R indices (all data)	R1 = 0.1893, wR2 = 0.3889
Absolute structure parameter	0.4(3)
Largest diff. peak and hole	0.593 and -0.795 e.Å-3

X-ray Crystallographic Analysis of **GHN105** (CCDC 2381441)

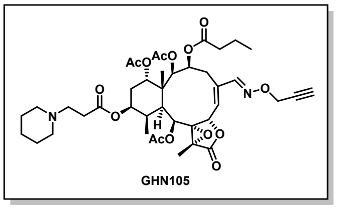

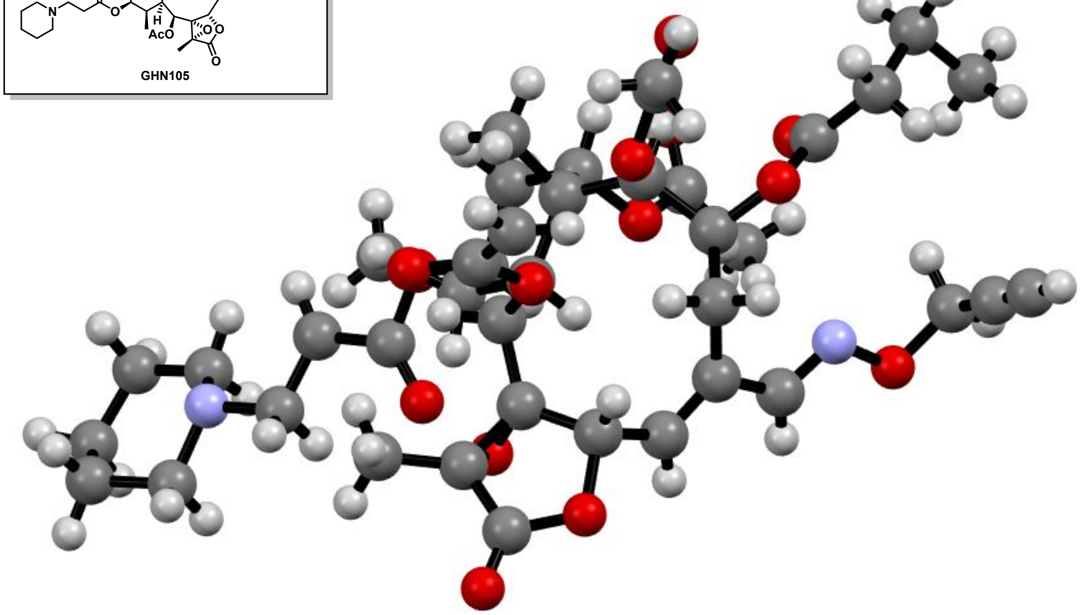

Table S8. Crystal data and structure refinement for **GHN105**.

Identification code	**GHN105**	
Empirical formula	C87 H120 Cl2 N4 O30	
Formula weight	1772.77	
Temperature	200(2) K	
Wavelength	0.71073 Å	
Crystal system	Triclinic	
Space group	P 1	
Unit cell dimensions	a = 9.1810(5) Å	a= 84.410(2)°.
	b = 13.3326(6) Å	b= 82.991(2)°.
	c = 20.8460(9) Å	g = 74.692(2)°.
Volume	2437.1(2) Å3	
Z	1	
Density (calculated)	1.208 Mg/m3	
Absorption coefficient	0.143 mm-1	
F(000)	944	
Crystal size	0.32 x 0.07 x 0.03 mm3	
Theta range for data collection	2.31 to 25.15°.	
Index ranges	-10<=h<=10, -15<=k<=15, -24<=l<=24	
Reflections collected	92458	

Independent reflections	17295 [R(int) = 0.0847]
Completeness to theta = 25.15°	99.5 %
Absorption correction	None
Max. and min. transmission	0.9957 and 0.9557
Refinement method	Full-matrix least-squares on F2
Data / restraints / parameters	17295 / 9 / 1075
Goodness-of-fit on F2	1.081
Final R indices [I>2sigma(I)]	R1 = 0.0755, wR2 = 0.2041
R indices (all data)	R1 = 0.0887, wR2 = 0.2194
Absolute structure parameter	0.1(2)
Largest diff. peak and hole	0.701 and -1.021 e.Å-3

Synthesis and characterization of ExcB derivatives.

Synthesis of **GHN026**.

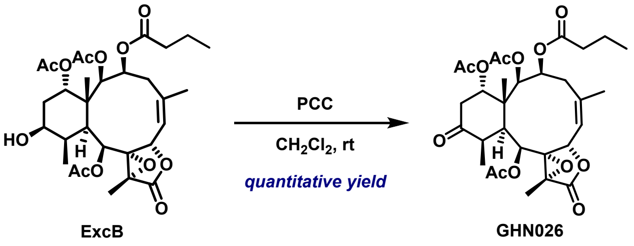

Under ambient atmosphere, a 20 mL vial with a stir bar was charged with **ExcB** (182.7 mg, 0.307 mmol, 1.00 equiv.) and dried CH_2Cl_2 (2 mL). PCC (132.4 mg, 0.614 mmol, 2.00 equiv.) was added, and the reaction mixture was stirred at room temperature for 3 h. The reaction mixture was filtered through a short pad of Celite®, rinsed with CH_2Cl_2. The filtrate was collected and concentrated by rotary evaporation. The residue was purified by flash column chromatography on silica gel (EtOAc : *n*-Hexane = 0 ∶ 1→1 ∶ 1) to afford **GHN026** (182.0 mg, quantitative yield) as a white solid.

TLC: $R_f = 0.33$ (EtOAc : *n*-Hexane = 1 ∶ 1, $KMnO_4$). ^{1}H NMR (600 MHz, $CDCl_3$, 22 ^{o}C) δ 5.78 (br s, 1H), 5.35 (s, 2H), 5.28 (br s, 1H), 5.25 (d, *J* = 9.3 Hz, 1H), 5.17 (dd, *J* = 9.8, 4.0 Hz, 1H), 3.61 (dd, *J* = 15.7, 4.9 Hz, 1H), 3.25 (dd, *J* = 9.3, 5.7 Hz, 1H), 3.01 – 2.93 (m, 1H), 2.90 – 2.82 (m, 1H), 2.78 (dd, *J* = 16.5, 4.0 Hz, 1H), 2.31 (s, 3H), 2.24 (t, *J* = 7.4 Hz, 2H), 2.18 (s, 3H), 2.11 (s, 3H), 2.10 – 2.06 (m, 1H), 1.81 (s, 3H), 1.66 – 1.60 (m, 2H), 1.59 (s, 3H), 1.23 (d, *J* = 7.5 Hz, 3H), 1.14 (s, 3H), 0.94 (t, *J* = 7.4 Hz, 3H).

^{13}C NMR (151 MHz, $CDCl_3$, 22 ^{o}C) δ 207.9, 172.3, 171.1, 170.1, 169.7, 168., 140.1, 122.3, 78.1 (br), 74.0, 73.6 (br), 69.3, 66.8, 60.5, 43.7, 40.3, 39.7 (br), 35.9, 33.7, 22.1, 21.6, 21.2, 18.2, 18.1, 14.1 (br), 13.6, 10.1.

HRMS-ESI (m/z) calcd for $C_{30}H_{40}O_{12}Na$ $[M+Na]^+$, 615.2412; found, 615.2416.

HPLC purity: t_R = 17.0 min, 98.9% (λ = 210 nm).

Synthesis of **GHN035**.

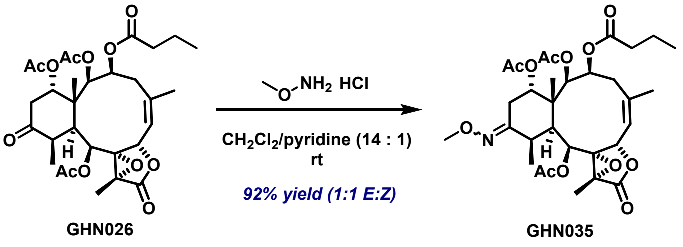

Under an ambient atmosphere, a 4 mL vial equipped with a stir bar was charged with **GHN026** (21.6 mg, 0.0364 mmol, 1.0 equiv.), CH_2Cl_2/pyridine (14 : 1, 1.0 mL) and *O*-methylhydroxylamine hydrochloride (6.1 mg, 0.0729 mmol, 2.0 equiv.). The vial was sealed with a Teflon cap, and the resulting solution was stirred at room temperature. After 18 h, the reaction mixture was directly purified by flash column chromatography (EtOAc ∶ *n*-Hexane = 0 ∶ 1→2 ∶ 3) gave **GHN035** (20.8 mg, 92% yield, *E* ∶ *Z* = 1 ∶ 1) as a white solid.

TLC: R_f = 0.28 (EtOAc : *n*-Hexane = 2 ∶ 3, $KMnO_4$).

^{1}H NMR (600 MHz, $CDCl_3$, 22 °C) δ 5.84 (br s, 1H), 5.69 (br s, 1H), 5.45 – 5.32 (m, 4H), 5.30 (d, *J* = 7.0 Hz, 1H), 5.21 (d, *J* = 10.7 Hz, 2H), 5.15 (d, *J* = 10.3 Hz, 1H), 5.08 (br s, 1H), 5.05 (dd, *J* = 9.6, 3.2 Hz, 1H), 4.02 – 3.93 (m, 1H), 3.83 (s, 3H), 3.81 (s, 3H), 3.77 – 3.69 (m, 1H), 3.65 (dd, *J* = 15.6, 5.2 Hz, 1H), 3.55 (dd, *J* = 15.7, 4.8 Hz, 1H), 3.13 (dd, *J* = 18.6, 11.8 Hz, 1H), 3.09 – 3.00 (m, 2H), 2.96 (dd, *J* = 10.7, 5.6 Hz, 1H), 2.80 (dd, *J* = 16.2, 9.2 Hz, 2H), 2.60 (dd, *J* = 16.4, 3.2 Hz, 1H), 2.33 (s, 3H), 2.28 (s, 3H), 2.26 (t, *J* = 7.4 Hz, 2H), 2.23 (t, *J* = 7.4 Hz, 2H), 2.17 (s, 6H), 2.13 (s, 3H), 2.09 (s, 3H), 2.09 – 2.00 (m, 2H), 1.83 (s, 3H), 1.77 (s, 3H), 1.68 –1.57 (m, 4H), 1.64 (s, 3H), 1.58 (s, 3H), 1.24 (s,

2H), 1.22 (d, J = 7.2 Hz, 3H), 1.17 (d, J = 7.3 Hz, 3H), 0.97 – 0.85 (m, 12H).

^{13}C NMR (151 MHz, $CDCl_3$, 22 °C) δ 172.2, 171.9, 171.4, 171.3, 170.4, 170.1, 170.0, 169.9, 169.0, 168.7, 157.0, 139.9, 139.6, 122.7, 122.1, 79.9 (br), 78.6 (br), 73.9, 73.8, 69.3, 68.9, 67.5, 66.6, 61.7, 61.4, 60.3, 59.9, 44.3, 43.9, 40.1, 38.9, 38.7, 36.6, 36.0, 36.0, 34.4, 34.3, 33.9, 33.8, 31.9, 30.4, 30.0, 29.7, 29.3, 29.1, 28.9, 28.4, 26.3 (br), 25.0, 23.7, 22.9, 22.7, 21.9, 21.9, 21.8, 21.3, 21.3, 21.2, 21.0, 20.3, 18.3, 18.2, 18.2, 17.7, 15.8, 14.4, 14.1, 14.0, 13.6, 13.5, 11.0, 10.2, 10.1.

HRMS-ESI (m/z) calcd for $C_{31}H_{43}NO_{12}Na$ $[M+Na]^+$ 644.2678, found 644.2669.

HPLC purity: t_R = 18.1 min, 49.6%; t_R = 18.9 min, 50.4% (λ = 210 nm).

Synthesis of **GHN051**.

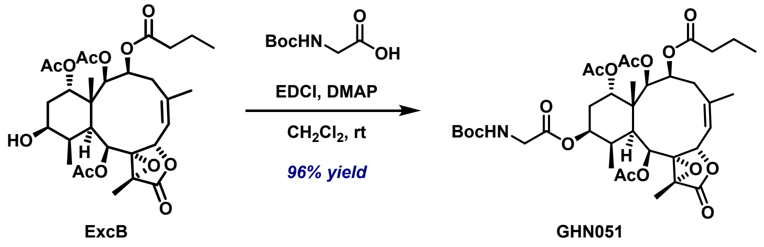

Under an ambient atmosphere, a 4 mL vial equipped with a stir bar was charged with **ExcB** (25.0 mg, 0.0420 mmol, 1.0 equiv.), Boc-Gly-OH (14.7 mg, 0.0840 mmol, 2.0 equiv.), DMAP (1.6 mg, 0.013 mmol, 0.3 equiv.), EDCI (24.2 mg, 0.126 mmol, 3.0 equiv.) and dried CH_2Cl_2 (0.5 mL). The vial was sealed with a Teflon cap, and the resulting solution was stirred at room temperature. After 18 h, the reaction mixture was diluted with CH_2Cl_2 and the organic layer was washed with H_2O. The organic layer was dried over $MgSO_4$, filtered, and concentrated by rotary evaporation. The residue was purified by flash column chromatography (EtOAc : n-Hexane = 0 : 1→1 : 1) to afford **GHN051** (30.4 mg, 96%

yield) as a white foam.

TLC: R_f = 0.43 (EtOAc : *n*-Hexane = 1 ∶ 1, $KMnO_4$).

^{1}H NMR (600 MHz, $(CD_3)_2CO$, -40 °C) δ 6.75 (X part of ABX system, J = 6.3 Hz, 1H), 5.85 (dd, J = 6.9, 1.8 Hz, 1H), 5.67 (d, J = 7.3 Hz, 1H), 5.53 (d, J = 10.4 Hz, 1H), 5.36 (dt, J = 7.4, 1.9 Hz, 1H), 5.16 (d, J = 2.4 Hz, 1H), 5.01 (ddd, J = 12.5, 5.0, 3.6 Hz, 1H), 4.74 – 4.66 (m, 1H), 4.01 (dd, J = 15.8, 7.3 Hz, 1H), 3.81, 3.78 (AB part of ABX system, J_{AB} = 18.0 Hz, J_{AX} = 6.6 Hz, J_{BX} = 6.6 Hz, 2H), 3.17 (dd, J = 10.4, 5.1 Hz, 1H), 2.68 – 2.60 (m, 1H), 2.41 (s, 3H), 2.23 (s, 6H), 2.22 – 2.06 (m, 4H), 1.95 (s, 3H), 1.90 – 1.85 (m, 1H), 1.55 – 1.46 (m, 2H), 1.50 (s, 3H), 1.37 (s, 9H), 1.10 (d, J = 7.1 Hz, 3H), 0.86 (s, 3H), 0.85 (t, J = 7.4 Hz, 3H).

^{13}C NMR (151 MHz, $(CD_3)_2CO$, -40 °C) δ 172.3, 172.1, 171.9, 170.7, 170.4, 170.1, 156.6, 140.0, 122.6, 81.4 (2xCH), 79.0, 74.2, 73.6, 70.8, 69.2, 65.2, 60.5, 44.2, 42.5, 40.3, 35.9, 34.3, 33.0, 28.1, 27.1, 22.6, 22.2, 22.1, 21.4, 18.4, 18.0, 13.6, 10.2, 10.0.

HRMS-ESI (m/z) calcd for $C_{37}H_{53}NO_{15}Na$ $[M+Na]^+$ 774.3307, found 774.3311.

HPLC purity: t_R = 19.4 min, 98.6% (λ = 210 nm).

Synthesis of **GHN052**.

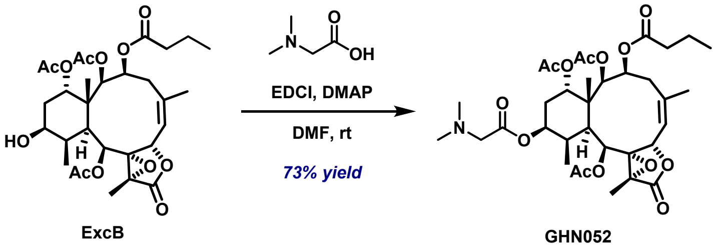

Under an ambient atmosphere, a 4 mL vial equipped with a stir bar was charged with **ExcB** (25.0 mg, 0.0420 mmol, 1.0 equiv.), dimethylglycine (8.7 mg, 0.0844 mmol, 2.0 equiv.),

DMAP (1.6 mg, 0.013 mmol, 0.3 equiv.), EDCI (24.2 mg, 0.126 mmol, 3.0 equiv.) and dried DMF (0.6 mL). The vial was sealed with a Teflon cap, and the resulting solution was stirred at room temperature. After 18 h, the reaction mixture was diluted with EtOAc and the organic layer was washed with H_2O. The organic layer was dried over $MgSO_4$, filtered, and concentrated by rotary evaporation. The residue was purified by flash column chromatography (EtOAc ∶ *n*-Hexane = 1 ∶ 1 → MeOH ∶ CH_2Cl_2 = 1 ∶ 10) to afford **GHN052** (20.7 mg, 73% yield) as a white foam.

TLC: R_f = 0.10 (EtOAc : *n*-Hexane = 1 ∶ 1, $KMnO_4$).

^{1}H NMR (600 MHz, $(CD_3)_2CO$, -40 °C) δ 5.85 (dd, *J* = 6.9, 1.7 Hz, 1H), 5.67 (d, *J* = 7.3 Hz, 1H), 5.52 (d, *J* = 10.4 Hz, 1H), 5.40 – 5.33 (m, 1H), 5.16 (d, *J* = 2.4 Hz, 1H), 5.03 (ddd, *J* = 12.4, 5.0, 3.6 Hz, 1H), 4.74 – 4.67 (m, 1H), 4.01 (dd, *J* = 15.8, 7.3 Hz, 1H), 3.27, 3.24 (ABq, *J* = 16.9 Hz, 2H), 3.16 (dd, *J* = 10.4, 5.1 Hz, 1H), 2.67 – 2.60 (m, 1H), 2.41 (s, 3H), 2.30 (s, 6H), 2.23 (s, 3H), 2.23 (s, 3H), 2.22 – 2.07 (m, 4H), 1.95 (s, 3H), 1.91 – 1.85 (m, 1H), 1.55 – 1.46 (m, 2H), 1.50 (s, 3H), 1.09 (d, *J* = 7.1 Hz, 3H), 0.86 (s, 3H), 0.85 (t, *J* = 7.4 Hz, 3H).

^{13}C NMR (151 MHz, $(CD_3)_2CO$, -40 °C) δ 172.2, 172.1, 171.9, 170.7, 170.1, 169.7, 140.0, 122.6, 81.4, 74.2, 73.6, 70.3, 69.2, 65.2, 60.6, 59.4, 44.5, 44.2, 40.3, 35.9, 34.3, 33.0, 27.2, 22.6, 22.2, 21.4, 18.4, 18.0, 13.7, 10.3, 10.0.

HRMS-ESI (m/z) calcd for $C_{34}H_{50}NO_{13}$ $[M+H]^+$ 680.3277, found 680.3279.

HPLC purity: t_R = 11.9 min, 96.4% (λ = 210 nm).

Synthesis of **GHN053**.

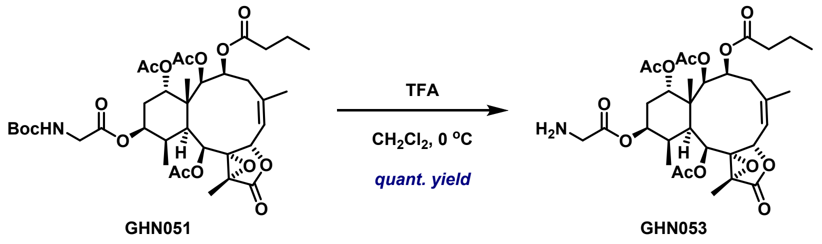

Under ambient atmosphere, a 4 mL vial equipped with a stir bar was charged with **GHN051** (41.9 mg, 0.0547 mmol, 1.0 equiv.), TFA (0.4 mL, d = 1.489 g/mL, 0.005 mmol, 0.1 equiv.) and dried CH_2Cl_2 (2.0 mL). The vial was sealed with a Teflon cap, and the resulting solution was stirred at 0 °C. After 1 h, the reaction mixture was diluted with CH_2Cl_2 and was added saturated $NaHCO_{3(aq.)}$(pH = 10). After the separation, the aqueous layer was extracted with CH_2Cl_2 for 2 times. The combined organic layers were dried over $MgSO_4$, filtered, and concentrated by rotary evaporation. The residue was purified by flash column chromatography (EtOAc : *n*-Hexane = 0 : 1→1 : 1→MeOH : CH_2Cl_2 (with 10% NH_4OH) = 1 : 15→1 : 10) to afford **GHN053** (36.8 mg, quantitative yield) as a white foam.

TLC: R_f = 0.23 (MeOH : CH_2Cl_2 = 1 : 10, $KMnO_4$).

^{1}H NMR (600 MHz, $(CD_3)_2CO$, -40 °C) δ 5.88 – 5.80 (m, 1H), 5.67 (d, *J* = 7.3 Hz, 1H), 5.57 – 5.50 (m, 1H), 5.39 – 5.33 (m, 1H), 5.19 – 5.13 (m, 1H), 5.03 (dt, *J* = 12.5, 4.3 Hz, 1H), 4.74 – 4.68 (m, 1H), 4.68 – 4.65 (m, 1H), 4.14 – 3.96 (m, 3H), 3.94 – 3.88 (m, 1H), 3.17 (dd, *J* = 10.3, 5.1 Hz, 1H), 3.08 (dd, *J* = 10.4, 5.1 Hz, 1H), 2.71 – 2.59 (m, 1H), 2.54 – 2.46 (m, 1H), 2.41 (s, 3H), 2.39 (s, 3H), 2.23 (s, 6H), 2.22 (s, 3H), 2.19 (s, 3H), 2.18 – 2.05 (m, 4H), 1.95 (d, *J* = 5.0 Hz, 3H), 1.92 – 1.82 (m, 1H), 1.57 – 1.45 (m, 5H), 1.10 (d, *J* = 7.0 Hz, 3H), 1.02 (d, *J* = 7.1 Hz, 3H), 0.89 – 0.80 (m, 6H).

^{13}C NMR (151 MHz, $(CD_3)_2CO$, -40 °C) δ 172.4, 172.3, 172.1, 171.9, 170.7, 170.2, 170.1,

140.0, 122.6, 82.0, 81.5, 81.4, 74.2, 73.6, 70.5, 69.3, 69.2, 65.9, 65.6, 65.2, 60.5, 60.4, 53.1, 44.2, 44.1, 40.3, 40.2, 39.1, 35.9, 34.4, 34.3, 33.0, 27.1, 25.5, 23.5, 23.3, 22.7, 22.6, 22.2, 22.2, 21.4, 21.4, 18.4, 18.3, 18.0, 14.4, 13.6, 11.1, 10.2, 10.0, 9.3.

Note: Two major conformers were observed at 233K in $(CD_3)_2CO$ in a 75/25 ratio.

HRMS-ESI (m/z) calcd for $C_{32}H_{46}NO_{13}$ $[M+H]^+$ 652.2964, found 652.2966.

HPLC purity: t_R = 11.1 min, 96.4% (λ = 210 nm).

Synthesis of **GHN058**.

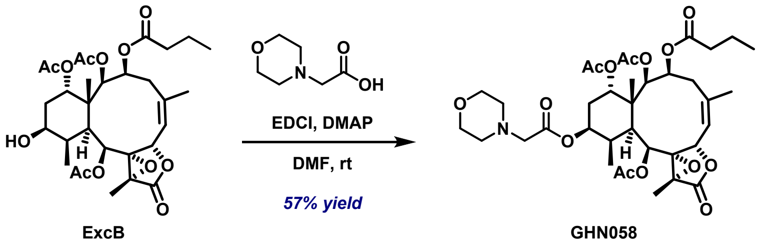

Under an ambient atmosphere, a 4 mL vial equipped with a stir bar was charged with **ExcB** (14.4 mg, 0.0242 mmol, 1.0 equiv.), 2-morpholinoacetic acid (7.0 mg, 0.0484 mmol, 2.0 equiv.), DMAP (0.9 mg, 7.4 µmol, 0.3 equiv.), EDCI (13.9 mg, 0.0726 mmol, 3.0 equiv.) and dried DMF (0.4 mL). The vial was sealed with a Teflon cap, and the resulting solution was stirred at room temperature. After 18 h, the reaction mixture was diluted with EtOAc and the organic layer was washed with H_2O. The organic layer was dried over $MgSO_4$, filtered, and concentrated by rotary evaporation. The residue was purified by flash column chromatography (EtOAc ∶ *n*-Hexane = 1 ∶ 1 → MeOH ∶ CH_2Cl_2 = 1 ∶ 10) to afford **GHN058** (9.9 mg, 57% yield) as a yellowish foam.

TLC: R_f = 0.10 (EtOAc : *n*-Hexane = 1 ∶ 1, $KMnO_4$).

^{1}H NMR (600 MHz, $(CD_3)_2CO$, -40 °C) δ 5.88 – 5.82 (m, 1H), 5.67 (d, *J* = 7.3 Hz, 1H),

5.52 (d, J = 10.4 Hz, 1H), 5.37 (dt, J = 7.4, 1.8 Hz, 1H), 5.16 (d, J = 2.4 Hz, 1H), 5.02 (ddd, J = 12.5, 5.0, 3.6 Hz, 1H), 4.70 (dd, J = 4.1, 2.1 Hz, 1H), 4.01 (dd, J = 15.8, 7.3 Hz, 1H), 3.79 – 3.40 (m, 4H), 3.24, 3.21 (ABq, J = 17.0 Hz, 2H), 3.16 (dd, J = 10.4, 5.2 Hz, 1H), 2.79 – 2.59 (m, 3H), 2.41 (s, 3H), 2.38 – 2.26 (m, 2H), 2.23 (s, 3H), 2.23 (s, 3H), 2.21 – 2.06 (m, 4H), 1.95 (s, 3H), 1.90 – 1.85 (m, 1H), 1.56 – 1.46 (m, 2H), 1.51 (s, 3H), 1.09 (d, J = 7.2 Hz, 3H), 0.86 (s, 3H), 0.85 (t, J = 7.4 Hz, 3H).

^{13}C NMR (151 MHz, $(CD_3)_2CO$, -40 ^{o}C) δ 172.2, 172.1, 171.9, 170.7, 170.1, 169.8, 140.0, 122.6, 81.4, 74.2, 73.6, 70.3, 69.2, 67.0, 65.2, 60.6, 59.1, 53.2, 44.2, 40.3, 35.9, 34.3, 33.0, 27.2, 22.6, 22.2, 21.4, 18.4, 18.0, 13.7, 10.3, 10.0.

HRMS-ESI (m/z) calcd for $C_{36}H_{52}NO_{14}$ $[M+H]^+$ 722.3382, found 722.3388.

HPLC purity: t_R = 12.3 min, 100.0% (λ = 210 nm).

Synthesis of **GHN059**.

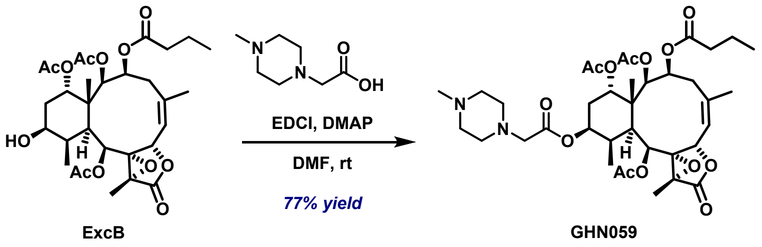

Under an ambient atmosphere, a 4 mL vial equipped with a stir bar was charged with **ExcB** (16.5 mg, 0.0277 mmol, 1.0 equiv.), 2-(4-methylpiperazin-1-yl)acetic acid (8.8 mg, 0.0554 mmol, 2.0 equiv.), DMAP (1.0 mg, 8.3 μmol, 0.3 equiv.), EDCI (16.0 mg, 0.0834 mmol, 3.0 equiv.) and dried DMF (0.4 mL). The vial was sealed with a Teflon cap, and the resulting solution was stirred at room temperature. After 18 h, the reaction mixture was diluted with EtOAc and the organic layer was washed with H_2O. The organic layer was

dried over $MgSO_4$, filtered, and concentrated by rotary evaporation. The residue was purified by flash column chromatography (EtOAc：*n*-Hexane = 1：1→MeOH：CH_2Cl_2 = 1：10→1：5) to afford **GHN059** (16.0 mg, 77% yield) as a yellowish foam.

TLC: R_f = 0.13 (MeOH：CH_2Cl_2 = 1：10, $KMnO_4$).

^{1}H NMR (600 MHz, $(CD_3)_2CO$, -40 °C) δ 5.85 (d, *J* = 7.6 Hz, 1H), 5.67 (d, *J* = 7.3 Hz, 1H), 5.52 (d, *J* = 10.4 Hz, 1H), 5.36 (dd, *J* = 7.3, 2.0 Hz, 1H), 5.16 (d, *J* = 2.3 Hz, 1H), 5.07 – 4.98 (m, 1H), 4.70 (br s, 1H), 4.01 (dd, *J* = 15.8, 7.3 Hz, 1H), 3.21, 3.18 (ABq, *J* = 16.9 Hz, 2H), 3.17 (dd, *J* = 10.6, 5.2 Hz, 1H), 2.77 – 2.66 (m, 2H), 2.68 – 2.58 (m, 3H), 2.41 (s, 3H), 2.30 – 2.15 (m, 3H), 2.23 (s, 6H), 2.14 – 2.01 (m, 3H), 2.12 (s, 3H), 1.99 – 1.91 (m, 2H) 1.95 (s, 3H), 1.91 – 1.84 (m, 1H), 1.53 – 1.47 (m, 2H), 1.50 (s, 3H), 1.09 (d, *J* = 7.1 Hz, 3H), 0.86 (s, 3H), 0.85 (t, *J* = 7.3 Hz, 3H).

^{13}C NMR (151 MHz, $(CD_3)_2CO$, -40 °C) δ 172.2, 172.1, 171.9, 170.7, 170.1, 169.9, 140.0, 122.6, 81.4, 74.2, 73.6, 70.2, 69.2, 65.2, 60.6, 59.0, 55.4, 52.8, 52.8, 46.2, 44.2, 40.3, 35.9, 34.3, 33.0, 27.2, 22.6, 22.2, 21.4, 18.4, 18.0, 13.7, 10.3, 10.0.

HRMS-ESI (m/z) calcd for $C_{37}H_{55}N_2O_{13}$ $[M+H]^+$ 735.3699, found 735.3694.

HPLC purity: t_R = 11.4 min, 95.8% (λ = 210 nm).

Synthesis of **GHN060**.

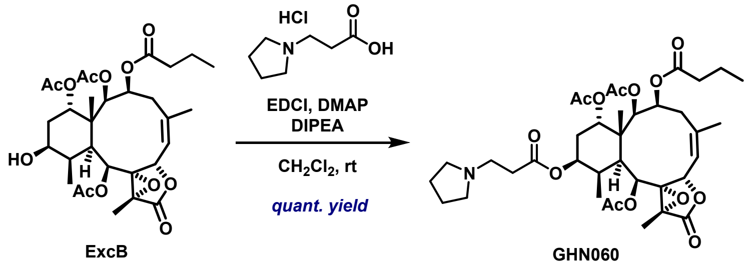

Under an ambient atmosphere, a 4 mL vial equipped with a stir bar was charged with **ExcB**

(16.4 mg, 0.0276 mmol, 1.0 equiv.), 3-(pyrrolidin-1-yl)propanoic acid hydrochloride (9.0 mg, 0.0628 mmol, 2.0 equiv.), DIPEA (2 drops), DMAP (1.0 mg, 8.3 μmol, 0.3 equiv.), EDCI (19.0 mg, 0.0991 mmol, 3.0 equiv.) and dried CH_2Cl_2 (0.3 mL). The vial was sealed with a Teflon cap, and the resulting solution was stirred at room temperature. After 18 h, the reaction mixture was diluted with EtOAc and the organic layer was washed with H_2O. The organic layer was dried over $MgSO_4$, filtered, and concentrated by rotary evaporation. The residue was purified by flash column chromatography (EtOAc：*n*-Hexane = 1：1→ MeOH：CH_2Cl_2 = 1：10) gave **GHN060** (20.5 mg, quantitative yield) as a yellowish foam.

TLC: R_f = 0.25 (MeOH：CH_2Cl_2 = 1：10, $KMnO_4$).

^{1}H NMR (600 MHz, $(CD_3)_2CO$, -40 °C) δ 5.88 – 5.82 (m, 1H), 5.67 (d, *J* = 7.3 Hz, 1H), 5.53 (d, *J* = 10.3 Hz, 1H), 5.36 (d, *J* = 7.3 Hz, 1H), 5.16 (d, *J* = 2.4 Hz, 1H), 4.99 (ddd, *J* = 12.4, 5.0, 3.6 Hz, 1H), 4.72 – 4.68 (m, 1H), 4.01 (dd, *J* = 15.8, 7.2 Hz, 1H), 3.15 (dd, *J* = 10.3, 5.1 Hz, 1H), 2.71 – 2.58 (m, 3H), 2.55 – 2.41 (m, 6H), 2.41 (s, 3H), 2.23 (s, 6H), 2.21 – 2.07 (m, 3H), 2.04 – 2.00 (m, 1H), 1.95 (s, 3H), 1.89 – 1.84 (m, 1H), 1.71 – 1.63 (m, 4H), 1.57 – 1.46 (m, 2H), 1.50 (s, 3H), 1.10 (d, *J* = 7.1 Hz, 3H), 0.86 (s, 3H), 0.85 (t, *J* = 7.3 Hz, 3H).

^{13}C NMR (151 MHz, $(CD_3)_2CO$, -40 °C) δ 172.2, 172.1, 171.9, 171.7, 170.7, 170.1, 139.9, 122.6, 81.5, 74.2, 73.7, 70.0, 69.3, 65.3, 60.6, 54.2, 51.6, 44.2, 40.4, 35.9, 34.3, 33.0, 27.1, 23.6, 22.6, 22.2, 21.4, 18.4, 18.0, 13.7, 10.3, 10.0.

HRMS-ESI (m/z) calcd for $C_{37}H_{54}NO_{13}$ $[M+H]^+$ 720.3590, found 720.3598.

HPLC purity: t_R = 13.1 min, 95.9% (λ = 210 nm).

Synthesis of **GHN063**.

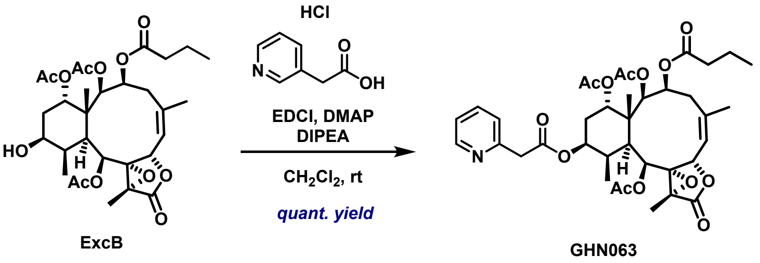

Under an ambient atmosphere, a 4 mL vial equipped with a stir bar was charged with **ExcB** (11.8 mg, 0.0198 mmol, 1.0 equiv.), 2-(pyridin-2-yl)acetic acid hydrochloride (6.9 mg, 0.0396 mmol, 2.0 equiv.), DIPEA (3 drops), DMAP (0.8 mg, 5.9 μmol, 0.3 equiv.), EDCI (11.4 mg, 0.0595 mmol, 3.0 equiv.) and dried CH_2Cl_2 (0.3 mL). The vial was sealed with a Teflon cap, and the resulting solution was stirred at room temperature. After 18 h, the reaction mixture was diluted with EtOAc and the organic layer was washed with H_2O. The organic layer was dried over $MgSO_4$, filtered, and concentrated by rotary evaporation. The residue was purified by flash column chromatography (EtOAc : *n*-Hexane = 1 : 1→ MeOH : CH_2Cl_2 = 1 : 10→1 : 5) to afford **GHN063** (14.1 mg, quantitative yield) as a yellowish foam.

TLC: R_f = 0.33 (EtOAc : *n*-Hexane = 1 : 1, $KMnO_4$).

^{1}H NMR (600 MHz, $(CD_3)_2CO$, -40 °C) δ 8.52 – 8.46 (m, 1H), 7.77 (td, *J* = 7.7, 1.8 Hz, 1H), 7.39 (d, *J* = 7.8 Hz, 1H), 7.28 (ddd, *J* = 7.6, 4.9, 1.1 Hz, 1H), 5.84 (dd, *J* = 6.9, 1.8 Hz, 1H), 5.66 (d, *J* = 7.3 Hz, 1H), 5.52 (d, *J* = 10.4 Hz, 1H), 5.39 – 5.33 (m, 1H), 5.16 (d, *J* = 2.4 Hz, 1H), 5.02 (ddd, *J* = 12.4, 5.0, 3.6 Hz, 1H), 4.72 – 4.67 (m, 1H), 4.00 (dd, *J* = 15.8, 7.3 Hz, 1H), 3.87, 3.84 (ABq, *J* = 16.0 Hz, 2H), 3.16 (dd, *J* = 10.4, 5.2 Hz, 1H), 2.68 – 2.60 (m, 1H), 2.40 (s, 3H), 2.22 (s, 3H), 2.19 (s, 3H), 2.19 – 2.06 (m, 2H), 2.03 – 1.99 (m, 1H), 1.94 (s, 3H), 1.91 – 1.86 (m, 1H), 1.55 – 1.46 (m, 2H), 1.51 (s, 3H) 1.06 (d,

J = 7.1 Hz, 3H), 0.85 (s, 3H), 0.85 (t, J = 7.5 Hz, 3H).

^{13}C NMR (151 MHz, $(CD_3)_2CO$, -40 ^oC) δ 172.2, 172.1, 171.9, 170.7, 170.5, 170.1, 155.5, 149.9, 140.0, 137.2, 124.9, 122.7, 122.6, 81.4, 74.2, 73.6, 70.7, 69.2, 65.3, 60.5, 44.2, 43.8, 40.3, 35.9, 34.3, 33.0, 27.1, 22.6, 22.2, 22.1, 21.4, 18.4, 18.0, 13.6, 10.2, 10.0. **HRMS-ESI** (m/z) calcd for $C_{37}H_{47}NO_{13}Na$ $[M+Na]^+$ 736.2940, found 736.2942.

HPLC purity: t_R = 13.0 min, 100.0% (λ = 210 nm).

Synthesis of **GHN070**.

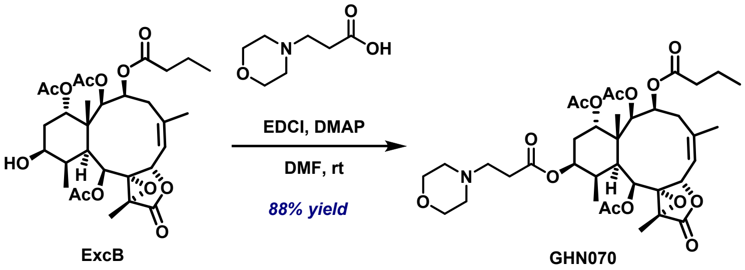

Under an ambient atmosphere, a 4 mL vial equipped with a stir bar was charged with **ExcB** (19.4 mg, 0.0326 mmol, 1.0 equiv.), 3-morpholinopropanoic acid (10.4 mg, 0.0625 mmol, 2.0 equiv.), DMAP (1.2 mg, 9.8 μmol, 0.3 equiv.), EDCI (18.8 mg, 0.0978 mmol, 3.0 equiv.) and dried DMF (0.4 mL). The vial was sealed with a Teflon cap, and the resulting solution was stirred at room temperature. After 18 h, the reaction mixture was diluted with EtOAc and the organic layer was washed with H_2O. The organic layer was dried over $MgSO_4$, filtered, and concentrated by rotary evaporation. The residue was purified by flash column chromatography (EtOAc : *n*-Hexane = 1 : 1→MeOH : CH_2Cl_2 = 1 : 20) gave **GHN070** (21.0 mg, 88% yield) as a yellowish foam.

TLC: R_f = 0.28 (MeOH : CH_2Cl_2 = 1 : 10, $KMnO_4$).

^{1}H NMR (600 MHz, $(CD_3)_2CO$, -40 ^oC) δ 5.85 (dd, J = 7.2, 1.4 Hz, 1H), 5.67 (d, J = 7.3

Hz, 1H), 5.53 (d, J = 10.3 Hz, 1H), 5.41 – 5.34 (m, 1H), 5.16 (d, J = 2.3 Hz, 1H), 5.01 (ddd, J = 12.5, 5.0, 3.6 Hz, 1H), 4.70 (dd, J = 4.0, 2.1 Hz, 1H), 4.01 (dd, J = 15.9, 7.2 Hz, 1H), 3.68 (br s, 2H), 3.36 (br s, 2H), 3.15 (dd, J = 10.4, 5.1 Hz, 1H), 2.74 – 2.61 (m, 3H), 2.56 – 2.51 (m, 2H), 2.51 – 2.44 (m, 2H), 2.41 (s, 3H), 2.23 (s, 6H), 2.21 – 2.02 (m, 4H), 2.02 – 1.98 (m, 1H), 1.96 (s, 3H), 1.90 – 1.84 (m, 1H), 1.56 – 1.45 (m, 2H), 1.51 (s, 3H), 1.10 (d, J = 7.1 Hz, 3H), 0.86 (s, 3H), 0.85 (t, J = 7.4 Hz, 3H).

^{13}C NMR (151 MHz, $(CD_3)_2CO$, -40 ^{o}C) δ 172.2, 172.1, 171.9, 171.7, 170.7, 170.1, 139.9, 122.6, 81.5, 74.3, 73.7, 69.9, 69.3, 67.0, 65.3, 60.6, 54.4, 53.9, 44.2, 40.4, 35.9, 34.3, 33.1, 32.4, 27.1, 22.6, 22.2, 21.4, 18.4, 18.1, 13.7, 10.3, 10.1.

HRMS-ESI (m/z) calcd for $C_{37}H_{54}NO_{14}$ $[M+H]^+$ 736.3539, found 736.3545.

HPLC purity: t_R = 12.6 min, 97.4% (λ = 210 nm).

Synthesis of **GHN072**.

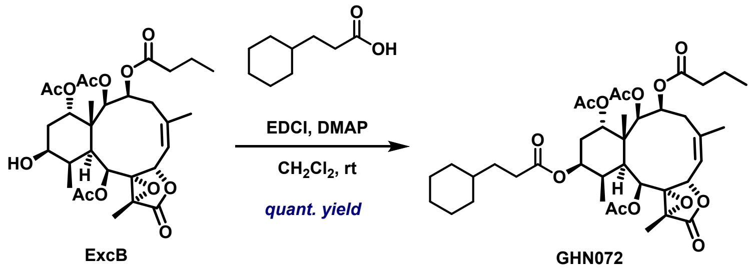

Under an ambient atmosphere, a 4 mL vial equipped with a stir bar was charged with **ExcB** (18.4 mg, 0.0309 mmol, 1.0 equiv.), 3-cyclohexylpropanoic acid (9.7 mg, 0.0618 mmol, 2.0 equiv.), DMAP (1.1 mg, 9.3 μmol, 0.3 equiv.), EDCI (17.8 mg, 0.0928 mmol, 3.0 equiv.) and dried CH_2Cl_2 (0.4 mL). The vial was sealed with a Teflon cap, and the resulting solution was stirred at room temperature. After 18 h, the reaction mixture was diluted with EtOAc and the organic layer was washed with H_2O. The organic layer was dried over

$MgSO_4$, filtered, and concentrated by rotary evaporation. The residue was purified by flash column chromatography (EtOAc : *n*-Hexane = 2 : 3) gave **GHN072** (22.6 mg, quantitative yield) as a white foam.

TLC: R_f = 0.58 (EtOAc : *n*-Hexane = 1 : 1, $KMnO_4$).

^{1}H NMR (600 MHz, $(CD_3)_2CO$, -40 °C) δ 5.88 – 5.82 (m, 1H), 5.67 (d, *J* = 7.3 Hz, 1H), 5.52 (d, *J* = 10.4 Hz, 1H), 5.40 – 5.32 (m, 1H), 5.16 (d, *J* = 2.3 Hz, 1H), 4.98 (dt, *J* = 12.4, 4.3 Hz, 1H), 4.70 (dd, *J* = 4.0, 2.1 Hz, 1H), 4.01 (dd, *J* = 15.8, 7.2 Hz, 1H), 3.15 (dd, *J* = 10.4, 5.1 Hz, 1H), 2.69 – 2.57 (m, 1H), 2.41 (s, 3H), 2.30 (td, *J* = 7.6, 4.7 Hz, 2H), 2.23 (s, 3H), 2.22 (s, 3H), 2.20 – 1.99 (m, 4H), 1.95 (s, 3H), 1.89 – 1.82 (m, 1H), 1.73 – 1.57 (m, 6H), 1.57 – 1.45 (m, 2H), 1.50 (s, 3H), 1.45 – 1.37 (m, 2H), 1.21 – 1.06 (m, 3H), 1.09 (d, *J* = 7.1 Hz, 3H), 0.90 – 0.77 (m, 2H), 0.86 (s, 3H), 0.85 (t, *J* = 7.5 Hz, 3H).

^{13}C NMR (151 MHz, $(CD_3)_2CO$, -40 °C) δ 173.1, 172.2, 172.1, 171.9, 170.7, 170.1, 140.0, 122.6, 81.5, 81.5, 74.2, 73.6, 69.9, 69.2, 65.3, 60.5, 44.2, 40.4, 37.5 (br), 35.9, 34.3, 33.3 (br), 33.0, 32.9 (br), 31.8, 27.1, 26.8, 26.7 (br), 22.6, 22.2, 21.4, 18.4, 18.1, 13.7, 10.3, 10.0.

HRMS-ESI (m/z) calcd for $C_{39}H_{56}O_{13}Na$ $[M+H]^+$ 755.3613, found 755.3621.

HPLC purity: t_R = 25.5 min, 100.0% (λ = 210 nm).

Synthesis of **GHN073**.

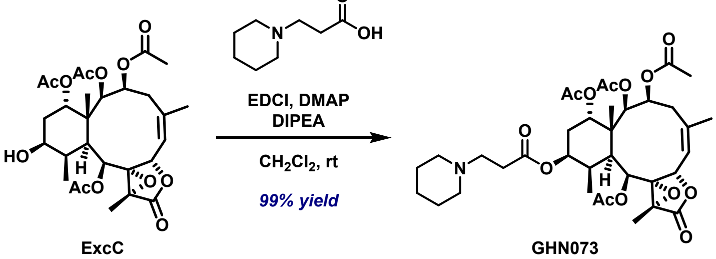

Under an ambient atmosphere, a 4 mL vial equipped with a stir bar was charged with **ExcC**

(16.0 mg, 0.0282 mmol, 1.0 equiv.), 3-(piperidin-1-yl)propanoic acid (8.9 mg, 0.0564 mmol, 2.0 equiv.), DMAP (1.0 mg, 8.5 μmol, 0.3 equiv.), EDCI (16.2 mg, 0.0847 mmol, 3.0 equiv.) and dried CH_2Cl_2 (0.3 mL). The vial was sealed with a Teflon cap, and the resulting solution was stirred at room temperature. After 18 h, the reaction mixture was diluted with EtOAc and the organic layer was washed with H_2O. The organic layer was dried over $MgSO_4$, filtered, and concentrated by rotary evaporation. The residue was purified by flash column chromatography (EtOAc：*n*-Hexane = 1：1→MeOH：CH_2Cl_2 = 1：10) gave **GHN073** (14.7 mg, 74% yield) as a yellowish oil.

TLC: R_f = 0.43 (MeOH：CH_2Cl_2 = 1：10, $KMnO_4$).

^{1}H NMR (600 MHz, $(CD_3)_2CO$, -40 ^{o}C) δ 5.84 – 5.76 (m, 1H), 5.67 (d, *J* = 7.4 Hz, 1H), 5.55 (d, *J* = 8.3 Hz, 1H), 5.53 (d, *J* = 8.3 Hz, 1H), 5.39 – 5.33 (m, 1H), 5.16 (dd, *J* = 5.6, 2.4 Hz, 1H), 5.03 – 4.96 (m, 1H), 4.68 (dd, *J* = 4.0, 2.1 Hz, 1H), 4.64 (dd, *J* = 3.8, 2.2 Hz, 1H), 4.00 (ddd, *J* = 15.7, 7.5, 3.2 Hz, 1H), 3.91 (dt, *J* = 12.5, 4.3 Hz, 1H), 3.58 (s, 3H), 3.14 (dd, *J* = 10.3, 5.1 Hz, 1H), 3.08 (dd, *J* = 10.4, 5.1 Hz, 1H), 2.93 – 2.73 (m, 2H), 2.69 – 2.60 (m, 1H), 2.54 – 2.42 (m, 4H), 2.41 (s, 3H), 2.40 (s, 3H), 2.23 (s, 3H), 2.21 (d, *J* = 0.9 Hz, 6H), 2.19 (s, 3H), 2.15 – 2.06 (m, 1H), 1.97 – 1.92 (m, 3H), 1.87 (d, *J* = 4.5 Hz, 4H), 1.85 – 1.74 (m, 2H), 1.75 – 1.68 (m, 1H), 1.66 – 1.34 (m, 7H), 1.10 (d, *J* = 7.1 Hz, 3H), 1.02 (d, *J* = 7.1 Hz, 3H), 0.85 (s, 3H), 0.81 (s, 3H).

^{13}C NMR (151 MHz, $(CD_3)_2CO$, -40 ^{o}C) δ 172.4, 172.3, 172.2, 171.9, 170.7, 170.1, 170.1, 169.7, 169.6, 139.9, 139.9, 122.6, 122.6, 82.1, 81.5, 81.4, 81.3, 74.3, 74.2, 73.8, 69.9, 69.3, 69.3, 65.9, 65.6, 65.3, 60.5, 60.4, 54.7, 54.6, 54.6, 51.5, 44.3, 44.1, 40.3, 40.2, 35.8, 34.2, 34.1, 33.0, 32.7, 32.0, 27.1, 26.2, 24.7, 22.7, 22.6, 22.3, 22.2, 22.1, 21.4, 21.4, 21.2, 20.8, 20.8, 18.3, 18.1, 18.0, 10.3, 10.0, 9.2.

Note: Two major conformers were observed at 233K in $(CD_3)_2CO$ in a 60/40 ratio.

HRMS-ESI (m/z) calcd for $C_{36}H_{52}NO_{13}$ $[M+H]^+$ 706.3433, found 706.3433.

HPLC purity: t_R = 9.36 min, 98.4% (λ = 210 nm).

Synthesis of **GHN085**.

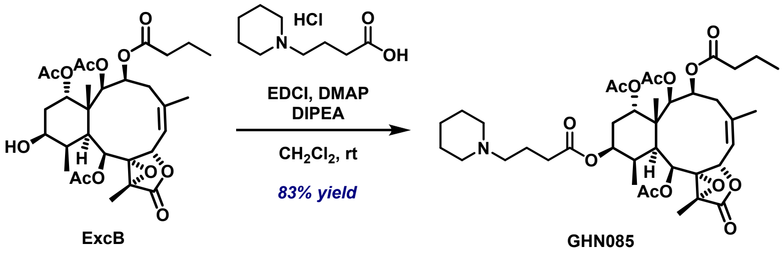

Under an ambient atmosphere, a 4 mL vial equipped with a stir bar was charged with **ExcB** (18.3 mg, 0.0308 mmol, 1.0 equiv.), 4-(piperidin-1-yl)butanoic acid hydrochloride (12.8 mg, 0.0616 mmol, 2.0 equiv.), DMAP (1.2 mg, 9.2 μmol, 0.3 equiv.), DIPEA (2 drops, excess), EDCI (17.7 mg, 0.0923 mmol, 3.0 equiv.) and dried CH_2Cl_2 (0.3 mL). The vial was sealed with a Teflon cap, and the resulting solution was stirred at room temperature. After 18 h, the reaction mixture was diluted with EtOAc and the organic layer was washed with H_2O. The organic layer was dried over $MgSO_4$, filtered, and concentrated by rotary evaporation. The residue was purified by flash column chromatography (EtOAc : *n*-Hexane = 1 : 1→MeOH : CH_2Cl_2 = 1 : 10) gave **GHN085** (19.2 mg, 83% yield) as a yellowish oil.

TLC: R_f = 0.30 (MeOH : CH_2Cl_2 = 1 : 10, $KMnO_4$).

^{1}H NMR (600 MHz, $(CD_3)_2CO$, -40 °C) δ 5.85 (dd, *J* = 7.2, 2.5 Hz, 1H), 5.67 (d, *J* = 7.3 Hz, 1H), 5.53 (d, *J* = 10.4 Hz, 1H), 5.36 (dt, *J* = 7.4, 1.8 Hz, 1H), 5.16 (d, *J* = 2.4 Hz, 1H), 4.99 (dt, *J* = 12.5, 4.3 Hz, 1H), 4.70 (dd, *J* = 4.0, 2.1 Hz, 1H), 4.01 (dd, *J* = 15.8, 7.3 Hz,

1H), 3.16 (dd, J = 10.4, 5.1 Hz, 1H), 2.94 (br s, 2H), 2.68 – 2.60 (m, 1H), 2.57 – 2.31 (m, 4H), 2.41 (s, 3H), 2.23 (s, 3H), 2.22 (s, 3H), 2.21 – 1.99 (m, 4H), 1.95 (s, 3H), 1.90 – 1.41 (m, 12H), 1.50 (s, 3H), 1.19 – 1.04 (m, 1H), 1.09 (d, J = 7.1 Hz, 3H), 0.86 (s, 3H), 0.85 (t, J = 7.4 Hz, 3H).

^{13}C NMR (151 MHz, $(CD_3)_2CO$, -40 °C) δ 172.7, 172.3, 172.1, 171.9, 170.7, 170.1, 140.0, 122.6, 81.5, 81.4, 74.2, 73.6, 70.0, 69.2, 65.3, 60.5, 44.2, 40.3, 35.9, 34.3, 33.0, 32.1, 27.1, 22.6, 22.1, 21.4, 18.4, 18.0, 13.6, 10.3, 10.0.

HRMS-ESI (m/z) calcd for $C_{39}H_{58}NO_{13}$ $[M+H]^+$ 748.3903, found 748.3897.

HPLC purity: t_R = 13.5 min, 97.8% (λ = 210 nm).

Synthesis of **GHN086**.

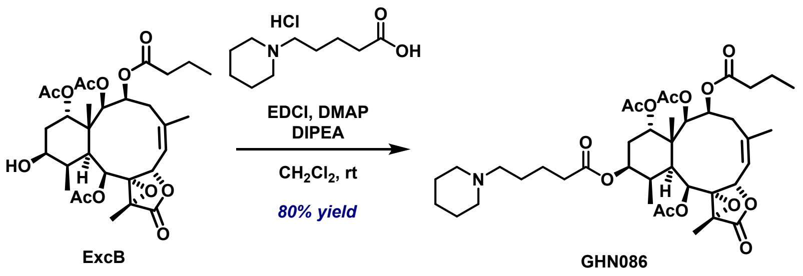

Under an ambient atmosphere, a 4 mL vial equipped with a stir bar was charged with **ExcB** (20.3 mg, 0.0341 mmol, 1.0 equiv.), 5-(piperidin-1-yl)pentanoic acid hydrochloride (15.1 mg, 0.0682 mmol, 2.0 equiv.), DMAP (1.2 mg, 9.2 μmol, 0.3 equiv.), DIPEA (2 drops, excess), EDCI (19.6 mg, 0.1024 mmol, 3.0 equiv.) and dried CH_2Cl_2 (0.4 mL). The vial was sealed with a Teflon cap, and the resulting solution was stirred at room temperature. After 18 h, the reaction mixture was diluted with EtOAc and the organic layer was washed with H_2O. The organic layer was dried over $MgSO_4$, filtered, and concentrated by rotary evaporation. The residue was purified by flash column chromatography (EtOAc : *n*-

Hexane = 1 ∶ 1→MeOH ∶ CH_2Cl_2 = 1 ∶ 10) gave **GHN086** (20.9 mg, 80% yield) as a yellowish oil.

TLC: R_f = 0.25 (MeOH ∶ CH_2Cl_2 = 1 ∶ 10, $KMnO_4$).

^{1}H NMR (600 MHz, $(CD_3)_2CO$, -40 °C) δ 5.85 (dd, J = 7.3, 2.5 Hz, 1H), 5.67 (d, J = 7.3 Hz, 1H), 5.52 (d, J = 10.4 Hz, 1H), 5.36 (dt, J = 7.3, 1.8 Hz, 1H), 5.16 (d, J = 2.4 Hz, 1H), 4.99 (dt, J = 12.4, 4.3 Hz, 1H), 4.69 (dd, J = 4.0, 2.1 Hz, 1H), 4.01 (dd, J = 15.8, 7.3 Hz, 1H), 3.16 (dd, J = 10.4, 5.2 Hz, 1H), 3.00 (br s, 2H), 2.70 – 2.57 (m, 1H), 2.53 – 2.26 (m, 4H), 2.41 (s, 3H), 2.23 (s, 3H), 2.22 (s, 3H), 2.22 – 2.00 (m, 4H), 1.95 (s, 3H), 1.88 – 1.76 (m, 2H), 1.74 – 1.42 (m, 12H), 1.50 (s, 3H), 1.30 – 1.14 (m, 1H), 1.09 (d, J = 7.1 Hz, 3H), 0.86 (s, 3H), 0.85 (t, J = 7.4 Hz, 3H).

^{13}C NMR (151 MHz, $(CD_3)_2CO$, -40 °C) δ 172.7, 172.2, 172.1, 171.9, 170.7, 170.1, 140.0, 122.6, 81.5, 81.4, 74.2, 73.6, 70.0, 69.2, 65.3, 60.5, 58.3 (br), 54.3 (br), 44.2, 40.3, 35.9, 34.3, 34.0, 33.0, 25.7 (br), 27.1, 22.8, 22.6, 22.2, 21.4, 18.4, 18.0, 13.7, 10.3, 10.0.

HRMS-ESI (m/z) calcd for $C_{40}H_{60}NO_{13}$ $[M+H]^+$ 762.4059, found 762.4049.

HPLC purity: t_R = 14.1 min, 95.0% (λ = 210 nm).

Synthesis of **GHN103**.

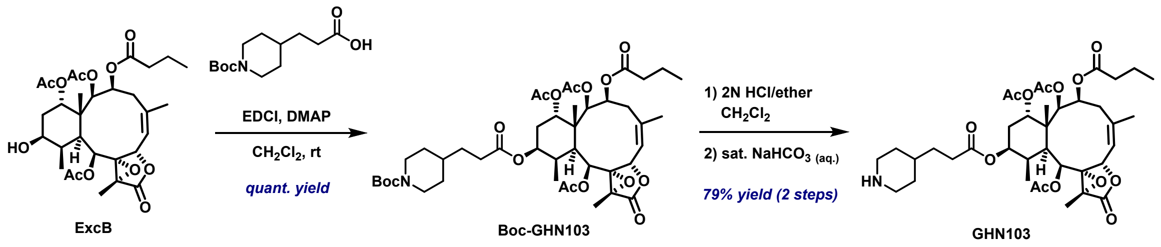

Under an ambient atmosphere, a 4 mL vial equipped with a stir bar was charged with **ExcB**

(31.8 mg, 0.0535 mmol, 1.0 equiv.), 3-(1-(*tert*-butoxycarbonyl)piperidin-4-yl)propanoic acid (27.5 mg, 0.1069 mmol, 2.0 equiv.), DMAP (2.0 mg, 16.1 μmol, 0.3 equiv.), EDCI (30.8 mg, 0.1607 mmol, 3.0 equiv.) and dried CH_2Cl_2 (0.4 mL). The vial was sealed with a Teflon cap, and the resulting solution was stirred at room temperature. After 18 h, the reaction mixture was diluted with EtOAc and the organic layer was washed with H_2O. The organic layer was dried over $MgSO_4$, filtered, and concentrated by rotary evaporation. The residue was purified by flash column chromatography (EtOAc：*n*-Hexane = 2：3→1：1) gave **Boc-GHN103** (44.5 mg, quantitative yield) as a colorless oil. To a solution of **Boc-GHN103** (44.5 mg, 0.0533 mmol, 1.0 equiv.) in dry CH_2Cl_2 (2 mL) was added 2N HCl/ether (0.3 mL, 0.53 mmol, 10.0 equiv.) at rt. After stirring at same temperature for 6 h, the reaction mixture was concentrated by rotary evaporation to afford a white solid (40.6 mg). The white solid was dissolved in CH_2Cl_2 (30 mL) then washed with saturated $NaHCO_3$ solution (2 times), brine, dried over $MgSO_4$, filtered, and concentrated by rotary evaporation to obtain **GHN103** (30.6 mg, 79% yield) as a white solid.

TLC: R_f = 0.18 (MeOH：CH_2Cl_2 = 1：10, $KMnO_4$).

^{1}H NMR (600 MHz, $(CD_3)_2CO$, -40 ^{o}C) δ 5.85 (dd, *J* = 7.2, 2.5 Hz, 1H), 5.73 (s, 1H), 5.67 (d, *J* = 7.3 Hz, 1H), 5.52 (d, *J* = 10.3 Hz, 1H), 5.36 (d, *J* = 7.2 Hz, 1H), 5.16 (d, *J* = 2.4 Hz, 1H), 4.98 (dt, *J* = 12.7, 4.2 Hz, 1H), 4.01 (dd, *J* = 15.8, 7.2 Hz, 1H), 3.20 – 3.07 (m, 3H), 2.69 – 2.55 (m, 3H), 2.41 (s, 3H), 2.37 – 2.29 (m, 2H), 2.22 (s, 6H), 2.21 – 2.04 (m, 4H), 2.04 – 1.99 (m, 1H), 1.95 (s, 3H), 1.90 – 1.82 (m, 1H), 1.71 (d, *J* = 12.9 Hz, 2H), 1.54 – 1.44 (m, 4H), 1.51 (s, 3H), 1.42 – 1.37 (m, 1H), 1.30 – 1.16 (m, 2H), 1.09 (d, *J* = 7.1 Hz, 3H), 0.85 (m, 6H).

^{13}C NMR (151 MHz, $(CD_3)_2CO$, -40 ^{o}C) δ 172.9, 172.2, 172.1, 171.9, 170.7, 170.1, 139.9,

122.6, 81.5, 81.4, 74.2, 73.6, 70.0, 69.2, 65.3, 60.5, 45.4 (br), 44.2, 40.3, 35.9, 35.1 (br), 34.3, 33.0, 32.0 (br), 31.4, 27.1, 22.6, 22.2, 21.4, 18.4, 18.0, 13.7, 10.3, 10.0. **HRMS-ESI** (m/z) calcd for $C_{38}H_{56}NO_{13}$ $[M+H]^+$ 734.3746, found 734.3741.

HPLC purity: t_R = 13.3 min, 97.7% (λ = 210 nm).

Synthesis of **GHN065**.

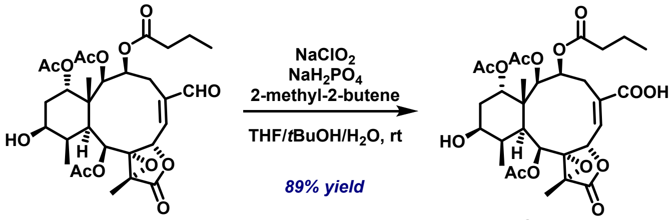

Under ambient atmosphere, a 4 mL vial equipped with a stir bar was charged with **GHN038** (54.1 mg, 0.091 mmol, 1.00 equiv.), THF (0.4 mL), *t*-BuOH (0.4 mL) and 2-methyl-2-butene (127.6 mg, d = 0.662 g/mL, 1.820 mmol, 20.0 equiv.) was added a solution of $NaClO_2$ (41.9 mg, 0.463 mmol, 5.5 equiv.), NaH_2PO_4 (65.7 mg, 0.547 mmol, 6.5 equiv.) in H_2O (0.8 mL). The vial was sealed with a Teflon cap, and the reaction mixture was stirred at room temperature. After 23 h, the reaction mixture was acidified with 1N HCl (pH~2) then extracted with EtOAc for three times. The combined organic layers were dried over $MgSO_4$, filtered, and concentrated by rotary evaporation. The residue was purified by flash column chromatography (EtOAc ∶ *n*-Hexane = 0 ∶ 1→2 ∶ 3→1 ∶ 1) to afford **GHN065** (49.4 mg, 89% yield) as a white solid.

TLC: R_f = 0.30 (EtOAc : *n*-Hexane = 1 ∶ 1, $KMnO_4$).

^{1}H NMR (600 MHz, $(CD_3)_2CO$, -40 °C) δ 12.05 (s, 1H), 6.70 (dd, *J* = 7.7, 2.3 Hz, 1H), 5.82 (dd, *J* = 7.6, 1.4 Hz, 1H), 5.73 (dd, *J* = 6.9, 2.3 Hz, 1H), 5.62 (d, *J* = 10.3 Hz, 1H),

5.20 (d, J = 2.5 Hz, 1H), 4.62 – 4.54 (m, 1H), 4.20 (d, J = 3.8 Hz, 1H, 12-OH), 3.93 (dd, J = 15.6, 7.6 Hz, 1H), 3.90 – 3.85 (m, 1H), 2.98 (dd, J = 10.4, 4.9 Hz, 1H), 2.58 – 2.51 (m, 2H), 2.41 (s, 3H), 2.21 (s, 3H), 2.17 – 2.10 (m, 1H), 2.09 – 2.04 (m, 1H), 2.03 (s, 3H), 1.91 – 1.81 (m, 1H), 1.80 – 1.74 (m, 1H), 1.53 (s, 3H), 1.49 (ddq, J = 11.1, 7.5, 3.7 Hz, 2H), 1.04 (d, J = 7.2 Hz, 3H), 0.87 (t, J = 7.4 Hz, 3H), 0.80 (s, 3H).

^{13}C NMR (151 MHz, $(CD_3)_2CO$, -40 ^{o}C) δ 172.2, 171.9, 171.7, 170.7, 169.9, 167.1, 136.6, 136.1, 82.0, 81.4, 75.3, 73.9, 69.7, 65.9, 65.3, 60.3, 44.7, 40.7, 36.1, 35.9, 30.0, 29.9, 22.7, 22.0, 21.4, 18.5, 18.0, 13.6, 9.9, 9.4.

HRMS-ESI (m/z) calcd for $C_{30}H_{40}O_{14}Na$ $[M+Na]^+$ 647.2310, found 647.2311.

HPLC purity: t_R = 9.4 min, 95.8% (λ = 210 nm).

Synthesis of **GHN102**.

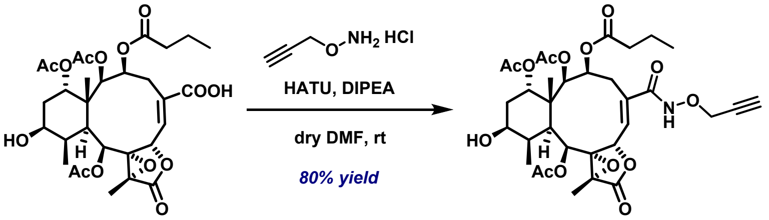

GHN065 GHN102

Under an ambient atmosphere, a 4 mL vial equipped with a stir bar was charged with **GHN065** (33.9 mg, 0.054 mmol, 1.0 equiv.), *O*-(prop-2-yn-1-yl)hydroxylamine hydrochloride (6.4 mg, 0.060 mmol, 1.1 equiv.), HATU (31.0 mg, 0.082 mmol, 1.5 equiv.), DIPEA (21.1 mg, 0.163 mmol, 3.0 equiv.) and dried DMF (0.3 mL). The vial was sealed with a Teflon cap, and the resulting solution was stirred at room temperature. After 20 h, the reaction mixture was diluted with EtOAc and the organic layer was washed with H_2O and brine. The organic layer was dried over $MgSO_4$, filtered, and concentrated by rotary

evaporation. The residue was purified by flash column chromatography (EtOAc ∶ *n*-Hexane = 1 ∶ 1→3 ∶ 1) gave **GHN102** (29.3 mg, 80% yield) as a white solid.

TLC (EtOAc : *n*-Hexane = 4 ∶ 1): R_f = 0.50 ($KMnO_4$).

^{1}H NMR (600 MHz, $(CD_3)_2CO$, -40 °C) δ 11.91 (s, 1H), 6.15 (dd, *J* = 7.3, 2.3 Hz, 1H), 5.77 (dd, *J* = 7.3, 1.5 Hz, 1H), 5.60 (d, *J* = 10.3 Hz, 1H), 5.53 (dd, *J* = 7.2, 2.3 Hz, 1H), 5.15 (d, *J* = 2.5 Hz, 1H), 4.64 – 4.55 (m, 2H), 4.56 – 4.52 (m, 1H), 4.08 (d, *J* = 4.4 Hz, 1H), 4.04 – 3.96 (m, 1H), 3.92 (dd, *J* = 15.6, 7.5 Hz, 1H), 3.35 (apparent t, *J* = 2.4 Hz, 1H), 2.99 (dd, *J* = 10.4, 4.9 Hz, 1H), 2.73 (d, *J* = 15.6 Hz, 1H), 2.56 – 2.49 (m, 1H), 2.40 (s, 3H), 2.20 (s, 3H), 2.17 – 2.09 (m, 1H), 2.05 (overlapped with residual solvent peak, 3H), 2.04 – 2.00 (m, 1H), 1.89 – 1.81 (m, 1H), 1.79 – 1.71 (m, 1H), 1.57 – 1.44 (m, 2H), 1.50 (s, 3H), 1.01 (d, *J* = 7.2 Hz, 3H), 0.88 (t, *J* = 7.4 Hz, 3H), 0.78 (s, 3H).

^{13}C NMR (151 MHz, $(CD_3)_2CO$, -40 °C) δ 172.2, 172.1, 171.7, 170.7, 170.1, 165.4, 138.6, 129.9, 82.2, 81.4, 79.3, 77.8, 74.7, 74.1, 69.5, 65.6, 65.4, 63.0, 60.1, 44.6, 40.4, 35.9, 35.5, 30.4, 22.7, 22.1, 21.4, 18.5, 18.0, 13.7, 10.0, 9.3.

HRMS-ESI (m/z) calcd for $C_{33}H_{43}NO_{14}Na$ $[M+Na]^+$ 700.2600, found 700.2606.

HPLC purity: t_R = 8.8 min, 98.9% (λ = 210 nm).

Synthesis of **GHN090.**

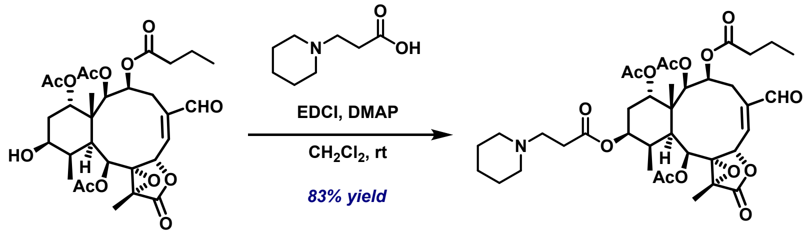

Under an ambient atmosphere, a 4 mL vial equipped with a stir bar was charged with **GHN038** (34.8 mg, 0.057 mmol, 1.0 equiv.), 3-(piperidin-1-yl)propanoic acid (11.7 mg, 0.074 mmol, 1.3 equiv.), DMAP (2.1 mg, 0.017 mmol, 0.3 equiv.), EDCI (21.9 mg, 0.114 mmol, 2.0 equiv.) and dried CH_2Cl_2 (0.6 mL). The vial was sealed with a Teflon cap, and the resulting solution was stirred at room temperature. After 20 h, the reaction mixture was directly purified by flash column chromatography (EtOAc : *n*-Hexane = 1 : 1→MeOH : CH_2Cl_2 = 1 : 10) gave **GHN090** (35.5 mg, 83% yield) as a colorless oil.

TLC (MeOH : CH_2Cl_2 = 1 : 10): R_f = 0.48 (UV 254 nm).

^{1}H NMR (600 MHz, $(CD_3)_2CO$, -40 ^{o}C) δ 9.68 (d, *J* = 1.5 Hz, 1H), 6.86 (dd, *J* = 7.4, 2.0 Hz, 1H), 5.96 (d, *J* = 7.3 Hz, 1H), 5.73 (dd, *J* = 7.3, 2.3 Hz, 1H), 5.63 (d, *J* = 10.3 Hz, 1H), 5.20 (d, *J* = 2.3 Hz, 1H), 4.93 (dt, *J* = 12.5, 4.5 Hz, 1H), 4.60 – 4.56 (m, 1H), 3.91 (dd, *J* = 15.8, 7.6 Hz, 1H), 2.98 (dd, 1H), 2.90 (br s, 2H), 2.72 – 2.65 (m, 1H), 2.64 – 2.41 (m, 4H), 2.47 (d, *J* = 15.6 Hz, 1H), 2.43 (s, 3H), 2.22 (s, 3H), 2.18 (dt, *J* = 16.0, 7.4 Hz, 1H), 2.11 – 2.07 (m, 1H), 2.02 (s, 3H), 2.00 – 1.96 (m, 1H), 1.93 – 1.74 (m, 3H), 1.69 – 1.36 (m, 7H), 1.54 (s, 3H), 1.16 – 1.09 (m, 1H), 1.11 (d, *J* = 7.1 Hz, 3H), 0.91 (t, *J* = 7.4 Hz, 3H), 0.83 (s, 3H).

^{13}C NMR (151 MHz, $(CD_3)_2CO$, -40 ^{o}C) δ 195.0, 172.2, 171.8, 171.7, 170.7, 170.0, 147.9, 144.3, 81.4, 81.2, 74.6, 73.9, 69.8, 64.9, 60.5, 54.5 (br), 44.7, 40.8, 35.9, 33.2, 32.7 (br),

27.6, 26.8, 26.0 (br), 24.5 (br), 22.7, 22.1, 21.4, 18.5, 17.8, 13.7, 10.3, 9.9.

HRMS-ESI (m/z) calcd for $C_{38}H_{53}NO_{14}Na$ $[M+Na]^+$ 770.3358, found 770.3360.

UPLC purity: t_R = 11.3 min, 97.0% (λ = 210 nm).

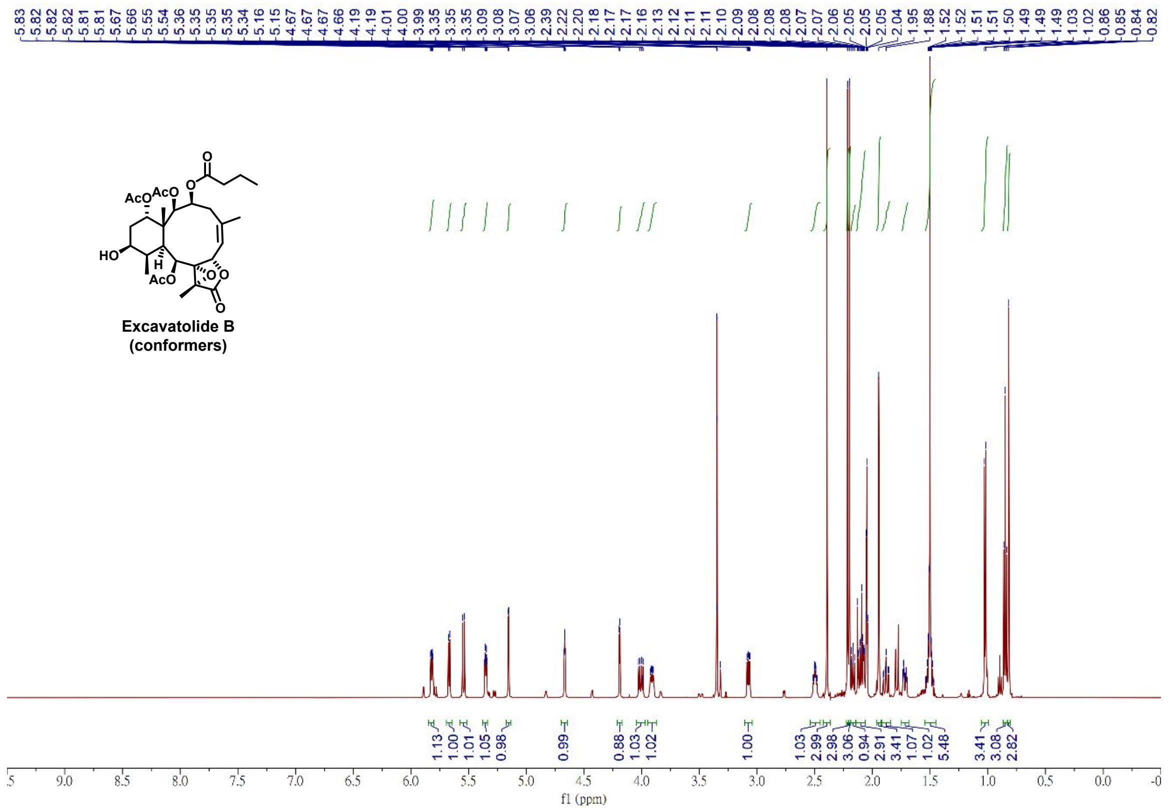

^{1}H NMR (600 MHz, $(CD_3)_2CO$, -40 °C) of **excavatolide B.**

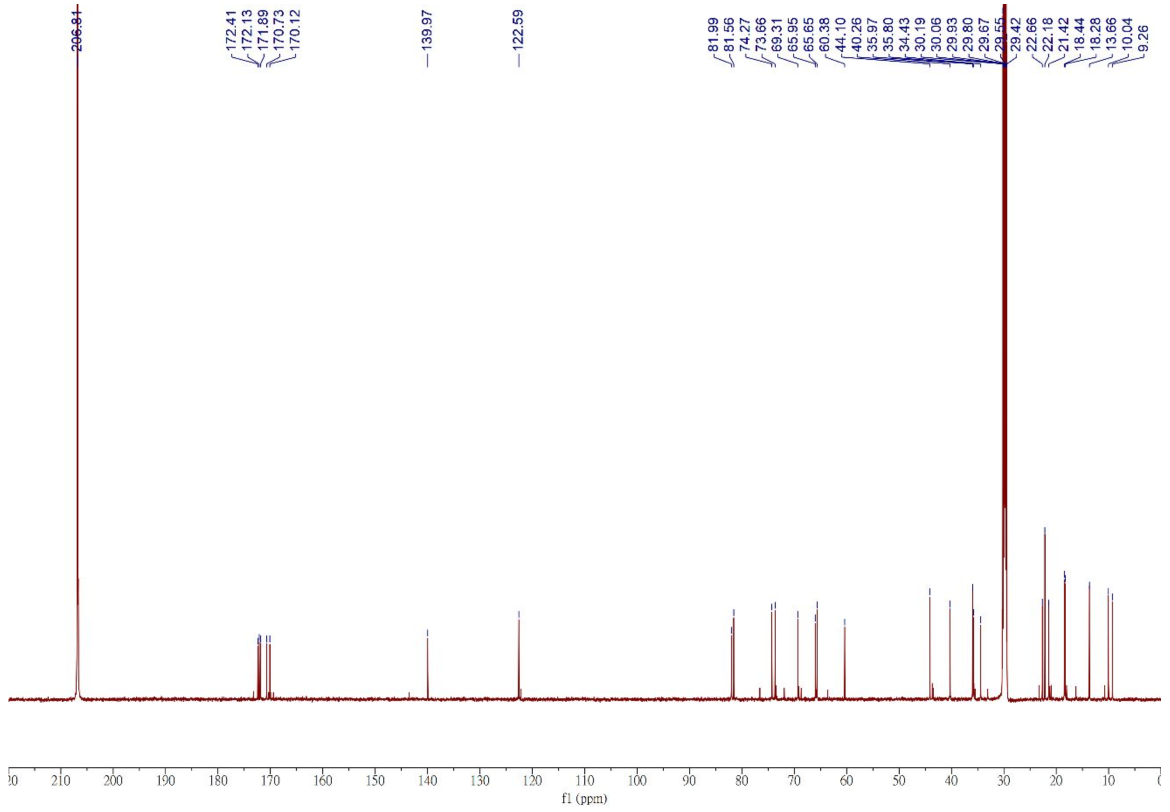

^{13}C NMR (151 MHz, $(CD_3)_2CO$, -40 °C) of **excavatolide B**.

Variable temperature for resolution improvement.

600 MHz ^{1}H NMR spectra of **ExcB** in $(CD_3)_2CO$.

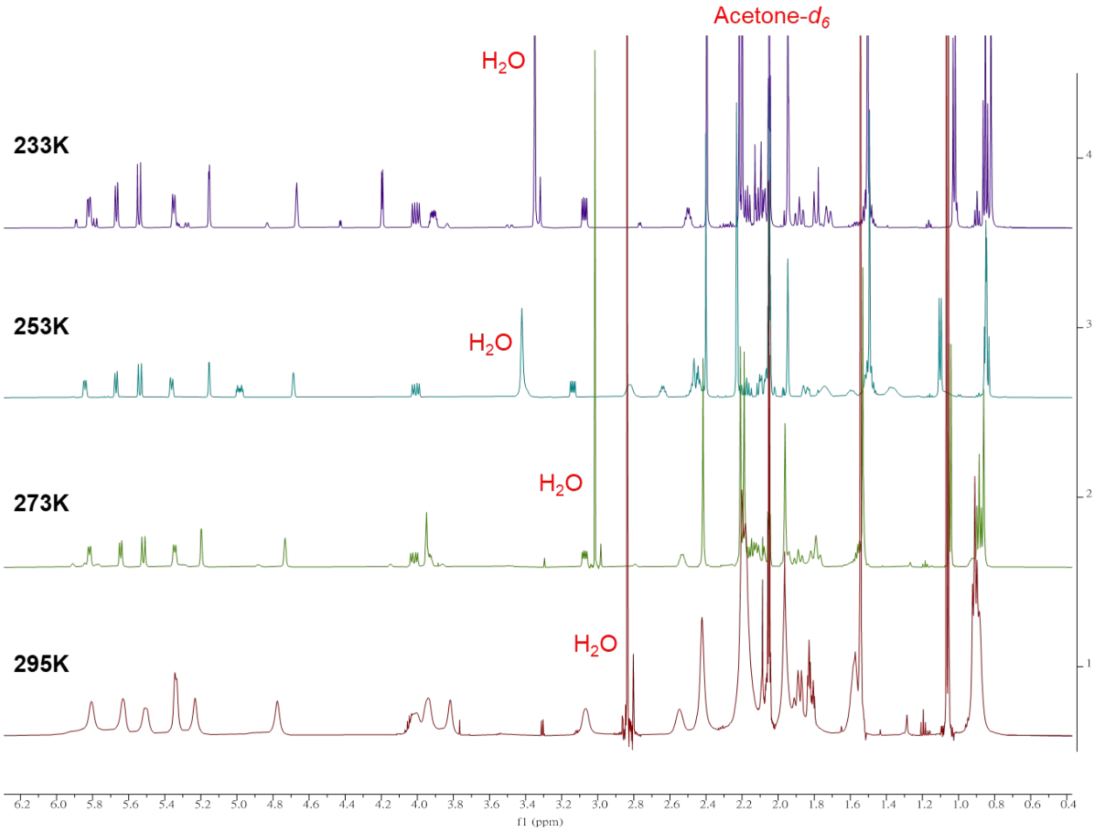

HPLC purity spectra of **excavatolide B**.

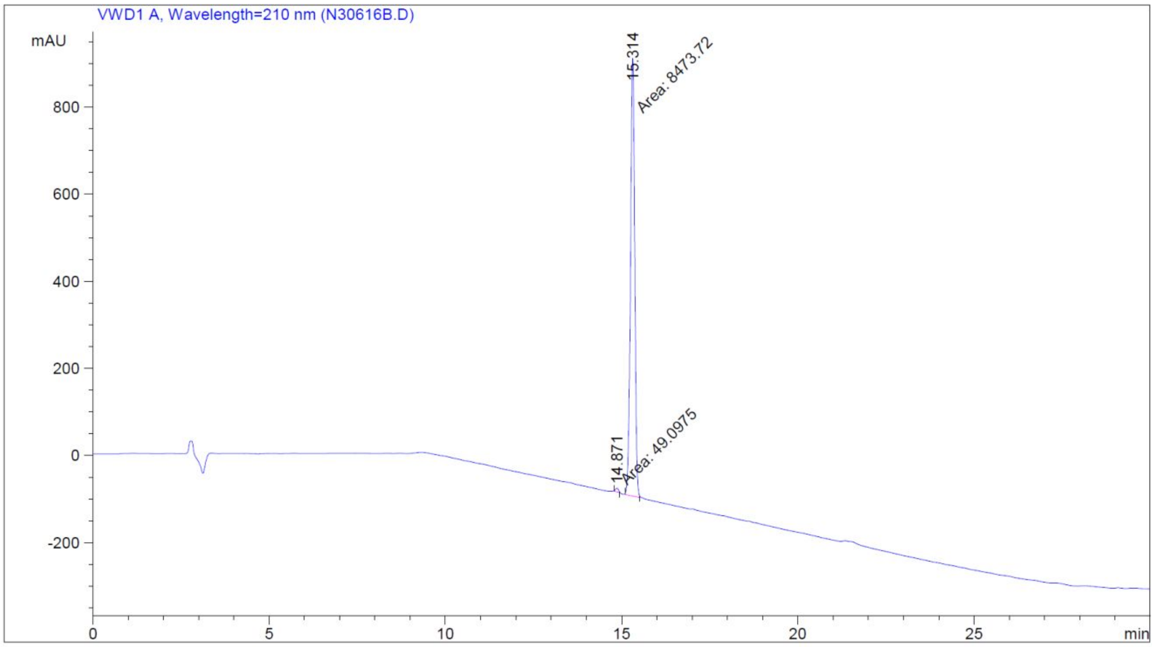


```
=====================================================================
                         Area Percent Report
=====================================================================

Sorted By             :      Signal
Multiplier            :      1.0000
Dilution              :      1.0000
Use Multiplier & Dilution Factor with ISTDs


Signal 1: VWD1 A, Wavelength=210 nm

Peak RetTime Type  Width     Area      Height     Area
  #   [min]        [min]  mAU   *s    [mAU   ]      %
----|-------|----|-------|----------|----------|--------|
   1  14.871 MM    0.0990   49.09754    8.26769   0.5761
   2  15.314 MM    0.1404 8473.71875 1005.84039  99.4239

Totals :                  8522.81629 1014.10809

 Results obtained with enhanced integrator!
=====================================================================
                         *** End of Report ***
```

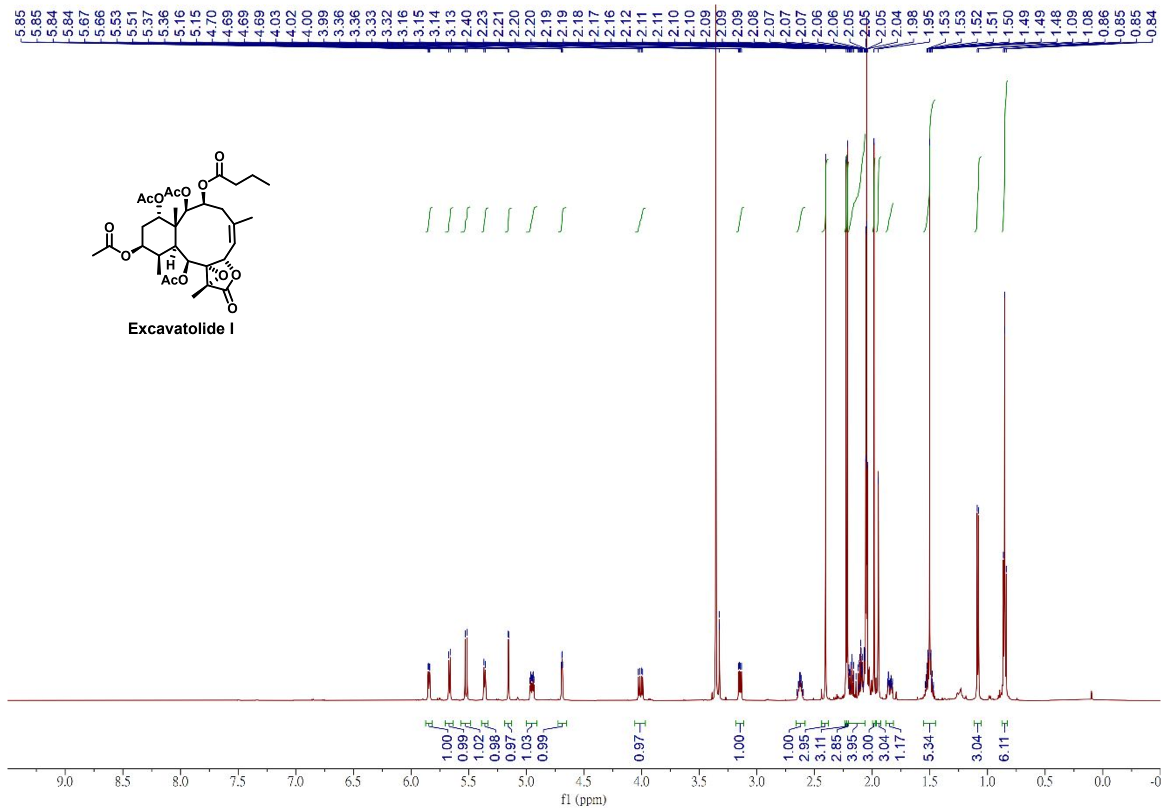

^{1}H NMR (600 MHz, $(CD_3)_2CO$, -40 ^oC) of **excavatolide I**.

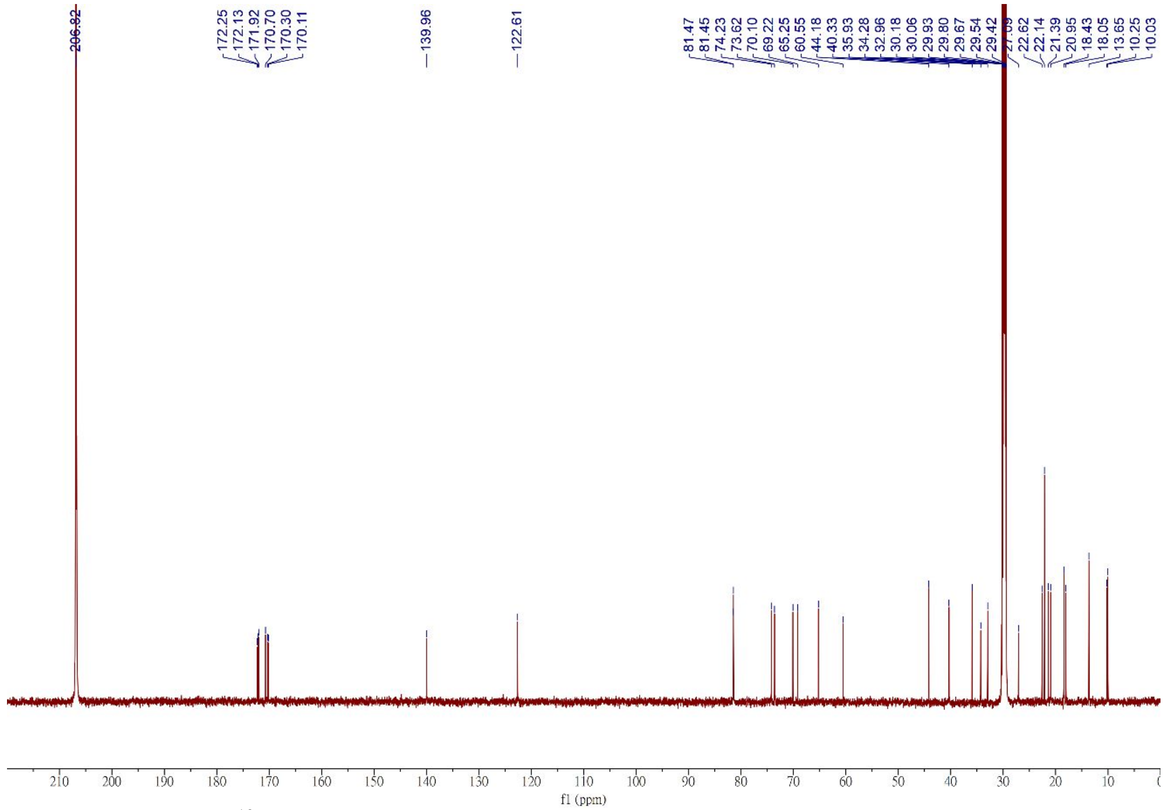

^{13}C NMR (151 MHz, $(CD_3)_2CO$, -40 ^oC) of **excavatolide I**.

HPLC purity spectra of **excavatolide I**.

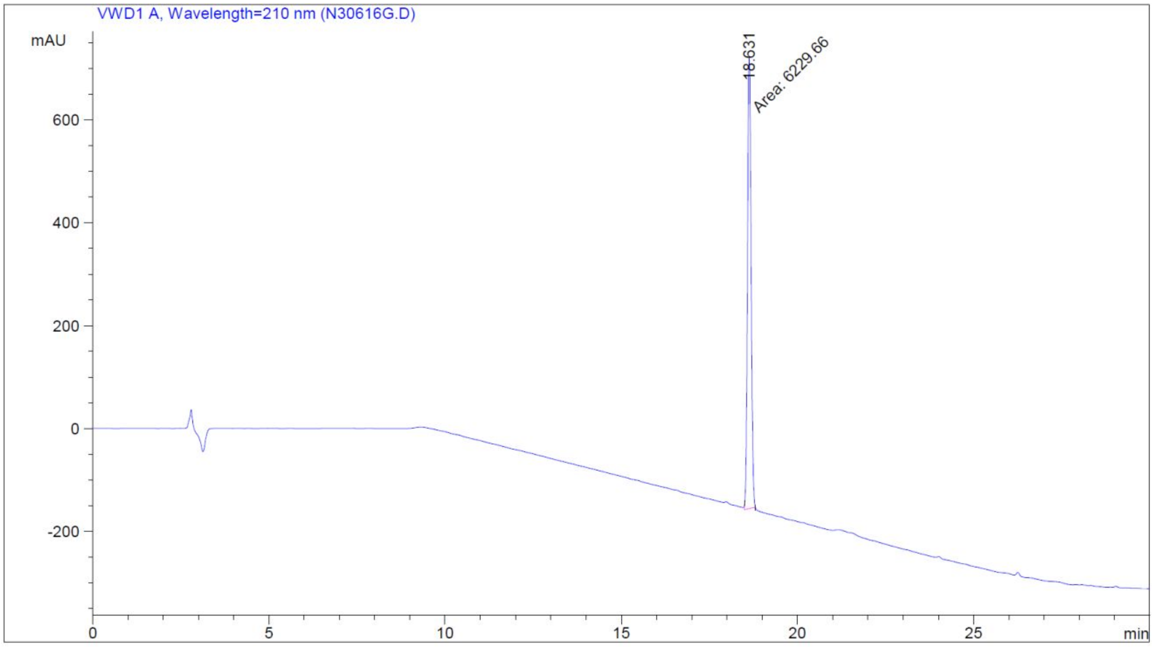


```
=====================================================================
                         Area Percent Report
=====================================================================

Sorted By             :      Signal
Multiplier            :      1.0000
Dilution              :      1.0000
Use Multiplier & Dilution Factor with ISTDs


Signal 1: VWD1 A, Wavelength=210 nm

Peak RetTime Type  Width     Area      Height     Area
  #   [min]        [min]  mAU   *s    [mAU   ]      %
----|-------|----|-------|----------|----------|--------|
   1  18.631 MM    0.1184 6229.66064  876.71411 100.0000

Totals :                  6229.66064  876.71411

 Results obtained with enhanced integrator!
=====================================================================
                          *** End of Report ***
```

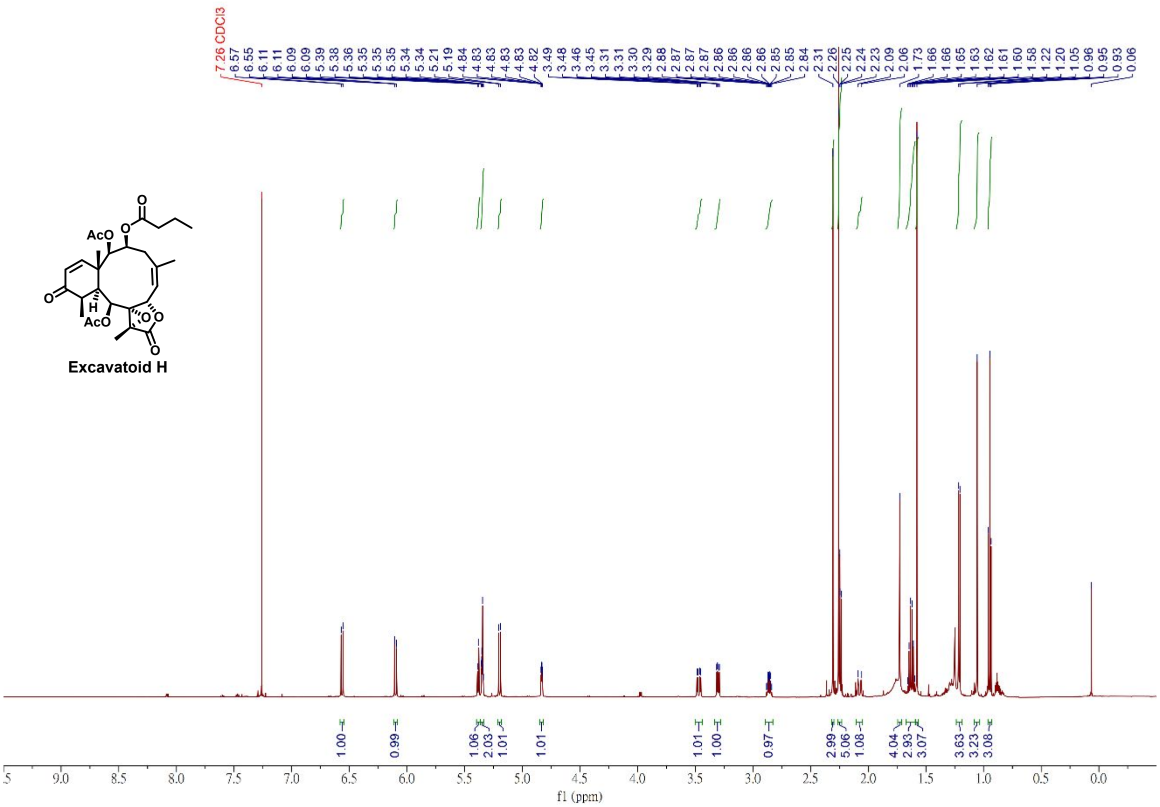

^{1}H NMR (600 MHz, $CDCl_3$, 22 ^{o}C) of **excavatoid H**.

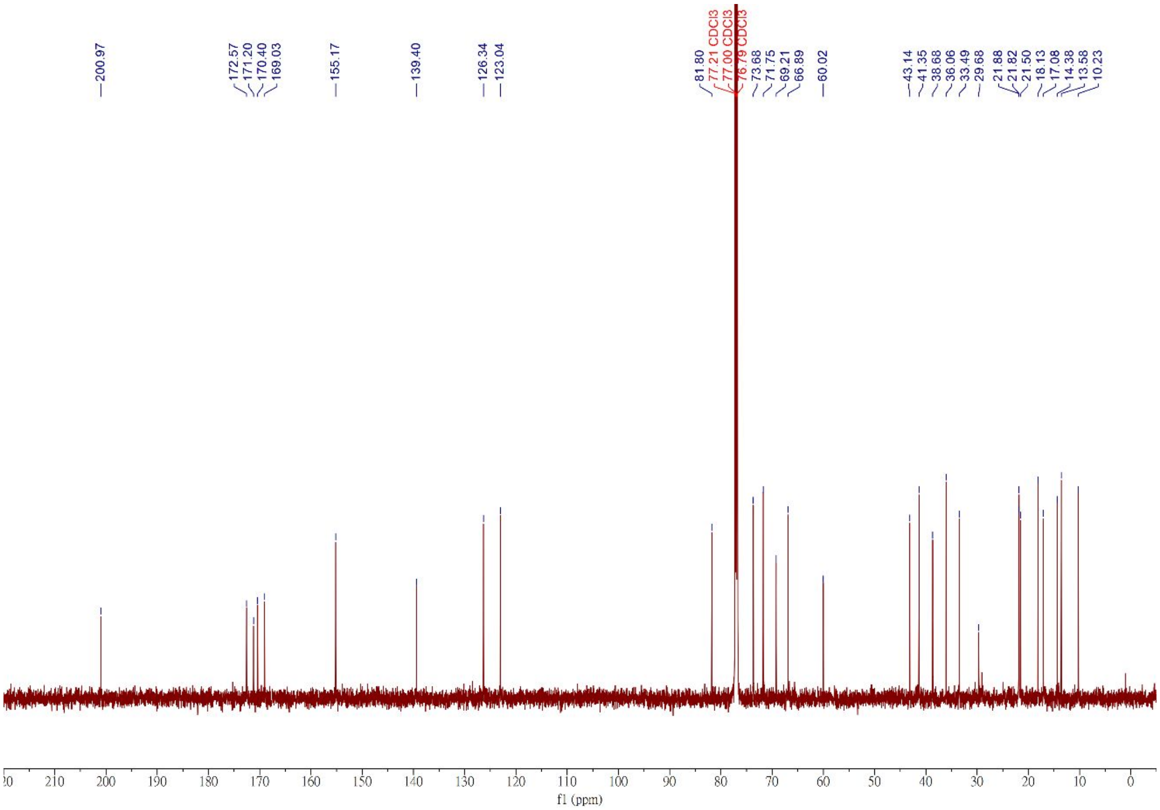

^{13}C NMR (151 MHz, $CDCl_3$, 22 ^{o}C) of **excavatoid H**.

HPLC purity spectra of **excavatoid H**.

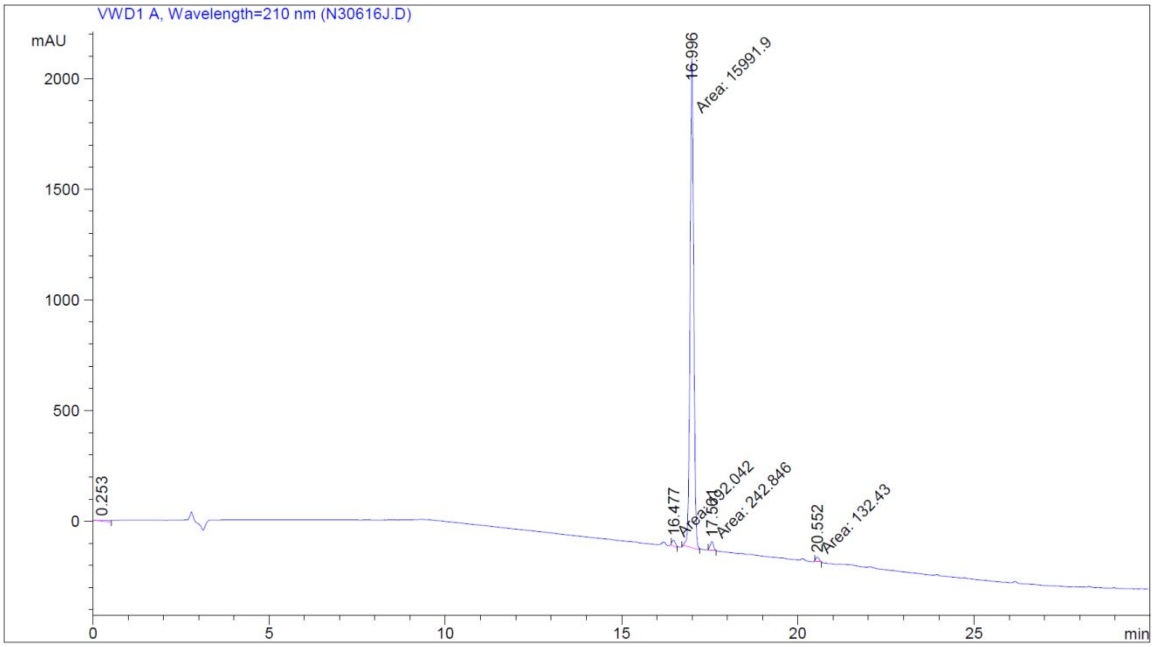


```
=====================================================================
                         Area Percent Report
=====================================================================

Sorted By             :      Signal
Multiplier            :      1.0000
Dilution              :      1.0000
Use Multiplier & Dilution Factor with ISTDs


Signal 1: VWD1 A, Wavelength=210 nm

Peak RetTime Type  Width     Area      Height     Area
  #   [min]        [min]  mAU   *s    [mAU   ]      %
----|-------|----|-------|----------|----------|--------|
   1   0.253 BV    0.3872  125.45933    4.20779   0.7519
   2  16.477 MM    0.1152  192.04205   27.77767   1.1510
   3  16.996 MM    0.1205 1.59919e4  2212.78784  95.8478
   4  17.561 MM    0.1069  242.84604   37.87573   1.4555
   5  20.552 MM    0.1061  132.42953   20.79831   0.7937

Totals :                  1.66847e4  2303.44734

 Results obtained with enhanced integrator!
=====================================================================
                         *** End of Report ***
```

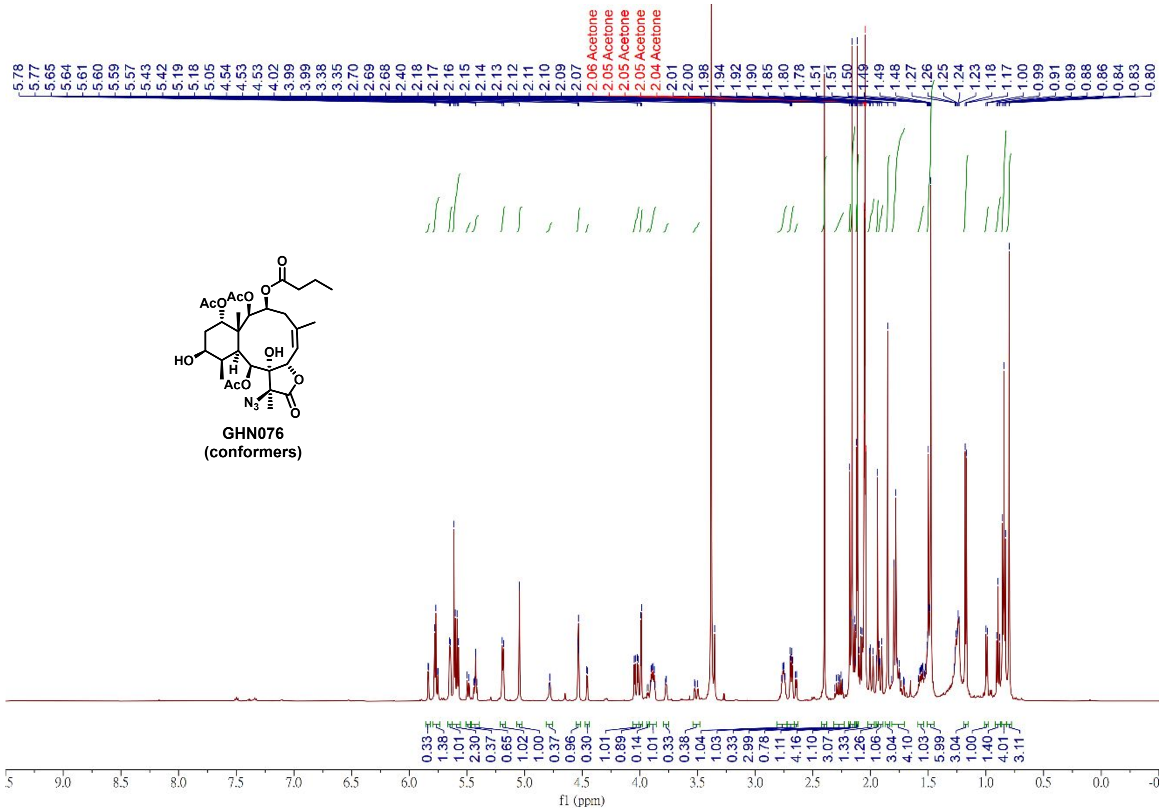

^{1}H NMR (600 MHz, $(CD_3)_2CO$, -40 ^{o}C) of **GHN076**.

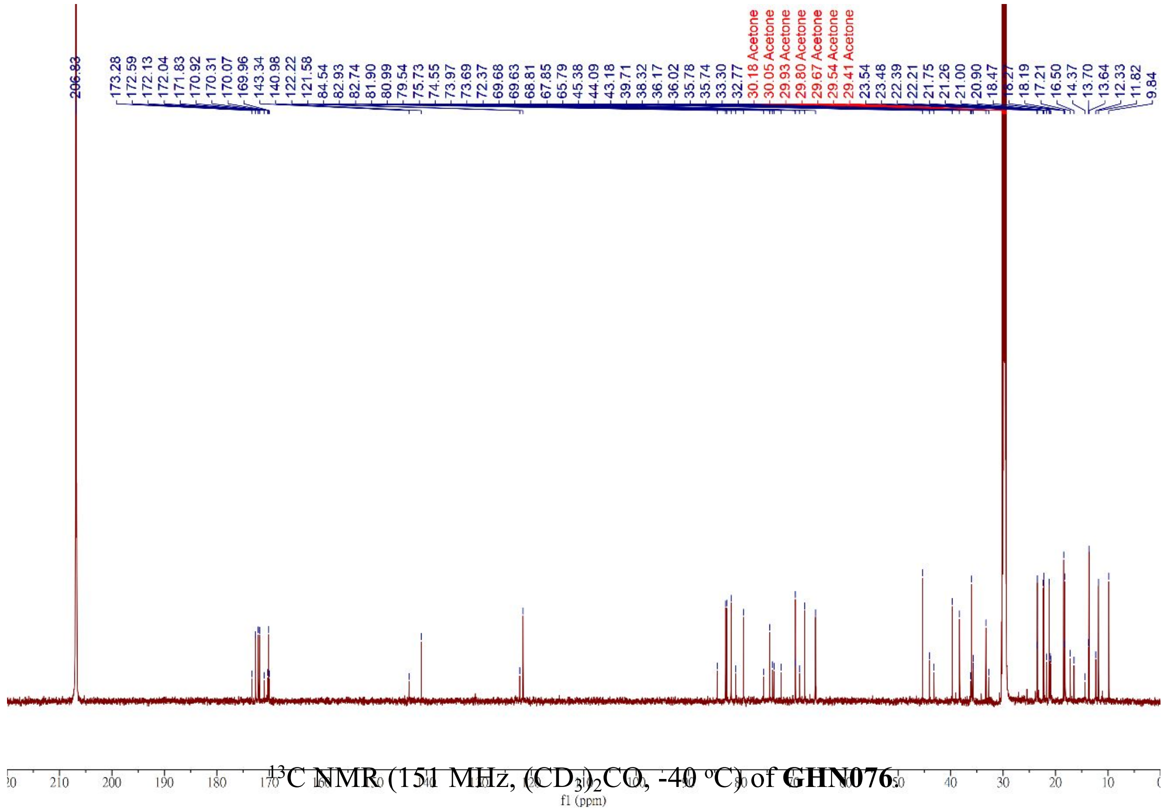

^{13}C NMR (151 MHz, $(CD_3)_2CO$, -40 ^{o}C) of **GHN076**.

HPLC purity spectra of **GHN076**.

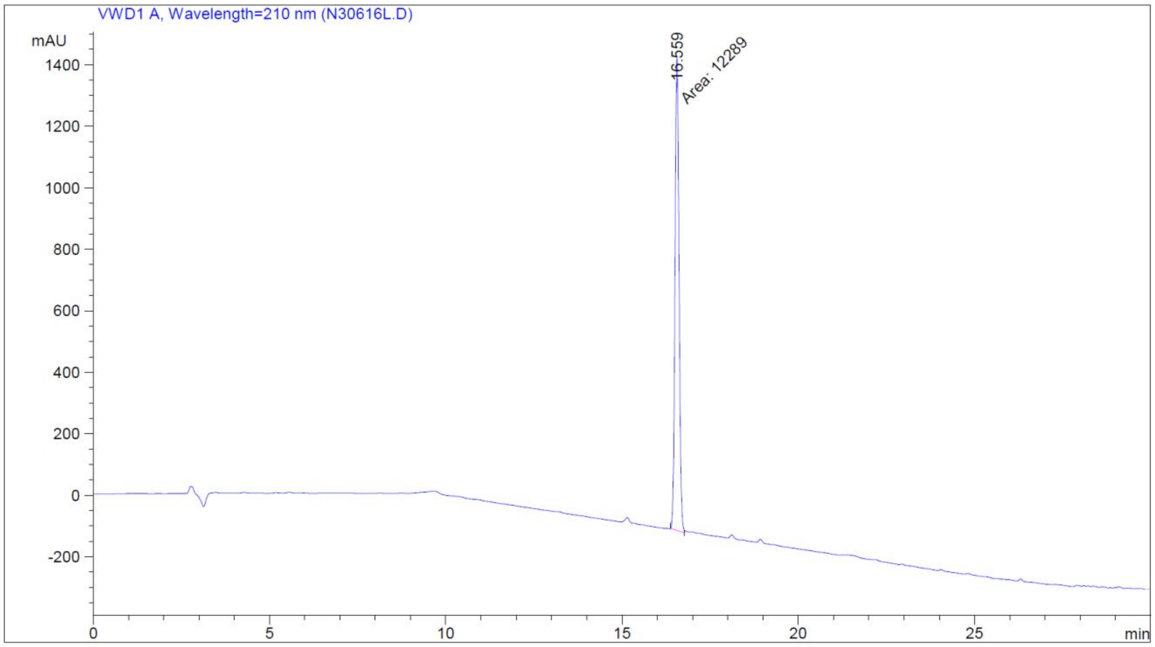


```
======================================================================
                          Area Percent Report
======================================================================

Sorted By             :      Signal
Multiplier            :      1.0000
Dilution              :      1.0000
Use Multiplier & Dilution Factor with ISTDs


Signal 1: VWD1 A, Wavelength=210 nm

Peak RetTime Type  Width     Area      Height     Area
  #   [min]        [min]  mAU    *s   [mAU   ]      %
----|-------|----|-------|----------|----------|--------|
   1  16.559 MM    0.1332 1.22890e4  1537.30200 100.0000

Totals :                  1.22890e4  1537.30200

 Results obtained with enhanced integrator!
======================================================================
                          *** End of Report ***
```

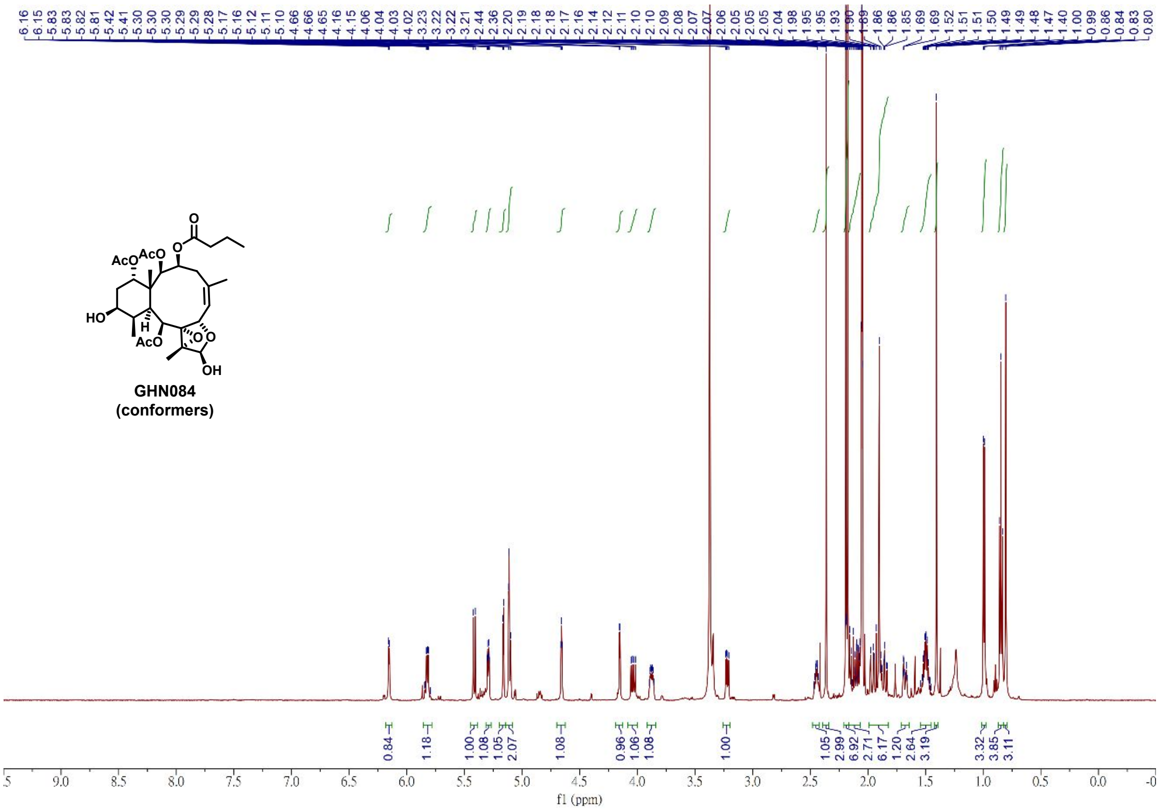

^{1}H NMR (600 MHz, $(CD_3)_2CO$, -40 °C) of **GHN084**.

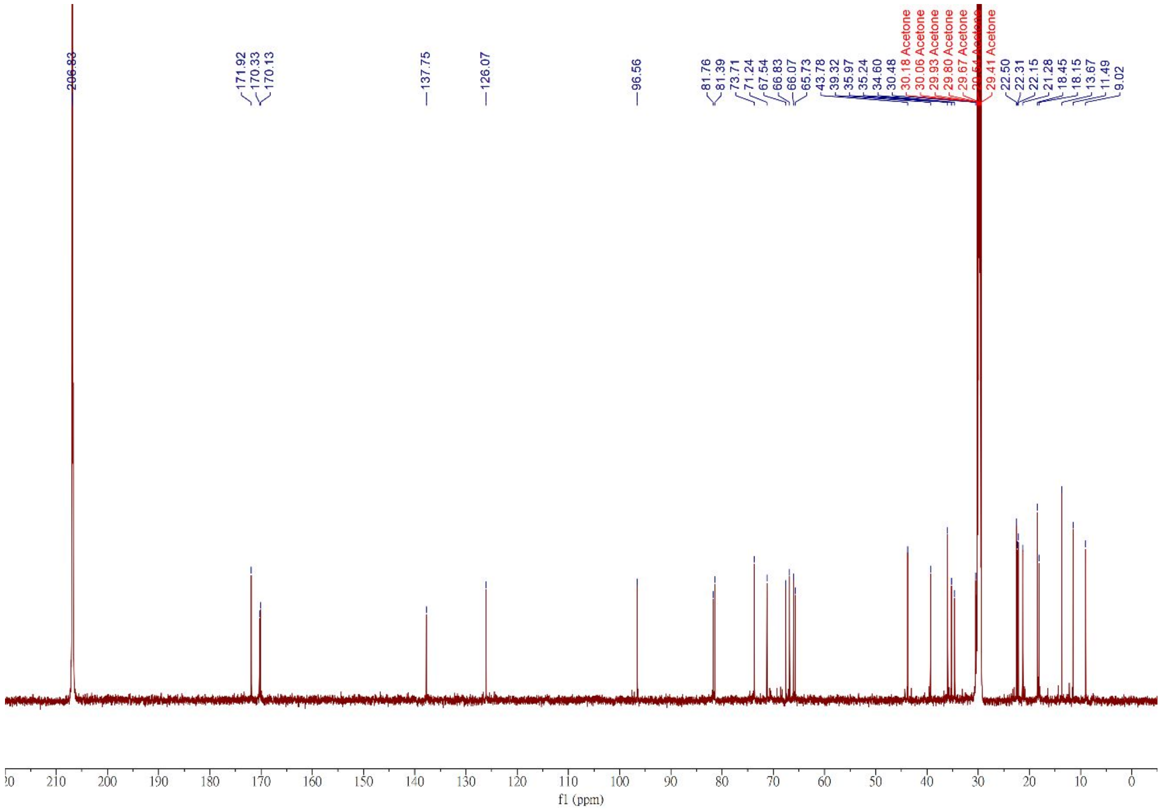

^{13}C NMR (151 MHz, $(CD_3)_2CO$, -40 °C) of **GHN084**.

HPLC purity spectra of **GHN084**.

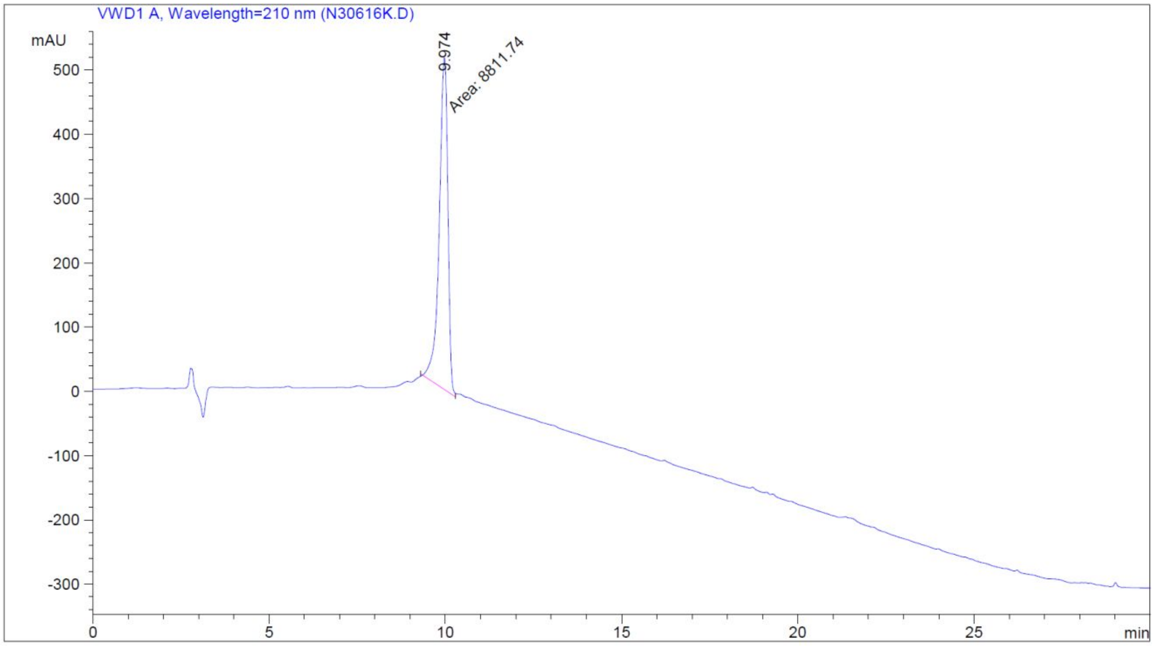


```
==================================================================
                        Area Percent Report
==================================================================

Sorted By             :      Signal
Multiplier            :      1.0000
Dilution              :      1.0000
Use Multiplier & Dilution Factor with ISTDs


Signal 1: VWD1 A, Wavelength=210 nm

Peak RetTime Type  Width     Area      Height     Area
  #   [min]        [min]  mAU   *s    [mAU   ]      %
----|-------|----|-------|----------|----------|--------|
   1   9.974 MM    0.2845 8811.73828  516.23907 100.0000

Totals :                  8811.73828  516.23907

 Results obtained with enhanced integrator!
==================================================================
                         *** End of Report ***
```

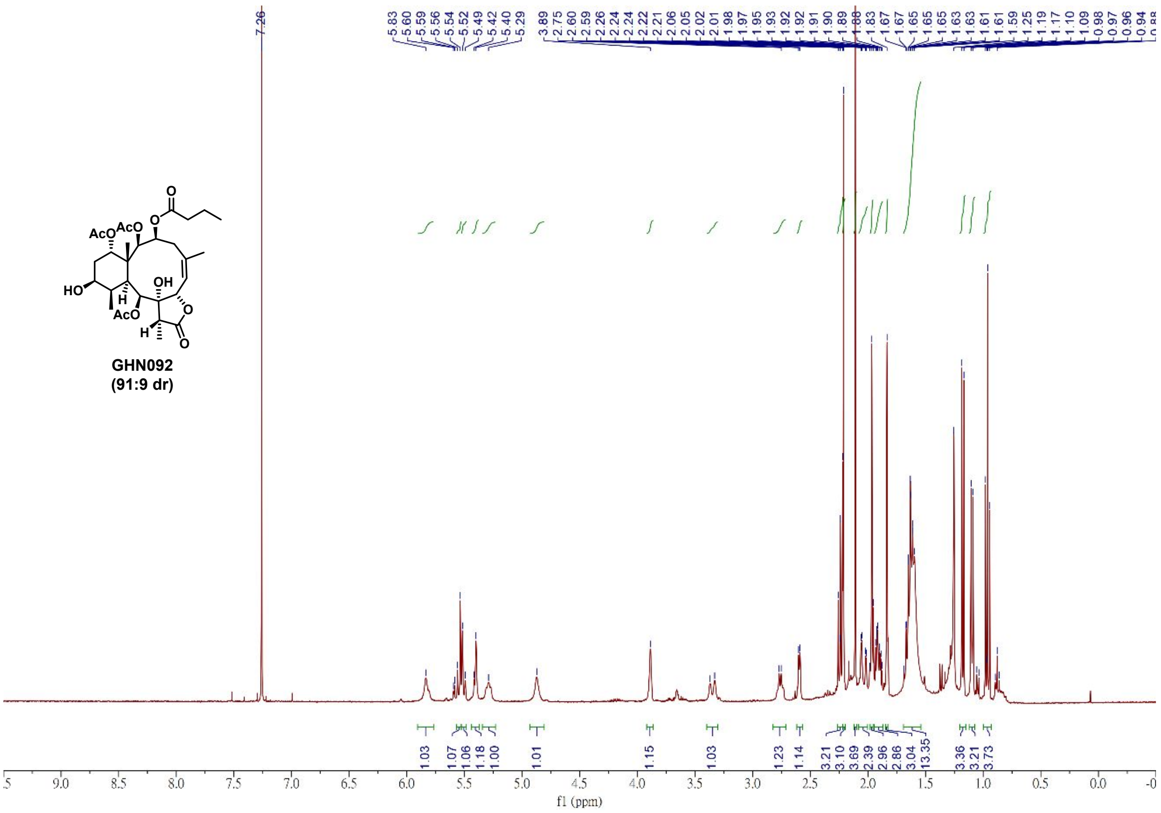

^1H NMR (400 MHz, $CDCl_3$, 24.5 ^oC) of **GHN092**.

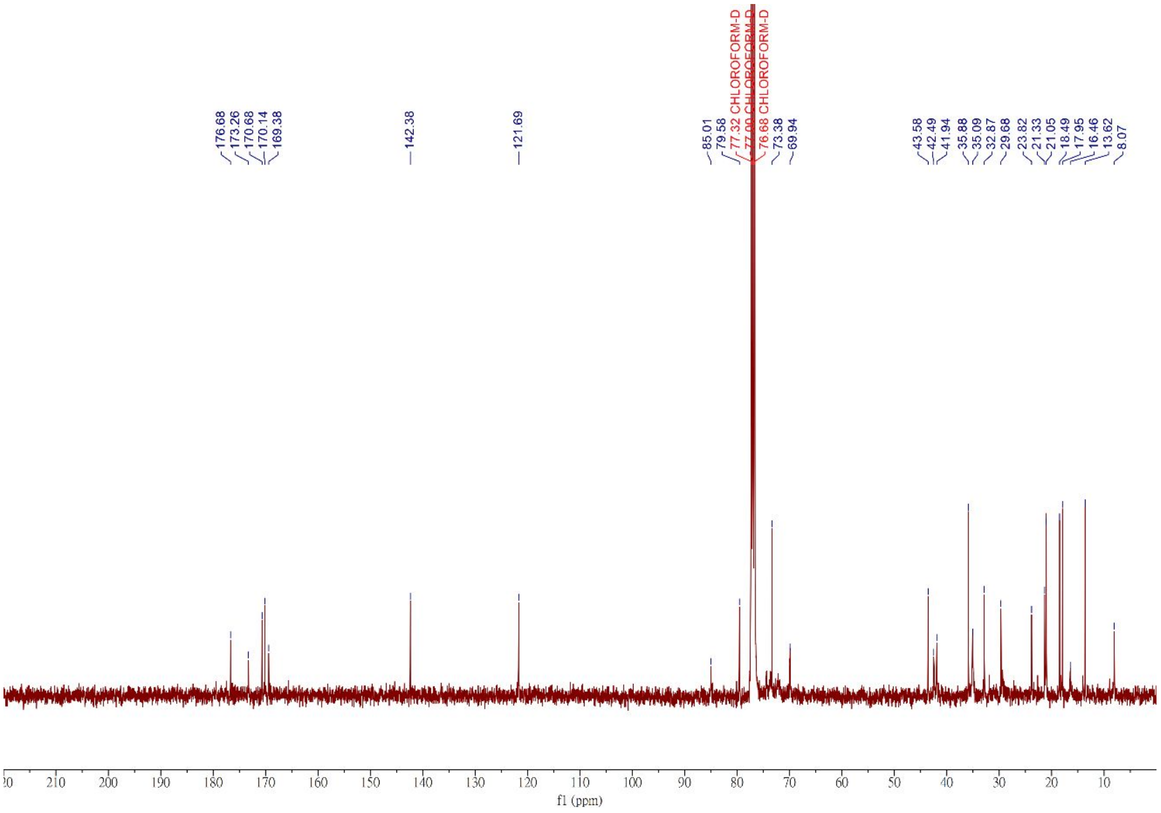

^{13}C NMR (101 MHz, $CDCl_3$, 24.5 ^oC) of **GHN092**.

NOSEY (400 MHz, $CDCl_3$, 24.5 °C) of **GHN092**.

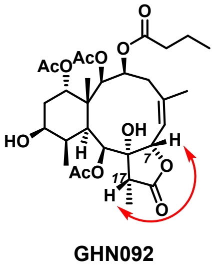

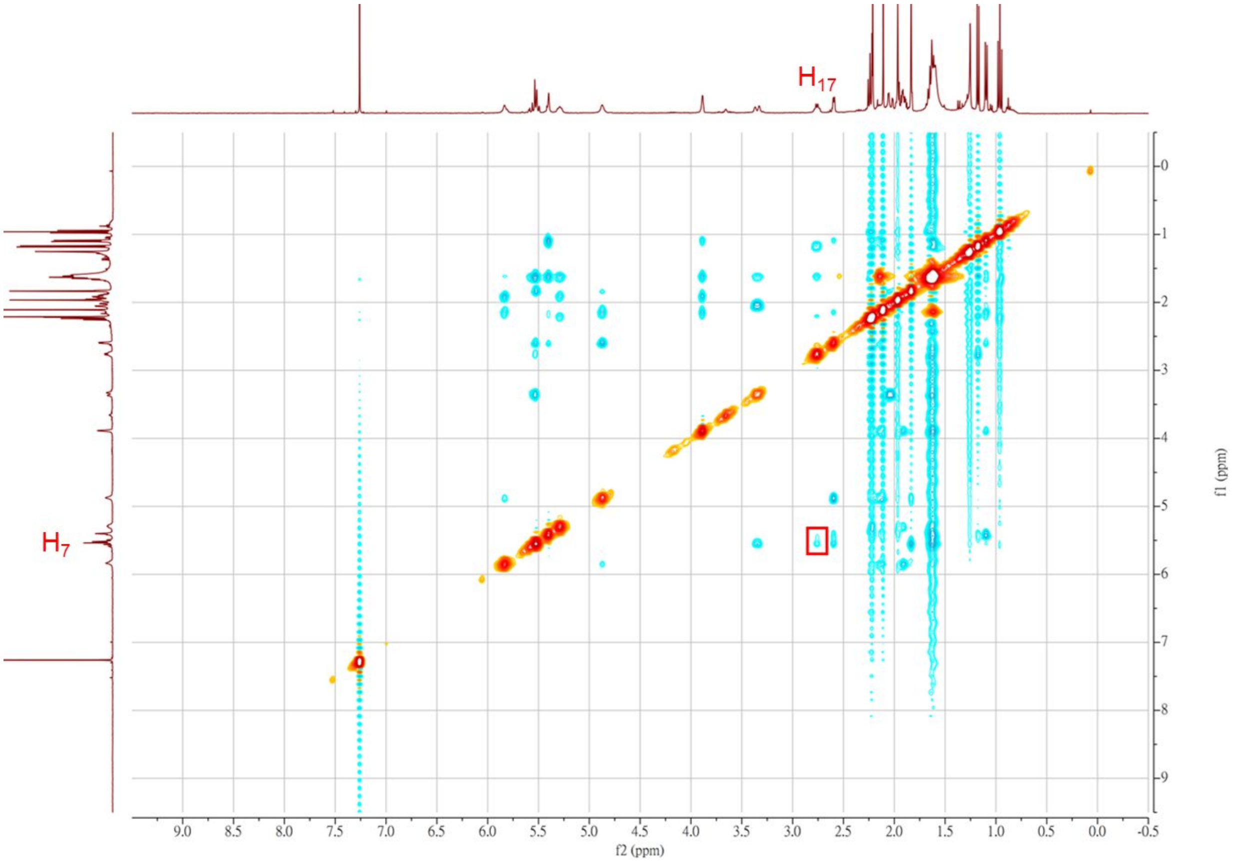

HPLC purity spectra of **GHN092**.

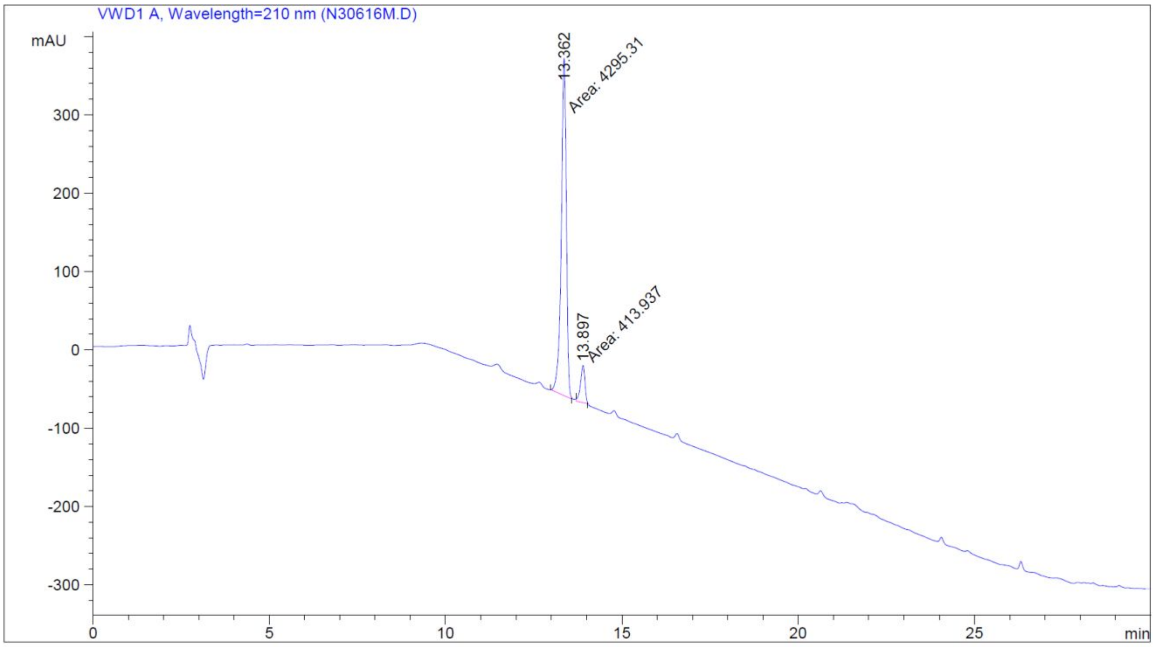


```
=====================================================================
                         Area Percent Report
=====================================================================

Sorted By             :      Signal
Multiplier            :      1.0000
Dilution              :      1.0000
Use Multiplier & Dilution Factor with ISTDs


Signal 1: VWD1 A, Wavelength=210 nm

Peak RetTime Type  Width     Area      Height     Area
  #   [min]        [min]  mAU   *s    [mAU   ]      %
----|-------|----|-------|----------|----------|--------|
   1  13.362 MM    0.1661 4295.30811  430.96713  91.2101
   2  13.897 MM    0.1455  413.93689   47.41761   8.7899

Totals :                  4709.24500  478.38474

 Results obtained with enhanced integrator!
=====================================================================
                          *** End of Report ***
```

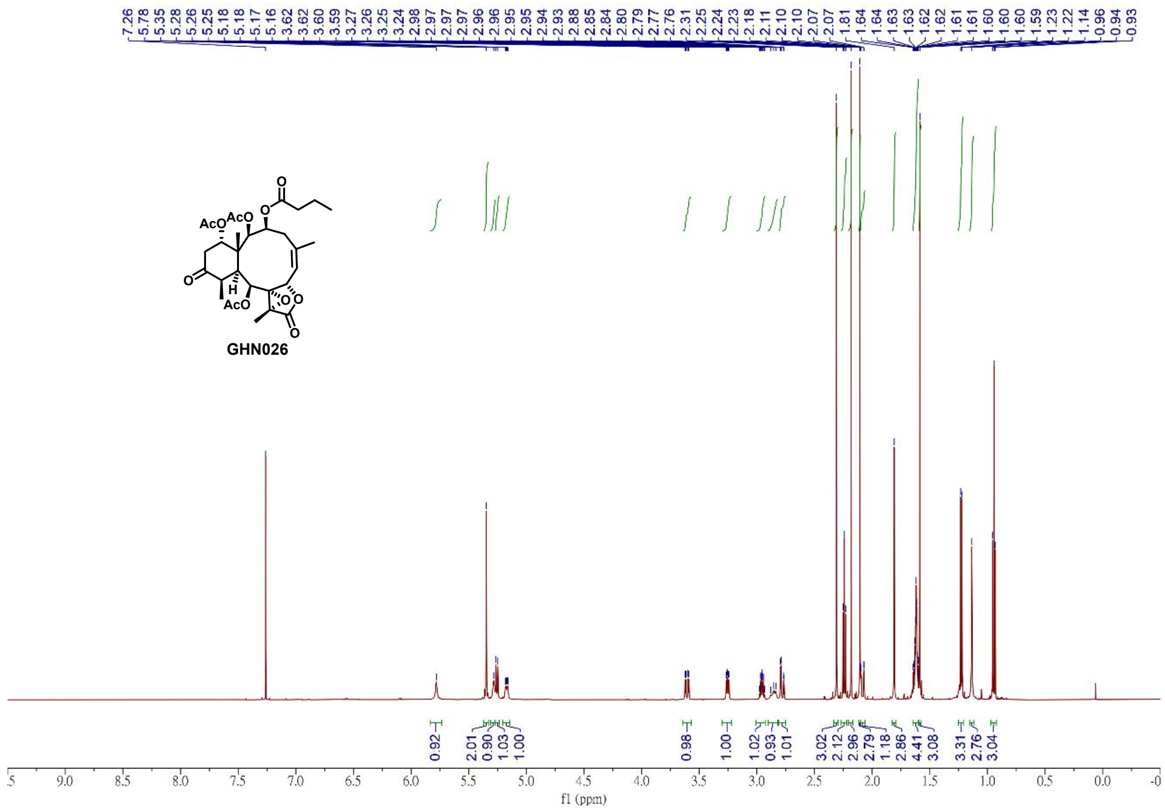

^{1}H NMR (600 MHz, $CDCl_3$, 22 ^{o}C) of **GHN026**.

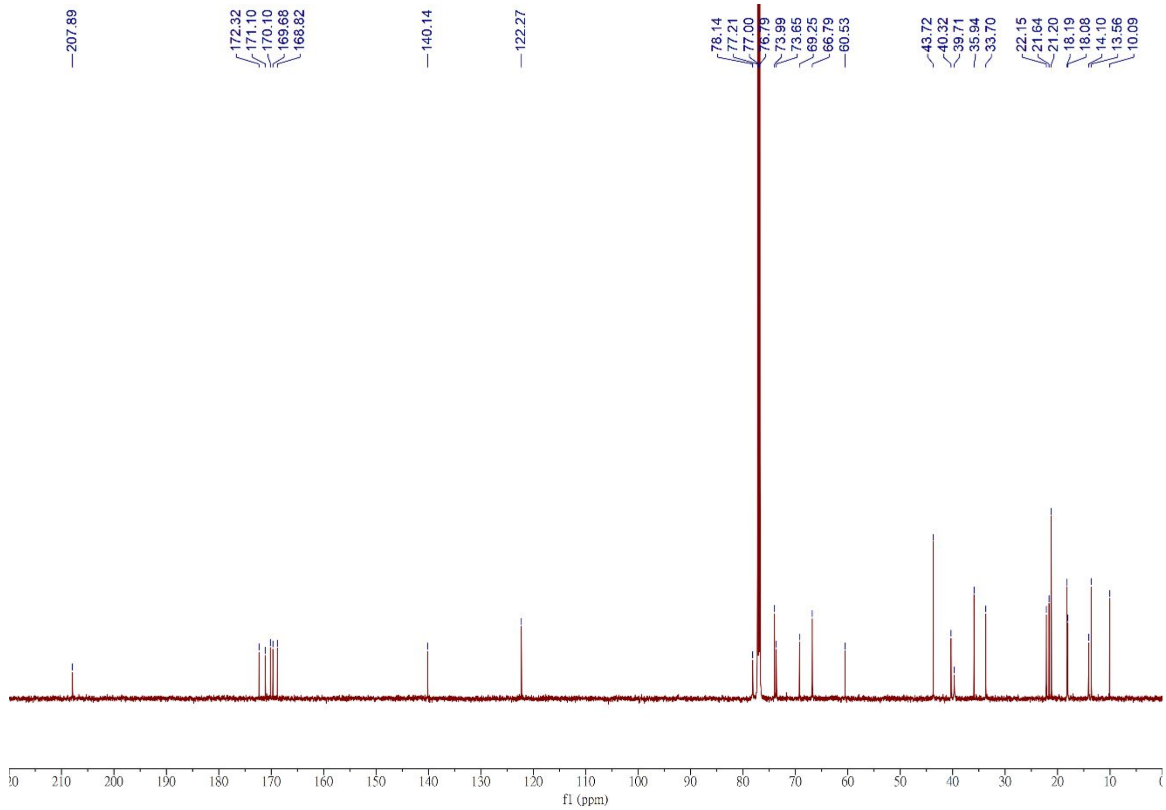

^{13}C NMR (151 MHz, $CDCl_3$, 22 ^{o}C) of **GHN026**.

HPLC purity spectra of **GHN026**.

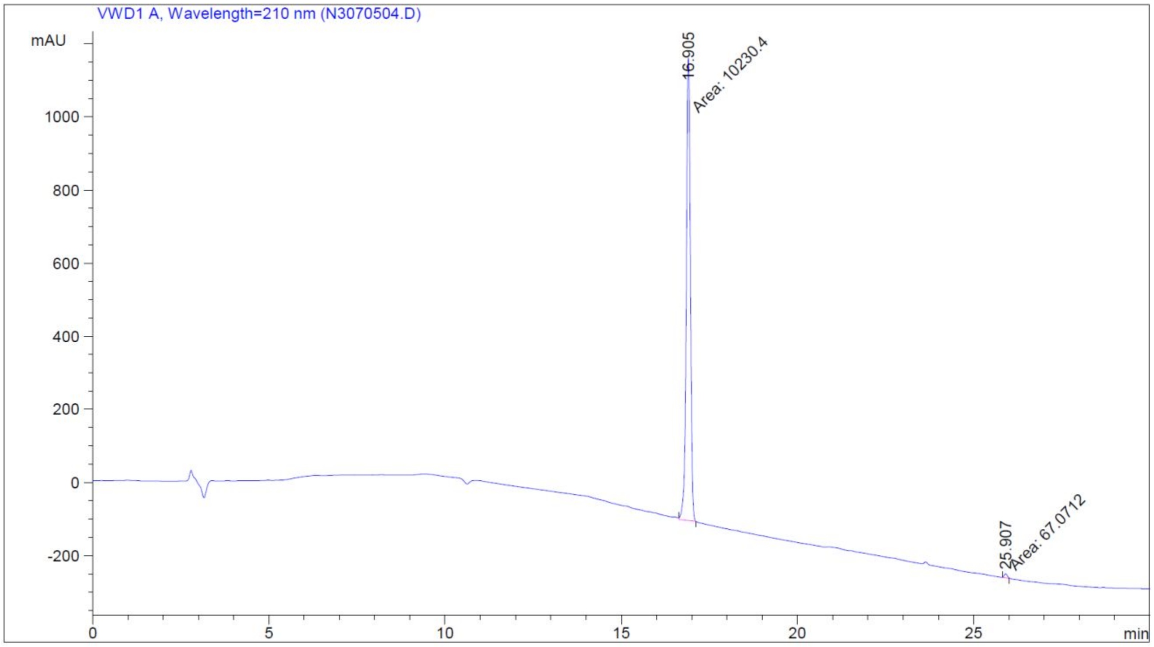


```
=====================================================================
                         Area Percent Report
=====================================================================

Sorted By             :      Signal
Multiplier            :      1.0000
Dilution              :      1.0000
Use Multiplier & Dilution Factor with ISTDs


Signal 1: VWD1 A, Wavelength=210 nm

Peak RetTime Type  Width     Area      Height     Area
  #   [min]        [min]  mAU    *s   [mAU   ]      %
----|-------|----|-------|----------|----------|--------|
   1  16.905 MM     0.1345 1.02304e4  1267.43604  99.3487
   2  25.907 MM     0.1030   67.07124   10.84898   0.6513

Totals :                  1.02974e4  1278.28501

 Results obtained with enhanced integrator!
=====================================================================
                          *** End of Report ***
```

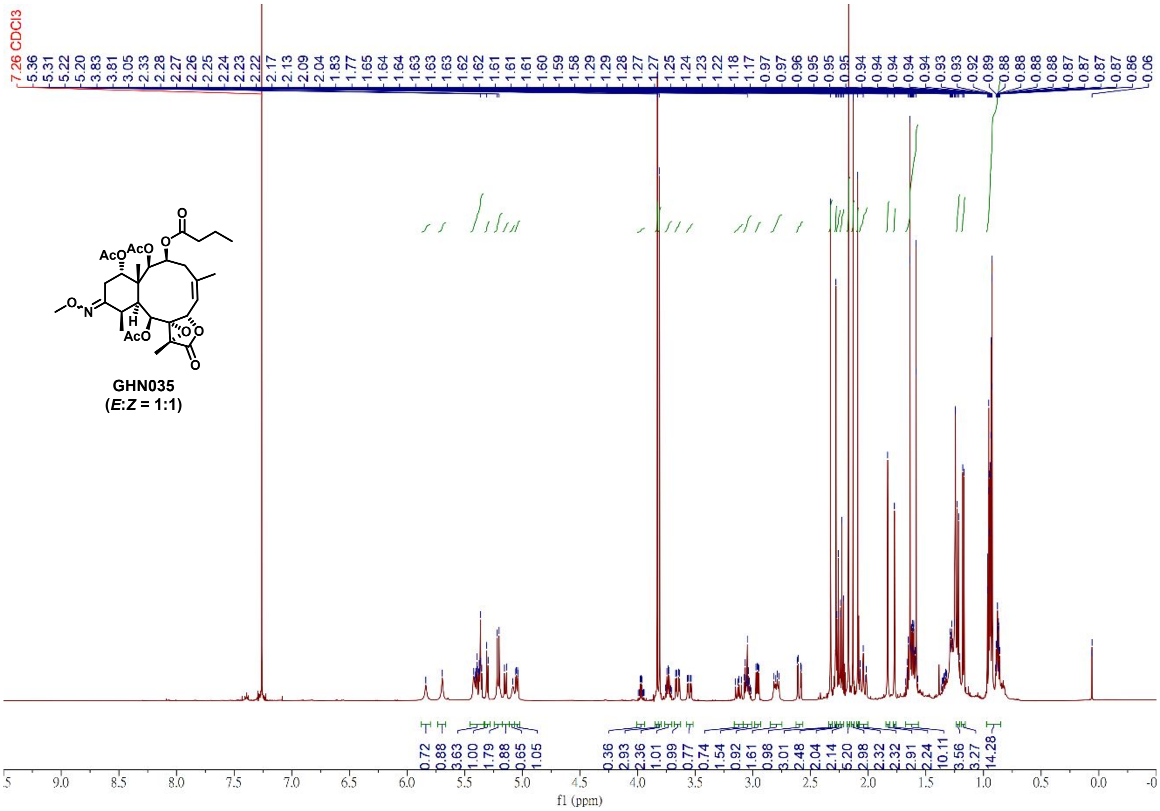

^{1}H NMR (600 MHz, $CDCl_3$, 22 ^{o}C) of **GHN035**.

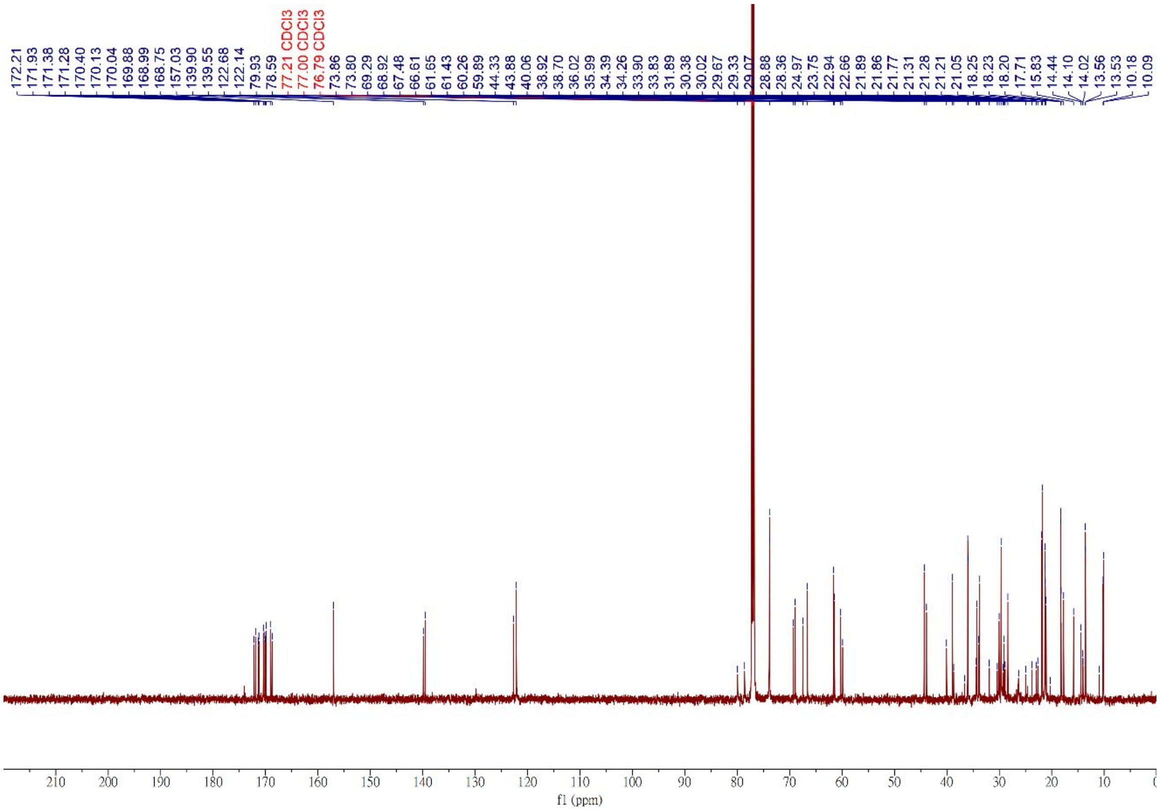

^{13}C NMR (151 MHz, $CDCl_3$, 22 ^{o}C) of **GHN035**.

HPLC purity spectra of **GHN035**.

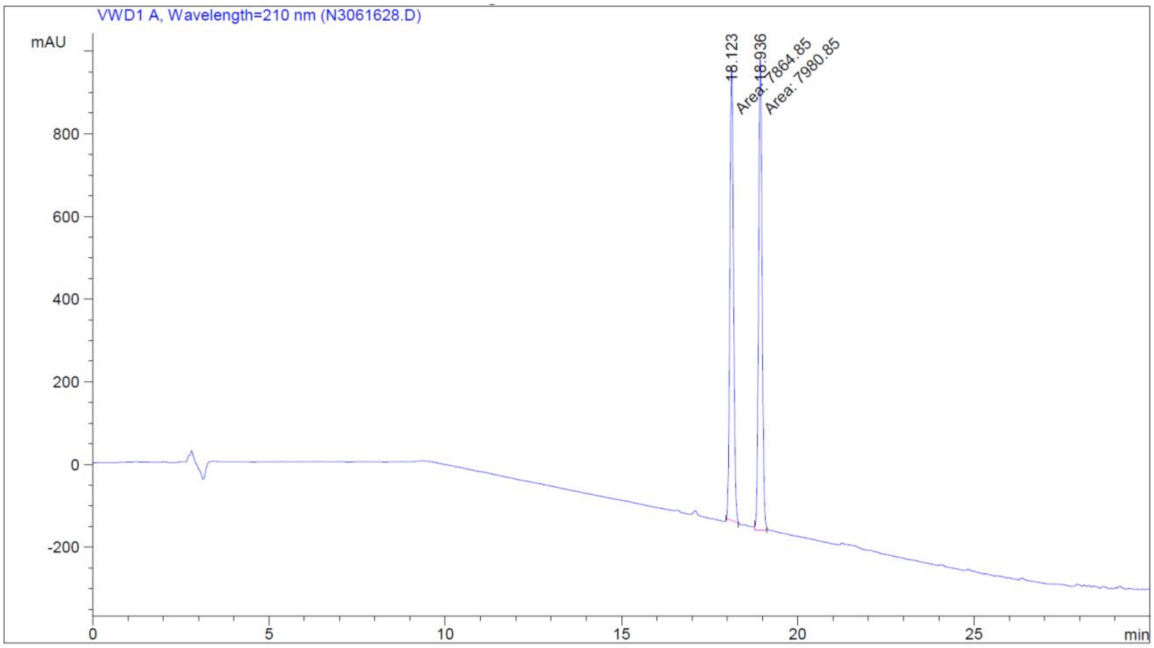


```
=====================================================================
                         Area Percent Report
=====================================================================

Sorted By             :      Signal
Multiplier            :      1.0000
Dilution              :      1.0000
Use Multiplier & Dilution Factor with ISTDs


Signal 1: VWD1 A, Wavelength=210 nm

Peak RetTime Type  Width     Area      Height     Area
  #   [min]        [min]  mAU   *s    [mAU   ]      %
----|-------|----|-------|----------|----------|--------|
   1  18.123 MM    0.1194 7864.85059 1097.55627  49.6340
   2  18.936 MM    0.1168 7980.85449 1138.49097  50.3660

Totals :                  1.58457e4  2236.04724

 Results obtained with enhanced integrator!
=====================================================================
                         *** End of Report ***
```

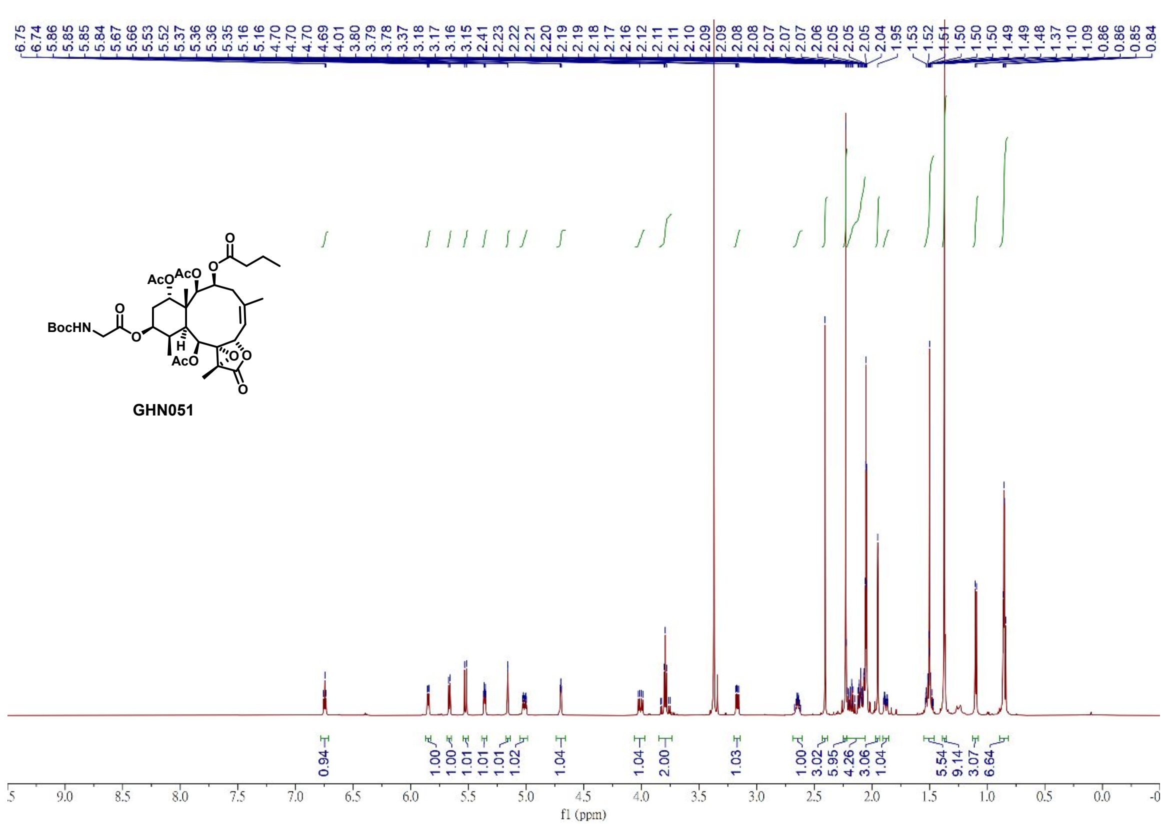

^{1}H NMR (600 MHz, $(CD_3)_2CO$, -40 ^{o}C) of **GHN051**.

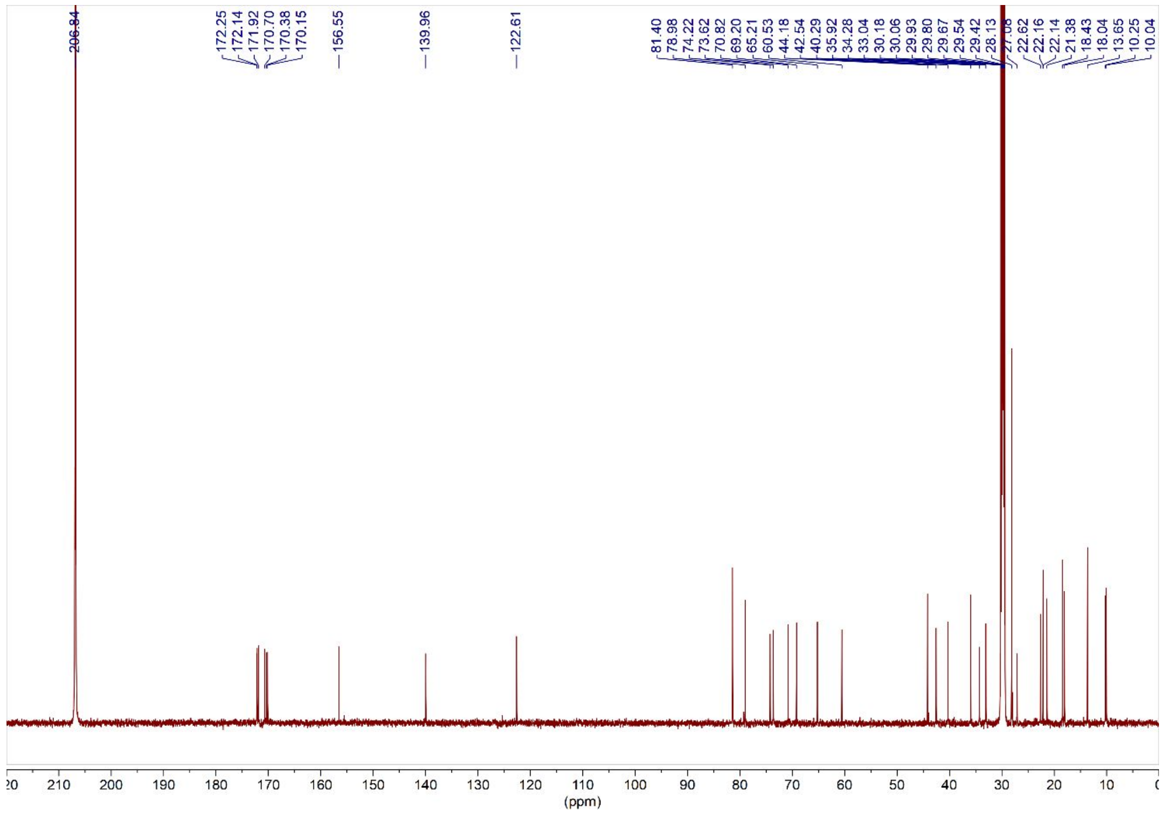

^{13}C NMR (151 MHz, $(CD_3)_2CO$, -40 ^{o}C) of **GHN051**.

HPLC purity spectra of **GHN051**.

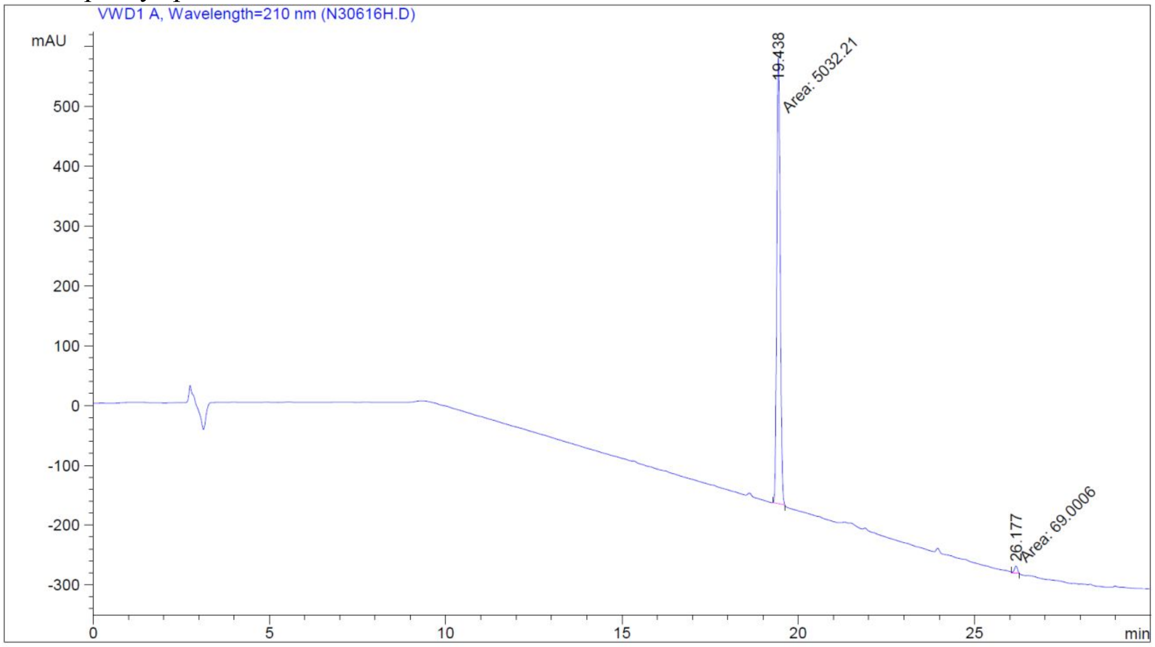


```
=====================================================================
                         Area Percent Report
=====================================================================

Sorted By             :      Signal
Multiplier            :      1.0000
Dilution              :      1.0000
Use Multiplier & Dilution Factor with ISTDs


Signal 1: VWD1 A, Wavelength=210 nm

Peak RetTime Type  Width     Area      Height     Area
  #   [min]        [min]  mAU   *s    [mAU   ]      %
----|-------|----|-------|----------|----------|--------|
   1  19.438 MM    0.1125 5032.21436  745.53186  98.6474
   2  26.177 MM    0.1006   69.00058   11.42586   1.3526

Totals :                  5101.21494  756.95772

 Results obtained with enhanced integrator!
=====================================================================
                         *** End of Report ***
```

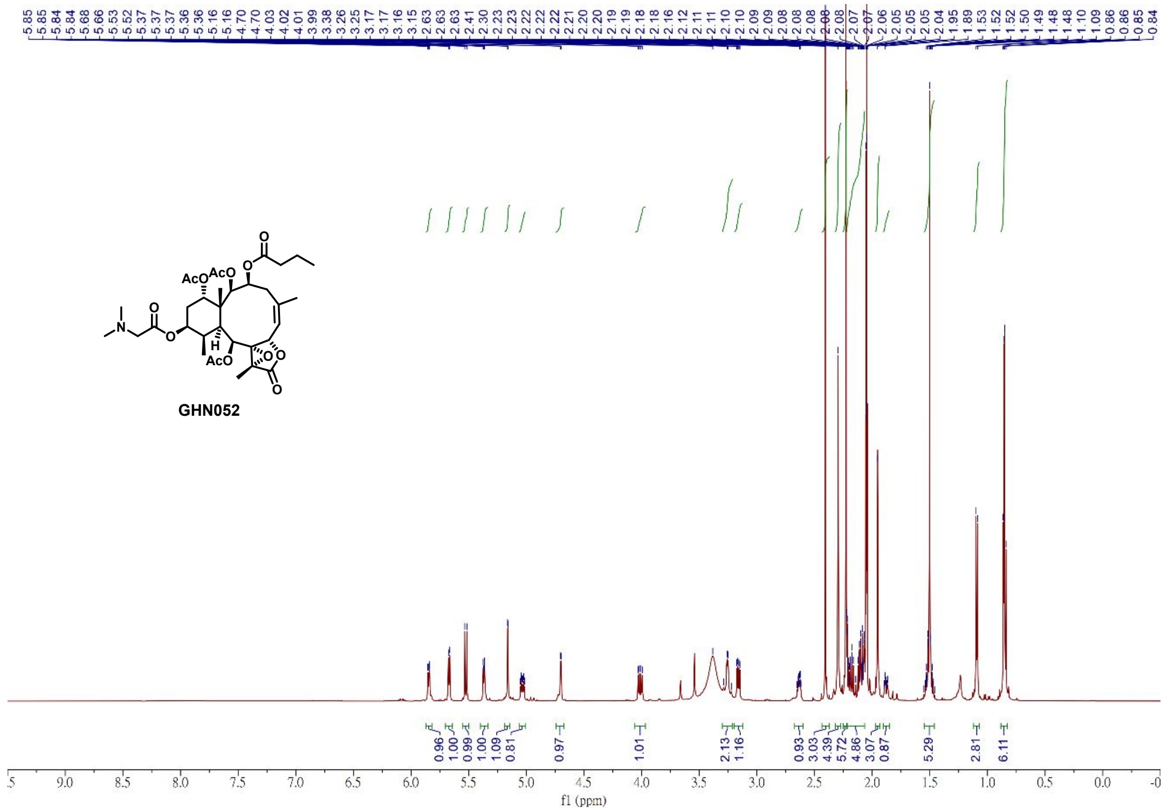

^{1}H NMR (600 MHz, $(CD_3)_2CO$, -40 °C) of **GHN052**.

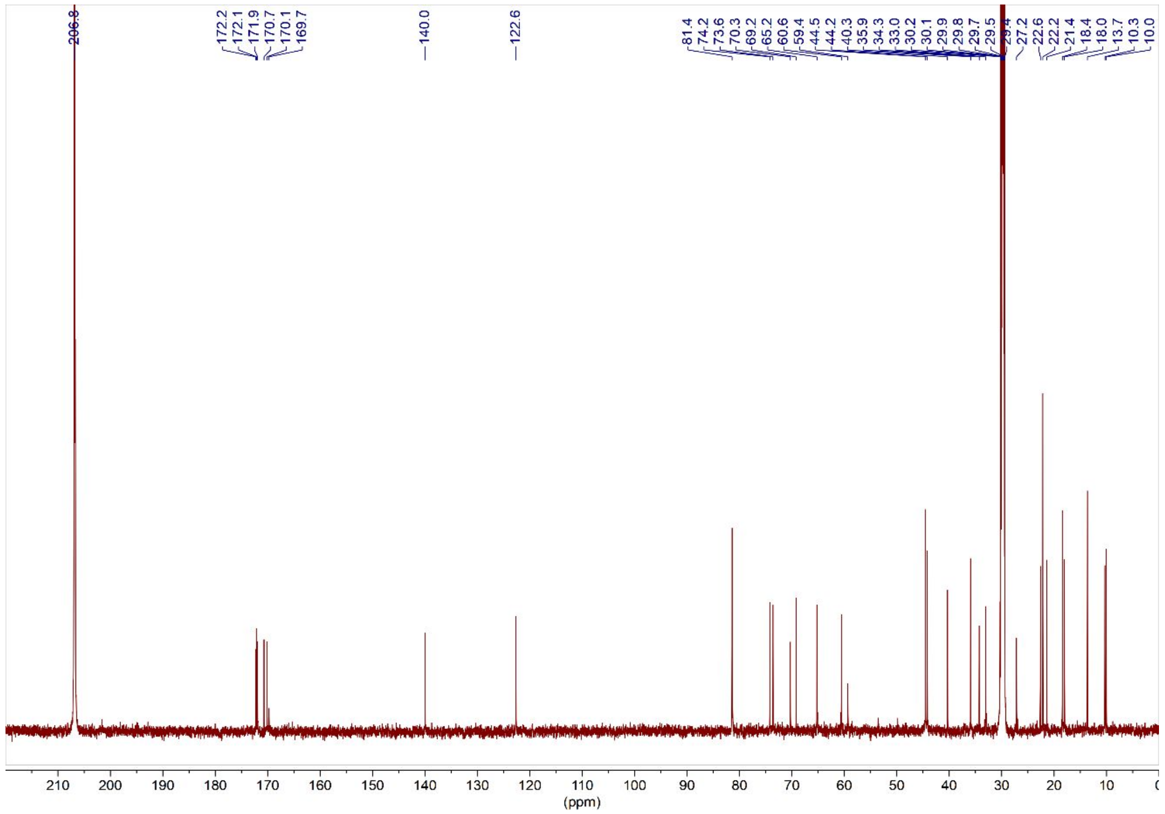

^{13}C NMR (151 MHz, $(CD_3)_2CO$, -40 °C) of **GHN052**.

HPLC purity spectra of **GHN052**.

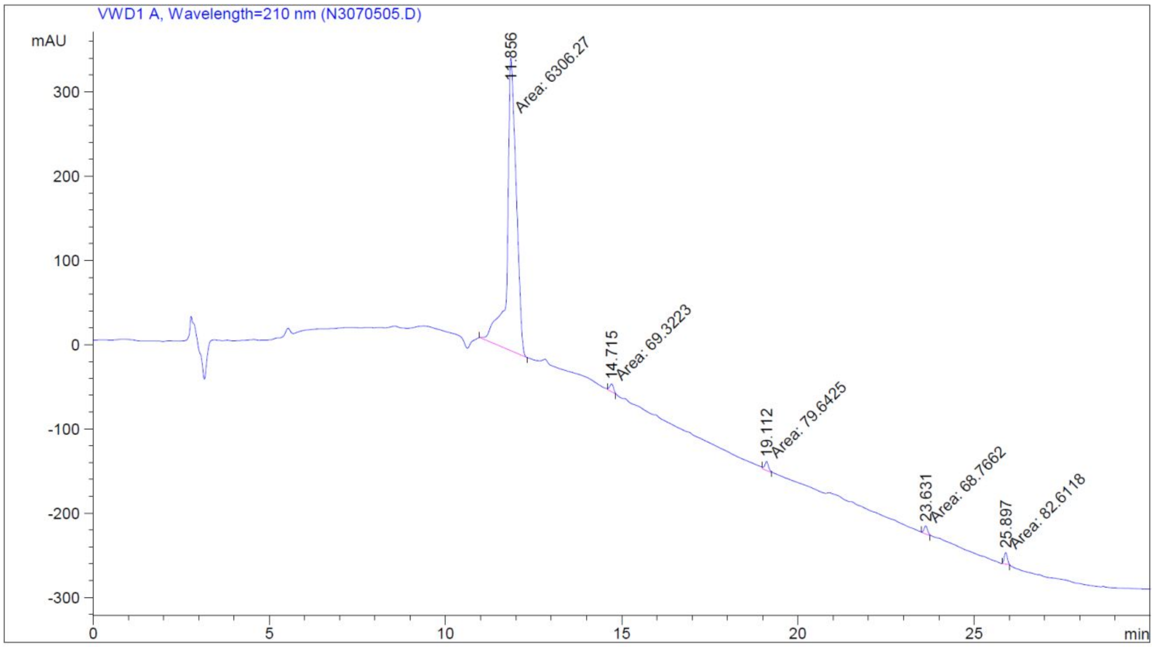

===

Area Percent Report

===

Sorted By : Signal
Multiplier : 1.0000
Dilution : 1.0000
Use Multiplier & Dilution Factor with ISTDs

Signal 1: VWD1 A, Wavelength=210 nm

Peak #	RetTime [min]	Type	Width [min]	Area mAU *s	Height [mAU]	Area %
1	11.856	MM	0.3026	6306.26758	347.34778	95.4539
2	14.715	MM	0.1244	69.32234	9.28501	1.0493
3	19.112	MM	0.1218	79.64246	10.89880	1.2055
4	23.631	MM	0.1181	68.76623	9.70643	1.0409
5	25.897	MM	0.1009	82.61181	13.64990	1.2504

Totals : 6606.61042 390.88792

Results obtained with enhanced integrator!

===

*** End of Report ***

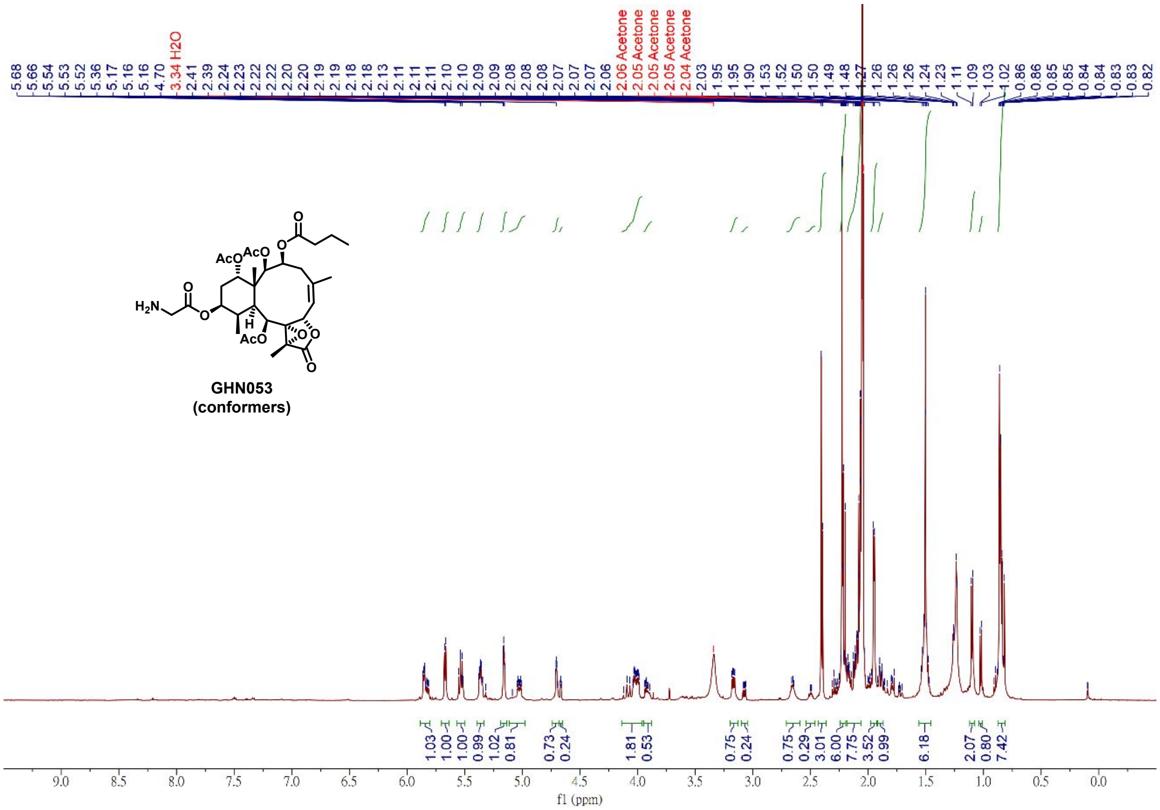

^{1}H NMR (600 MHz, $(CD_3)_2CO$, -40 ^{o}C) of **GHN053**.

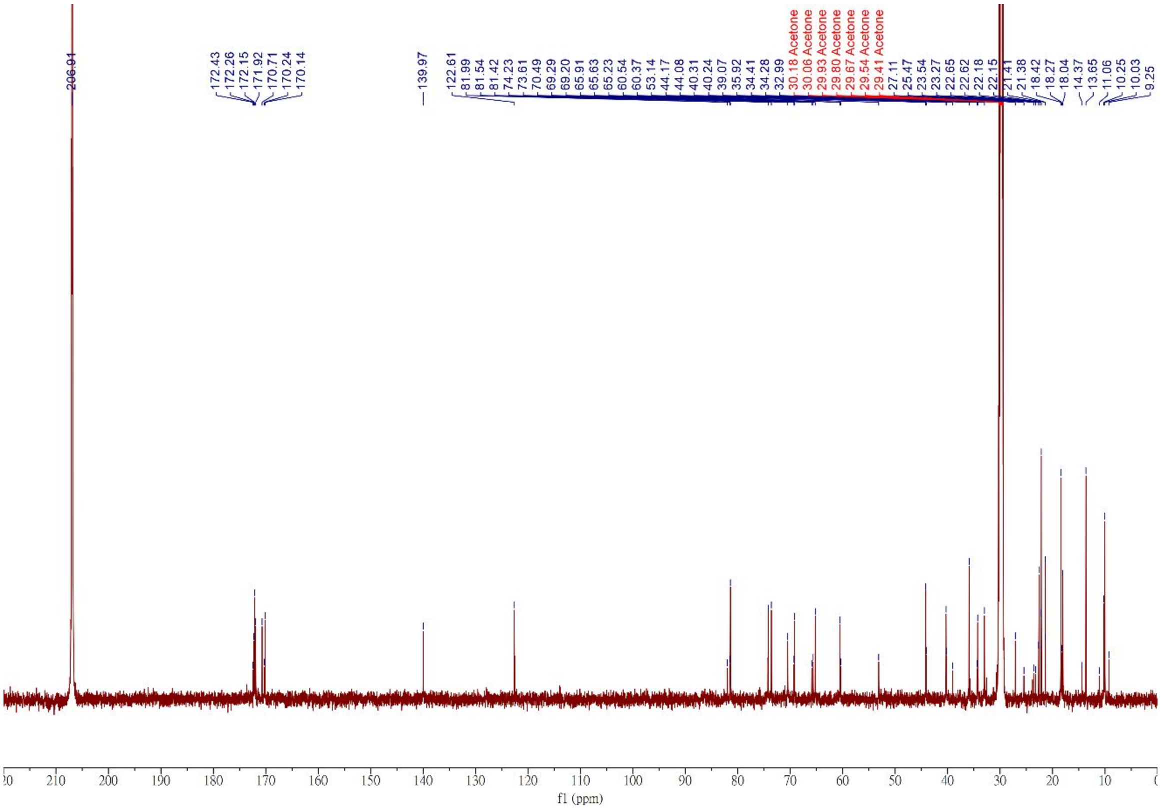

^{13}C NMR (151 MHz, $(CD_3)_2CO$, -40 ^{o}C) of **GHN053**.

HPLC purity spectra of **GHN053**. (λ = 210 nm)

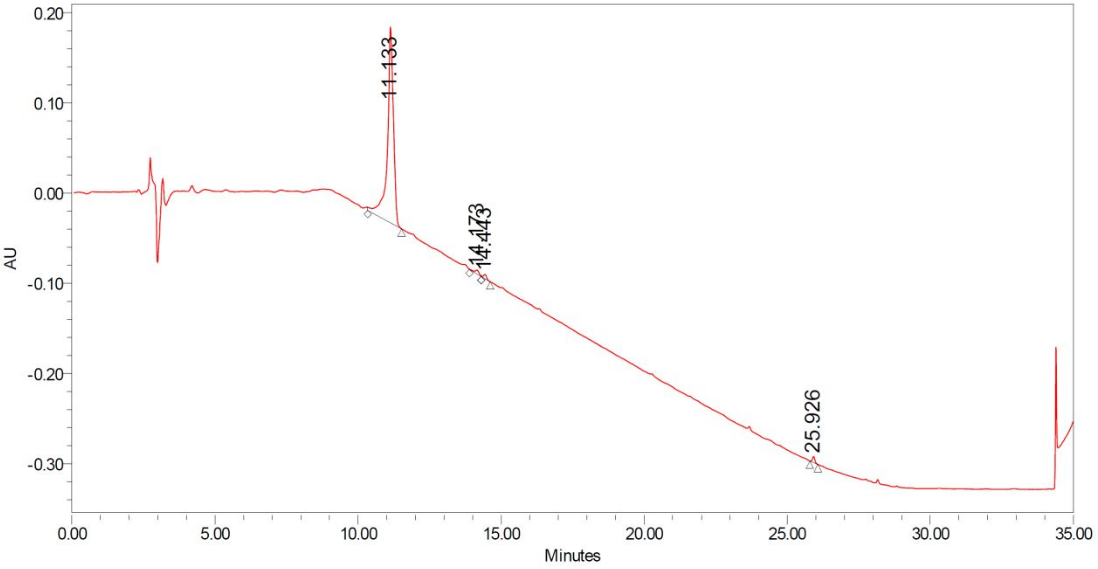

	RT	Area	% Area	Height
1	11.133	3193478	96.36	217042
2	14.173	45407	1.37	4491
3	14.443	35957	1.08	4773
4	25.926	39289	1.19	6773

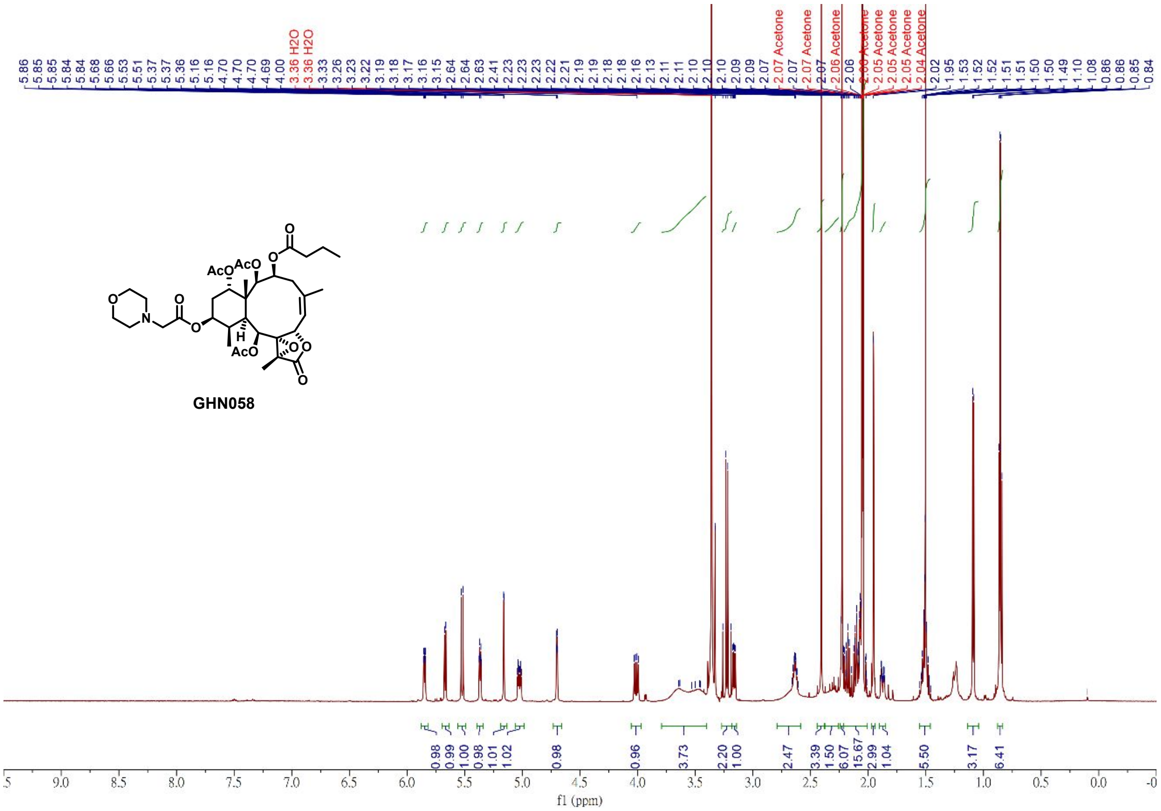

^{1}H NMR (600 MHz, $(CD_3)_2CO$, -40 °C) of **GHN058**.

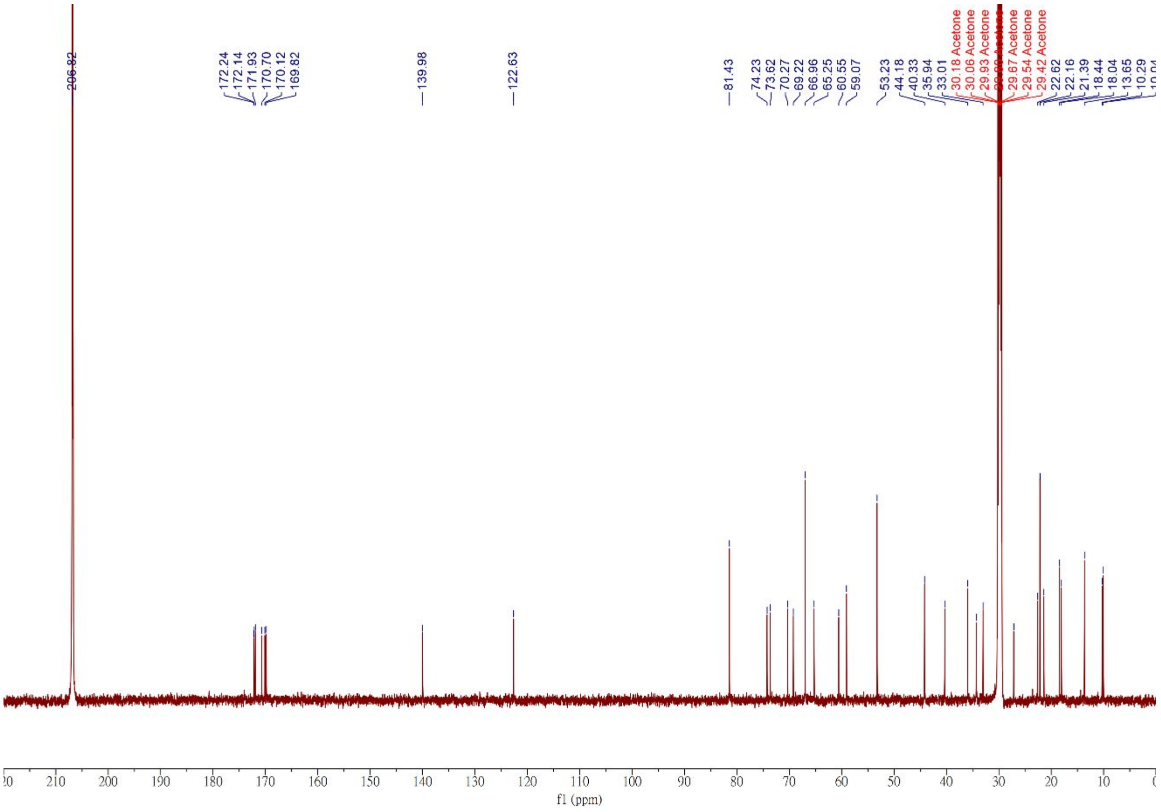

^{13}C NMR (151 MHz, $(CD_3)_2CO$, -40 °C) of **GHN058**.

HPLC purity spectra of **GHN058**.

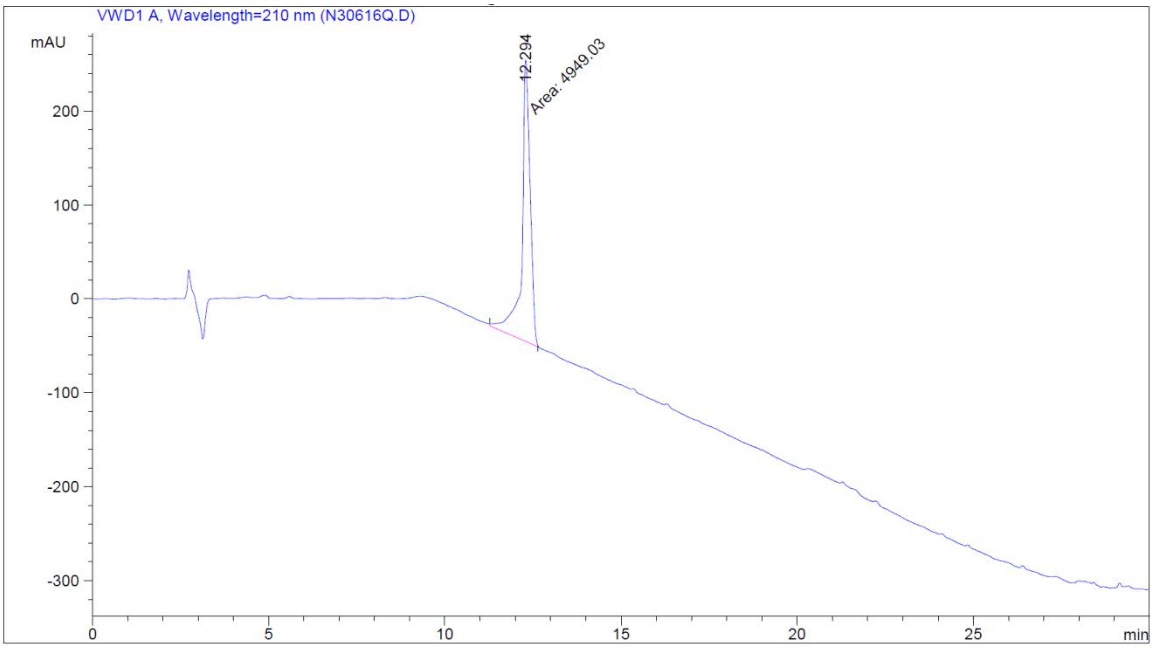


```
=====================================================================
                         Area Percent Report
=====================================================================

Sorted By             :      Signal
Multiplier            :      1.0000
Dilution              :      1.0000
Use Multiplier & Dilution Factor with ISTDs


Signal 1: VWD1 A, Wavelength=210 nm

Peak RetTime Type  Width     Area      Height     Area
  #   [min]        [min]  mAU    *s   [mAU    ]     %
----|-------|----|-------|----------|----------|--------|
   1  12.294 MM    0.2750 4949.02979  299.97275 100.0000

Totals :                  4949.02979  299.97275

 Results obtained with enhanced integrator!
=====================================================================
                          *** End of Report ***
```

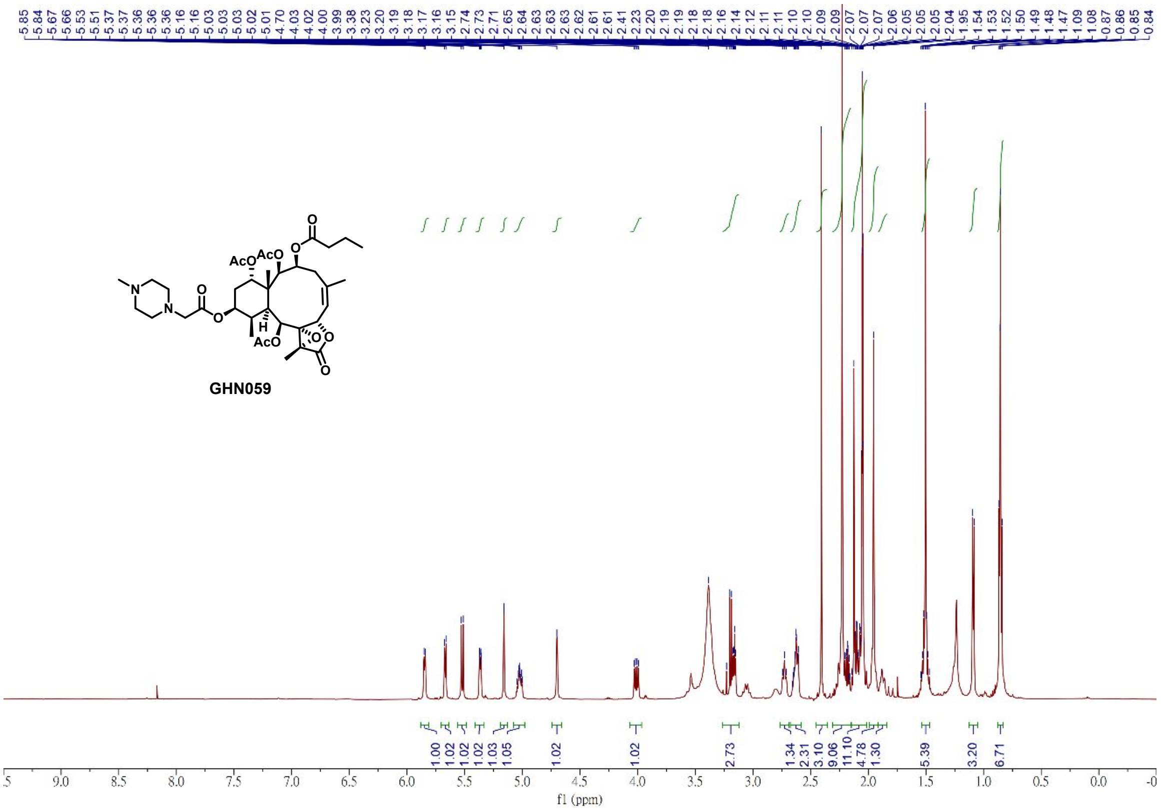

^{1}H NMR (600 MHz, $(CD_3)_2CO$, -40 ^{o}C) of **GHN059**.

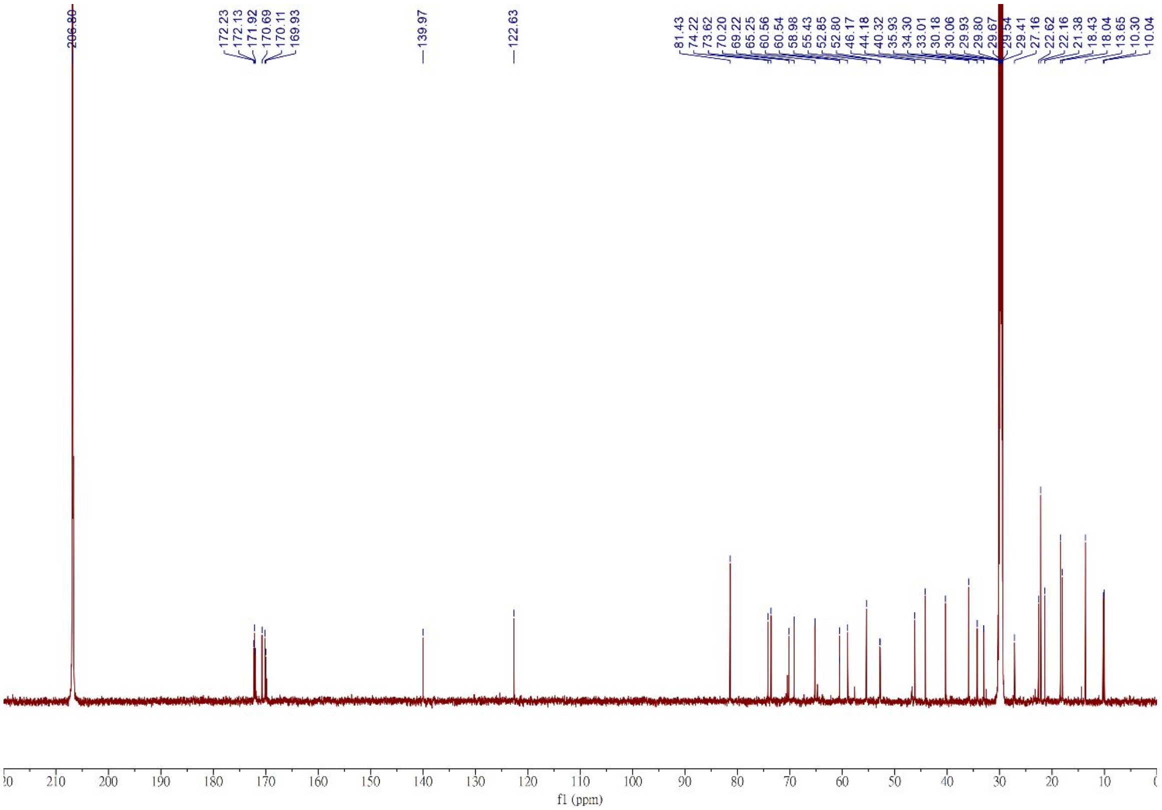

^{13}C NMR (151 MHz, $(CD_3)_2CO$, -40 ^{o}C) of **GHN059**.

HPLC purity spectra of **GHN059**.

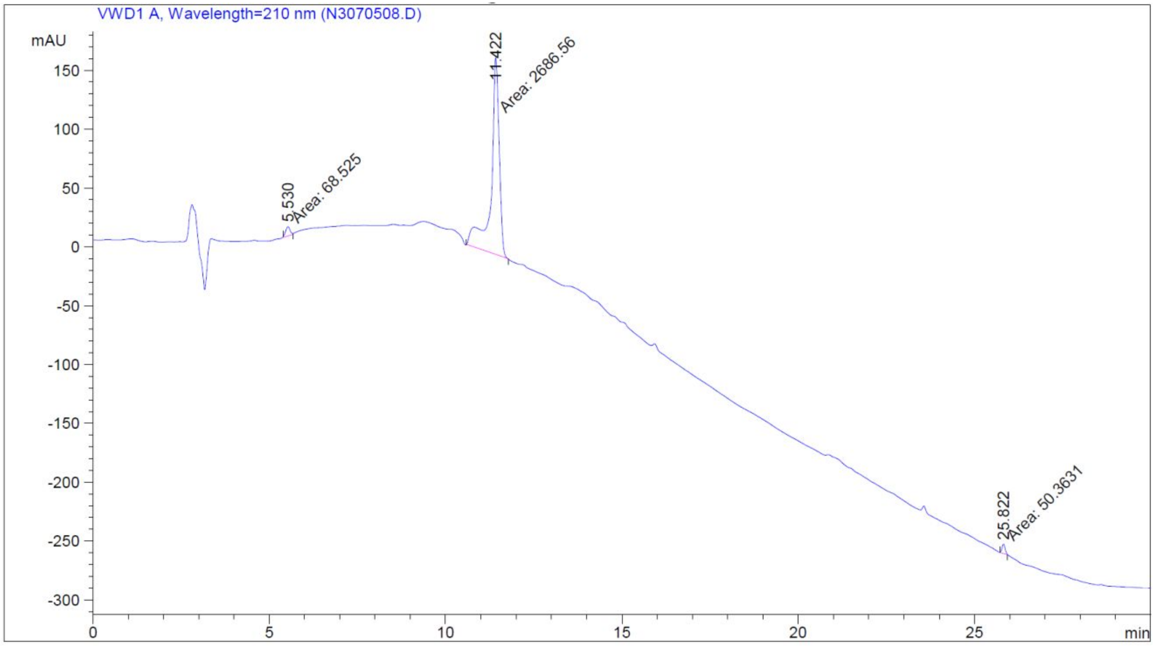


```
=====================================================================
                         Area Percent Report
=====================================================================

Sorted By             :      Signal
Multiplier            :      1.0000
Dilution              :      1.0000
Use Multiplier & Dilution Factor with ISTDs


Signal 1: VWD1 A, Wavelength=210 nm

Peak RetTime Type  Width     Area      Height     Area
  #   [min]        [min]  mAU   *s    [mAU   ]      %
----|-------|----|-------|----------|----------|--------|
   1   5.530 MM    0.1449   68.52496    7.88432   2.4426
   2  11.422 MM    0.2682 2686.55518  166.93108  95.7622
   3  25.822 MM    0.1038   50.36307    8.08378   1.7952

Totals :                  2805.44320  182.89917

 Results obtained with enhanced integrator!
=====================================================================
                          *** End of Report ***
```

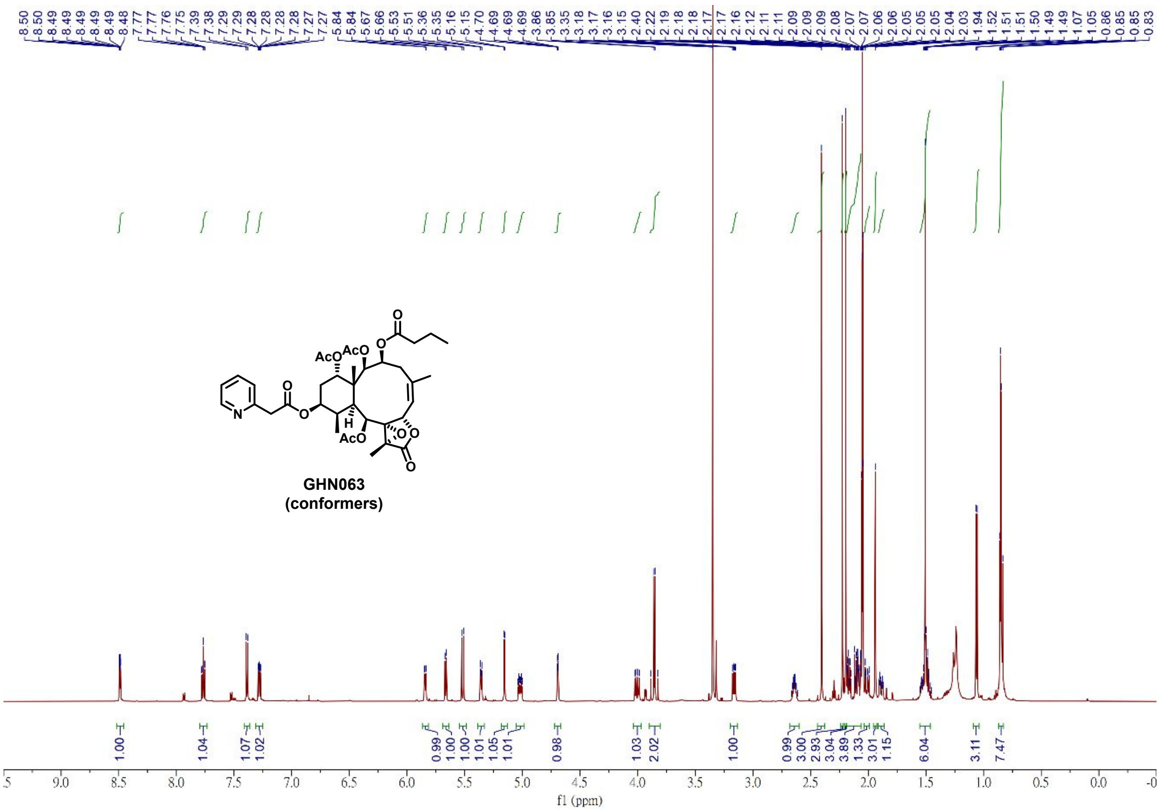

^{1}H NMR (600 MHz, $(CD_3)_2CO$, -40 ^{o}C) of **GHN063**.

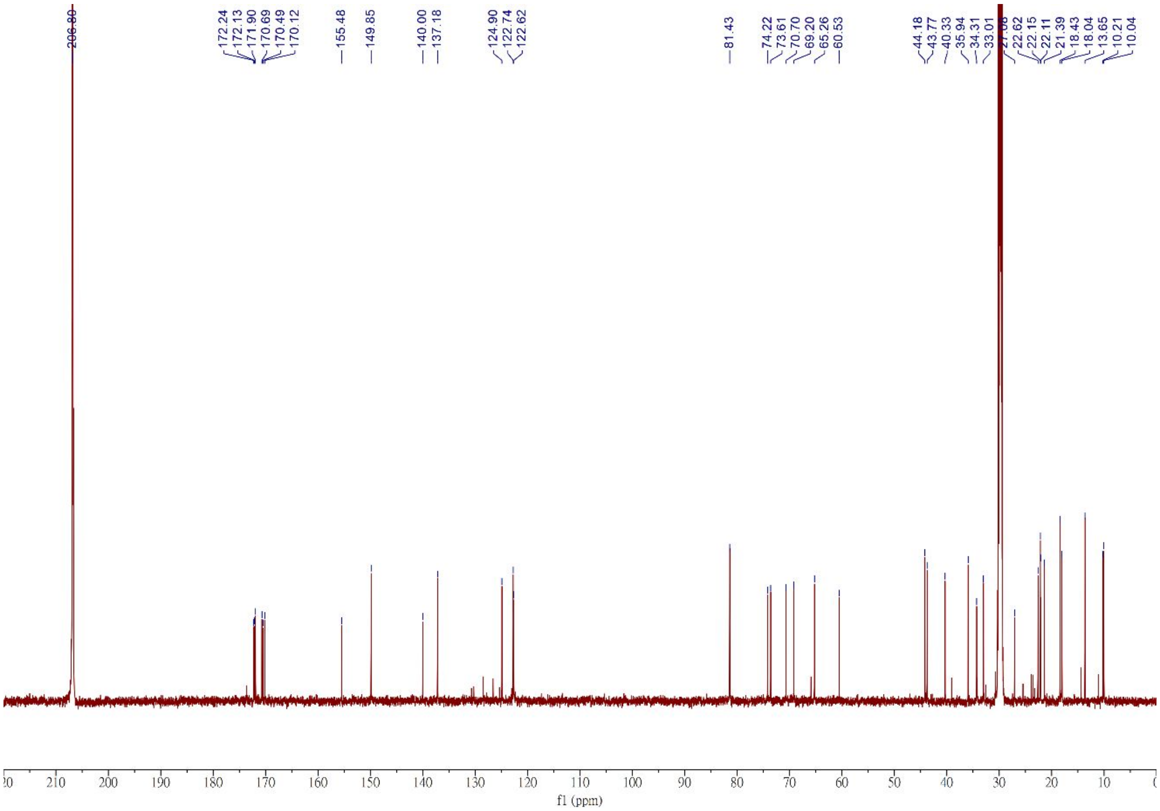

^{13}C NMR (151 MHz, $(CD_3)_2CO$, -40 ^{o}C) of **GHN063**.

HPLC purity spectra of **GHN063**.

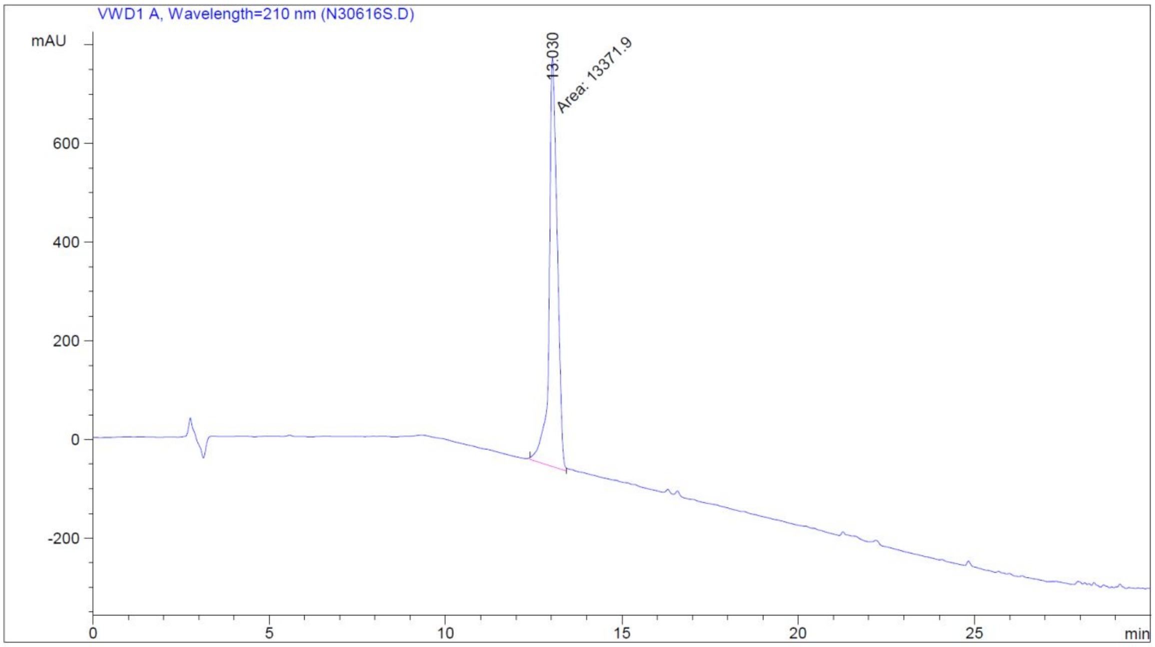


```
=====================================================================
                         Area Percent Report
=====================================================================

Sorted By             :      Signal
Multiplier            :      1.0000
Dilution              :      1.0000
Use Multiplier & Dilution Factor with ISTDs


Signal 1: VWD1 A, Wavelength=210 nm

Peak RetTime Type  Width     Area      Height     Area
  #   [min]        [min]  mAU    *s   [mAU   ]      %
----|-------|----|-------|----------|----------|--------|
   1  13.030 MM    0.2691 1.33719e4  828.24908 100.0000

Totals :                  1.33719e4  828.24908

 Results obtained with enhanced integrator!
=====================================================================
                          *** End of Report ***
```

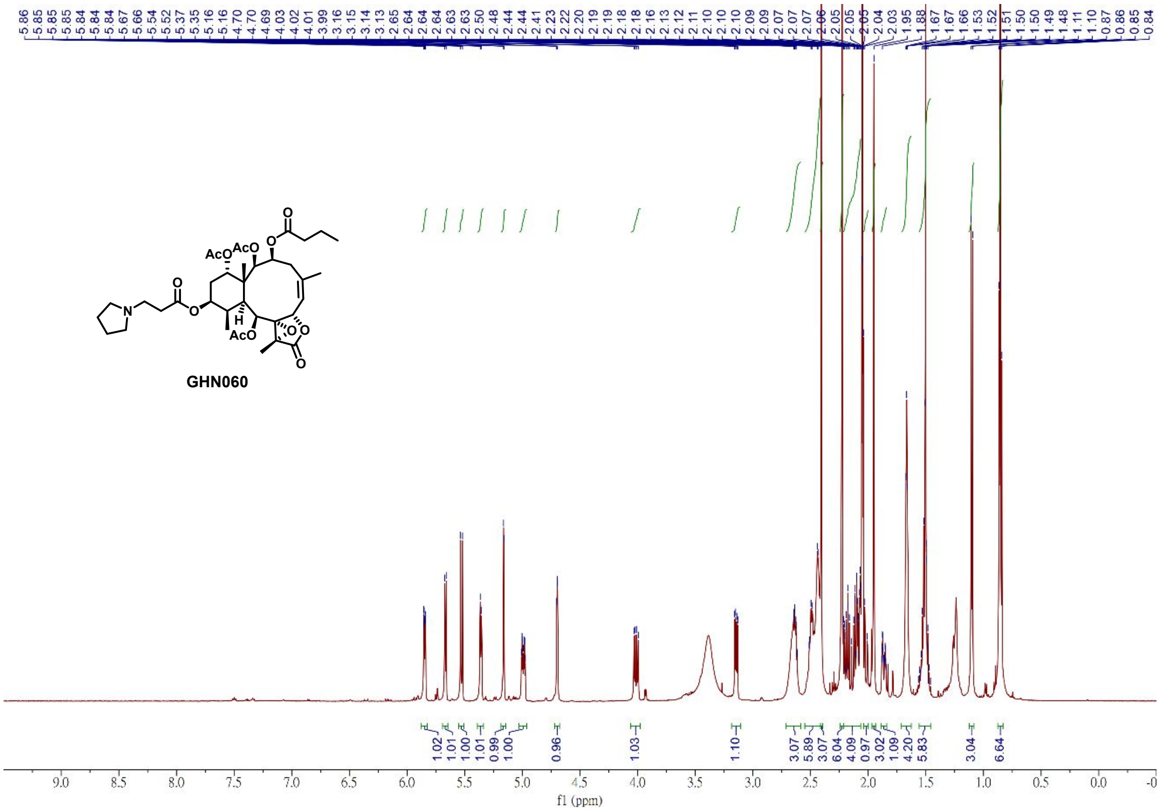

^{1}H NMR (600 MHz, $(CD_3)_2CO$, -40 ^{o}C) of **GHN060**.

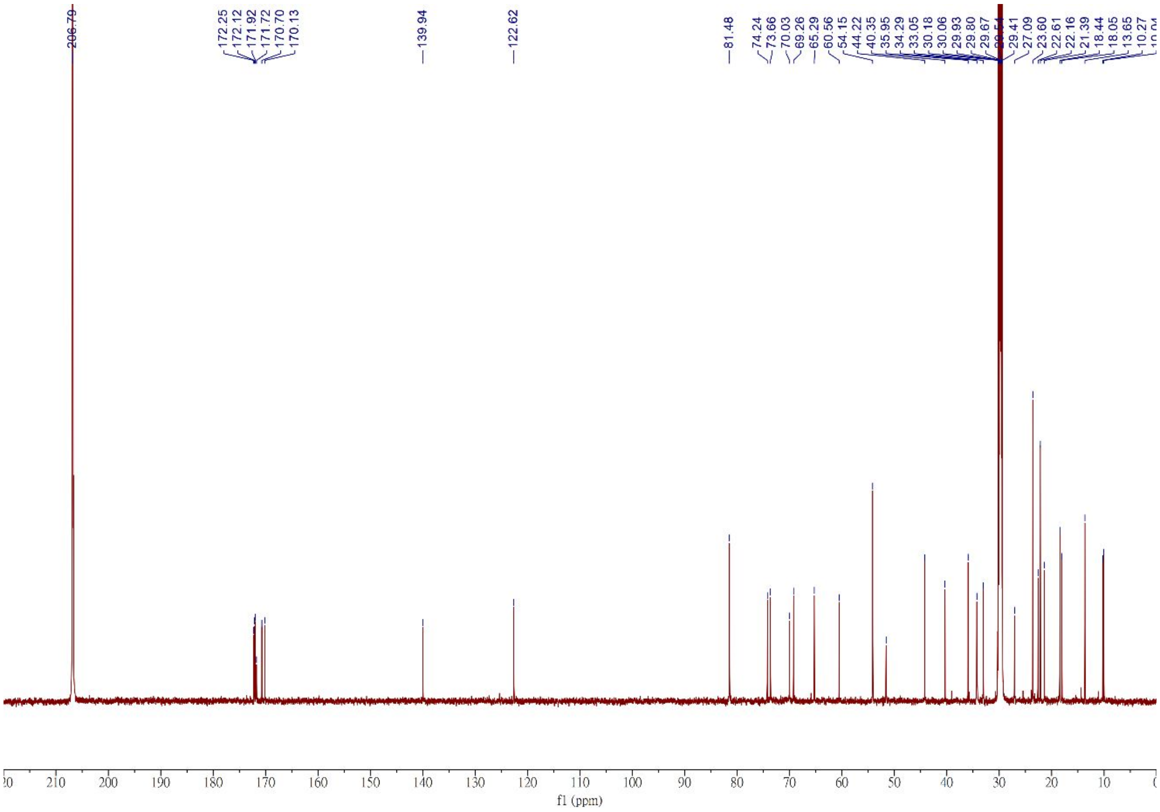

^{13}C NMR (151 MHz, $(CD_3)_2CO$, -40 ^{o}C) of **GHN060**.

HPLC purity spectra of **GHN060**.

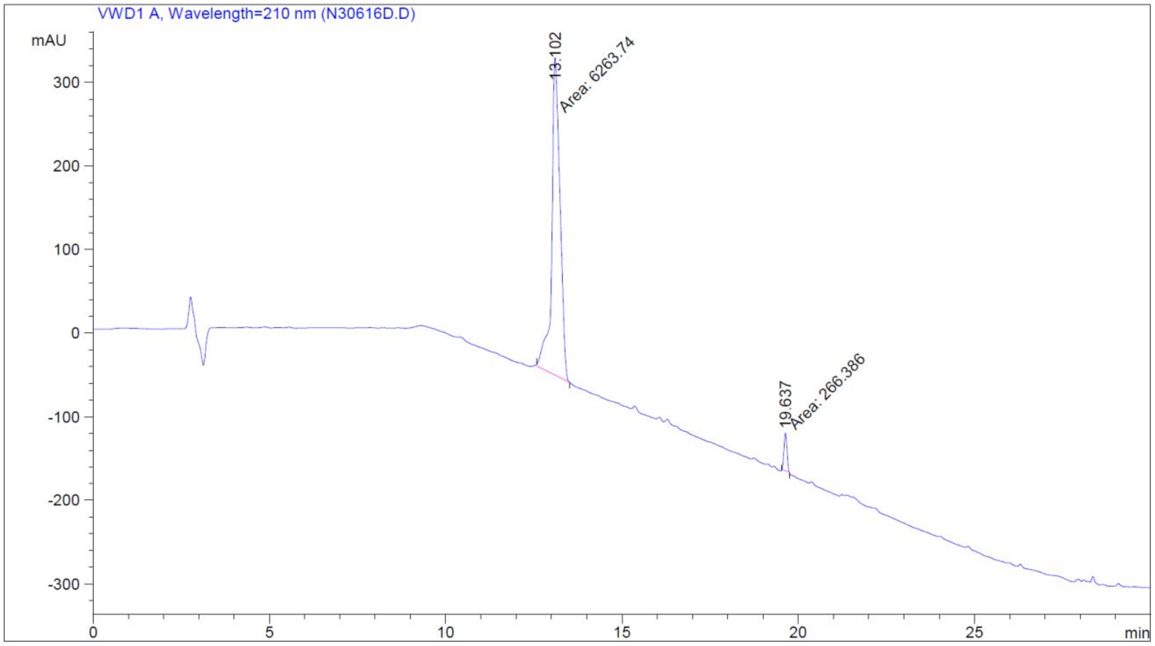


```
=====================================================================
                         Area Percent Report
=====================================================================

Sorted By             :      Signal
Multiplier            :      1.0000
Dilution              :      1.0000
Use Multiplier & Dilution Factor with ISTDs


Signal 1: VWD1 A, Wavelength=210 nm

Peak RetTime Type  Width     Area      Height     Area
  #   [min]        [min]  mAU    *s   [mAU    ]      %
----|-------|----|-------|----------|----------|--------|
   1  13.102 MM    0.2748 6263.74414  379.96088  95.9207
   2  19.637 MM    0.0987  266.38553   44.98750   4.0793

Totals :                  6530.12967  424.94838

 Results obtained with enhanced integrator!
=====================================================================
                          *** End of Report ***
```

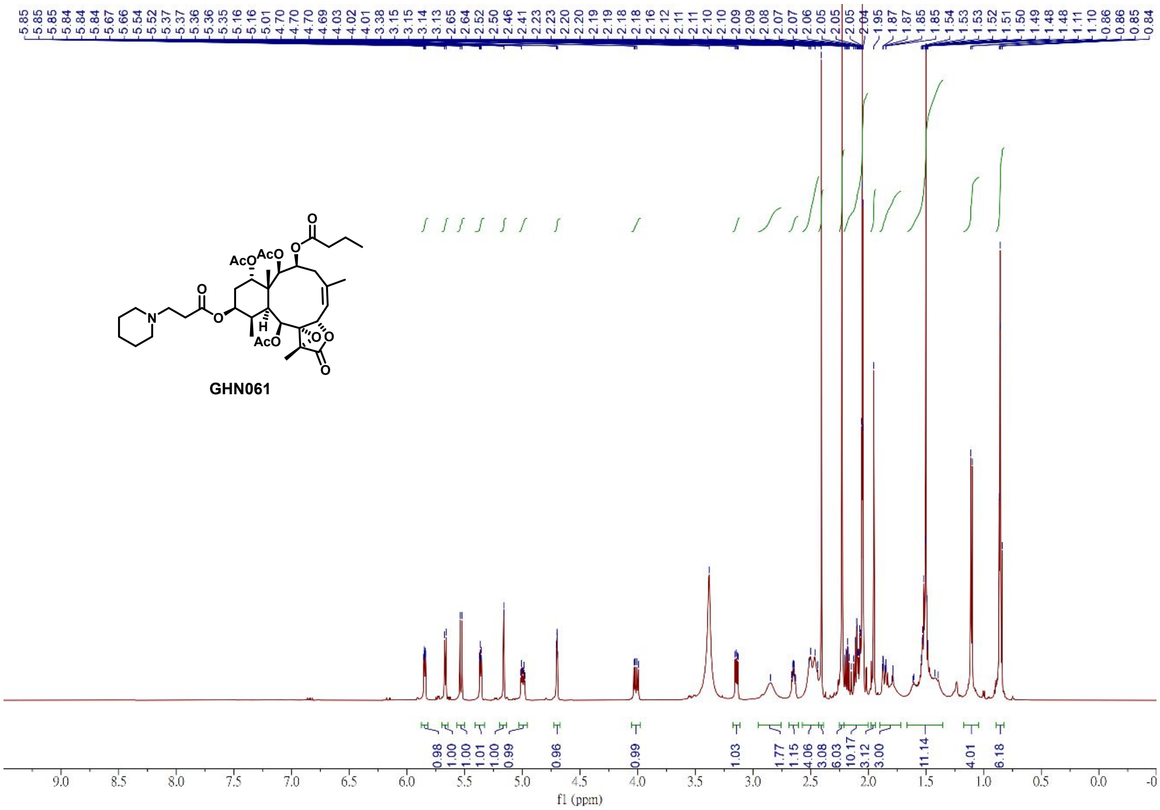

^1H NMR (600 MHz, $(CD_3)_2CO$, -40 °C) of **GHN061**.

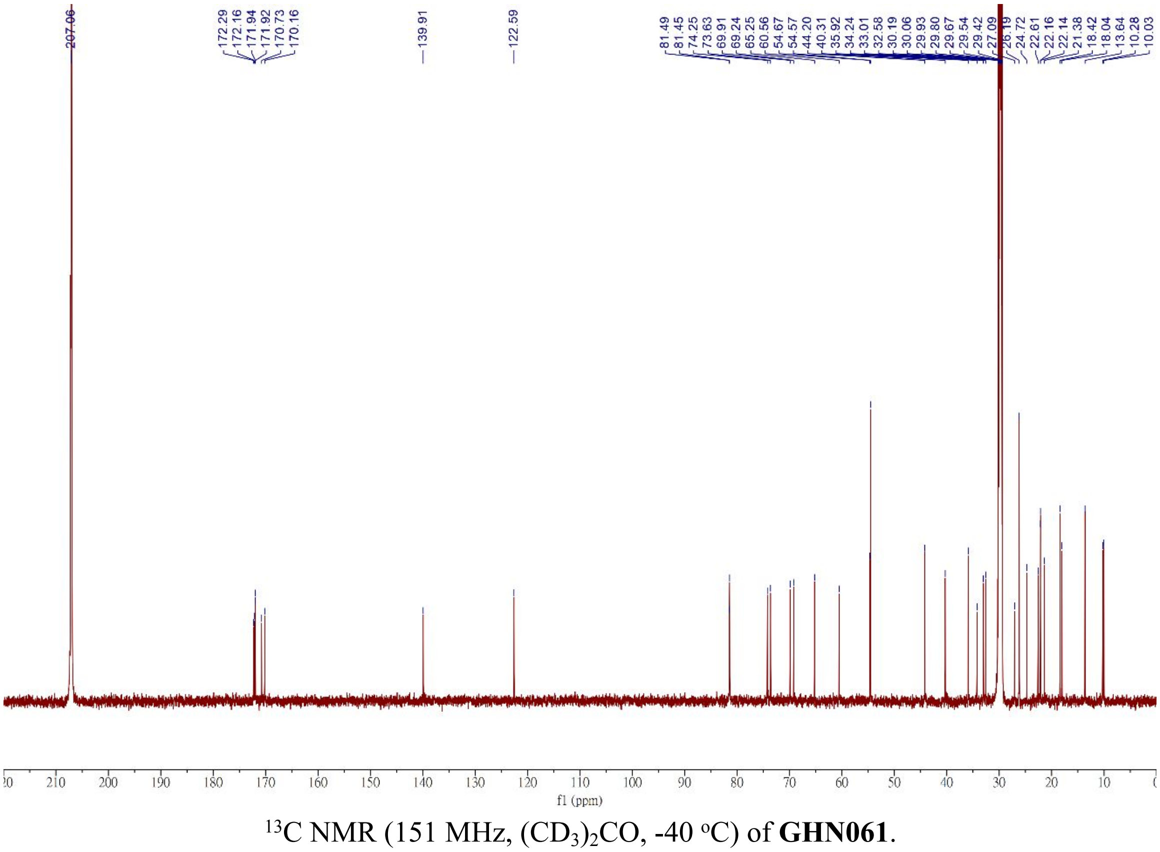

^{13}C NMR (151 MHz, $(CD_3)_2CO$, -40 °C) of **GHN061**.

HPLC purity spectra of **GHN061**.

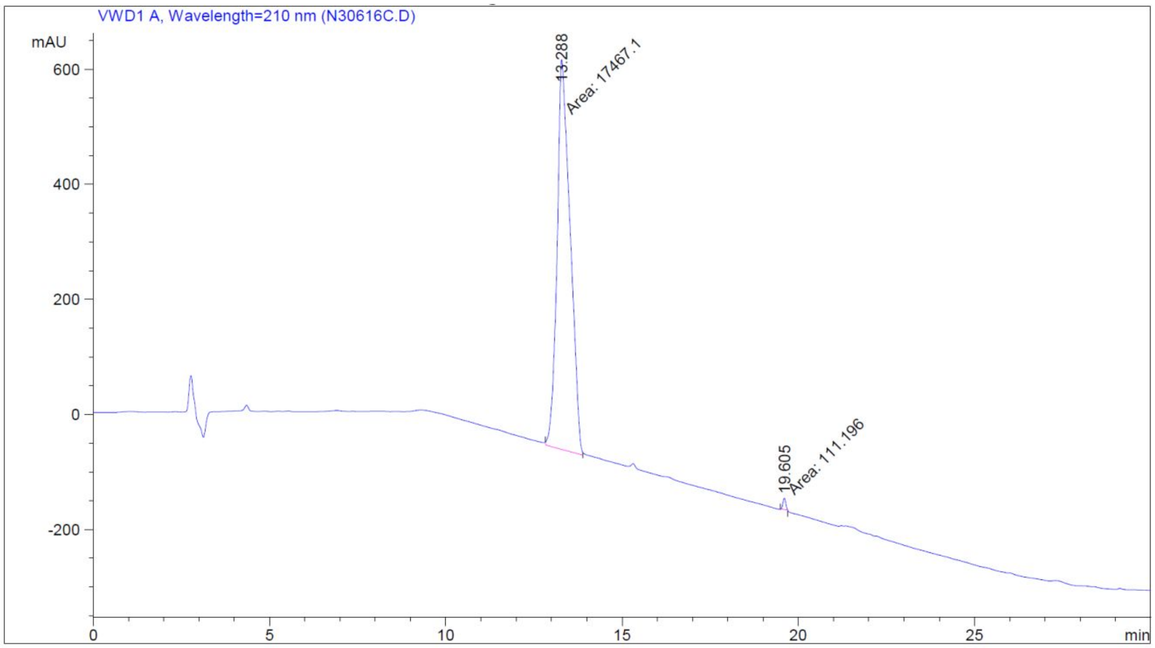


```
=====================================================================
                         Area Percent Report
=====================================================================

Sorted By             :      Signal
Multiplier            :      1.0000
Dilution              :      1.0000
Use Multiplier & Dilution Factor with ISTDs


Signal 1: VWD1 A, Wavelength=210 nm

Peak RetTime Type  Width     Area      Height     Area
  #   [min]        [min]  mAU    *s   [mAU   ]      %
----|-------|----|-------|----------|----------|--------|
   1  13.288 MM    0.4298 1.74671e4   677.26672  99.3674
   2  19.605 MM    0.0939  111.19569   19.72701   0.6326

Totals :                  1.75783e4   696.99373

 Results obtained with enhanced integrator!
=====================================================================
                         *** End of Report ***
```

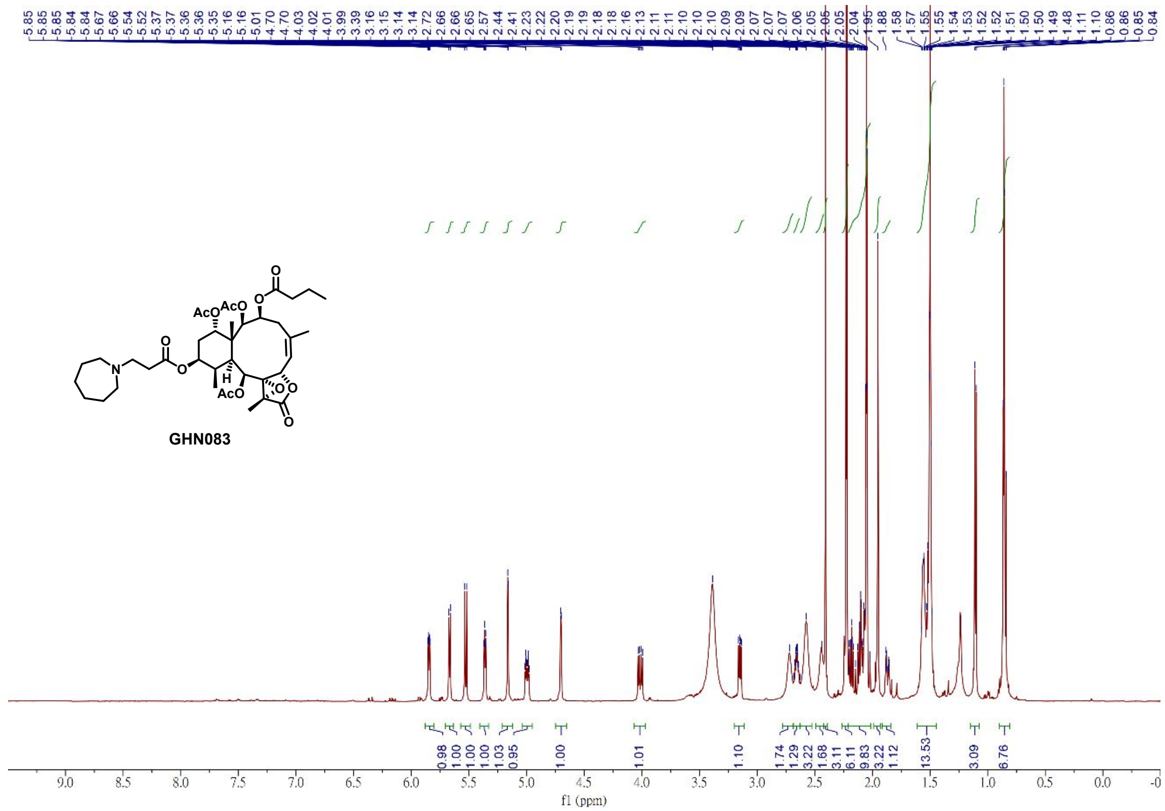

^{1}H NMR (600 MHz, $(CD_3)_2CO$, -40 ^{o}C) of **GHN083**.

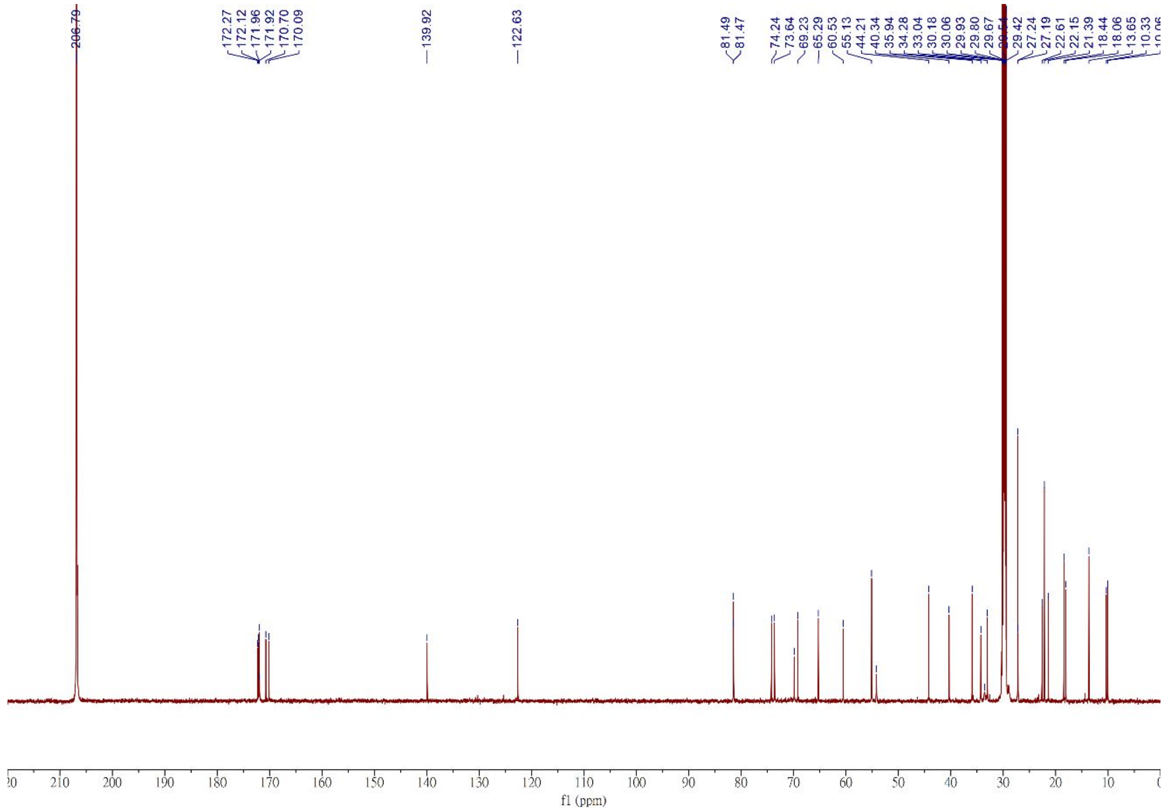

^{13}C NMR (151 MHz, $(CD_3)_2CO$, -40 ^{o}C) of **GHN083**.

HPLC purity spectra of **GHN083**.

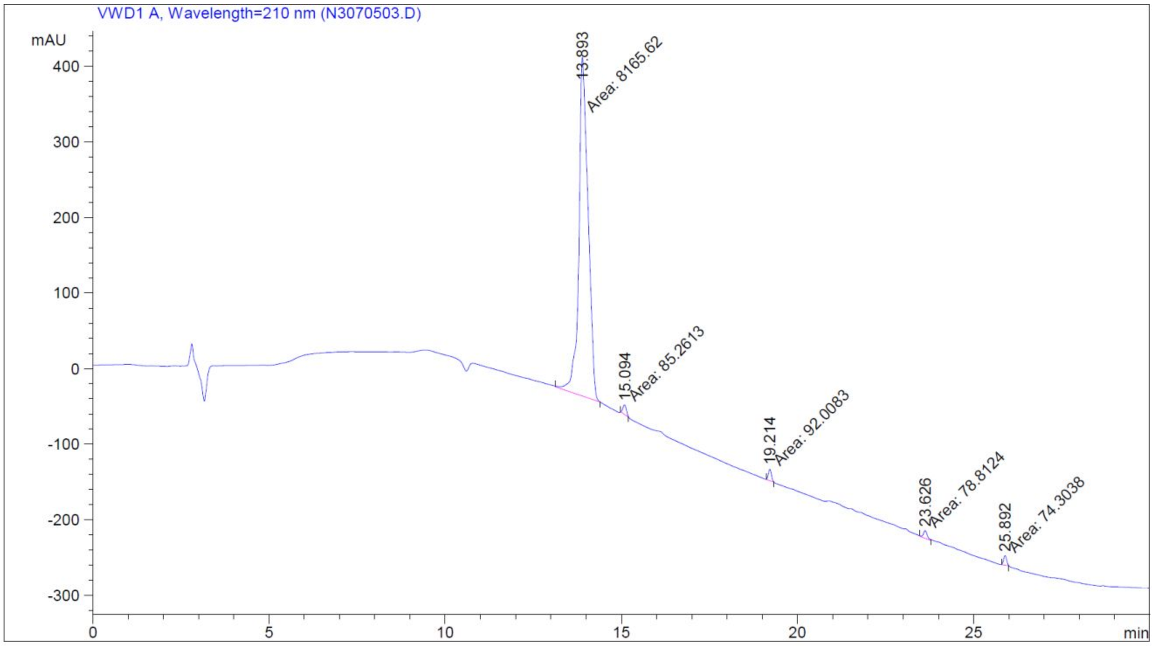


```
=====================================================================
                         Area Percent Report
=====================================================================

Sorted By             :      Signal
Multiplier            :      1.0000
Dilution              :      1.0000
Use Multiplier & Dilution Factor with ISTDs


Signal 1: VWD1 A, Wavelength=210 nm

Peak RetTime Type  Width     Area      Height     Area
  #   [min]        [min]  mAU    *s   [mAU    ]     %
----|-------|----|-------|----------|----------|--------|
   1  13.893 MM    0.3036 8165.61719  448.21310 96.1113
   2  15.094 MM    0.1125   85.26128   12.63684  1.0035
   3  19.214 MM    0.1016   92.00827   15.08699  1.0830
   4  23.626 MM    0.1289   78.81236   10.19014  0.9276
   5  25.892 MM    0.0994   74.30376   12.45264  0.8746

Totals :                  8496.00286  498.57971

 Results obtained with enhanced integrator!
=====================================================================
                         *** End of Report ***
```

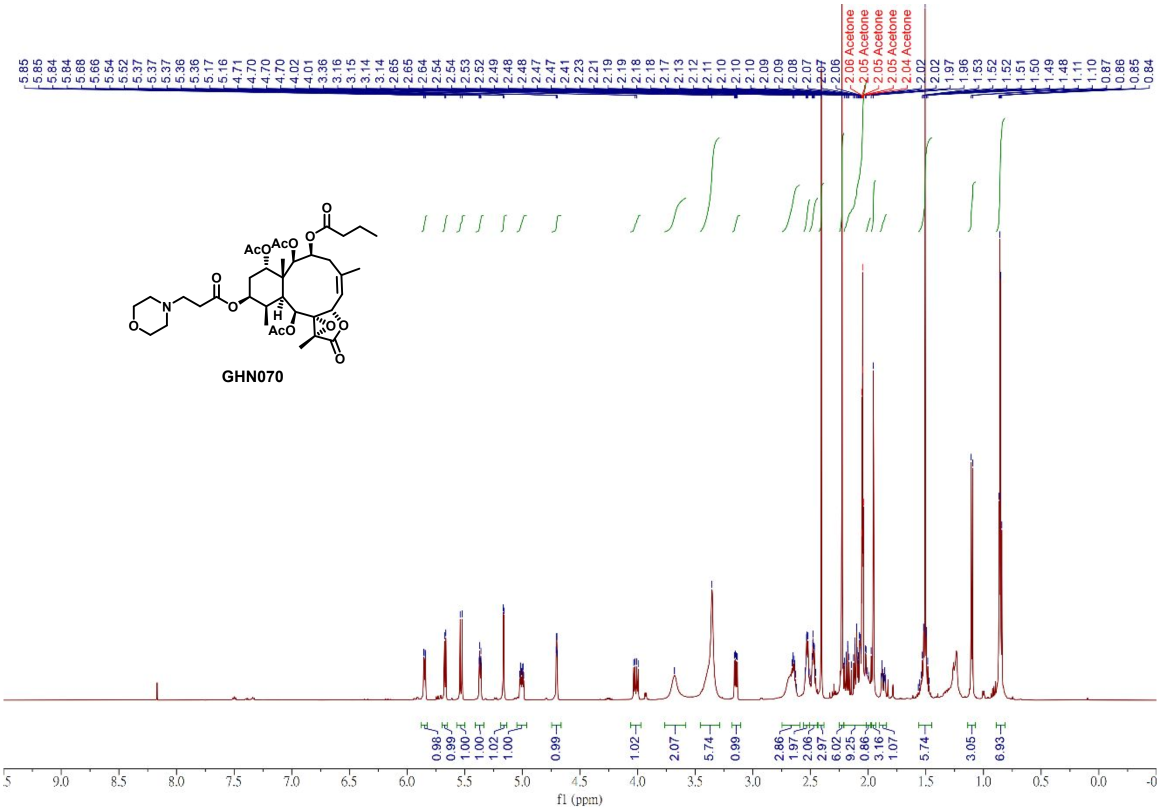

^{1}H NMR (600 MHz, $(CD_3)_2CO$, -40 °C) of **GHN070**.

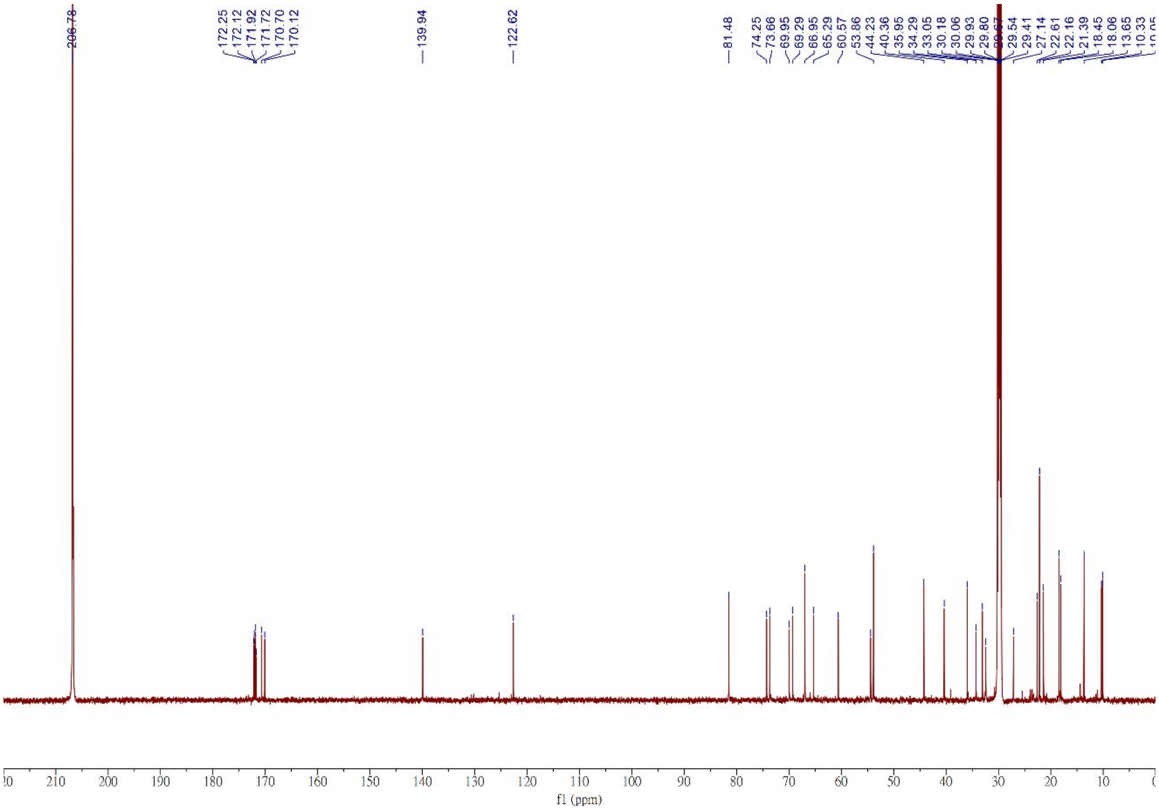

^{13}C NMR (151 MHz, $(CD_3)_2CO$, -40 °C) of **GHN070**.

HPLC purity spectra of **GHN070**.

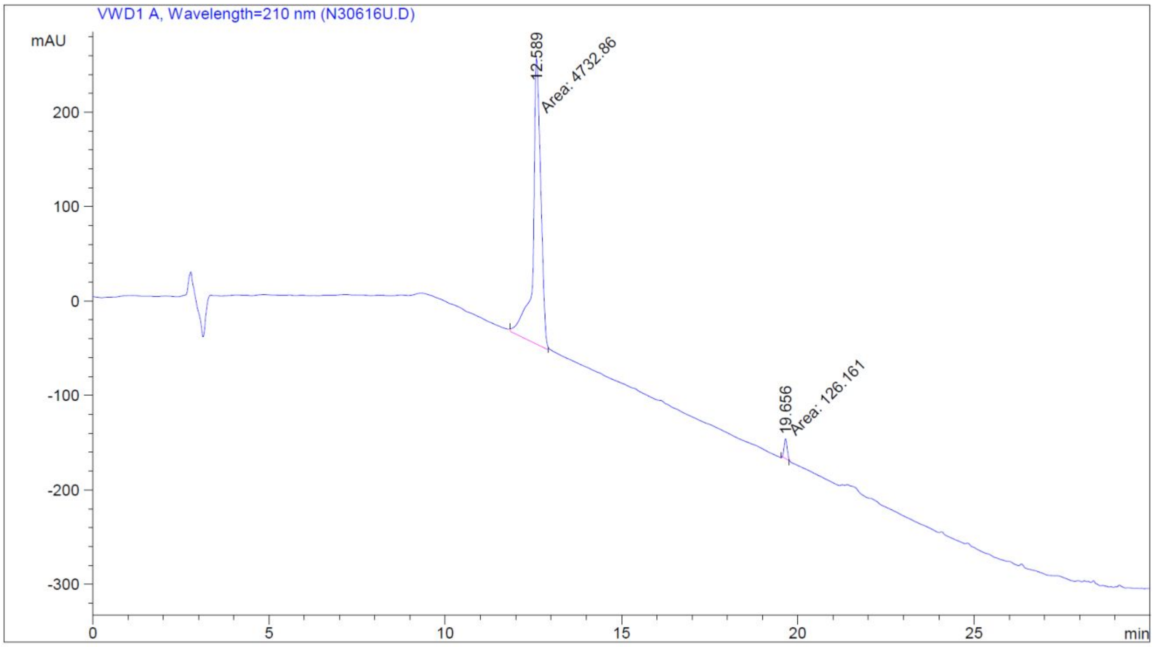


```
=====================================================================
                          Area Percent Report
=====================================================================

Sorted By             :      Signal
Multiplier            :      1.0000
Dilution              :      1.0000
Use Multiplier & Dilution Factor with ISTDs


Signal 1: VWD1 A, Wavelength=210 nm

Peak RetTime Type  Width     Area      Height     Area
  #   [min]        [min]  mAU    *s   [mAU   ]      %
----|-------|----|-------|----------|----------|--------|
   1  12.589 MM    0.2604 4732.85596  302.92249  97.4036
   2  19.656 MM    0.1003  126.16105   20.95737   2.5964

Totals :                  4859.01701  323.87986

 Results obtained with enhanced integrator!
=====================================================================
                          *** End of Report ***
```

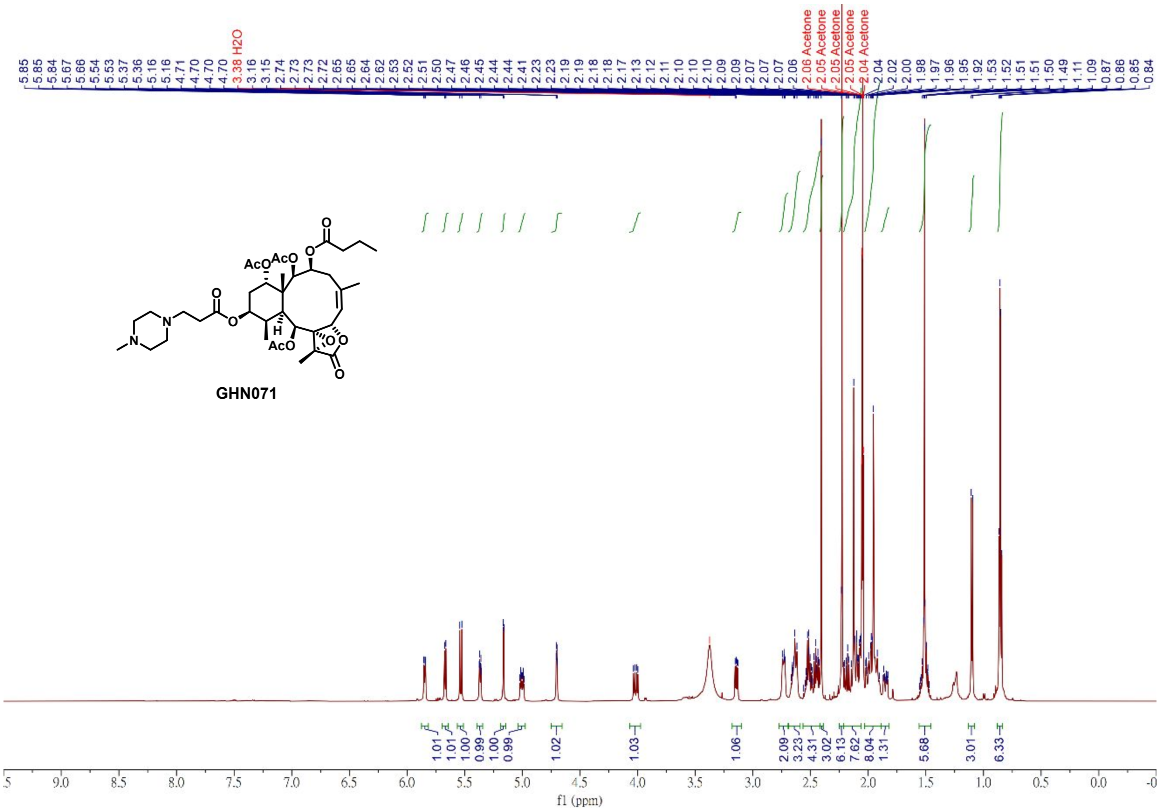

^{1}H NMR (600 MHz, $(CD_3)_2CO$, -40 ^{o}C) of **GHN071**.

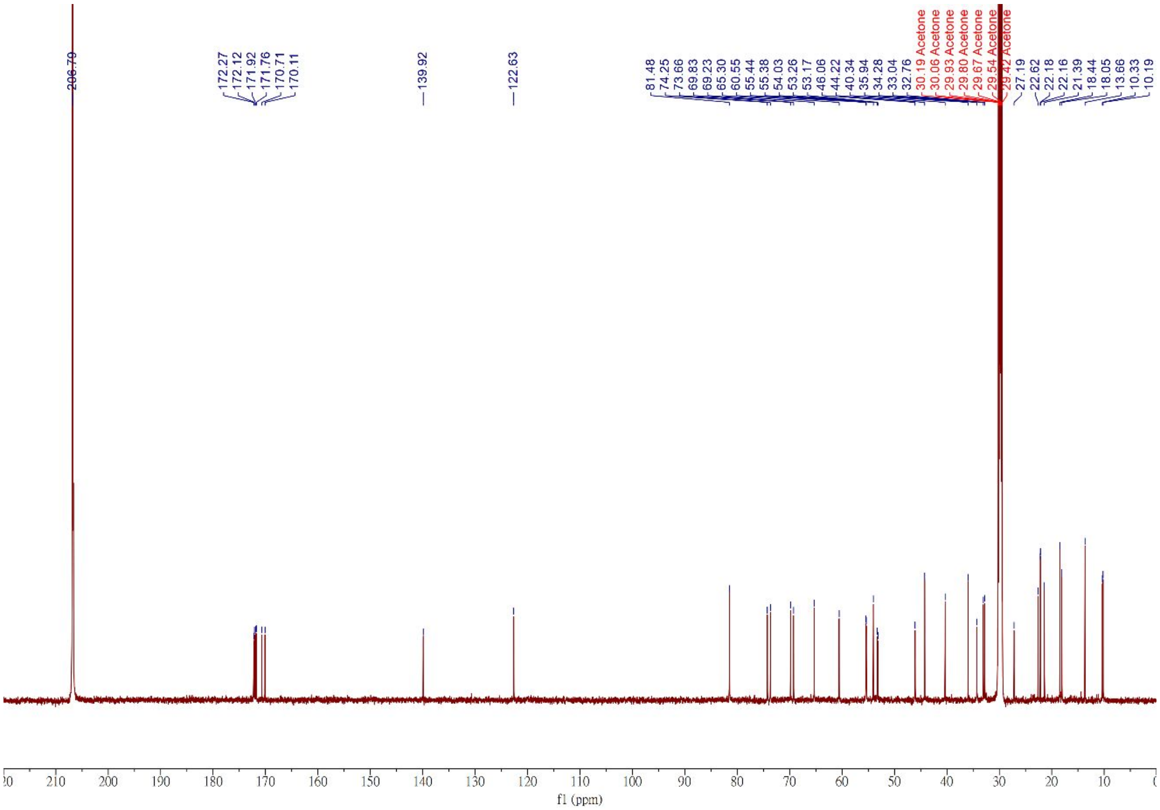

^{13}C NMR (151 MHz, $(CD_3)_2CO$, -40 ^{o}C) of **GHN071**.

HPLC purity spectra of **GHN071**.

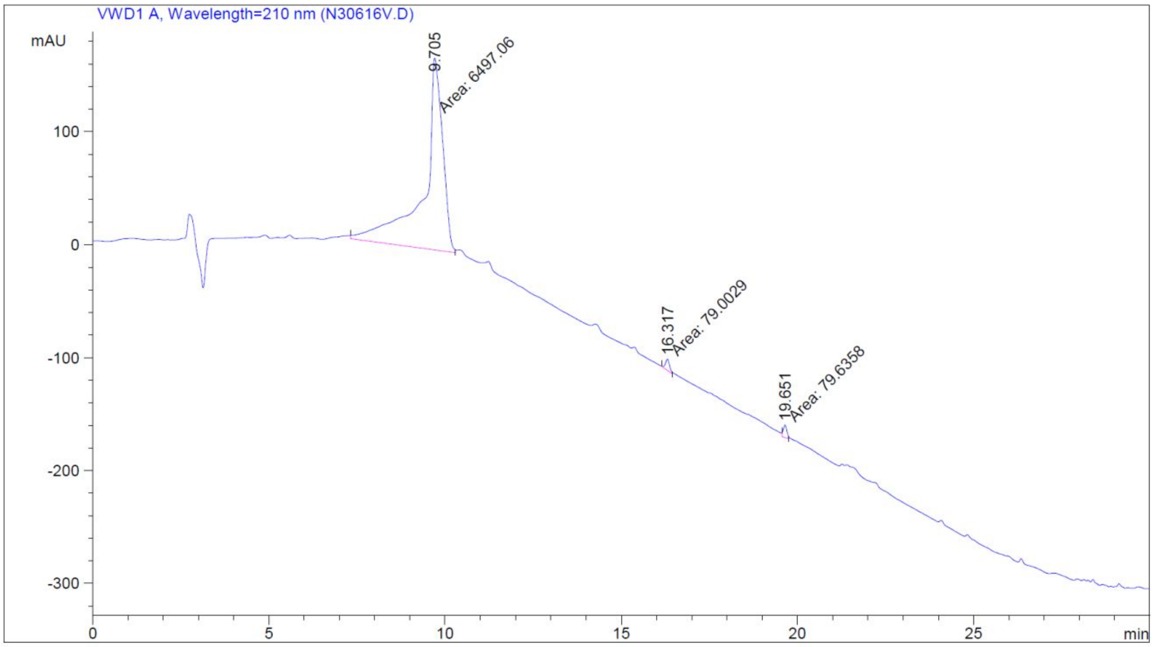


```
=====================================================================
                        Area Percent Report
=====================================================================

Sorted By             :      Signal
Multiplier            :      1.0000
Dilution              :      1.0000
Use Multiplier & Dilution Factor with ISTDs


Signal 1: VWD1 A, Wavelength=210 nm

Peak RetTime Type  Width     Area      Height     Area
  #   [min]        [min]  mAU   *s   [mAU   ]      %
----|-------|----|-------|----------|----------|--------|
   1   9.705 MM    0.6380 6497.05811  169.72058 97.6165
   2  16.317 MM    0.1323   79.00291    9.95508  1.1870
   3  19.651 MM    0.1184   79.63585   11.20760  1.1965

Totals :                  6655.69686  190.88326

 Results obtained with enhanced integrator!
=====================================================================
                          *** End of Report ***
```

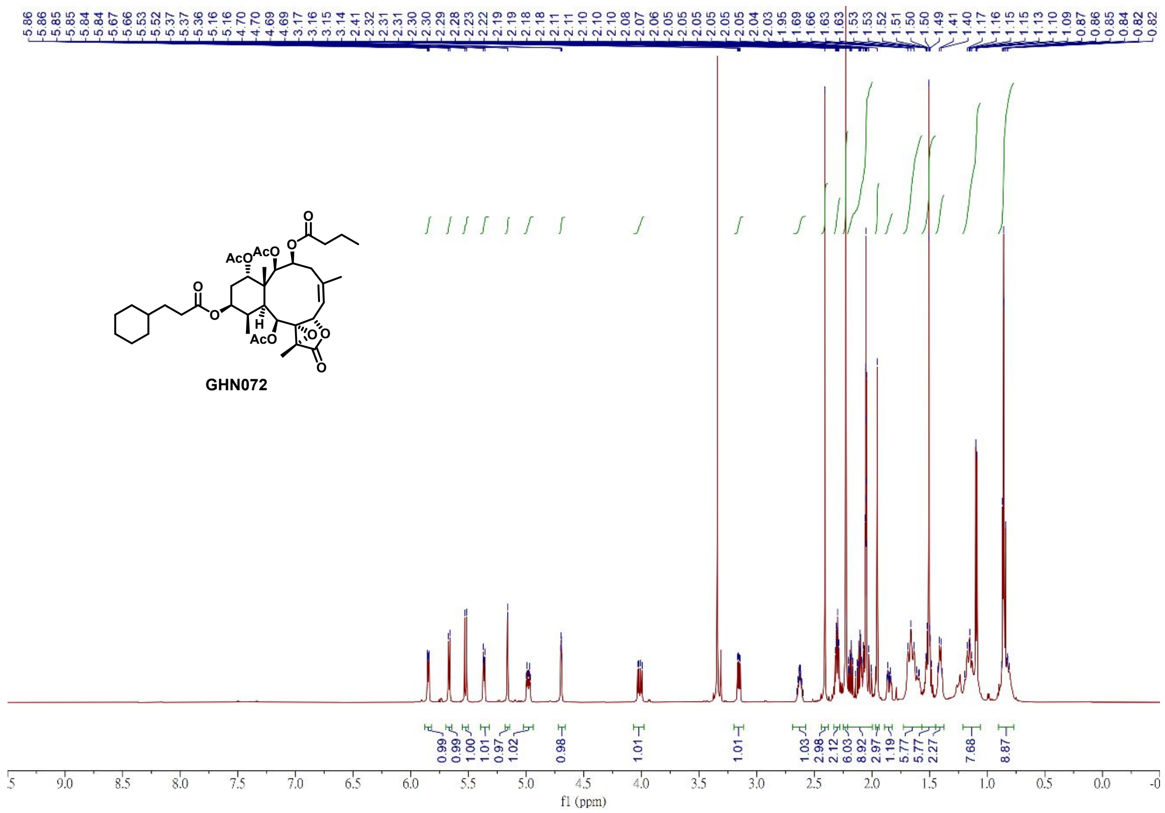

^1H NMR (600 MHz, $(CD_3)_2CO$, -40 °C) of **GHN072**.

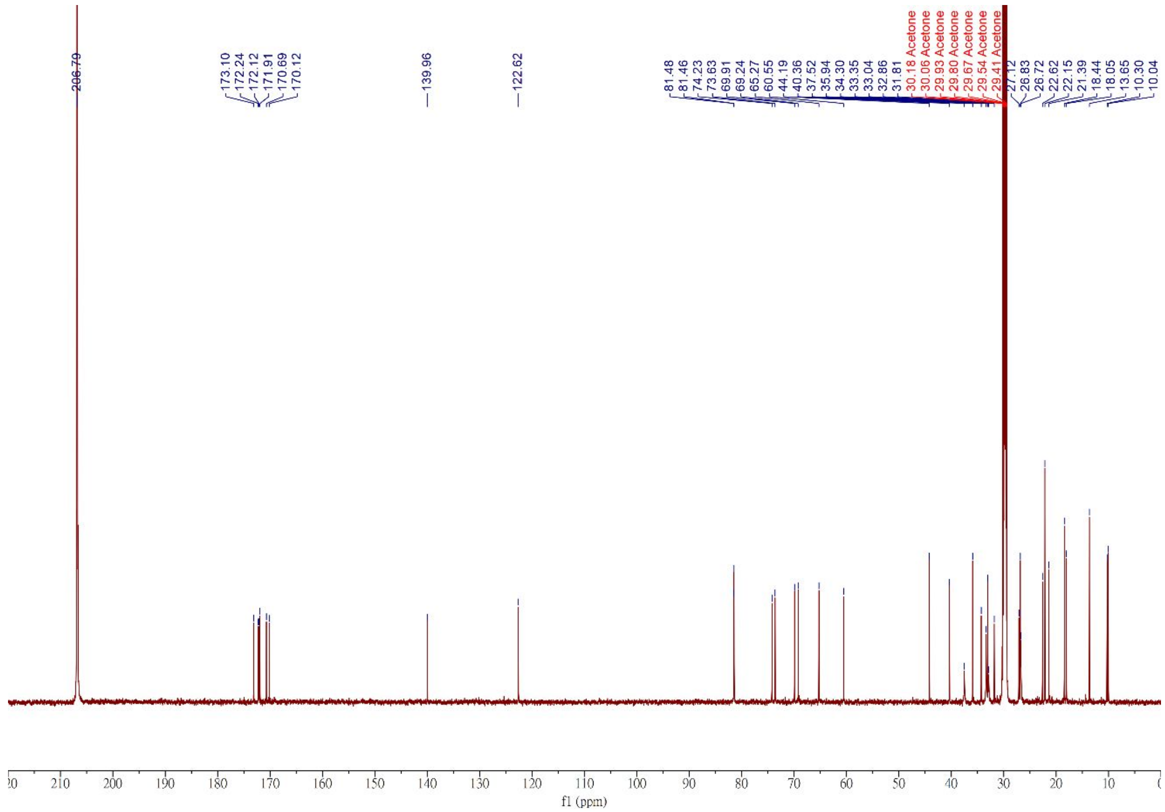

^{13}C NMR (151 MHz, $(CD_3)_2CO$, -40 °C) of **GHN072**.

HPLC purity spectra of **GHN072**.

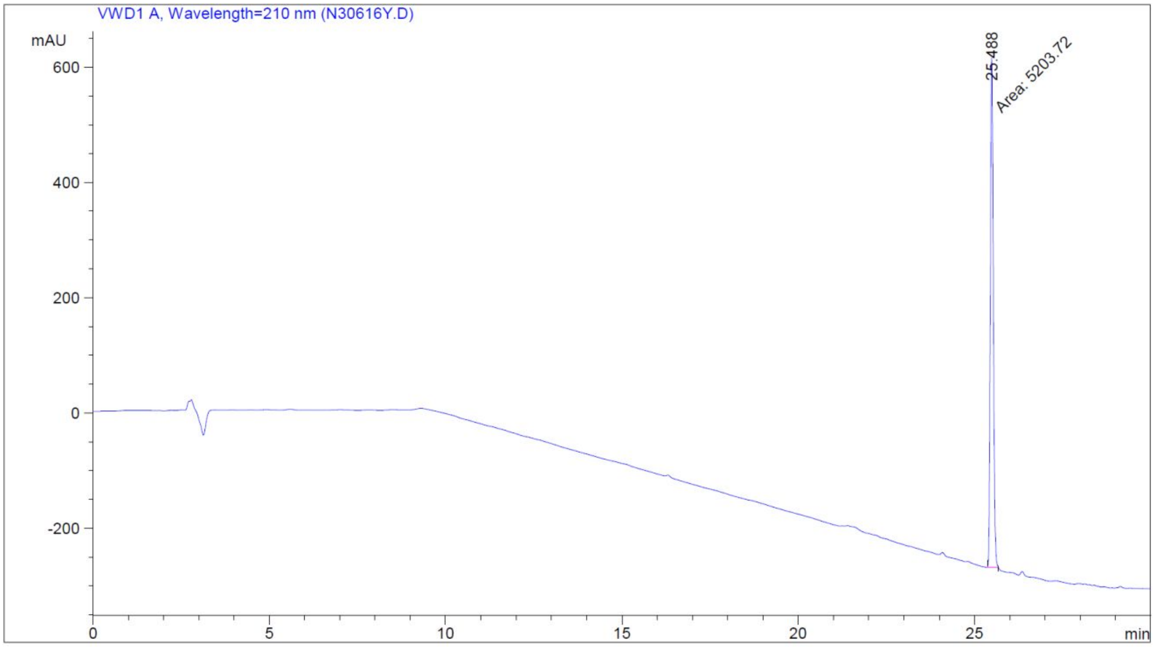


```
=====================================================================
                         Area Percent Report
=====================================================================

Sorted By             :      Signal
Multiplier            :      1.0000
Dilution              :      1.0000
Use Multiplier & Dilution Factor with ISTDs


Signal 1: VWD1 A, Wavelength=210 nm

Peak RetTime Type  Width     Area      Height     Area
  #   [min]        [min]  mAU   *s   [mAU   ]      %
----|-------|----|-------|----------|----------|--------|
   1  25.488 MM    0.0981 5203.71631  884.47375 100.0000

Totals :                  5203.71631  884.47375

 Results obtained with enhanced integrator!
=====================================================================
                          *** End of Report ***
```

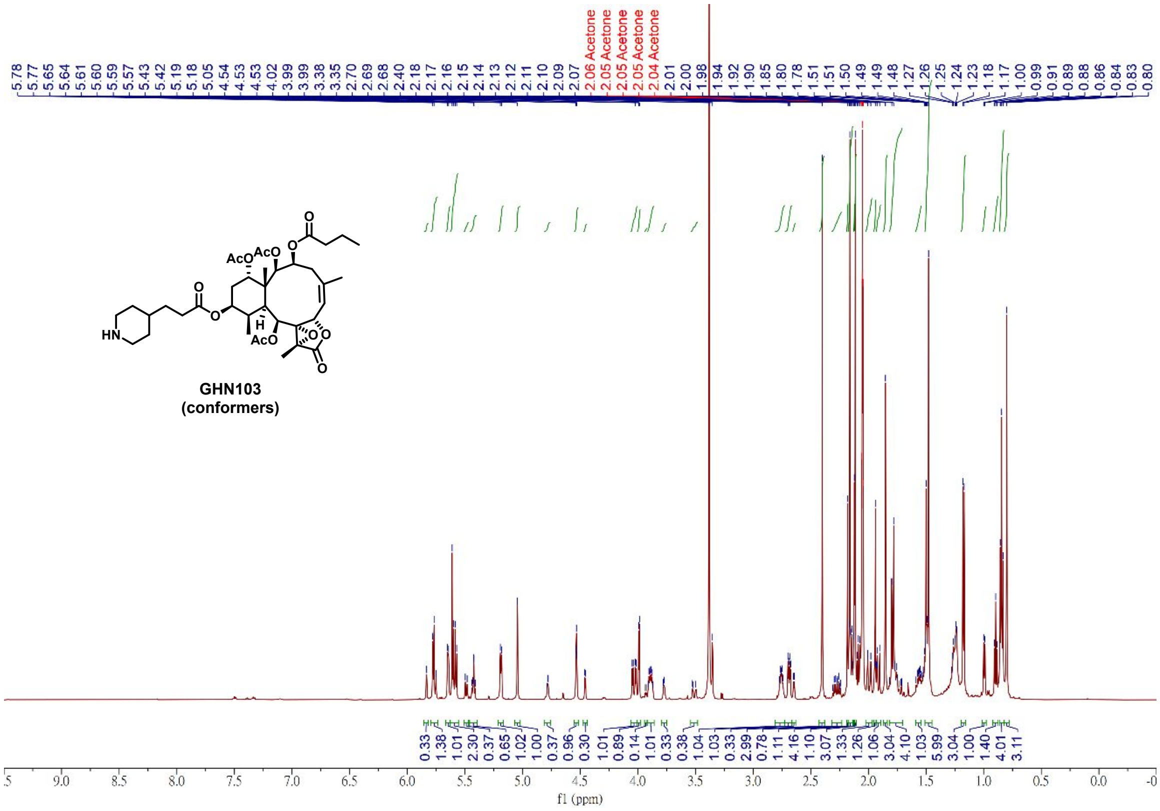

^{1}H NMR (600 MHz, $(CD_3)_2CO$, -40 ^{o}C) of **GHN103**.

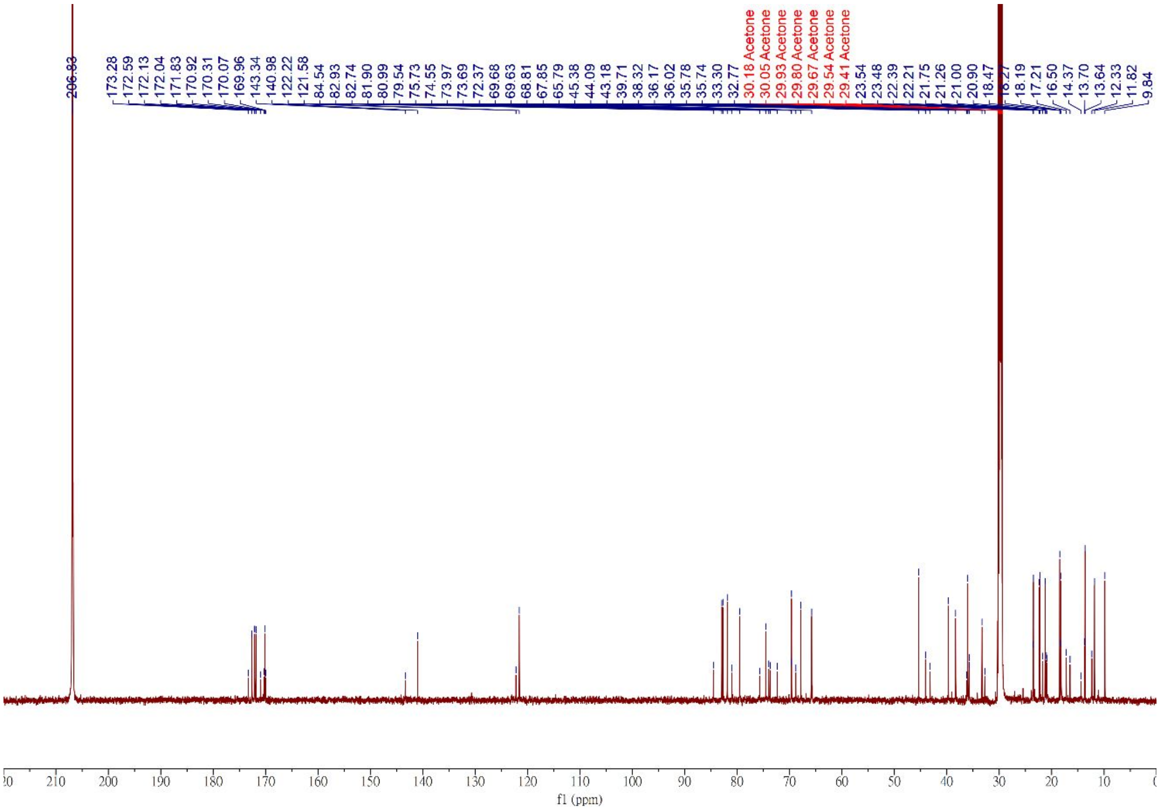

^{13}C NMR (151 MHz, $(CD_3)_2CO$, -40 ^{o}C) of **GHN103**.

HPLC purity spectra of **GHN103**.

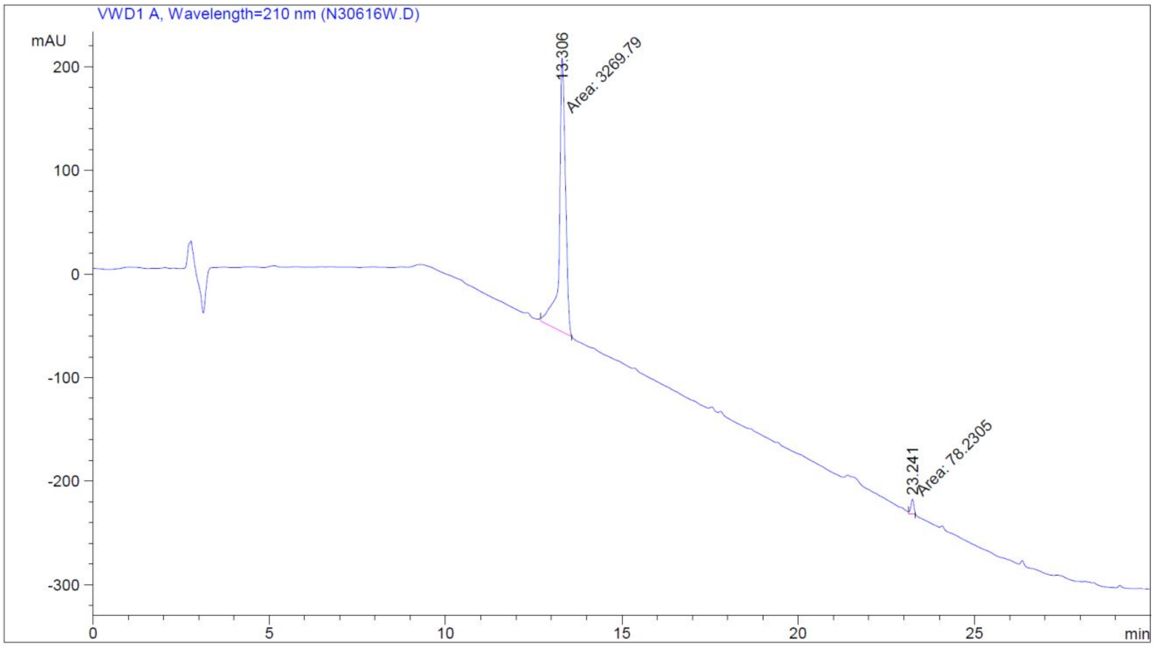


```
=====================================================================
                         Area Percent Report
=====================================================================

Sorted By             :      Signal
Multiplier            :      1.0000
Dilution              :      1.0000
Use Multiplier & Dilution Factor with ISTDs


Signal 1: VWD1 A, Wavelength=210 nm

Peak RetTime Type  Width     Area      Height     Area
  #   [min]        [min]  mAU   *s    [mAU   ]      %
----|-------|----|-------|----------|----------|--------|
   1  13.306 MM    0.2062 3269.78711  264.30026  97.6634
   2  23.241 MM    0.0948   78.23046   13.75509   2.3366

Totals :                  3348.01757  278.05535

 Results obtained with enhanced integrator!
=====================================================================
                          *** End of Report ***
```

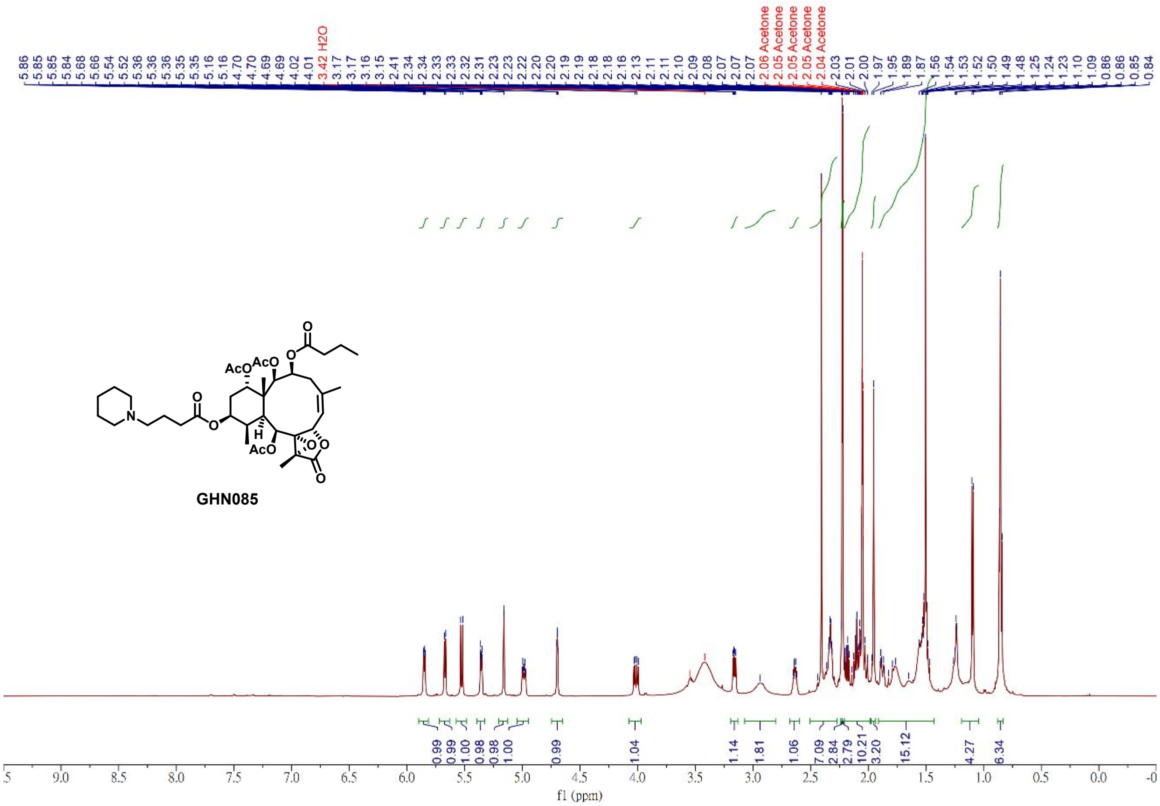

^{1}H NMR (600 MHz, $(CD_3)_2CO$, -40 ^{o}C) of **GHN085**.

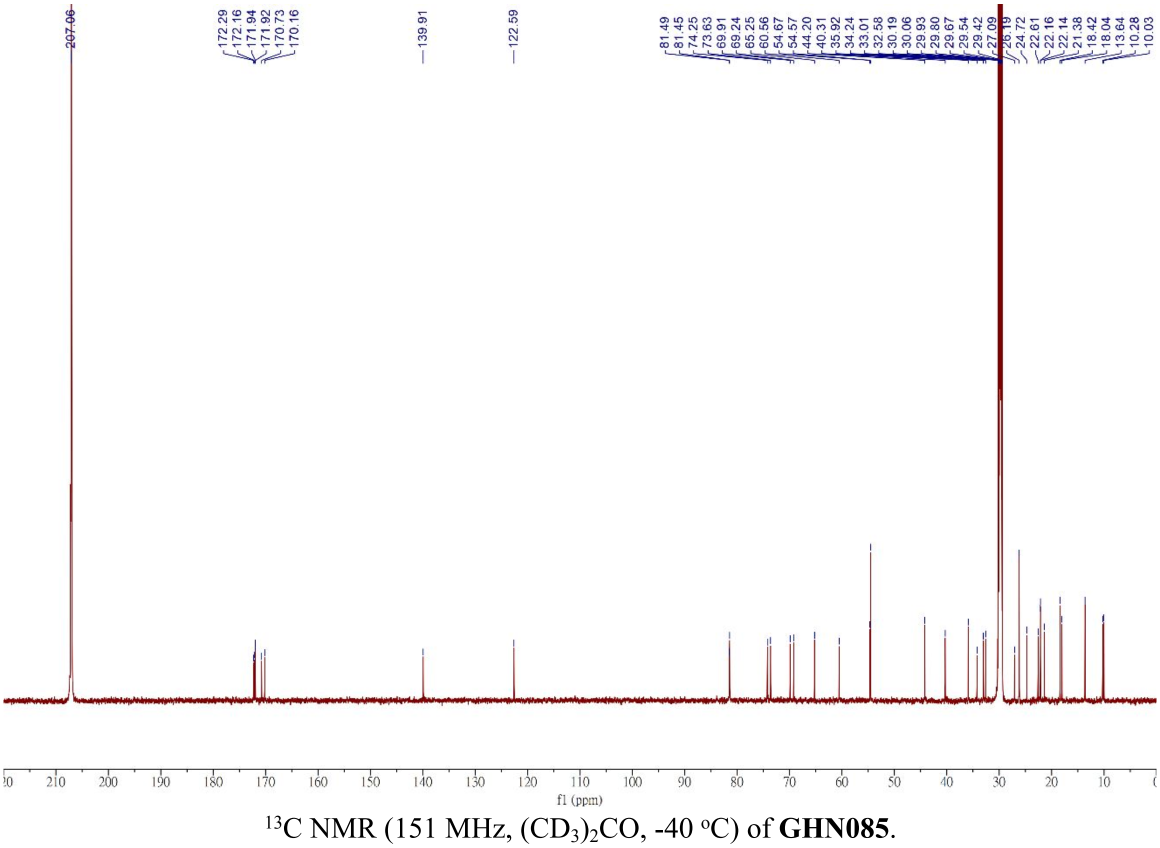

^{13}C NMR (151 MHz, $(CD_3)_2CO$, -40 ^{o}C) of **GHN085**.

HPLC purity spectra of **GHN085**.

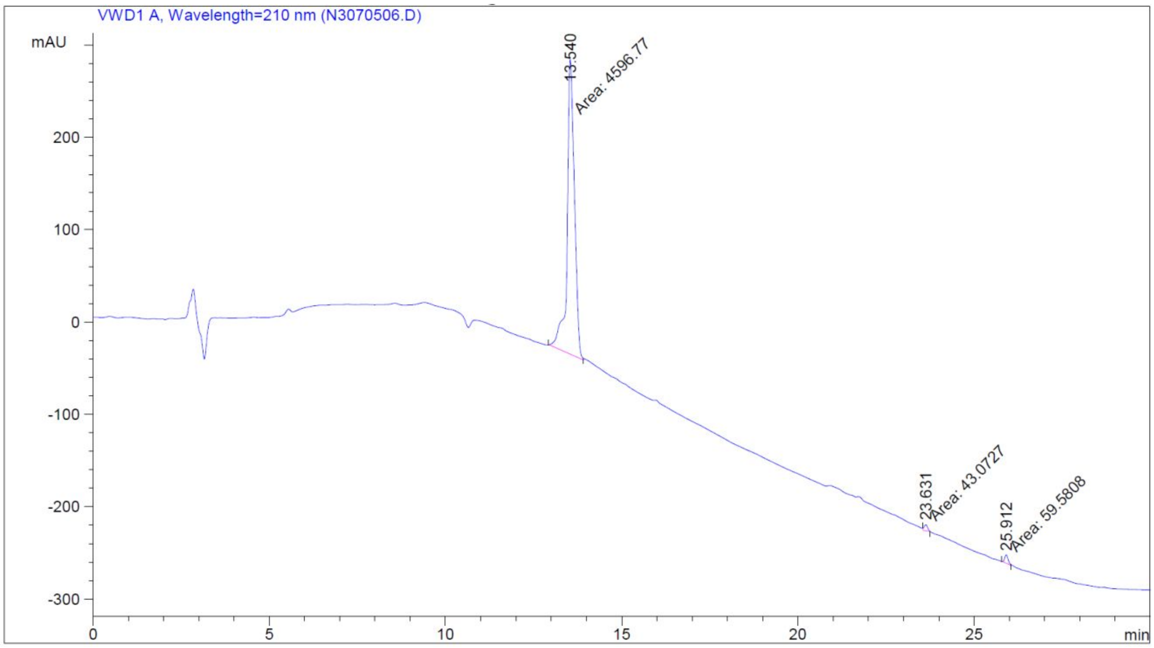


```
=====================================================================
                          Area Percent Report
=====================================================================

Sorted By             :      Signal
Multiplier            :      1.0000
Dilution              :      1.0000
Use Multiplier & Dilution Factor with ISTDs


Signal 1: VWD1 A, Wavelength=210 nm

Peak RetTime Type  Width     Area      Height     Area
  #   [min]        [min]   mAU    *s   [mAU   ]      %
----|-------|----|-------|----------|----------|--------|
   1  13.540 MM    0.2399 4596.76953  319.37723  97.8156
   2  23.631 MM    0.1099   43.07265    6.53230   0.9166
   3  25.912 MM    0.1104   59.58077    8.99527   1.2678

Totals :                  4699.42295  334.90480

 Results obtained with enhanced integrator!
=====================================================================
                          *** End of Report ***
```

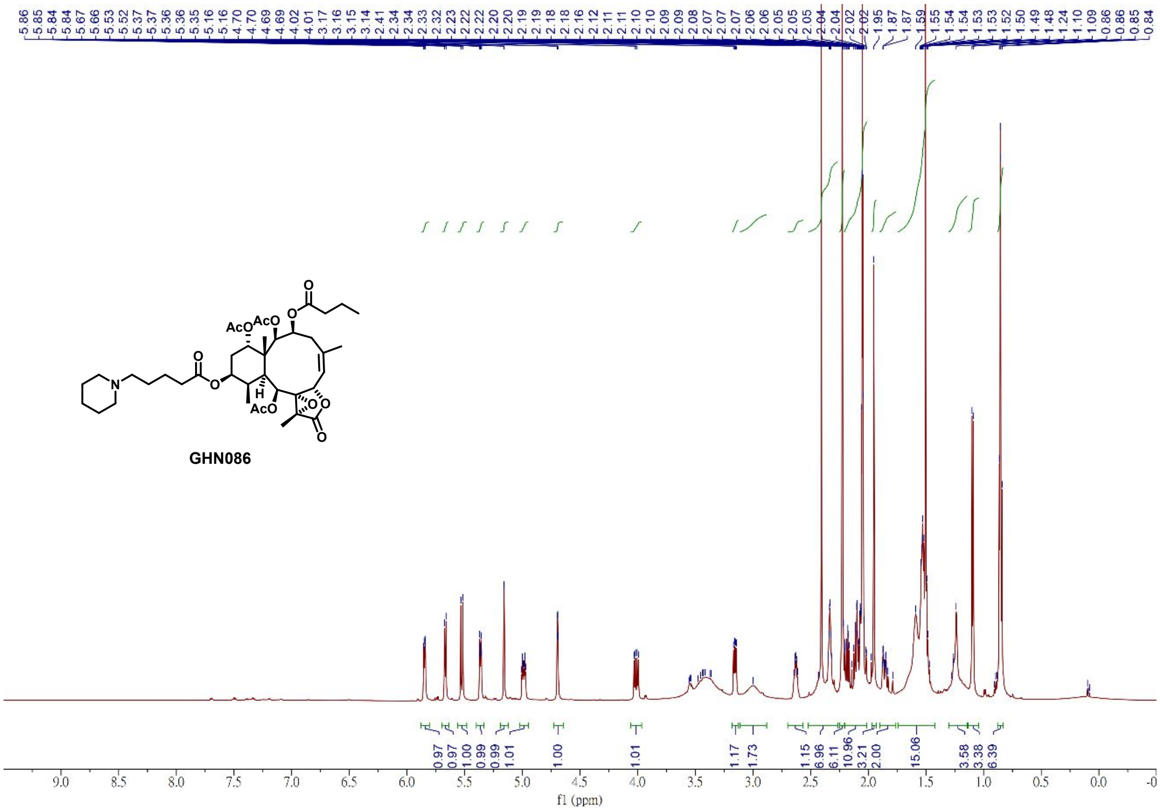

^{1}H NMR (600 MHz, $(CD_3)_2CO$, -40 °C) of **GHN086**.

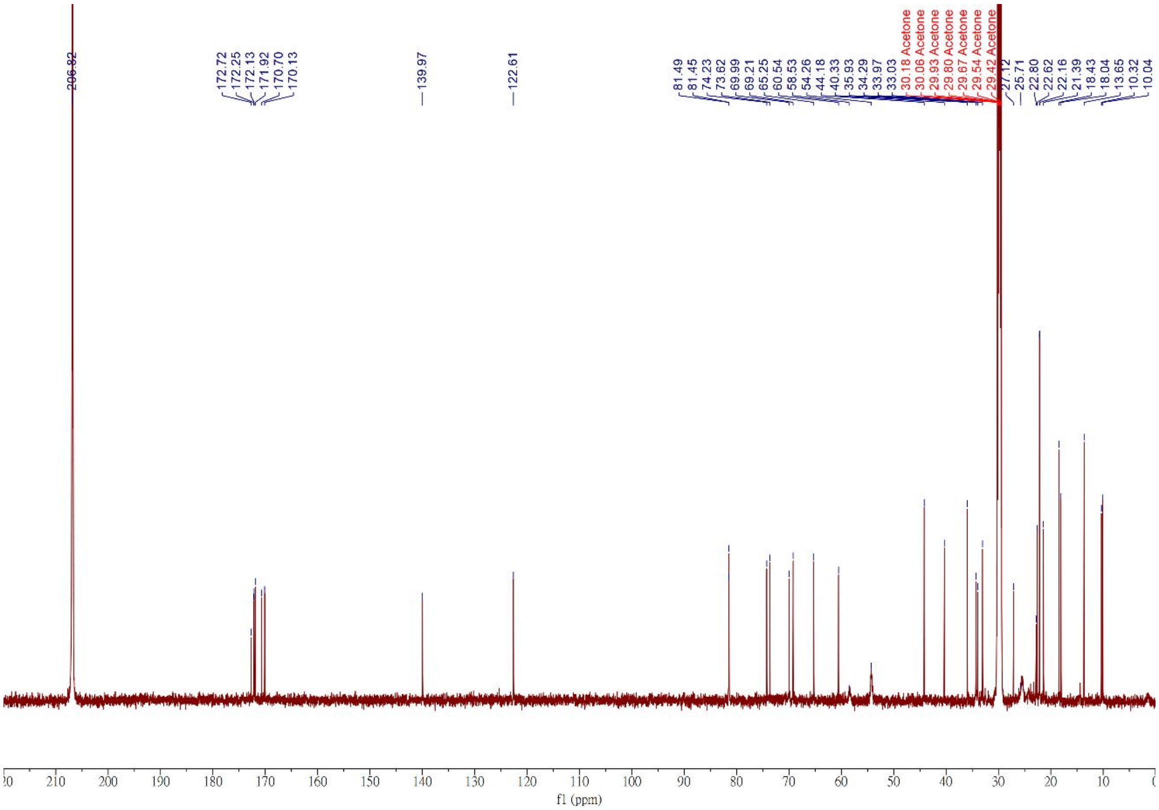

^{13}C NMR (151 MHz, $(CD_3)_2CO$, -40 °C) of **GHN086**.

HPLC purity spectra of **GHN086**.

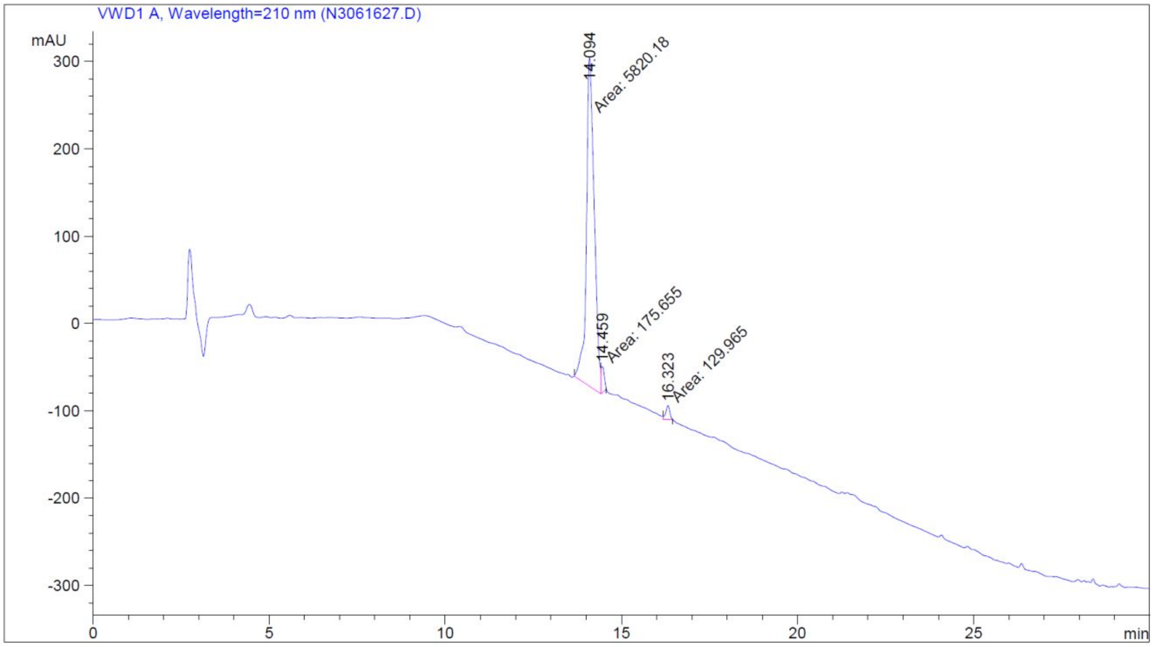

===
Area Percent Report
===

Sorted By : Signal
Multiplier : 1.0000
Dilution : 1.0000
Use Multiplier & Dilution Factor with ISTDs

Signal 1: VWD1 A, Wavelength=210 nm

Peak #	RetTime [min]	Type	Width [min]	Area mAU *s	Height [mAU]	Area %
1	14.094	MM	0.2580	5820.17773	376.04123	95.0109
2	14.459	MM	0.0967	175.65549	30.27708	2.8675
3	16.323	MM	0.1336	129.96490	16.21058	2.1216

Totals : 6125.79813 422.52889

Results obtained with enhanced integrator!
===
*** End of Report ***

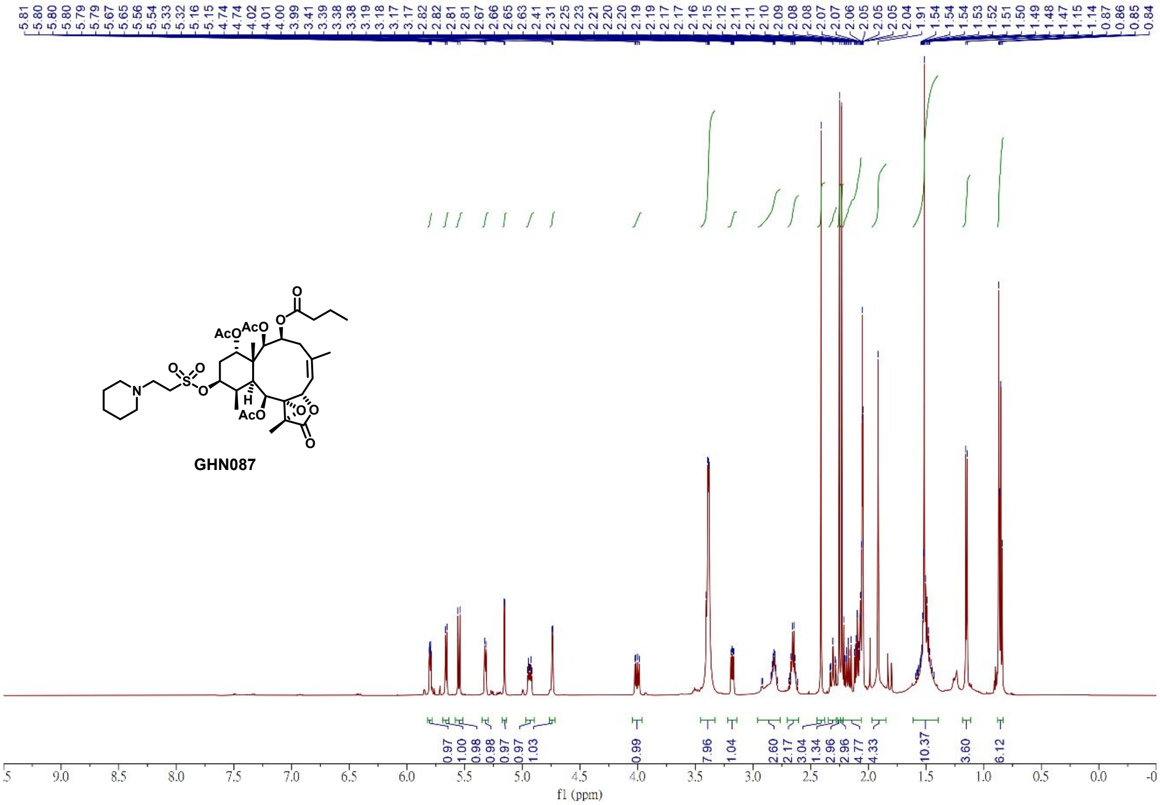

GHN087

^{1}H NMR (600 MHz, $(CD_3)_2CO$, -40 ^{o}C) of **GHN087**.

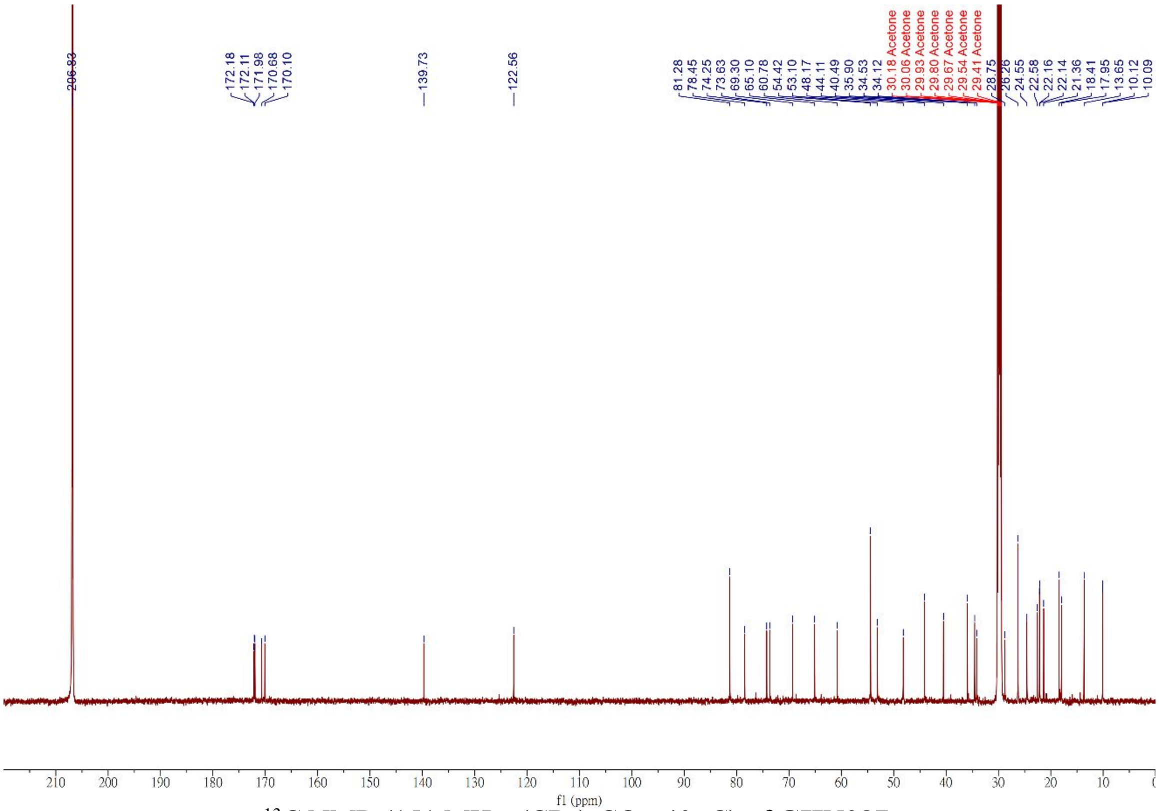

^{13}C NMR (151 MHz, $(CD_3)_2CO$, -40 ^{o}C) of **GHN087**.

HPLC purity spectra of **GHN087**.

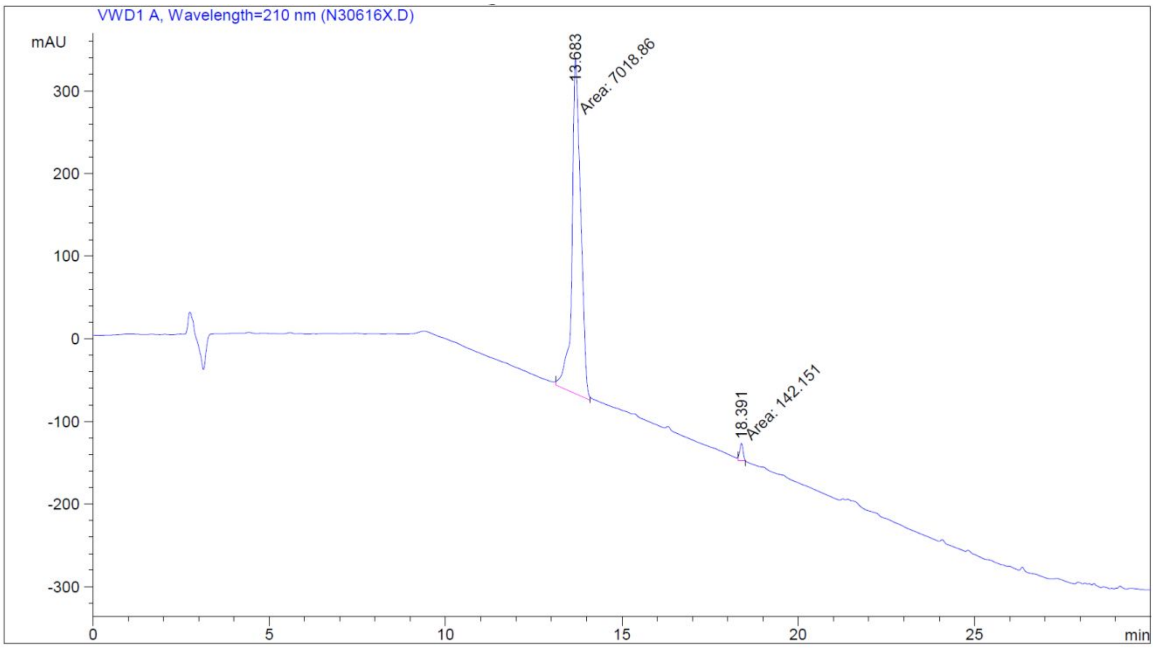


```
=====================================================================
                         Area Percent Report
=====================================================================

Sorted By             :      Signal
Multiplier            :      1.0000
Dilution              :      1.0000
Use Multiplier & Dilution Factor with ISTDs


Signal 1: VWD1 A, Wavelength=210 nm

Peak RetTime Type  Width     Area      Height     Area
  #   [min]        [min]  mAU   *s    [mAU   ]     %
----|-------|----|-------|----------|----------|--------|
   1  13.683 MM    0.2891 7018.86475  404.63629  98.0149
   2  18.391 MM    0.1127  142.15135   21.02473   1.9851

Totals :                  7161.01610  425.66102

 Results obtained with enhanced integrator!
=====================================================================
                         *** End of Report ***
```

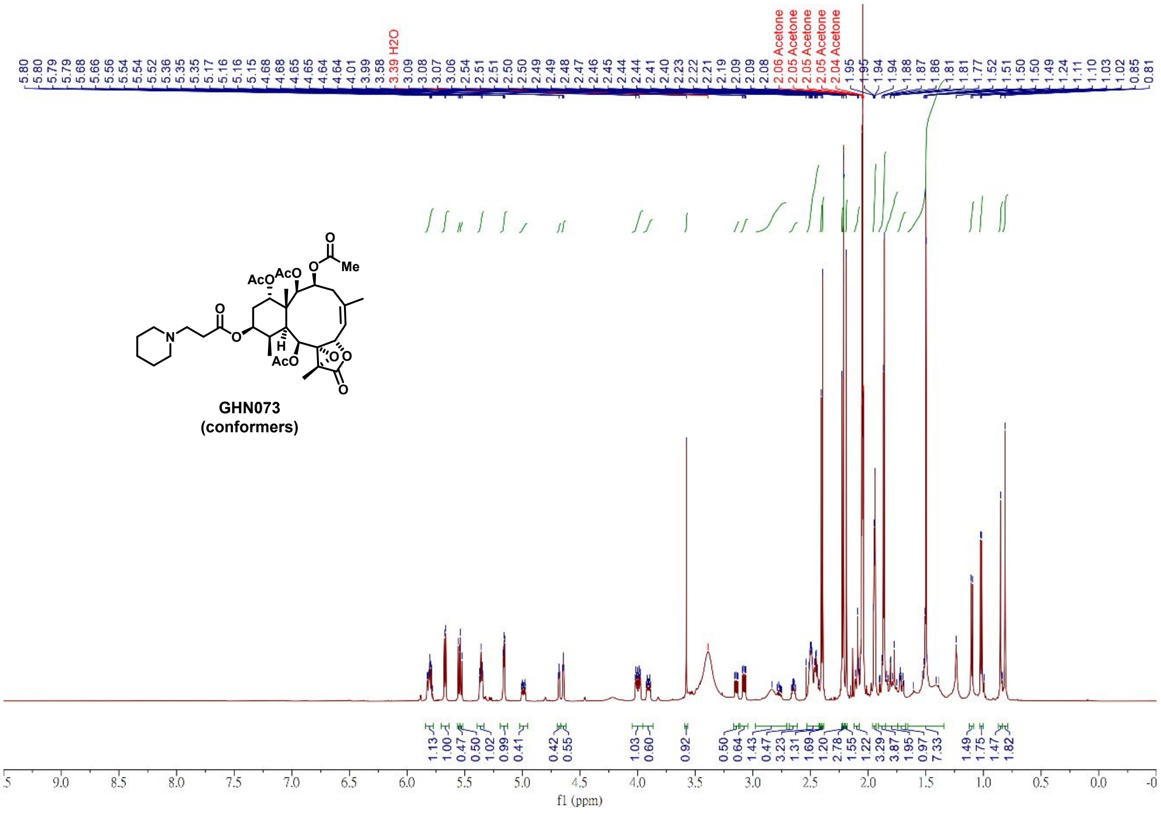

^1H NMR (600 MHz, $(CD_3)_2CO$, -40 °C) of **GHN073**.

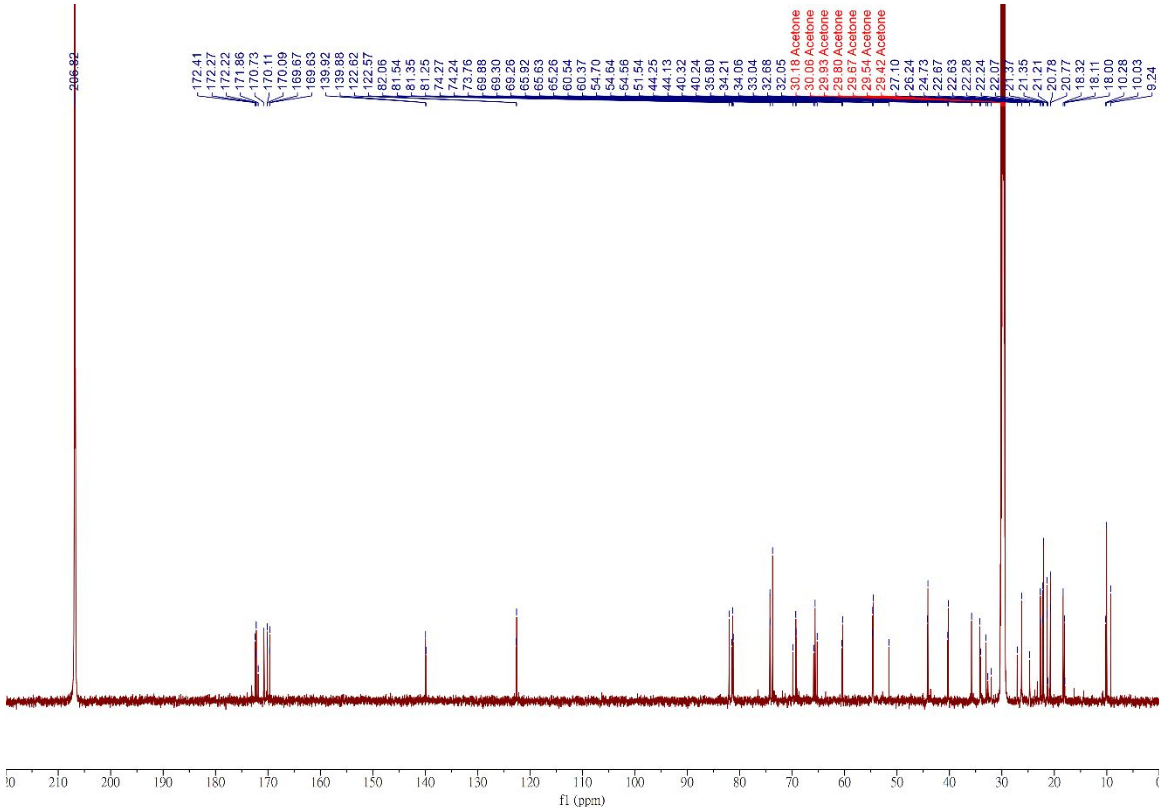

^{13}C NMR (151 MHz, $(CD_3)_2CO$, -40 °C) of **GHN073**.

HPLC purity spectra of **GHN073**. (λ = 210 nm)

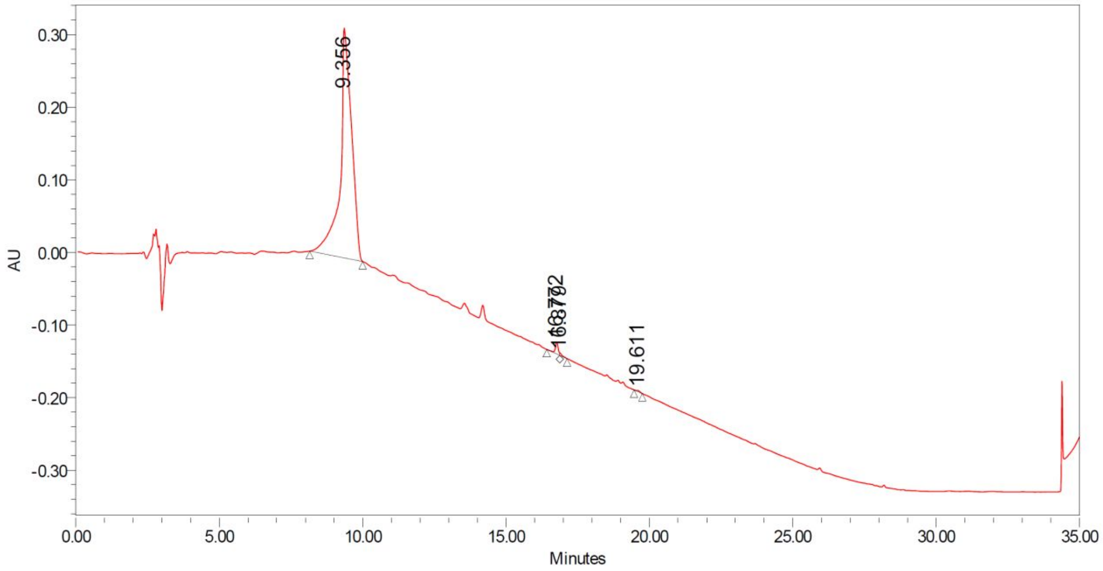

	RT	Area	% Area	Height
1	9.356	8799147	98.38	316500
2	16.772	113421	1.27	16761
3	16.879	18472	0.21	3498
4	19.611	13112	0.15	1736

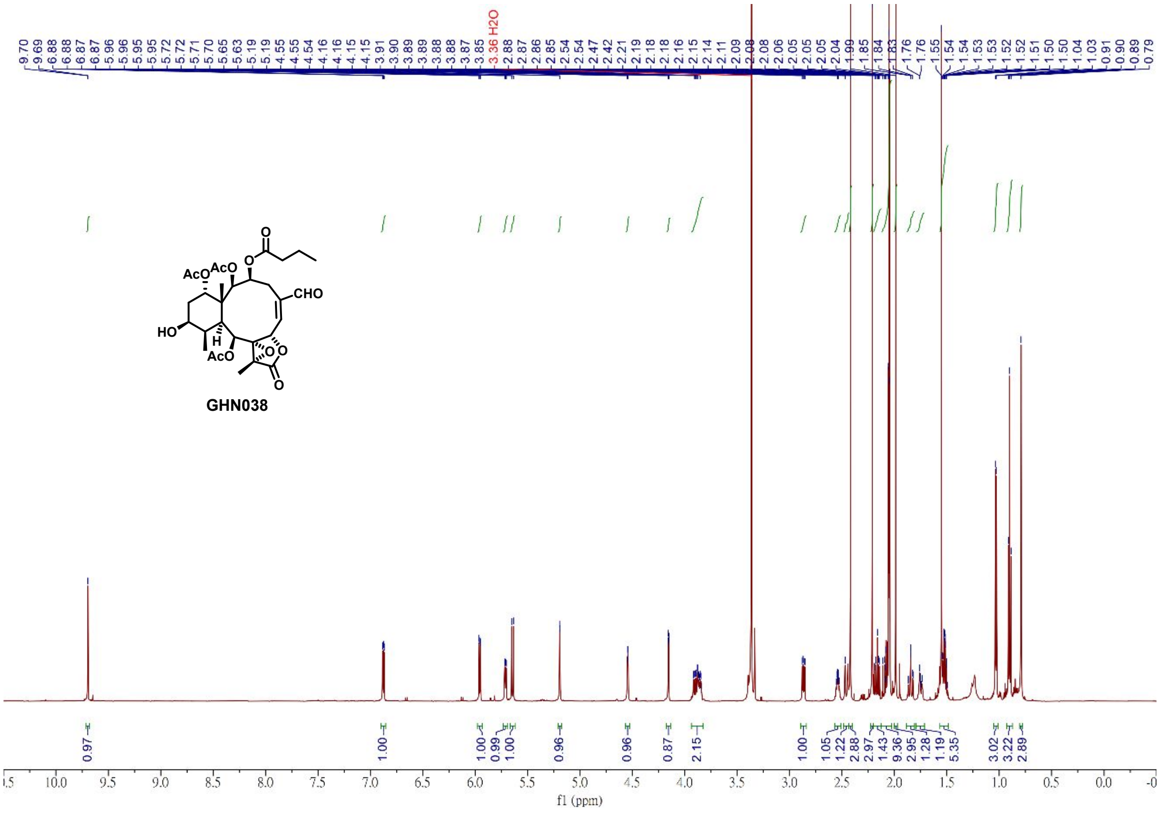

^{1}H NMR (600 MHz, $(CD_3)_2CO$, -40 °C) of **GHN038**.

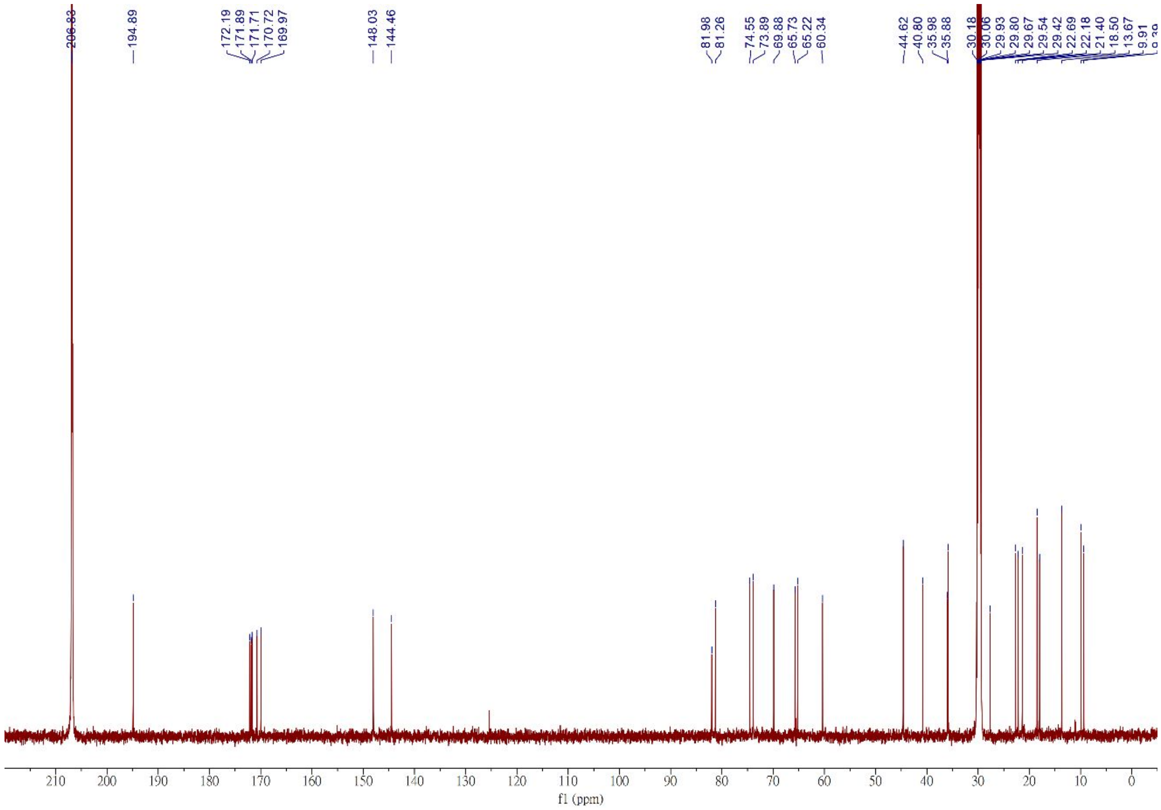

^{13}C NMR (151 MHz, $(CD_3)_2CO$, -40 °C) of **GHN038**.

HPLC purity spectra of **GHN038**.

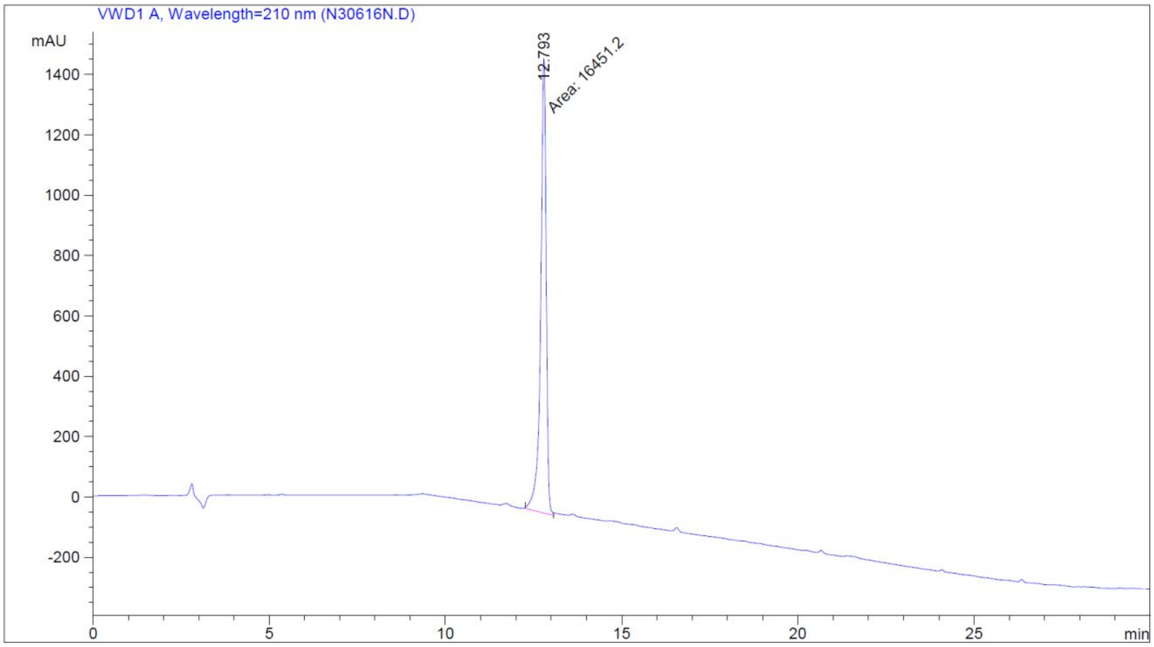


```
==================================================================
                        Area Percent Report
==================================================================

Sorted By             :      Signal
Multiplier            :      1.0000
Dilution              :      1.0000
Use Multiplier & Dilution Factor with ISTDs


Signal 1: VWD1 A, Wavelength=210 nm

Peak RetTime Type  Width     Area      Height     Area
  #   [min]        [min]  mAU   *s    [mAU   ]      %
----|-------|----|-------|----------|----------|--------|
   1  12.793 MM    0.1819 1.64512e4  1507.72510 100.0000

Totals :                  1.64512e4  1507.72510

 Results obtained with enhanced integrator!
==================================================================
                         *** End of Report ***
```

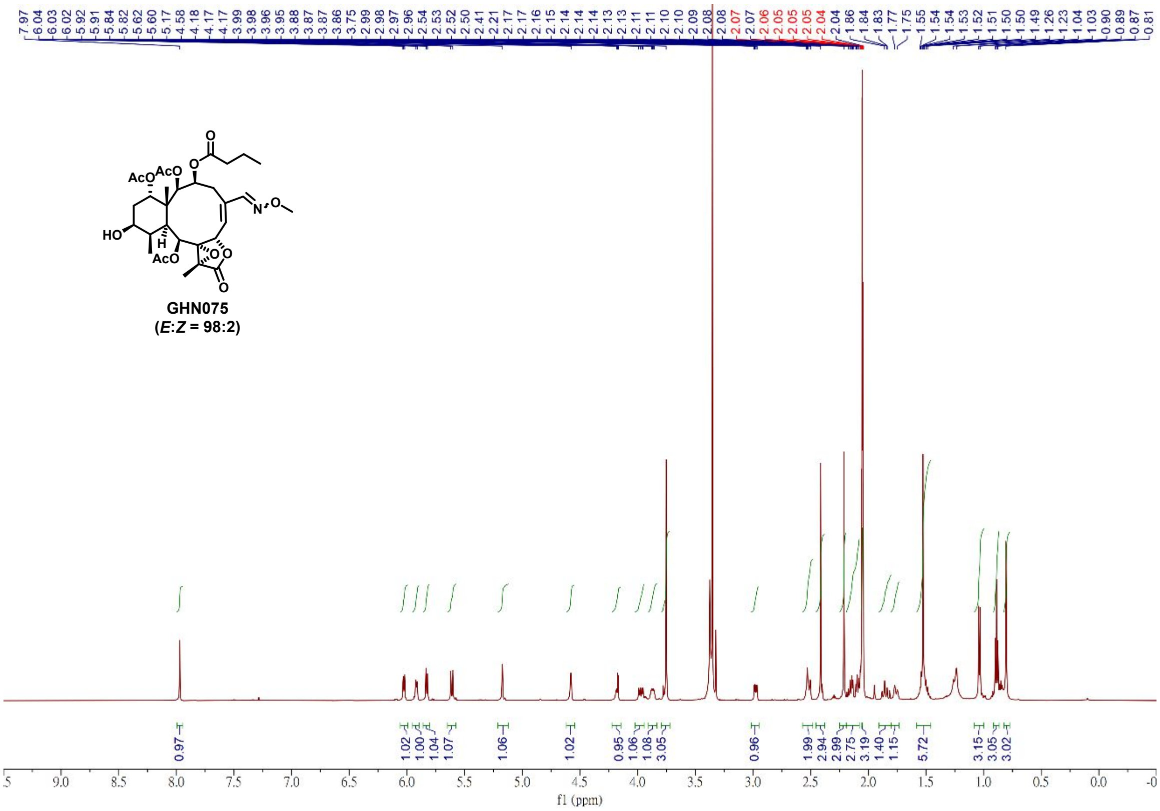

^{1}H NMR (600 MHz, $(CD_3)_2CO$, -40 ^{o}C) of **GHN075**.

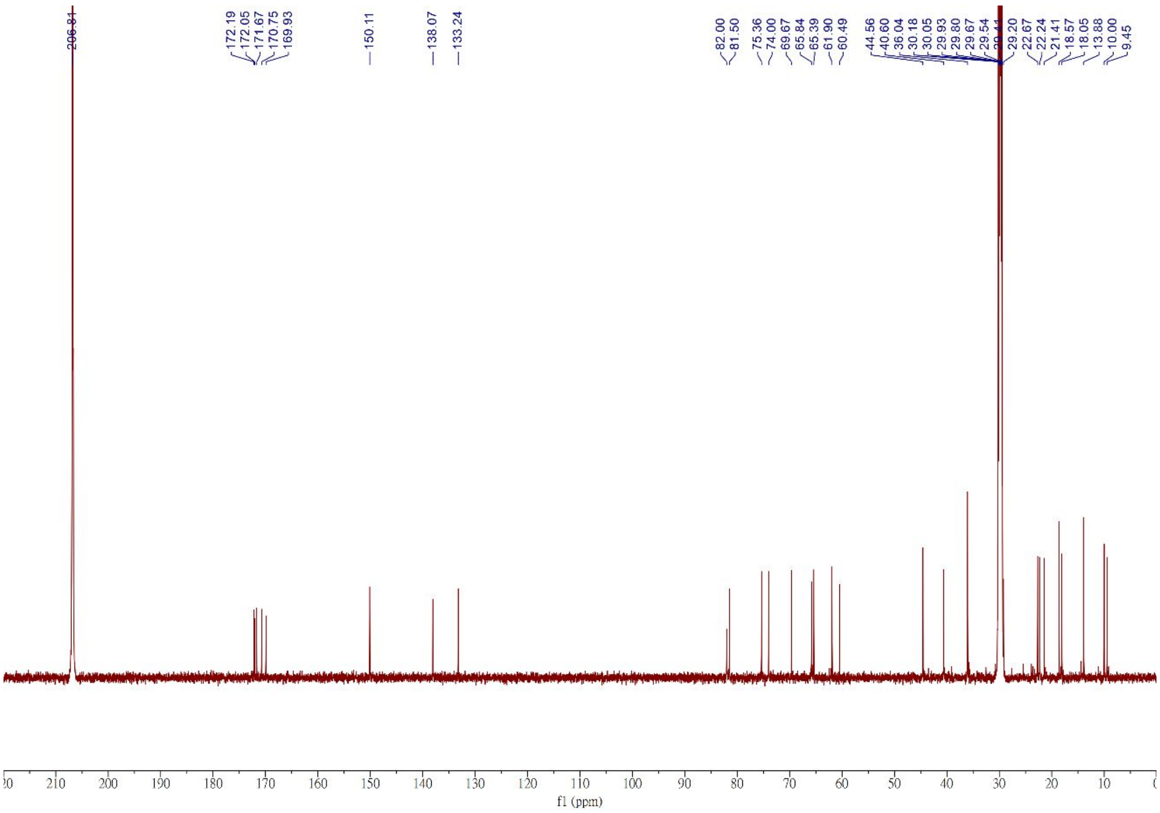

^{13}C NMR (151 MHz, $(CD_3)_2CO$, -40 ^{o}C) of **GHN075**.

HPLC purity spectra of **GHN075**. (λ = 254 nm)

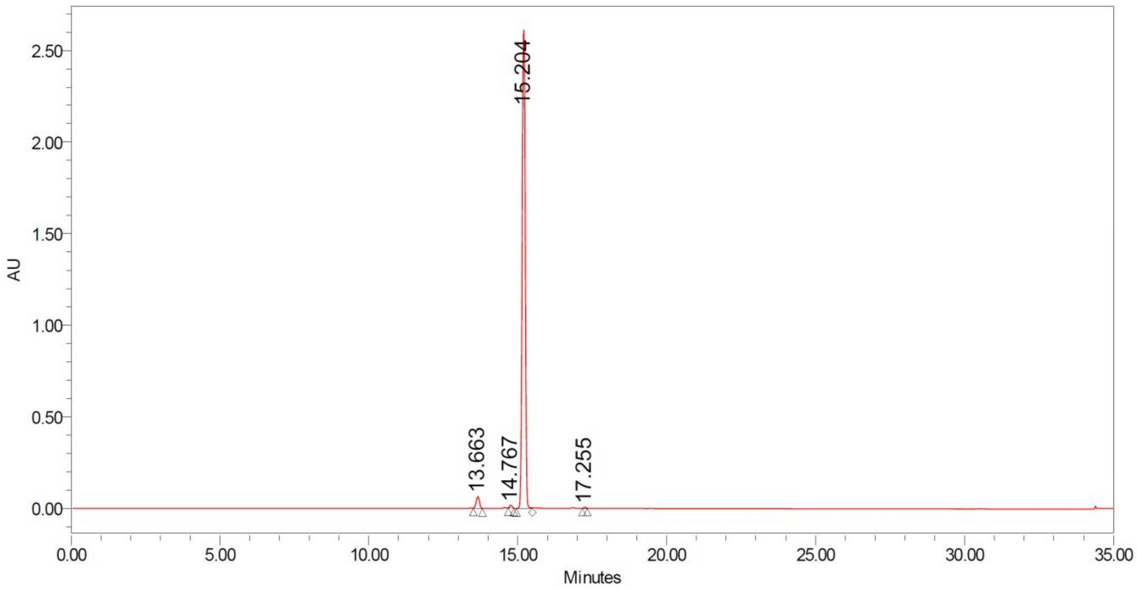

	RT	Area	% Area	Height
1	13.663	482603	2.47	63729
2	14.767	89520	0.46	15539
3	15.204	18898221	96.91	2610016
4	17.255	31176	0.16	6176

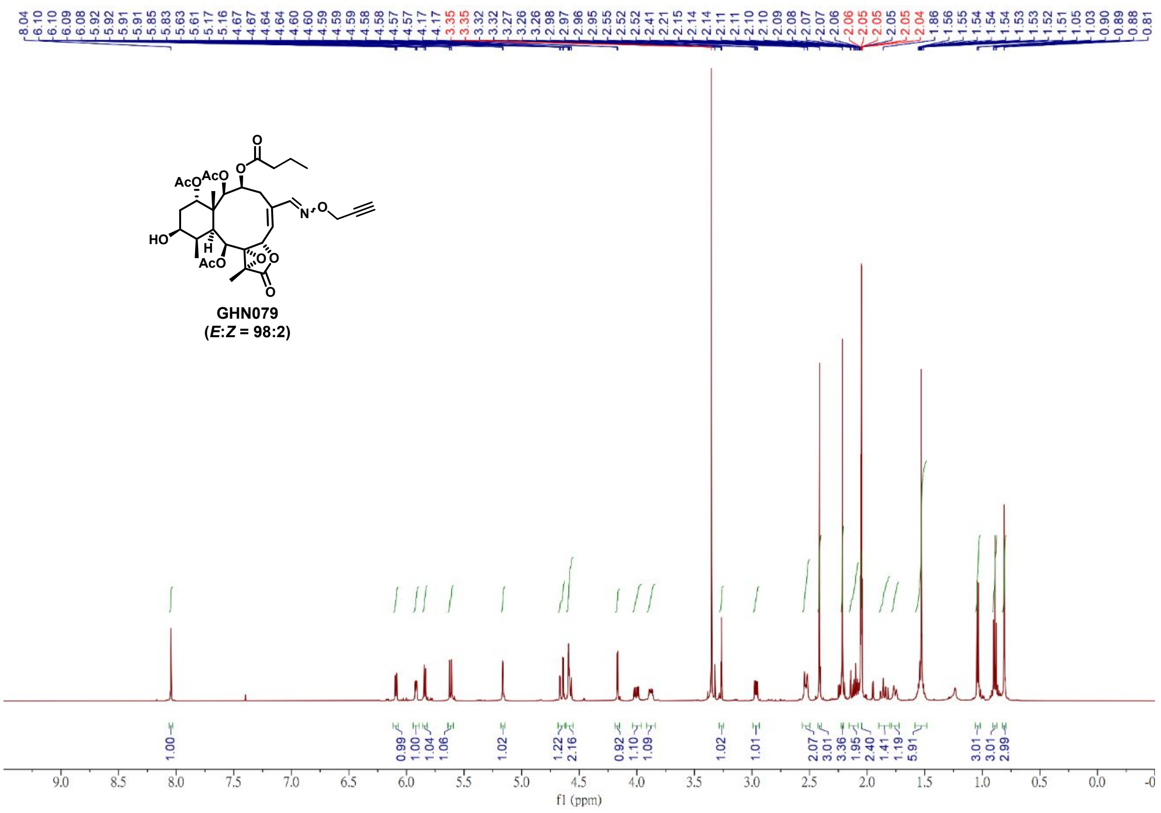

^1H NMR (600 MHz, $(CD_3)_2CO$, -40 ^oC) of **GHN079**.

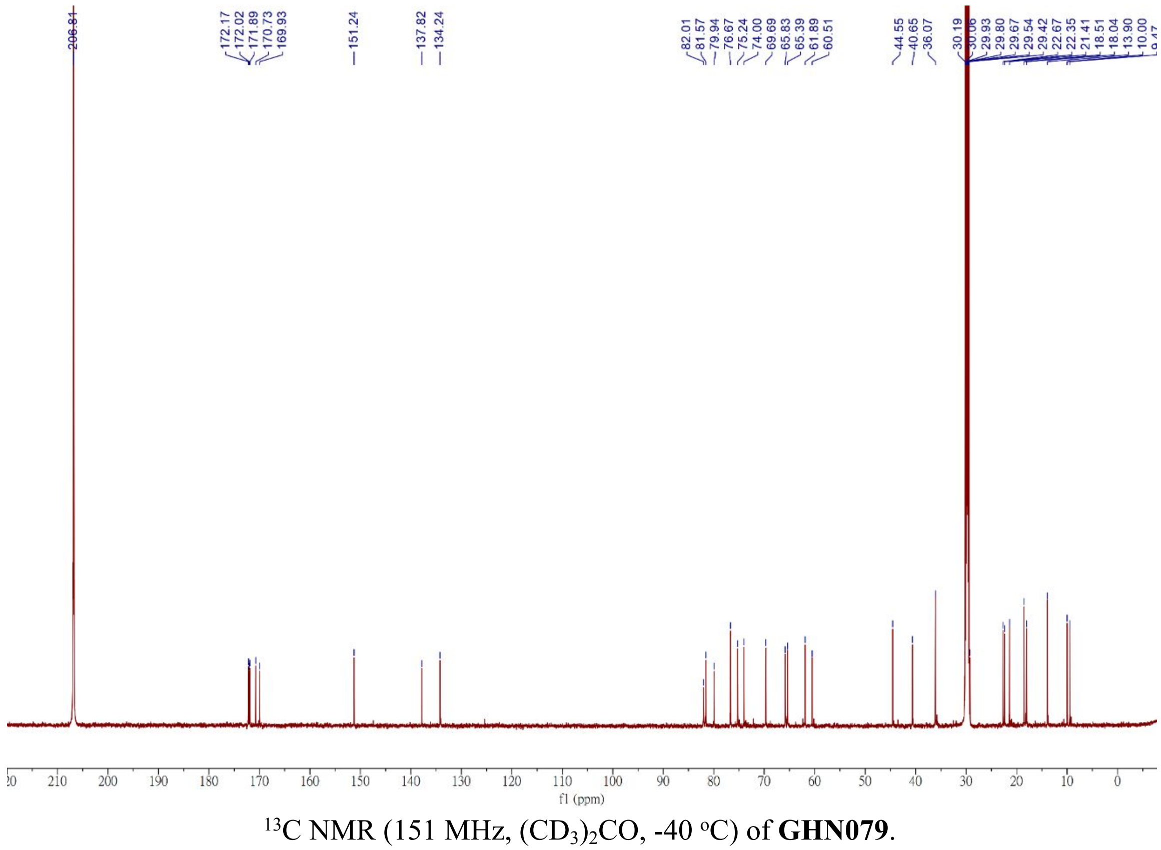

^{13}C NMR (151 MHz, $(CD_3)_2CO$, -40 ^oC) of **GHN079**.

UPLC purity spectra of **GHN079**. (λ = 254 nm)

UPLC system: Waters Acquity UPLC/BSM with photodiode array detector.

Column: Waters Acquity BEH-C18, 50 × 2.1 mm, 1.7 μm particle size.

Mobile phase: Acetonitrile/H_2O (10-90%) containing with 2 mM ammonium acetate and 0.1% formic acid.

Flow rate: 0.6 mL/min.

Run time: 6.5 mins.

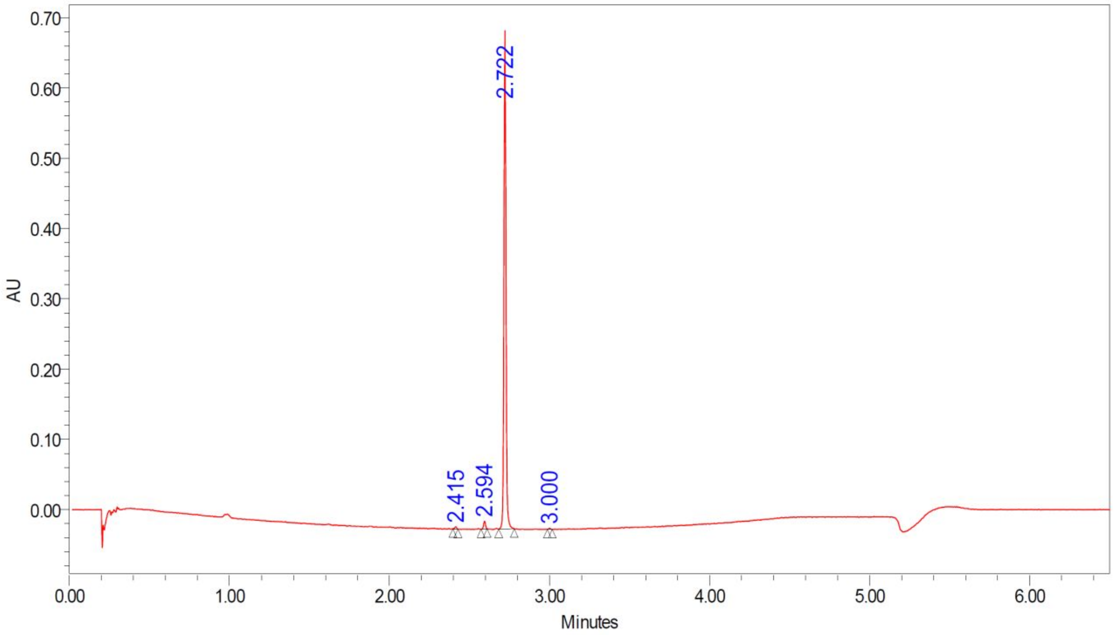

	RT	Height	Area	% Area
1	2.415	3124	2766	0.43
2	2.594	11357	9215	1.45
3	2.722	709383	622369	97.86
4	3.000	1828	1597	0.25

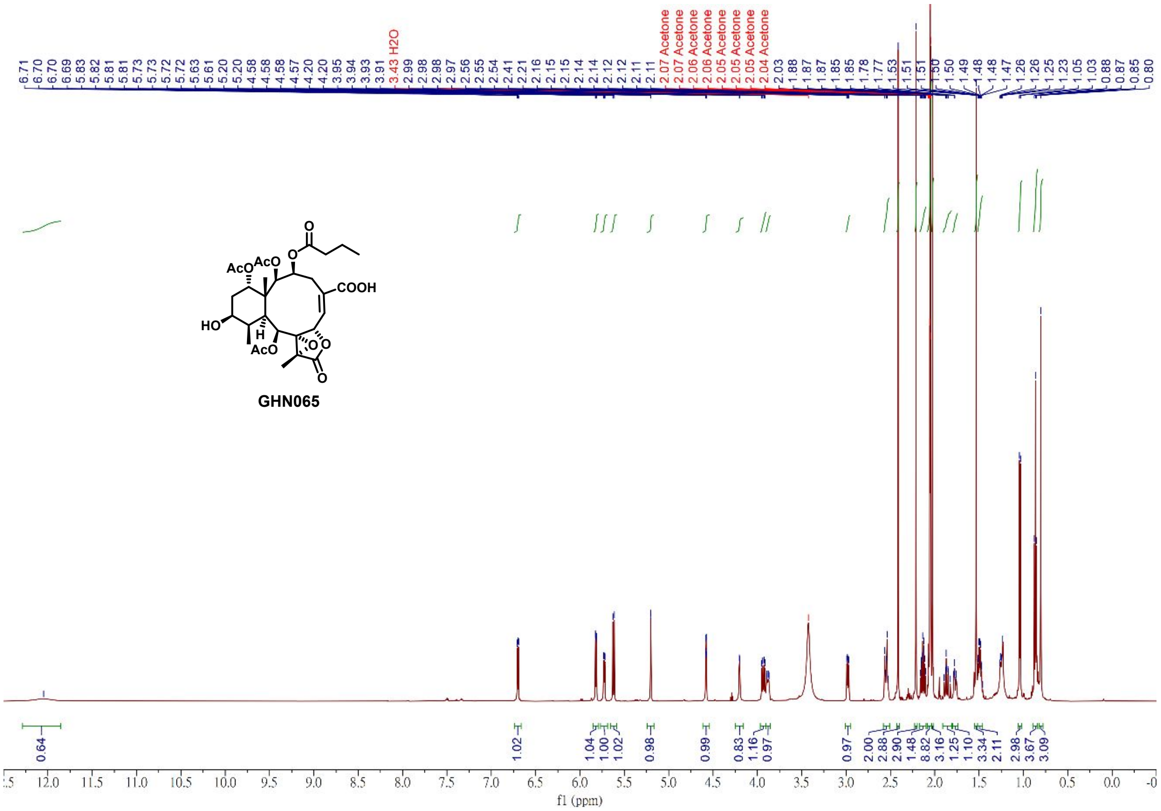

^{1}H NMR (600 MHz, $(CD_3)_2CO$, -40 ^{o}C) of **GHN065**.

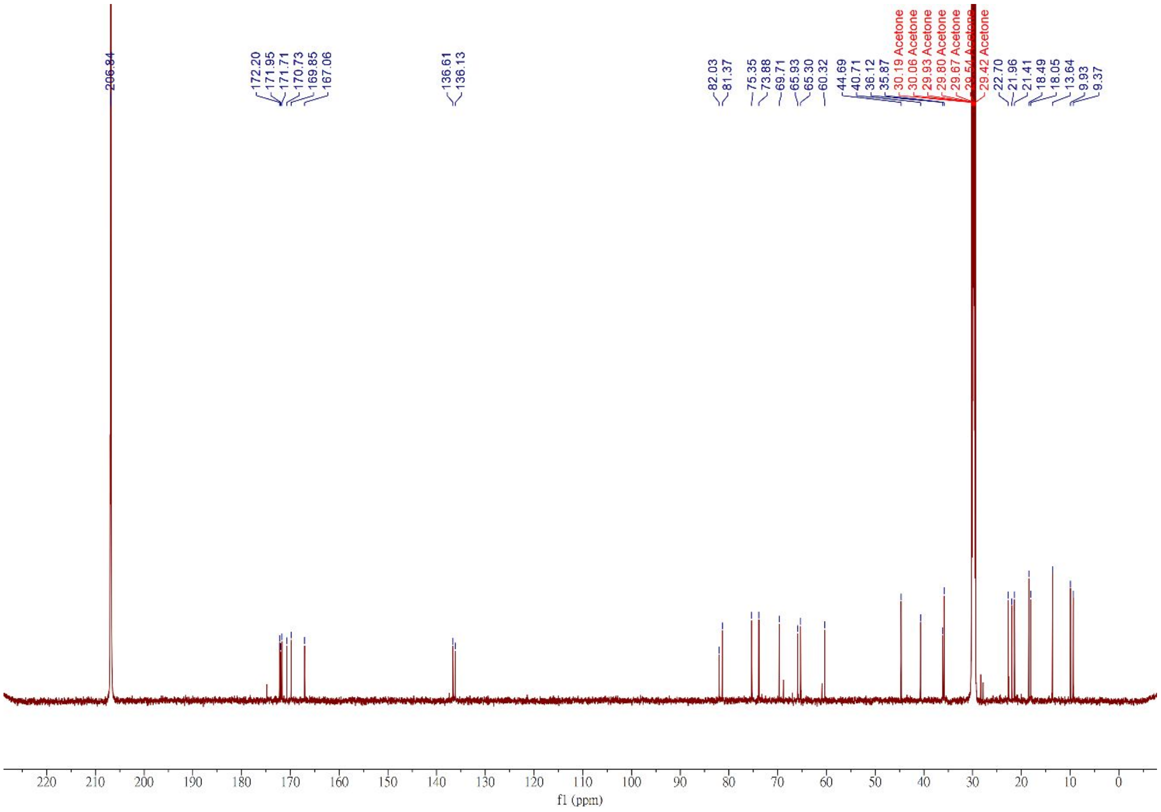

^{13}C NMR (151 MHz, $(CD_3)_2CO$, -40 ^{o}C) of **GHN065**.

HPLC purity spectra of **GHN065**.

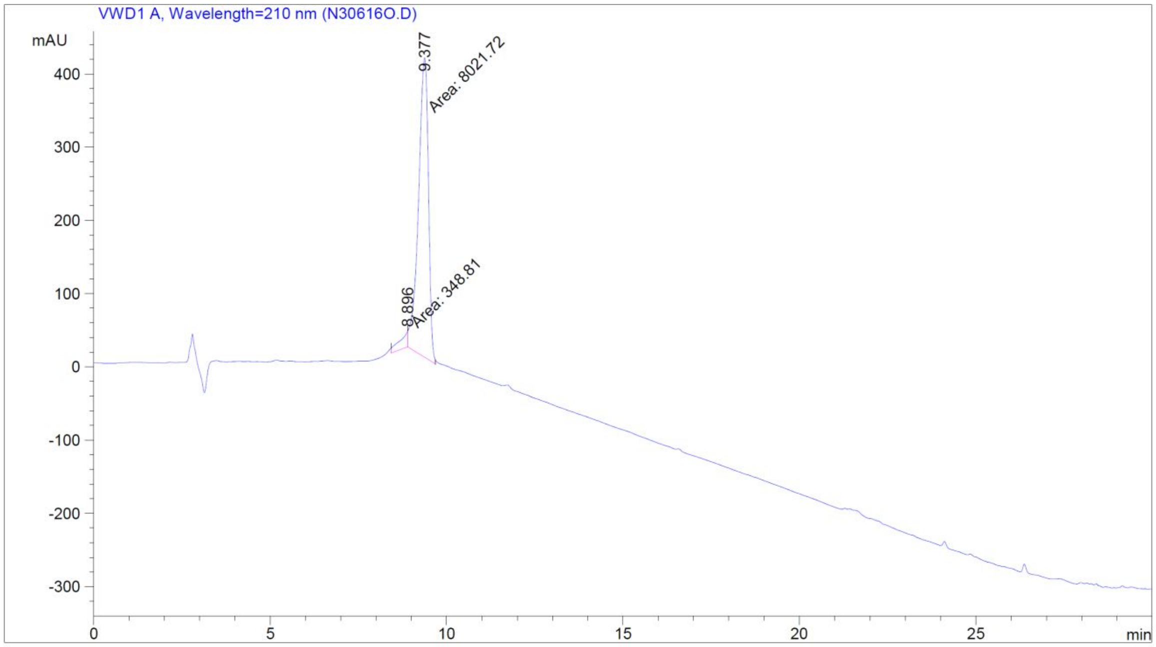


```
=====================================================================
                         Area Percent Report
=====================================================================

Sorted By             :      Signal
Multiplier            :      1.0000
Dilution              :      1.0000
Use Multiplier & Dilution Factor with ISTDs


Signal 1: VWD1 A, Wavelength=210 nm

Peak RetTime Type  Width     Area      Height     Area
  #   [min]        [min]  mAU    *s   [mAU   ]      %
----|-------|----|-------|----------|----------|--------|
   1   8.896 MM    0.2753  348.81039   21.11827   4.1671
   2   9.377 MM    0.3269 8021.71924  408.96432  95.8329

Totals :                  8370.52963  430.08259

 Results obtained with enhanced integrator!
=====================================================================
                          *** End of Report ***
```

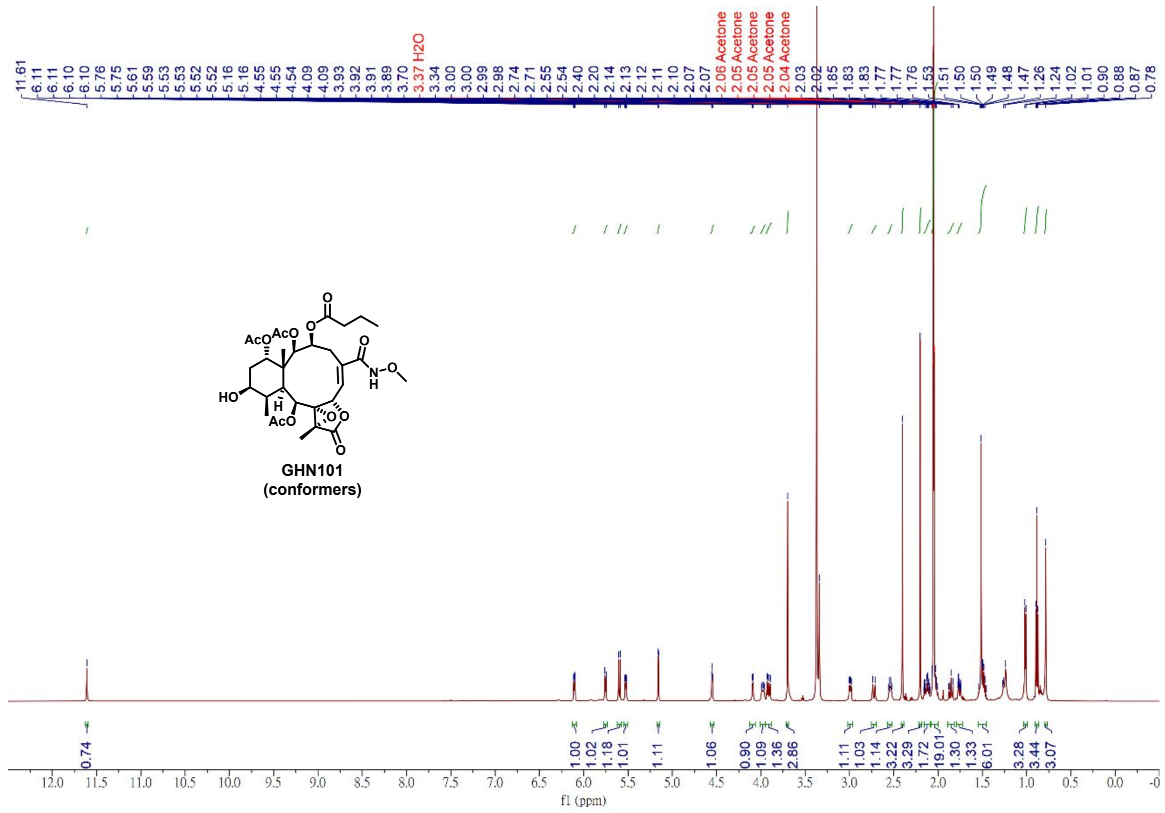

^{1}H NMR (600 MHz, $(CD_3)_2CO$, -40 °C) of **GHN101**.

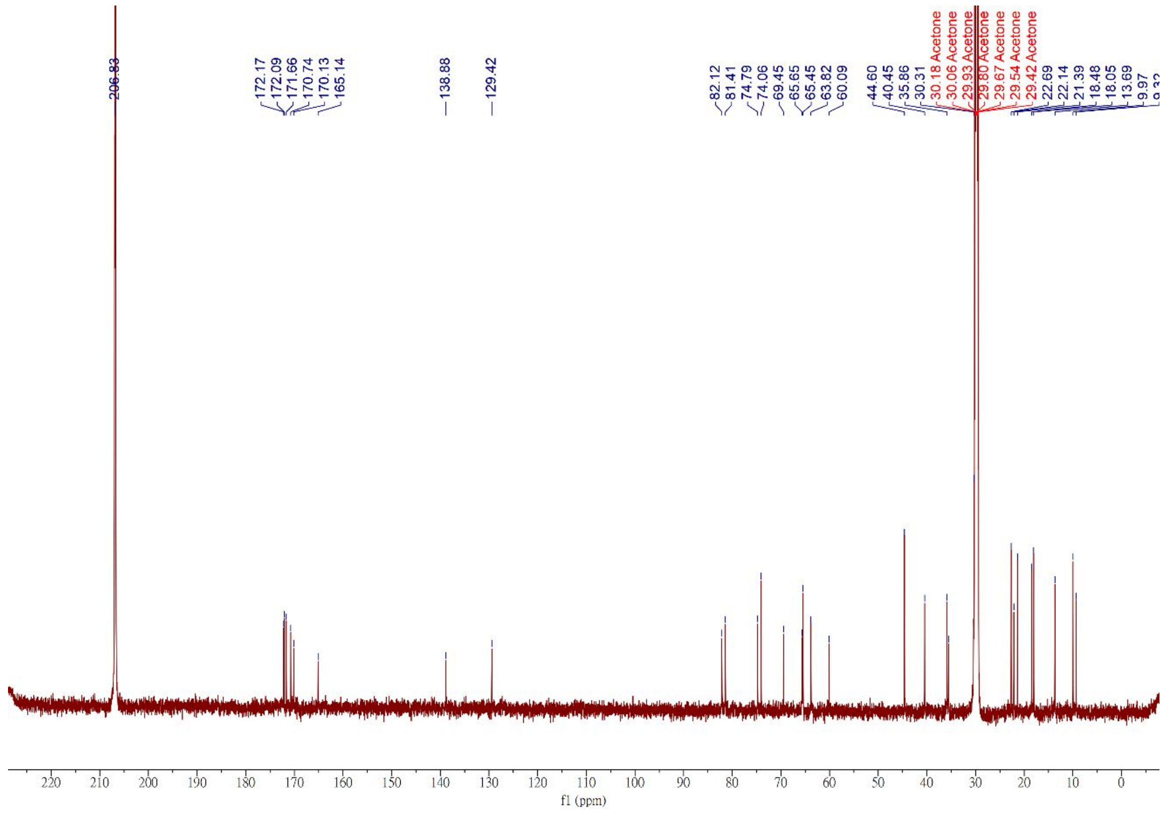

^{13}C NMR (151 MHz, $(CD_3)_2CO$, -40 °C) of **GHN101**.

HPLC purity spectra of **GHN101.**

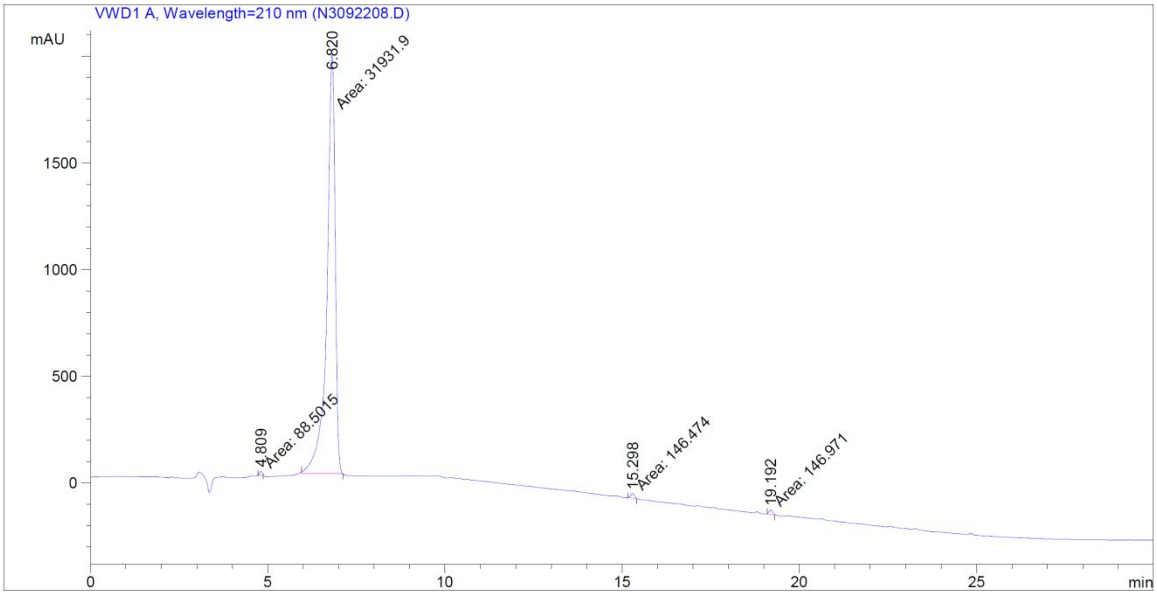


```
=====================================================================
                          Area Percent Report
=====================================================================

Sorted By             :      Signal
Multiplier            :      1.0000
Dilution              :      1.0000
Use Multiplier & Dilution Factor with ISTDs


Signal 1: VWD1 A, Wavelength=210 nm

Peak RetTime Type  Width     Area      Height     Area
  #   [min]        [min]  mAU   *s   [mAU   ]       %
----|-------|----|-------|----------|----------|--------|
   1   4.809 MM    0.0860   88.50153   17.15259   0.2739
   2   6.820 MM    0.2709 3.19319e4 1964.39771  98.8180
   3  15.298 MM    0.1169  146.47406   20.88943   0.4533
   4  19.192 MM    0.1203  146.97078   20.36591   0.4548

Totals :                  3.23138e4  2022.80563

 Results obtained with enhanced integrator!
=====================================================================
                          *** End of Report ***
```

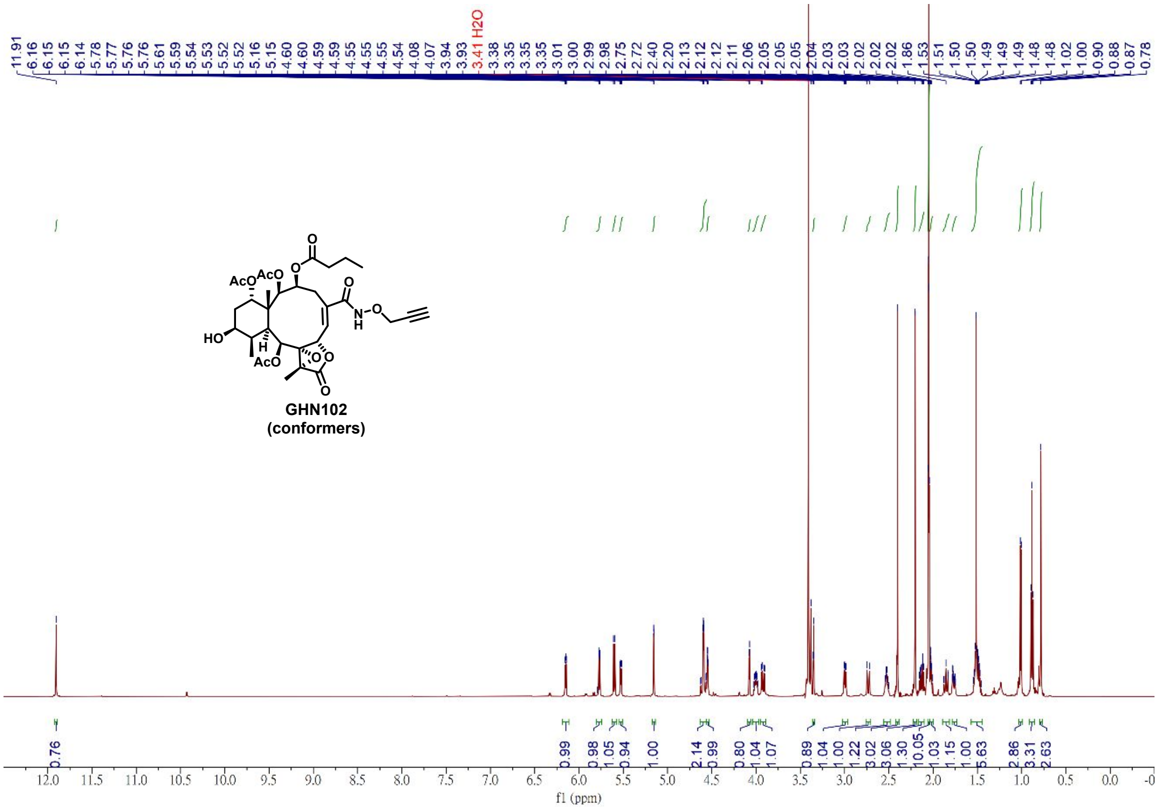

^{1}H NMR (600 MHz, $(CD_3)_2CO$, -40 ^{o}C) of **GHN102**.

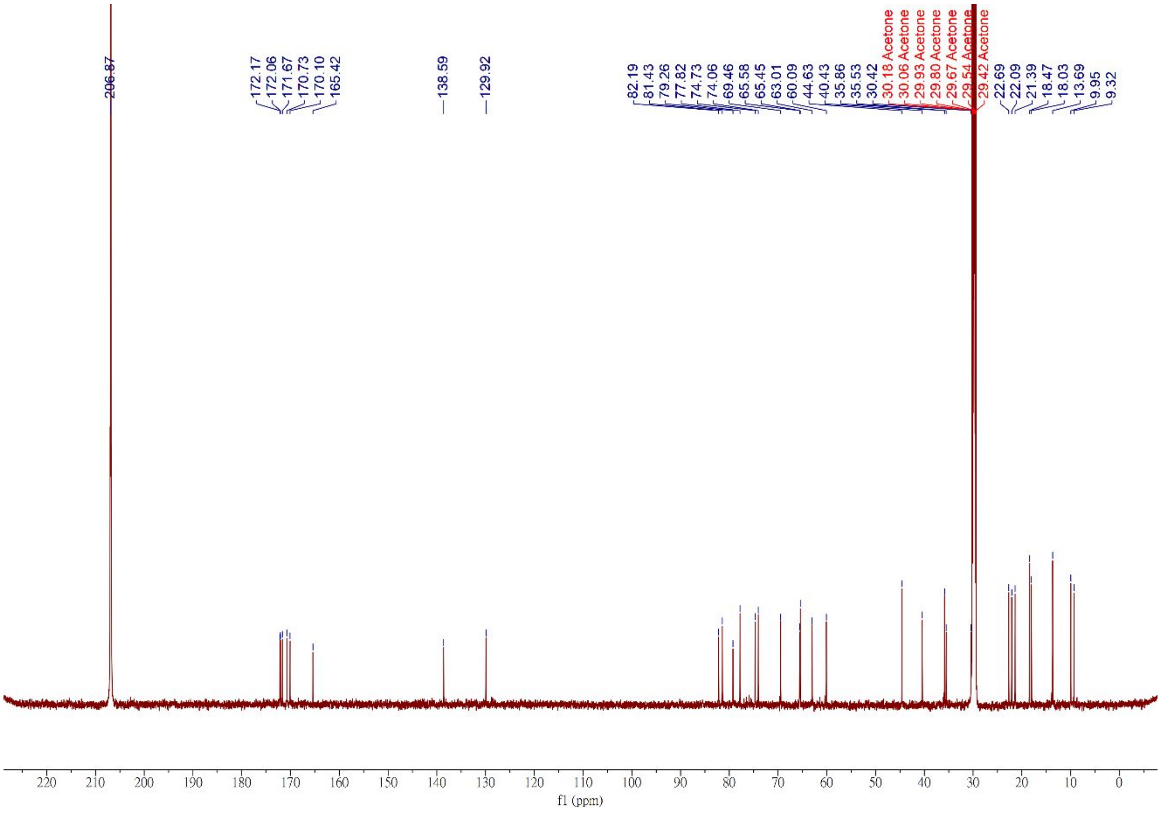

^{13}C NMR (151 MHz, $(CD_3)_2CO$, -40 ^{o}C) of **GHN102**.

HPLC purity spectra of **GHN102**.

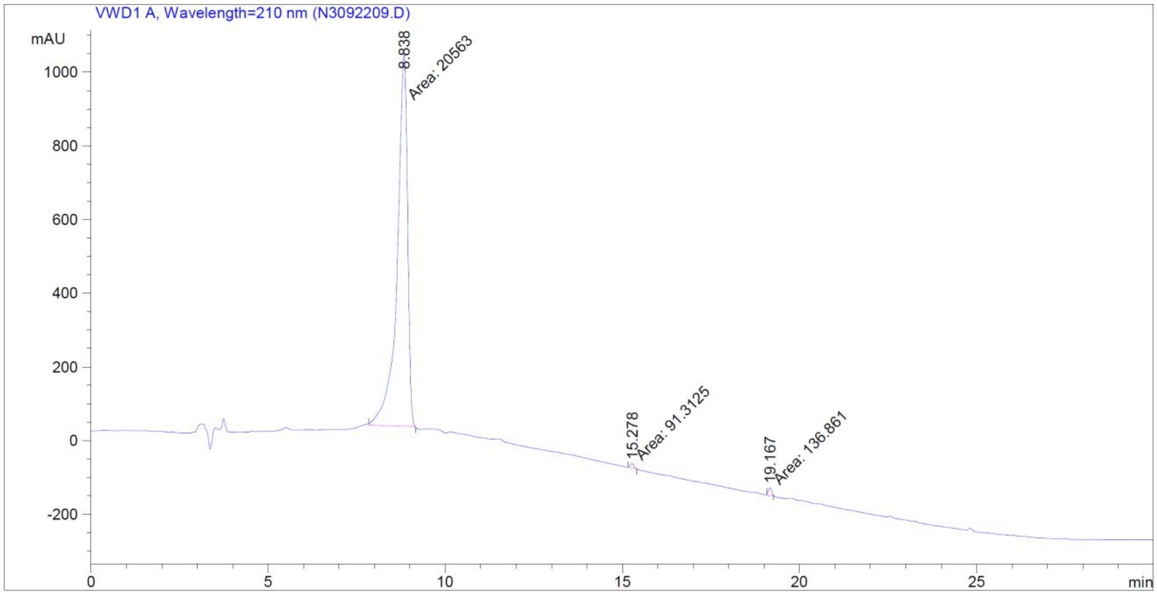


```
=====================================================================
                         Area Percent Report
=====================================================================

Sorted By             :      Signal
Multiplier            :      1.0000
Dilution              :      1.0000
Use Multiplier & Dilution Factor with ISTDs


Signal 1: VWD1 A, Wavelength=210 nm
```

Peak #	RetTime [min]	Type	Width [min]	Area mAU *s	Height [mAU]	Area %
1	8.838	MM	0.3393	2.05630e4	1009.92078	98.9025
2	15.278	MM	0.1231	91.31251	12.36668	0.4392
3	19.167	MM	0.1072	136.86092	21.27391	0.6583

```
Totals :                  2.07911e4  1043.56137

 Results obtained with enhanced integrator!
=====================================================================
                          *** End of Report ***
```

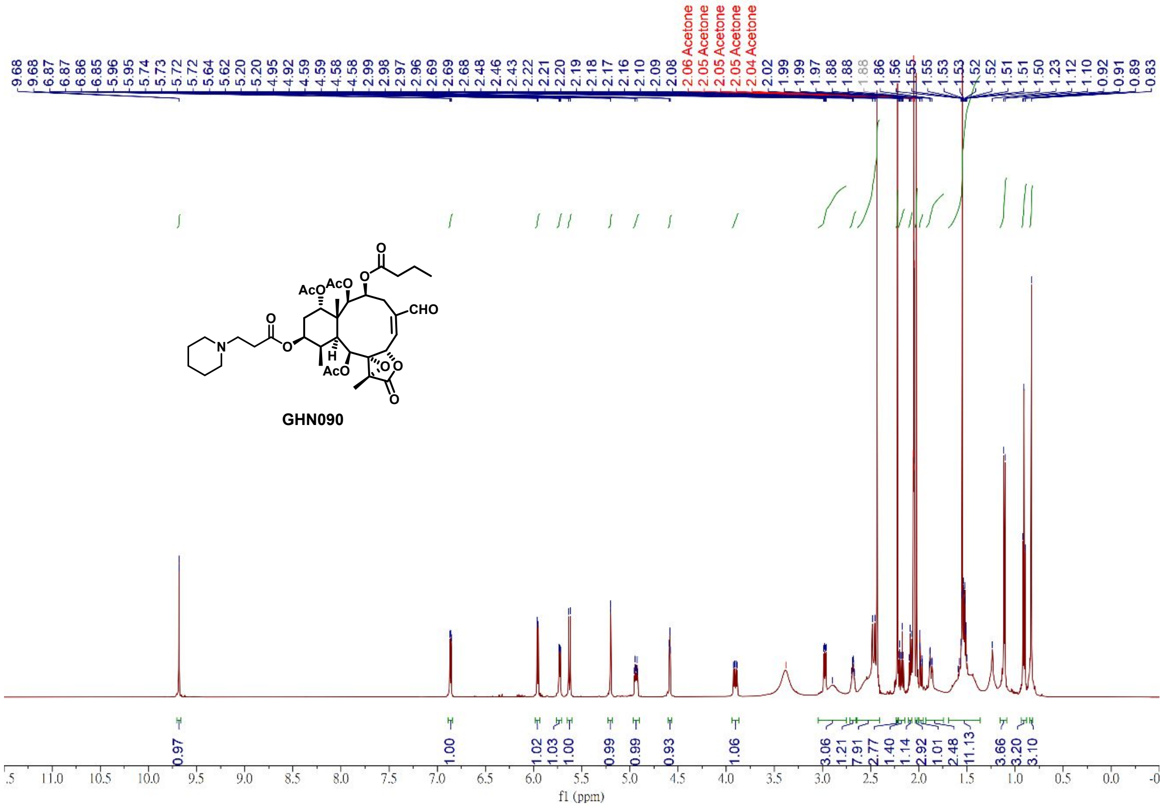

^{1}H NMR (600 MHz, $(CD_3)_2CO$, -40 °C) of **GHN090**.

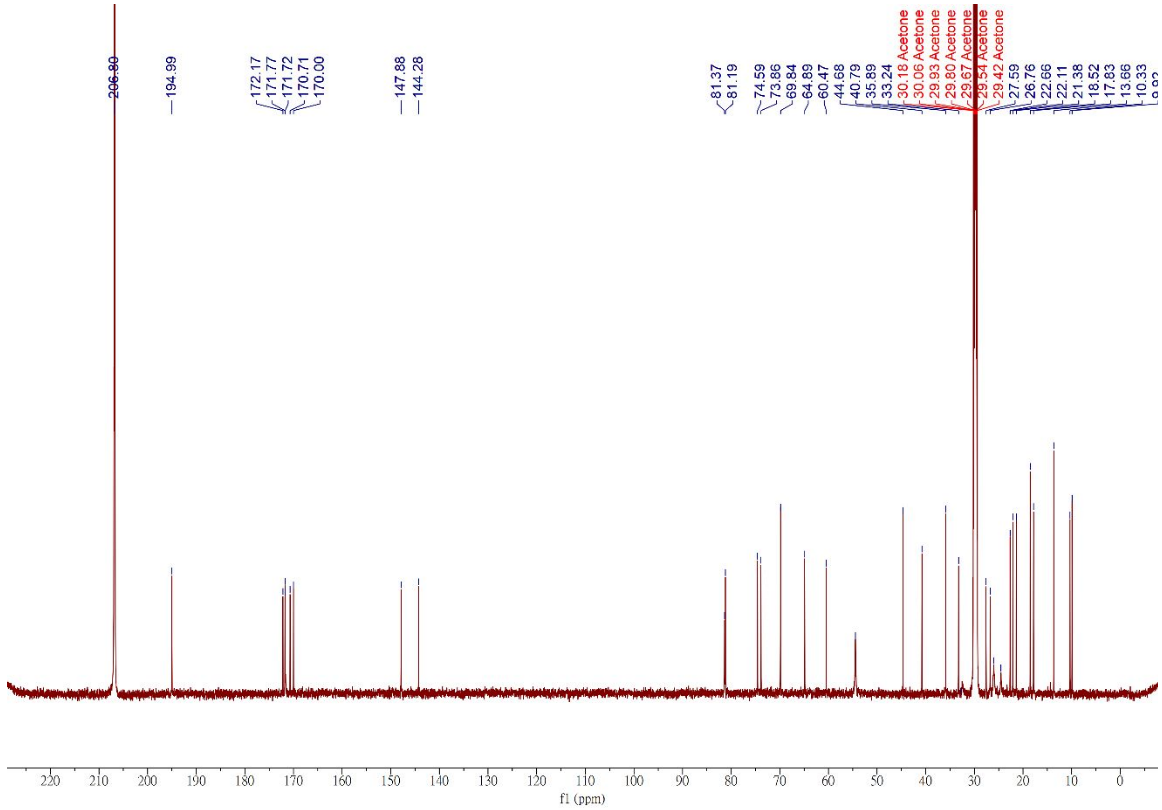

^{13}C NMR (151 MHz, $(CD_3)_2CO$, -40 °C) of **GHN090**.

HPLC purity spectra of **GHN090**. (λ = 210 nm)

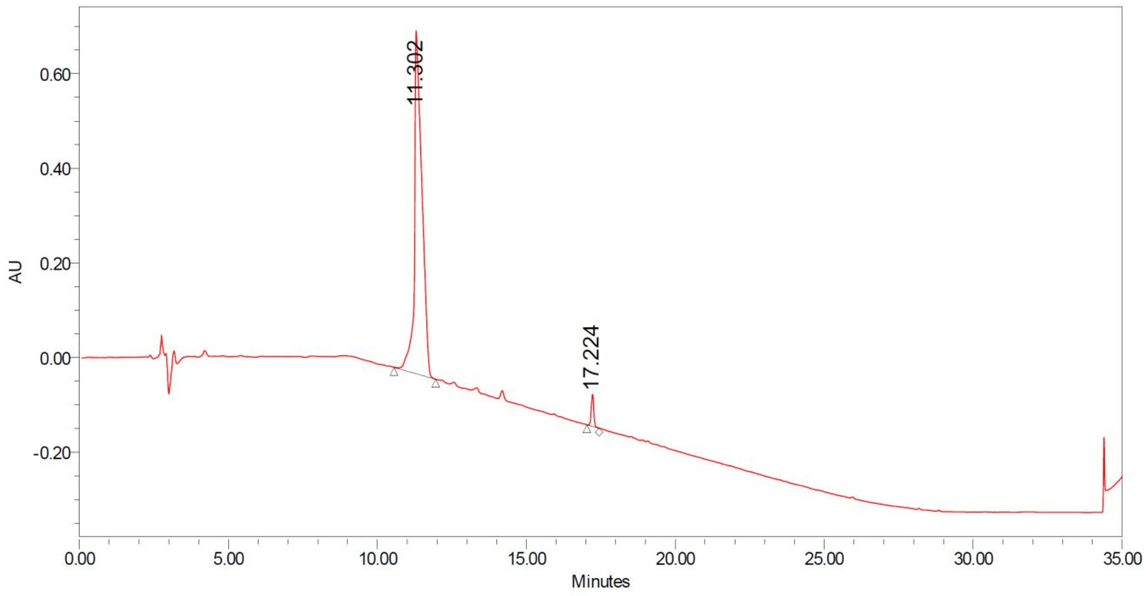

	RT	Area	% Area	Height
1	11.302	12713891	96.96	725049
2	17.224	398671	3.04	67767

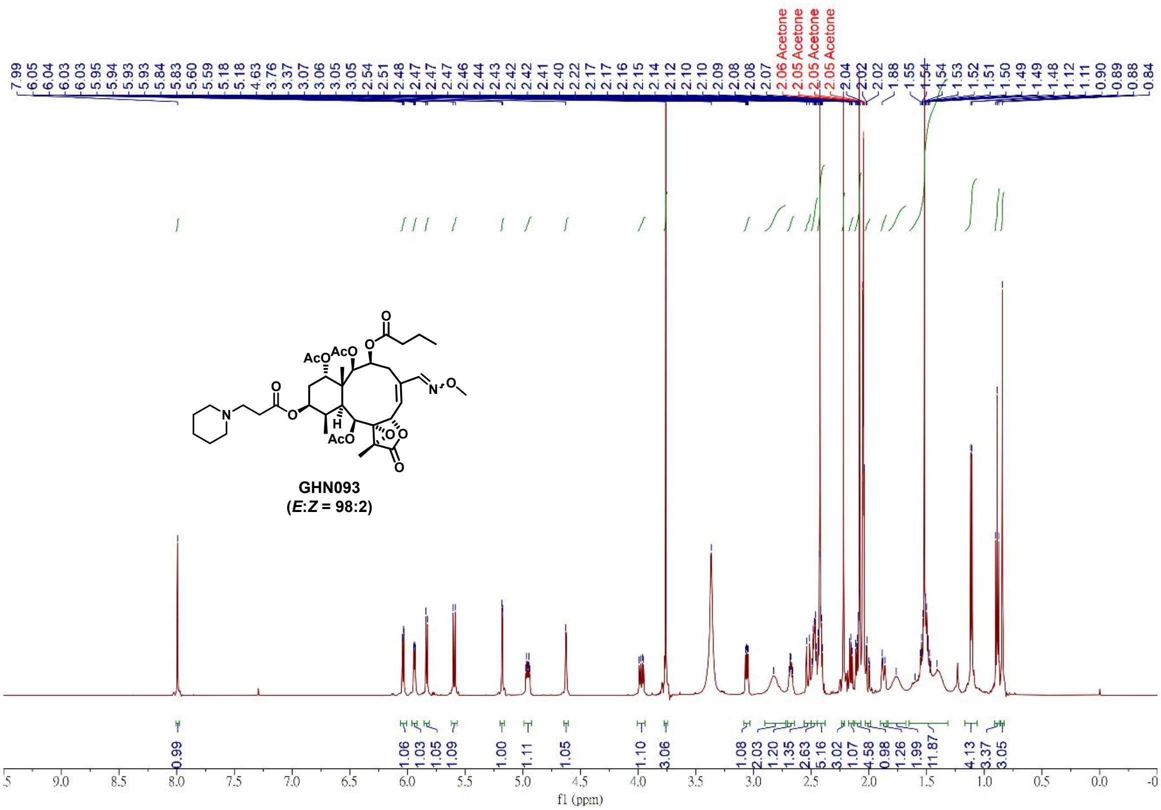

^{1}H NMR (600 MHz, $(CD_3)_2CO$, -40 ^{o}C) of **GHN093**.

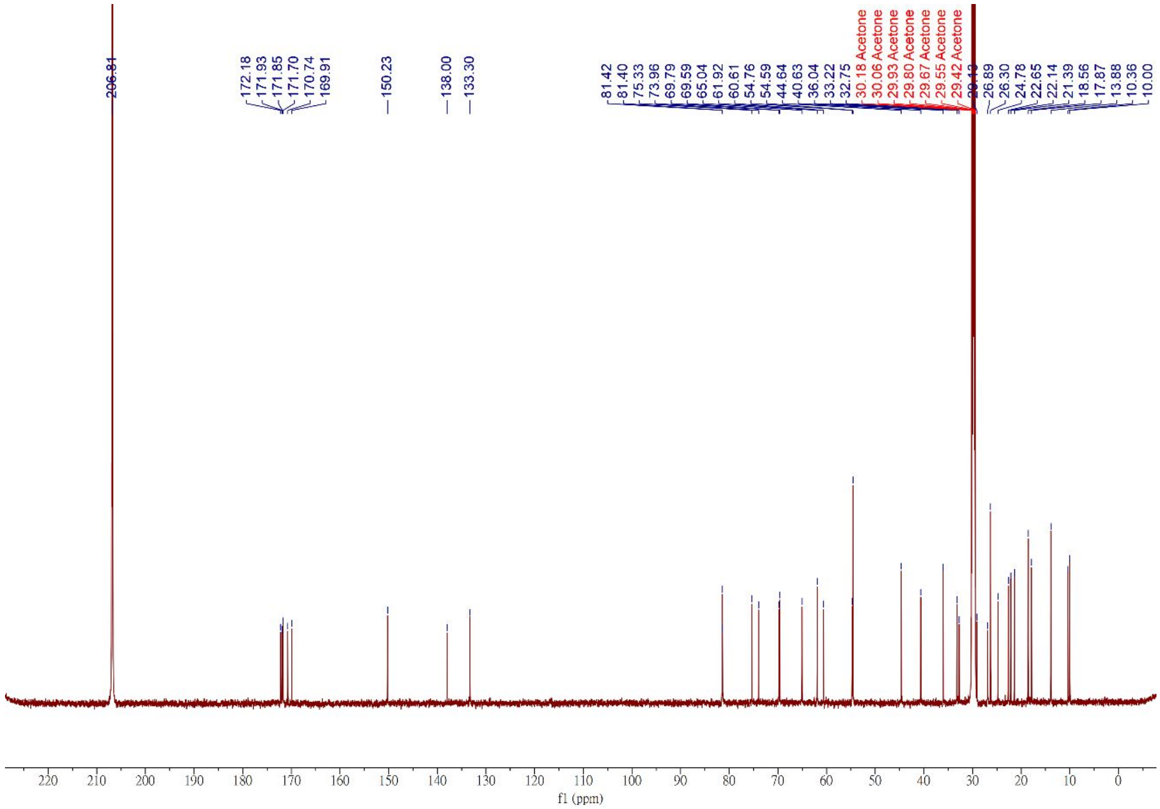

^{13}C NMR (151 MHz, $(CD_3)_2CO$, -40 ^{o}C) of **GHN093**.

UPLC purity spectra of **GHN093**. (λ = 254 nm)

UPLC system: Waters Acquity UPLC/BSM with photodiode array detector.

Column: Waters Acquity BEH-C18, 50 × 2.1 mm, 1.7 μm particle size.

Mobile phase: Acetonitrile/H_2O (10-90%) containing with 2 mM ammonium acetate and 0.1% formic acid.

Flow rate: 0.6 mL/min.

Run time: 6.5 mins.

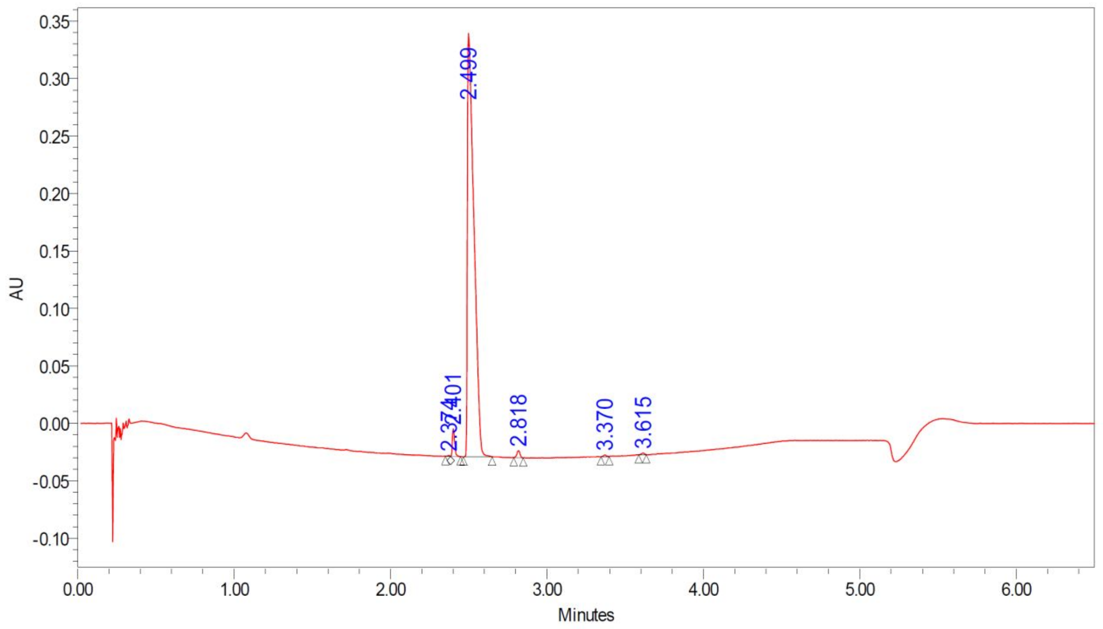

	RT	Height	Area	% Area
1	2.374	822	874	0.08
2	2.401	24100	24804	2.24
3	2.499	368694	1070263	96.58
4	2.818	6103	8421	0.76
5	3.370	1416	1832	0.17
6	3.615	1459	1947	0.18

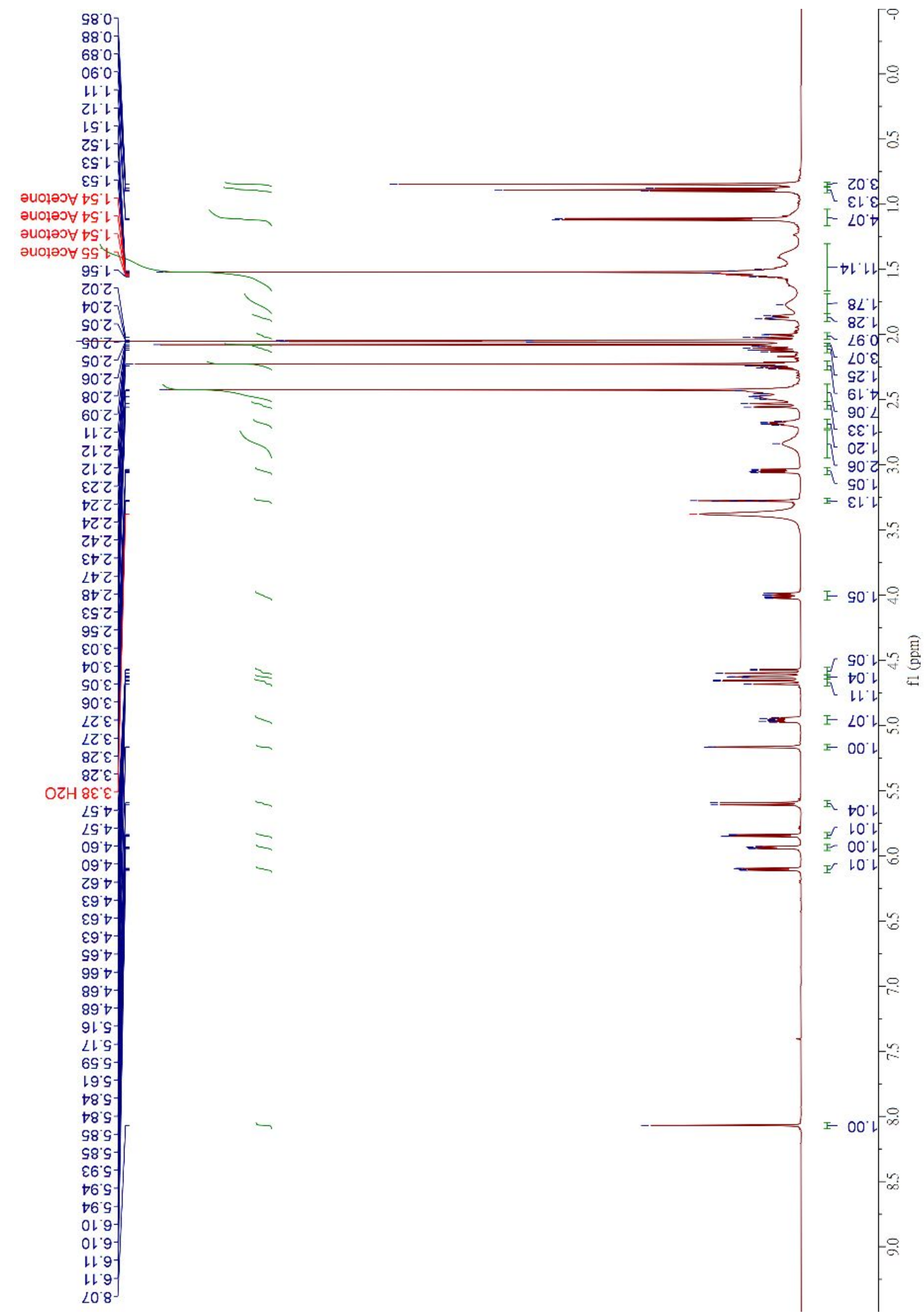

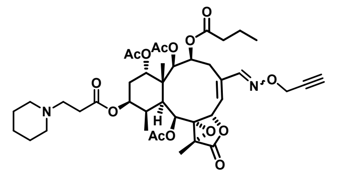

GHN105
(*E*:*Z* = 98:2)

^{1}H NMR (600 MHz, $(CD_3)_2CO$, -40 °C) of **GHN105**.

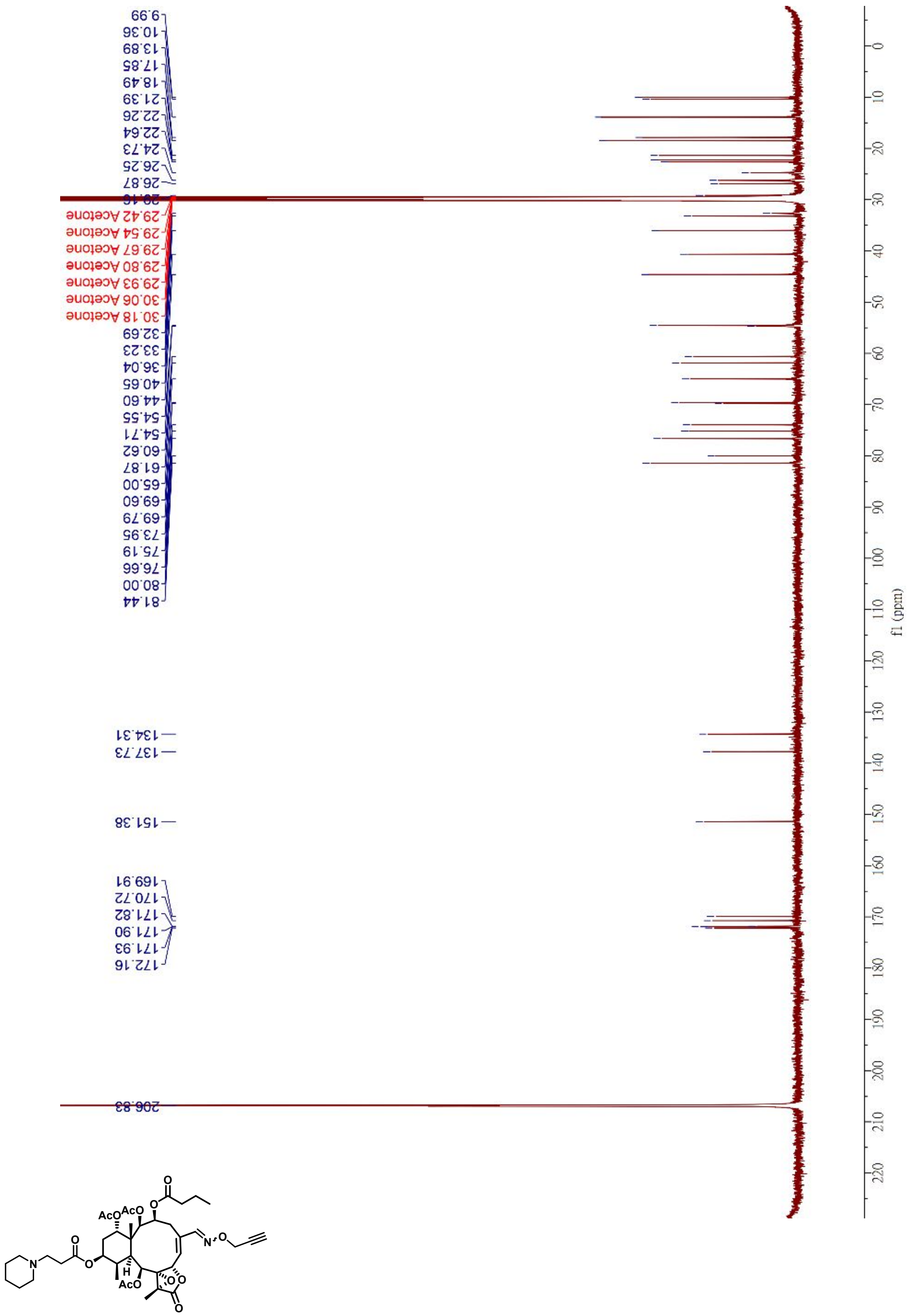

GHN105
(*E*:*Z* = 98:2)

^{13}C NMR (151 MHz, $(CD_3)_2CO$, -40 ^{o}C) of **GHN105**.

UPLC purity spectra of **GHN105**. (λ = 254 nm)

UPLC system: Waters Acquity UPLC/BSM with photodiode array detector.

Column: Waters Acquity BEH-C18, 50 × 2.1 mm, 1.7 μm particle size.

Mobile phase: Acetonitrile/H_2O (10-90%) containing with 2 mM ammonium acetate and 0.1% formic acid.

Flow rate: 0.6 mL/min.

Run time: 6.5 mins.

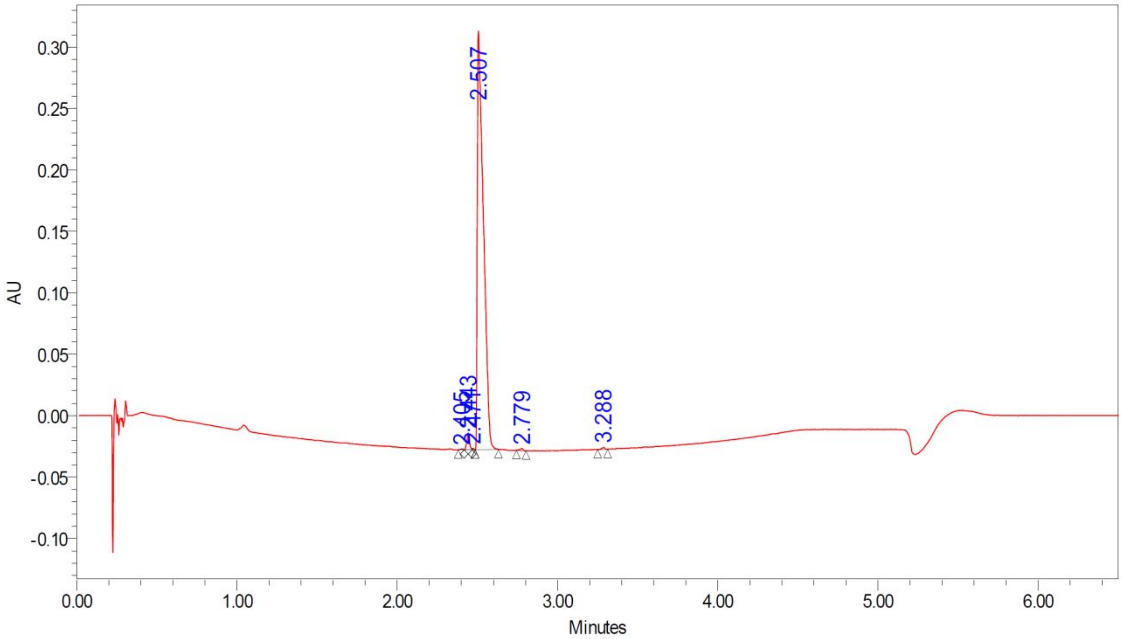

	RT	Height	Area	% Area
1	2.405	731	857	0.09
2	2.443	13860	15821	1.71
3	2.471	1227	837	0.09
4	2.507	341004	903396	97.61
5	2.779	1800	2577	0.28
6	3.288	1617	2057	0.22

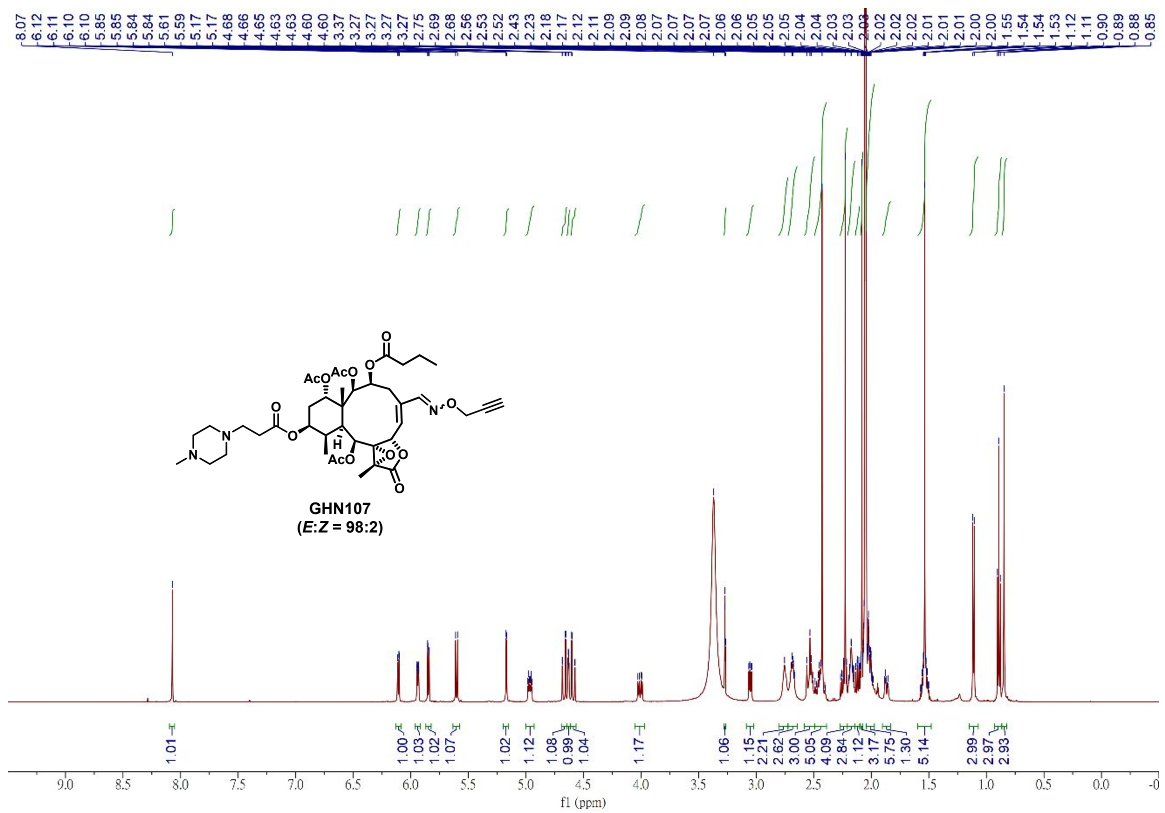

^{1}H NMR (600 MHz, $(CD_3)_2CO$, -40 °C) of **GHN107**.

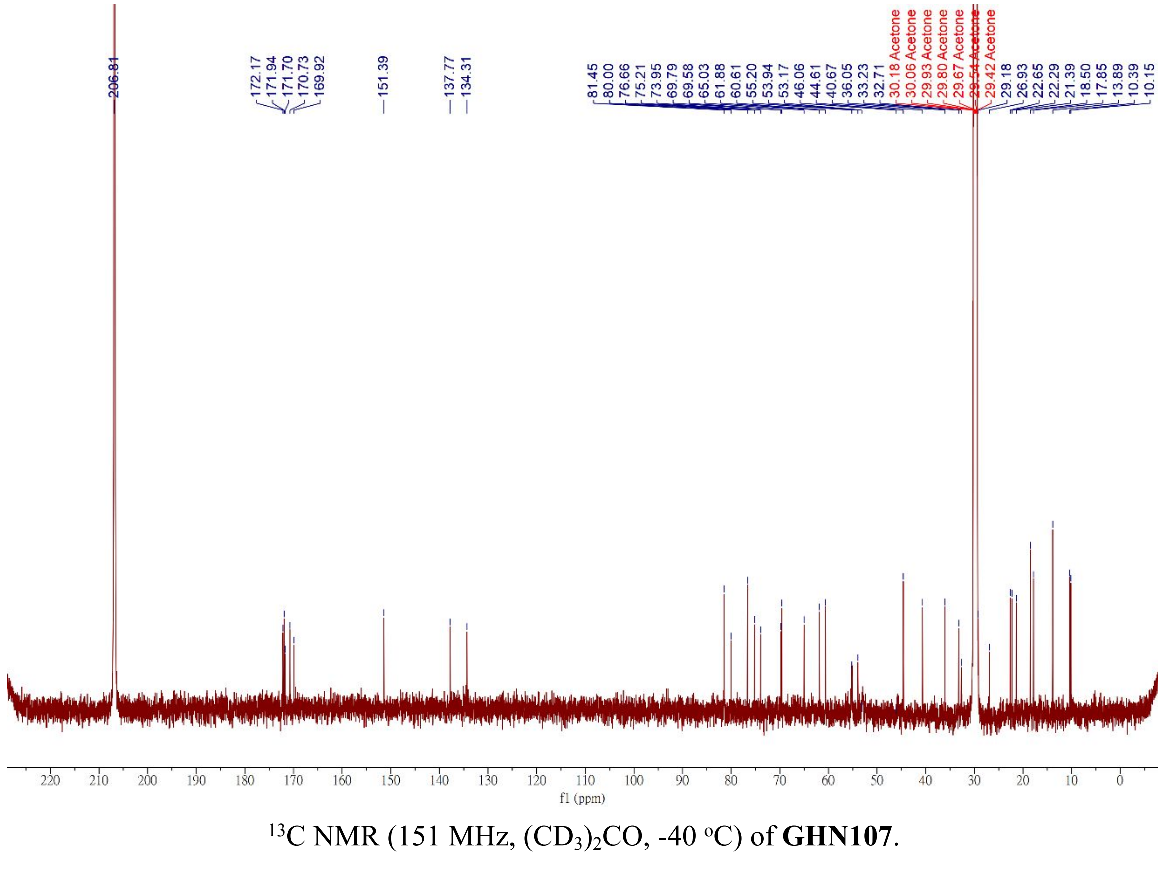

^{13}C NMR (151 MHz, $(CD_3)_2CO$, -40 °C) of **GHN107**.

UPLC purity spectra of **GHN107**. (λ = 254 nm)

UPLC system: Waters Acquity UPLC/BSM with photodiode array detector.

Column: Waters Acquity BEH-C18, 50 × 2.1 mm, 1.7 μm particle size.

Mobile phase: Acetonitrile/H_2O (10-90%) containing with 2 mM ammonium acetate and 0.1% formic acid.

Flow rate: 0.6 mL/min.

Run time: 6.5 mins.

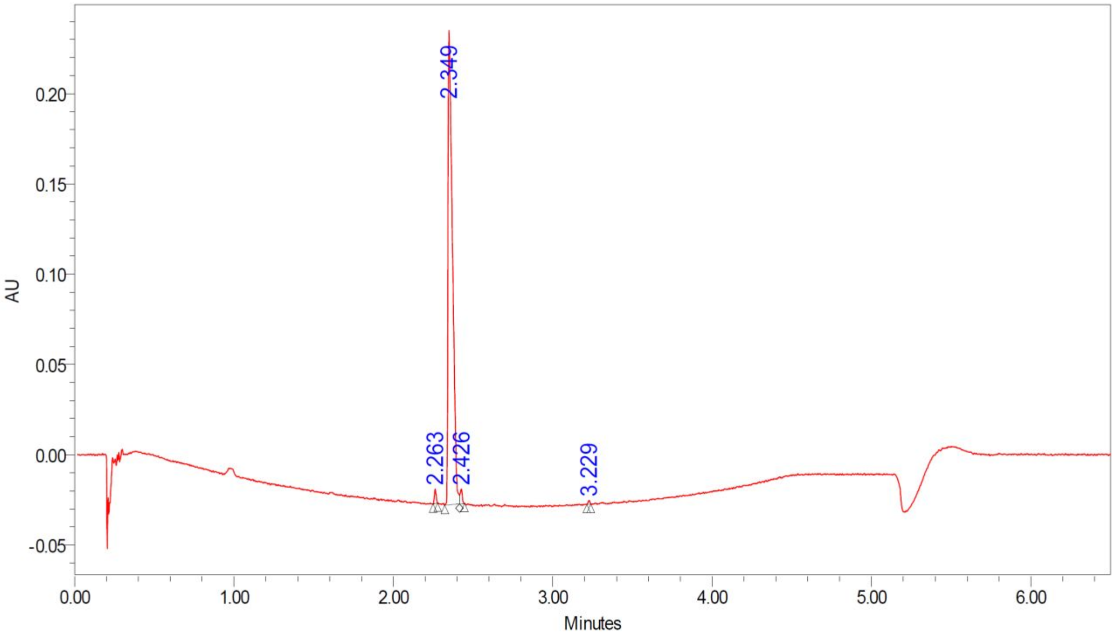

	RT	Height	Area	% Area
1	2.263	8054	6018	1.15
2	2.349	262769	507262	97.00
3	2.426	8213	7983	1.53
4	3.229	2143	1675	0.32

Supplementary References

(1) Zhou, Y. M.; Shao, C. L.; Huang, H.; Zhang, X. L.; Wang, C. Y. New briarane-type diterpenoids from gorgonian Ellisella dollfusi from the South China Sea. *Nat Prod Res* **2014**, *28* (1), 7-11. DOI: 10.1080/14786419.2013.827191.
